# Acid-Switchable Synthesis of Trifluoromethylated Triazoles and Isoxazoles via Reaction of CF_3_-Ynones with NaN_3_: DFT Study of the Reaction Mechanism

**DOI:** 10.3390/ijms232314522

**Published:** 2022-11-22

**Authors:** Vasiliy M. Muzalevskiy, Zoia A. Sizova, Mikhail S. Nechaev, Valentine G. Nenajdenko

**Affiliations:** 1 Department of Chemistry, Lomonosov Moscow State University, 119899 Moscow, Russia; 2 A. V. Topchiev Institute of Petrochemical Synthesis, Russian Academy of Sciences, 119991 Moscow, Russia

**Keywords:** CF_3_-ynones, sodium azide, triazoles, isoxazoles, CF_3_-group

## Abstract

A detailed study of the reaction of CF_3_-ynones with NaN_3_ was performed. It was found that the reaction permits the selective synthesis of either 4-trifluoroacetyltriazoles or 5-CF_3_-isoxazoles. The chemoselectivity of the reaction was switchable via acid catalysis. The reaction of CF_3_-ynones with NaN_3_ in EtOH produced high yields of 4-trifluoroacetyltriazoles. In contrast, the formation of 5-CF_3_-isoxazoles was observed under catalysis by acids. This acid-switchable procedure can be performed at sub-gram scale. The possible reaction mechanism was supported by DFT calculations. The synthetic utility of the prepared 4-trifluoroacetyltriazoles was demonstrated.

## 1. Introduction

The chemistry of fluorinated compounds is a rapidly developing area of modern organic chemistry [1,2,3,4]. Owing to their unique physicochemical and biological properties, organofluorine compounds are used intensively as construction materials, components of liquid crystalline compositions, agrochemicals and pharmaceuticals [5,6,7,8,9,10]. Nowadays about 20% of currently used drugs [11,12,13,14,15,16,17,18,19] and agrochemicals [20,21,22,23] contain at least one fluorine atom. Heterocycles are also attractive for medicinal chemistry due to their various biological activities. Thus, numerous drugs and agrochemicals are heterocyclic compounds [24,25,26,27,28,29]. About 59% of small-molecule drugs approved by the US FDA contain a nitrogen heterocycle in their structure [30]. As a result, the search for novel effective methodologies for the synthesis of fluorinated heterocycles has been an important task in recent decades [31,32,33].

**Figure 1:**
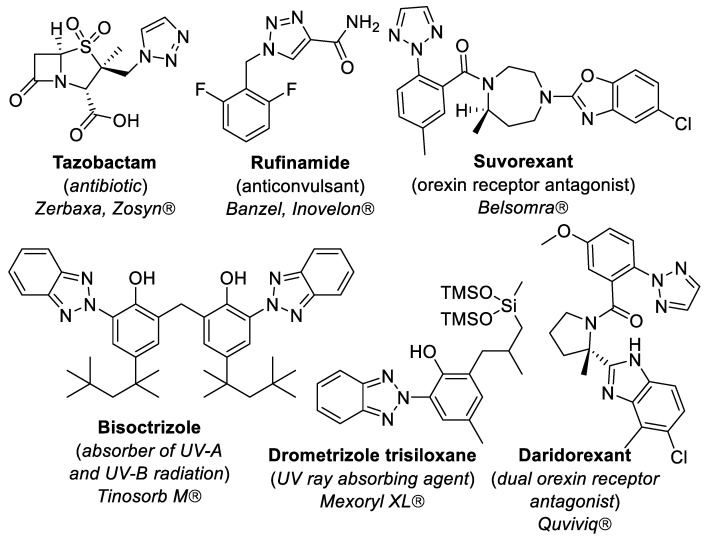
1,2,3-Triazole-containing marketed drugs.

The 1,2,3-triazole scaffold has been intensively investigated in recent decades, boosted by the discovery of CuAAC–RuAAC reactions (metal-catalyzed alkyne–azide cycloaddition) [34]. Many triazoles were found to exhibit useful practical properties and found applications as agrochemicals, pigments, metal chelators, photostabilizers and corrosion inhibitors [35]. N-2-Aryl-1,2,3-triazole derivatives behave as highly efficient UV/blue-light-emitting fluorophores [36,37,38]. Pharmaceutical and therapeutic applications of triazoles are of special interest [39]. Triazoles revealed properties of antimicrobial, anti-inflammatory, antiepileptic, antiviral, antimalarial, anti-Alzheimer’s, antioxidant, antitubercular, antidiabetic, antiobesity, anticonvulsant, anti-cancer, anti-acetylcholinesterase, and antiparasitic agents [40,41,42]. Several 1,2,3-triazole derivatives have been approved by FDA and are already on the market: tazobactam (antibiotic) [43], rufinamide (anticonvulsant) [44], suvorexant (orexin receptor antagonist for the treatment of insomnia) [45], bisoctrizole (absorber of UV-A and UV-B radiation) [46], drometrizole trisiloxane (UV ray absorbing agent) [47] and daridorexant (dual orexin receptor antagonist used to treat insomnia in adults) [48] (Figure 1).

CF_3_-ynones are valuable building-blocks for the synthesis of various fluorinated heterocycles [49,50]. Recently, on the basis of CF_3_-ynones, we elaborated novel approaches to fluorinated diazepines [51], pyrimidines [52], thiophenes [53], triazoles [54], pyrazoles [55,56,57], 1,3-oxazinoquinolines [58,59,60,61] and quinolones [62]. This article reports novel effective acid-switchable pathways to 4-trifluoroacetyltriazoles and 5-trifluoromethylisoxazoles based on the reaction of CF_3_-ynones with sodium azide.

## 2. Results

We envisioned that the reaction of CF_3_-ynones with sodium azide would lead to triazoles **2** via a Michael addition followed by spontaneous cyclization of the intermediates formed. We chose CF_3_-ynone **1a** as a model compound for exploring the cyclization conditions. First, several solvents were tested to search for an optimal reaction medium. It was found that the reaction is extremely sensitive to the nature of a solvent. Thus, the transformation of CF_3_-ynone **1a** in non-polar toluene led to triazole **2a** in a 2% yield only (see Table 1). Better yields of **2a** were observed in more polar acetone, acetonitrile, THF and ethyl acetate (entries 2–5). In the case of most polar aprotic solvents in the row (DMF, DMSO and NMP, entries 6–8) the yields of **2a** increased to about 50%. Eventually, we found that the best yields of **2a** can be obtained in alcohols. Thus, the reactions in methanol and ethanol led to **2a** in 77% and 81% yields, respectively (entries 9–10). It should be noted that full conversion of CF_3_-ynone **1a** was observed in all mentioned solvents. It was also found that the main byproduct of the reaction was isoxazole **3a**, which was formed in small amounts.

**Scheme 1:**
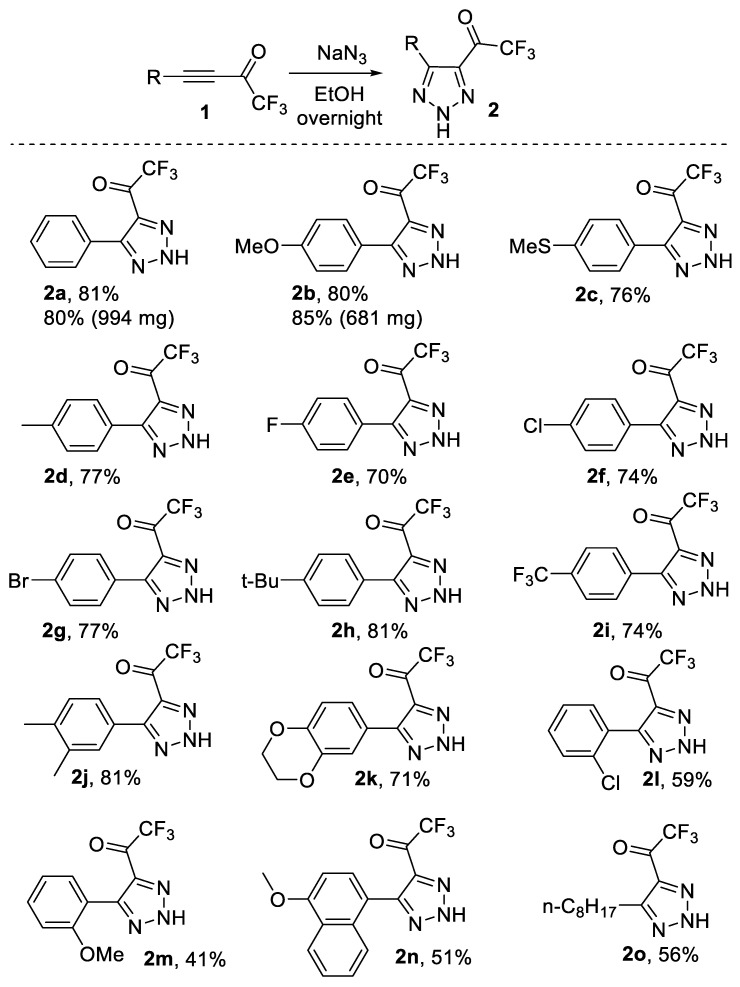
Reactions of CF_3_-ynones **1** with NaN_3_ in ethanol.

Having optimal conditions in hand, we decided to study the scope of the synthesis of trifluoroacylated triazoles. To this end, we performed a series of reactions with various CF_3_-ynones. To our delight, the reaction was found to be very general. The yield of **3** did not depend on electronic nature of the substituents in the aryl ring of CF_3_-ynones to give target triazoles in 70–80% yields (**2a**–**k**). The steric demand was found to be more crucial for the reaction outcome. Thus, the reaction with *ortho*-substituted CF_3_-ynones **1l,m** and naphthalene derivative **1n** produced triazoles in 41–59% yields. Alkyl-substituted ynone **1o** was also successfully involved in the reaction to form triazole **2o** in good yields. We also demonstrated scalability of the method (Scheme 1). Gram-scale synthesis of triazoles **2a,b** was performed to give the products in high yields (80–85%). It should be noted, that NH-triazoles **2** are a novel class of fluorinated triazoles which have not been reported yet. The structure of triazoles **2** was confirmed beyond doubt by NMR, FT-IR and HRMS spectra. Thus, the presence of the trifluoroacetyl group (**2a** as an example) was proven by quadruplets in ^13^C NMR at 174.4 ppm (C=O) and 116.2 ppm (CF_3_) as well as by the signal at 1719 cm^−1^ (C=O) in FT-IR. Two signals in the low field in ^13^C NMR at 147.7 ppm and 136.1 ppm were the quaternary carbons of the triazole moiety. A broad singlet at 12.75 ppm in ^1^H NMR and a broad band near 3000 cm^−1^ in FT-IR confirm the presence of a NH-triazole moiety.

The acidic nature of NH-triazoles is well known (pKa~9.26) [63]. The presence of the electron-withdrawing trifluoroacetyl group made triazoles **2** highly acidic and soluble in water at basic pH. We successfully used this feature of triazoles **2** for their purification. After the evaporation of ethanol, the residue was dispersed between water and a mixture of heptane and ethyl acetate. The organic phase containing all by-products was thrown away and the water phase was acidified to form pure triazole **2**. It should be noted that all solvents used in this protocol are listed as green ones and the amount of waste was minimal. The E-factor calculated for gram scale synthesis of **2a** was 13.6, which is a good value for fine chemicals. Therefore, we can describe the method proposed as a green one.

Next, we focused our attention on isoxazoles **3**, formed as by-products in the reaction of CF_3_-ynones with sodium azide. Isoxazole is another popular scaffold for drug design. These heterocycles exhibit antimicrobial, antiviral, antifungal, anti-tuberculosis, anticancer, anti-inflammatory, analgesic, antipyretic, immunomodulatory, anticonvulsant and anti-diabetic properties [64,65]. Several isoxazole-containing drugs have already been marketed [66].

**Scheme 2:**
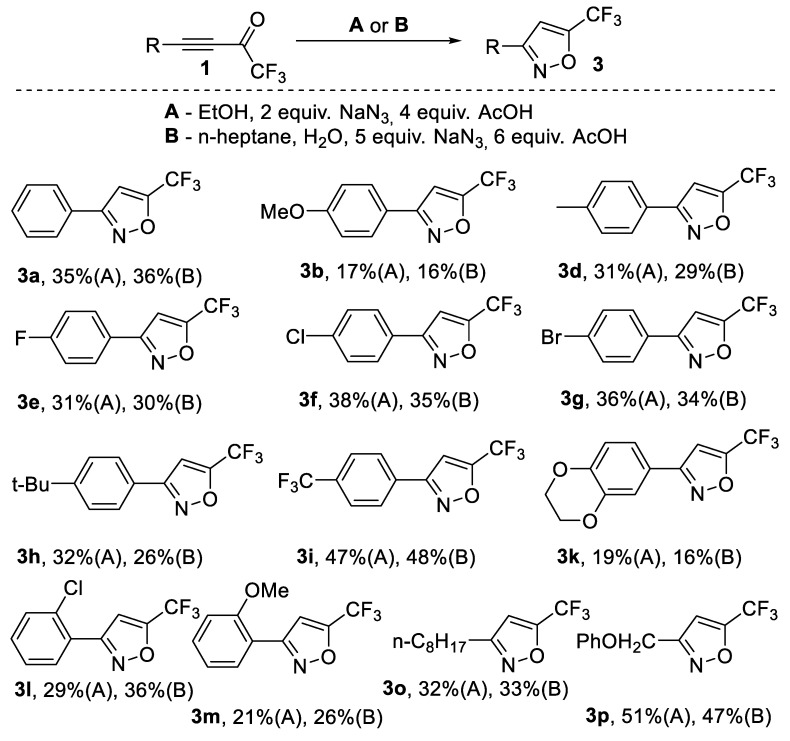
Reactions of ynones **1** with NaN_3_ in ethanol and in *n*-heptane in the presence of acid.

We proposed the possibility of switching the reaction direction from triazole **2** to isoxazole **3**. One can notice that NaN_3_ is a salt that is strongly basic and weakly acidic. As a result, solutions formed by NaN_3_ have a slightly basic pH. We decided to add acid to the reaction in order to neutralize even acidic pH. To our delight, this manipulation permitted us to switch the selectivity of the reaction to make the formation of izoxazole **3** a major reaction path. Thus, the addition of various organic acids such as HCOOH, CH_3_COOH, CH_2_ClCOOH and CF_3_COOH changed the reaction direction in the same solvent (EtOH) to form isoxazole **3**, while triazole **2** became a minor product. Moreover, similar results were obtained in the heptane-H_2_O system. Both conditions gave isoxazole **3a** in isolated yields of 35–36%. A control experiment without the addition of any acid was performed in heptane-H_2_O. It was found, that CF_3_-ynone **1a** was fully consumed and the reaction led to trace amounts of triazole **2a** and isoxazole **3a** along with much tarring. So, the role of acid is crucial in the formation of isoxazole **3a**. Next, we examined several other solvents in the reaction using acetic acid as a catalyst (see Supplementary Materials). Again, the reaction in the presence of acid led to isoxazole **3a** in 35–41% yields (by ^19^F NMR) in MeOH, EtOAc, PhMe, dioxane and TCE (1,1,2-trichloroethylene) (Table S2). The reaction in quite acidic CF_3_CH_2_OH could be performed without the addition of any acids to give isoxazole **3a** in a 37% yield. Eventually, a reaction in acetic acid led to the same yield of isoxazole. It should be noted that the application of stronger acids (MeSO_3_H and HCl) blocks the conversion of CF_3_-ynone in the reaction mixture. Taking into account the list of green solvents, we chose the EtOH and *n*-heptane-water systems as solvents for further investigation of the reaction. Ethanol was chosen because, in this case, there was obviously a possibility of elegantly managing the reaction direction via the simple addition of acetic acid (also a green solvent). The use of n-heptane simplified the isolation and purification of isoxazoles **3**. Thus, after completion of the reaction, isoxazole **3a** was placed in n-heptane (upper phase), while the by-products were mostly present the third phase below water. n-Heptane phase could be easily separated to give a solution of pure isoxazole in most cases (Scheme 2).

Using the above-mentioned optimal conditions for the synthesis of isoxazoles **3**, we carried out a series of reactions with a set of CF_3_-ynones. It was found that unlike in the synthesis of triazoles, the yields of isoxazoles were very sensitive to the electronic nature of the substituents in the aryl ring of CF_3_-ynones. Thus, the CF_3_-ynone with an electron-withdrawing CF_3_ group in the aryl ring produced isoxazole **3i** yields of 48%. In contrast, the reaction with ynones bearing electron-donating alkoxy-groups (**1b,k,m**) led to isoxazoles **3** in lower yields. The yields of other aryl isoxazoles were in the range of 26–36%. Alkyl-substituted ynones **1o,p** were also successfully used in the reaction to give isoxazoles **3o,p** in 33 and 51% yields, respectively (Scheme 2).

**Scheme 3:**
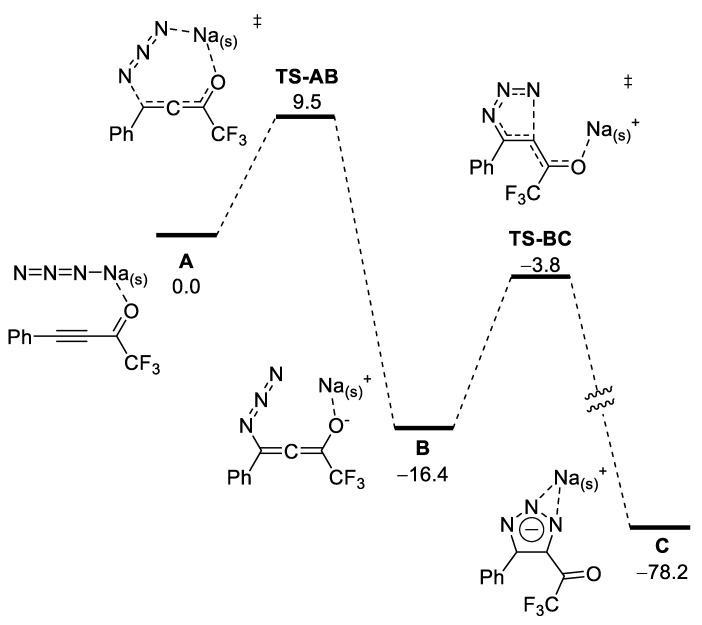
Plausible DFT mechanism of formation of triazoles in absence of acid (numbers are relative Gibbs free energies in kcal/mol; Na_(s)_^+^ = Na(EtOH)_4_^+^).

To rationalize the observed selectivity of the reactions of sodium azide with ynones, we performed DFT modeling (for details, see supporting information). Precomplex **A** consists of ynone, and sodium azide solvated by four ethanol molecules. The addition of azide to the triple bond proceeds via low-lying transition state **TS-AB** to give allene **B**. Then, through transition state **TS-BC,** the triazolate **C** is formed. The reaction is highly exergonic (ΔG = −78.2 kcal/mol). The rate-limiting energy barrier corresponds to **TS-BC** (ΔΔG^≠^ = ΔG(**TS-BC**) − ΔG(**B**) = 12.6 kcal/mol) [67]. Thus, under non-acidic conditions, the reaction proceeds with a very low barrier to give triazole at a rate that is determined mainly by the rate of dissolution of sodium azide in the reaction medium (Scheme 3).

**Scheme 4:**
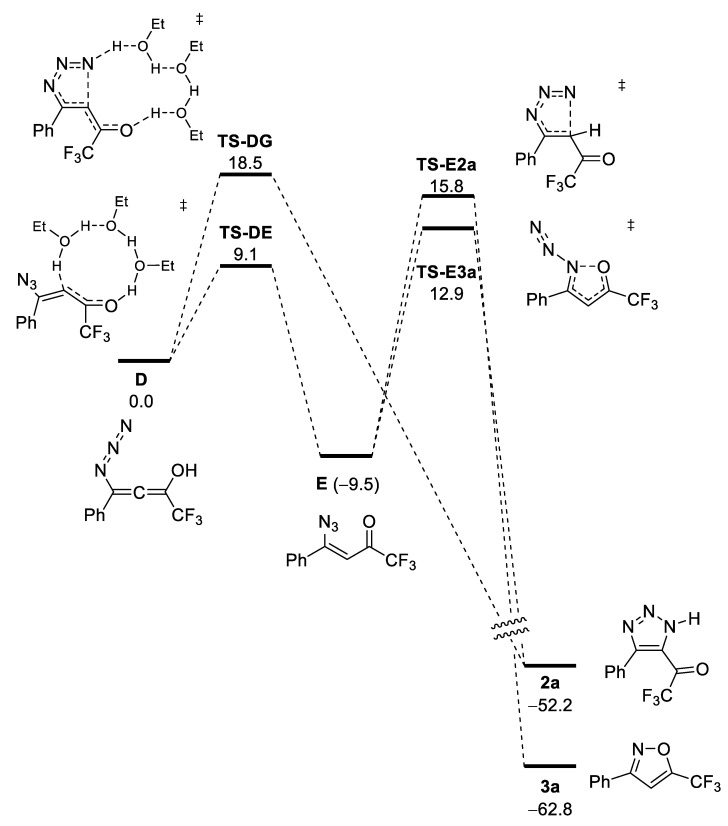
Plausible DFT mechanism of formation of isoxazoles in presence of acid (numbers are relative Gibbs free energies in kcal/mol).

Hydrogen azide and acetic acid have virtually the same pKa values (in water), at 4.75 and 4.76, respectively. It should be expected that in the presence of acetic acid, NaN_3_ and HN_3_ are in equilibrium in the reaction media. Thus, it can be supposed that under such conditions, azide is bound to ynone to give allene **B** in the same manner as in the absence of acetic acid (Scheme 3). Importantly, the allene **B** can be regarded as sodium enolate, which is strongly basic. Thus, we suppose that under acidic conditions, enolate **B** is immediately protonated to give a neutral form of allene **D** (Scheme 4).

Allene **D** can undergo isomerization via transition state **TS-DE** to give azide **E**, or via transition state **TS-DG** to give triazole **2a**. Since **TS-DE** lies 9.4 kcal/mol lower in energy than **TS-DG**, the main route of the reaction is the formation of azide **E**. Isoxazole **3a** is formed via cyclization and the simultaneous release of dinitrogen via transition state **TS-E3a**. Alternatively, triazole **2a** can be formed from **E** via transition state **TS-E2a**. Transition states leading to isoxazole and triazole are rather close in energy; however, **TS-E3a** lies 2.9 kcal/mol lower. Thus, under room temperature conditions, selectivity of the formation of isoxazole:triazole should be expected to be greater than 100:1. Overall the energetic barrier of the formation of isoxazole ΔΔG^≠^ = ΔG(**TS-E3a**) − ΔG(**E**) = 22.4 kcal/mol. Such an energy barrier corresponds well with the observed completion of the reaction within a few hours at r.t.

The DFT calculation can also explain the lower yield of isoxazoles **3**. We believe that only Z-configurated vinylazide **E** can cyclize into izoxazole. Another diastereomer has a disfavored arrangement of the azide group and the trifluoroacetyl moiety. As a result, the formation of polymeric products takes place.

**Scheme 5:**
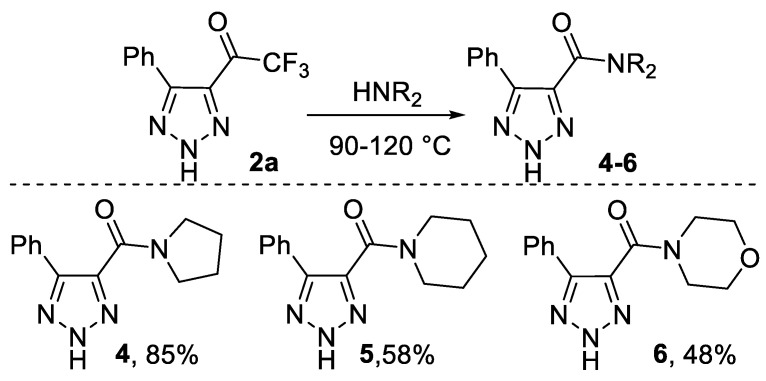
Reactions of triazole **2a** with secondary amines.

It should be noted that N-unsubstituted 4-trifluoroacyl triazoles **2** are a novel class of fluorinated compounds which have not been reported yet. In this article, we started an investigation of the chemical properties of triazoles. We found that trifluoroacetyl triazoles react with secondary amines at elevated temperatures to give the corresponding amides (derivatives of pyrrolidine, piperidine and morpholine) in quite good yields (Scheme 5).

**Scheme 6:**
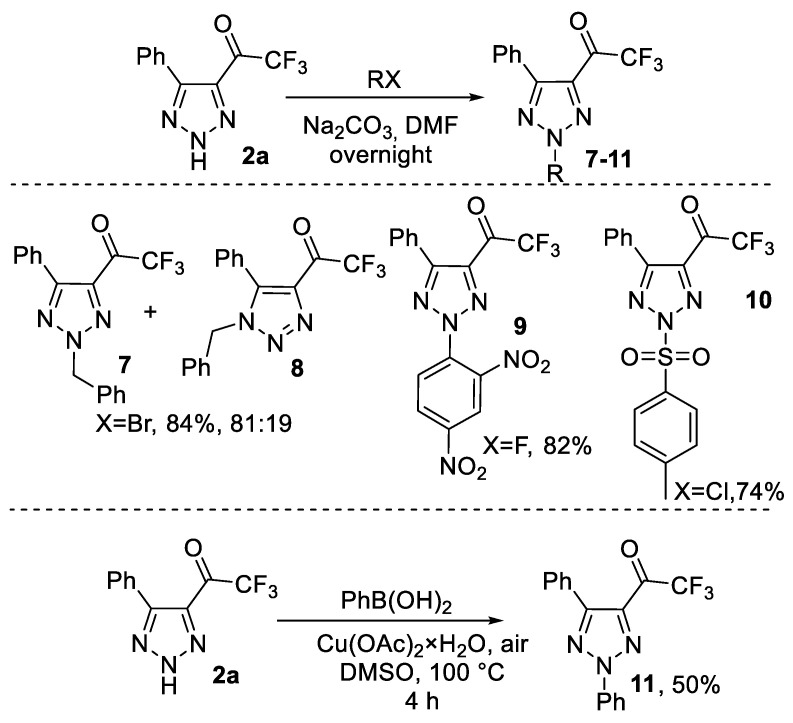
Modification of NH moiety of triazole **2a**.

Next, we studied modification of the NH moiety of triazoles **2**. We performed alkylation of model triazole **2a** in DMF using Na_2_CO_3_ as a base. It was found that the reaction with benzyl bromide led mostly to 2-benzyltriazole **7** along with 1-benzylisomer **8** in an 81:19 ratio. In contrast, the arylation and sulfonylation of **2a** proceeded 100% regioselectively to give only 2-substituted products, **9** and **10**, in high yields. Similarly, the reaction with phenyl boronic acid in non-optimized conditions of the Chan–Lam reaction 100% regioselectively produced a 50% yield of 2-phenyltriazole **11**. It should be noted that N-2-aryl-1,2,3-triazoles are of special interest due to their possible properties of UV/blue-light-emitting fluorophores [36,37,38]. Thorough investigation of the alkylation and arylation of triazoles **2,** as well as their UV/blue-light-emitting properties, is currently in progress and is going to be published soon (Scheme 6).

## 3. Materials and Methods

**General remarks.**^1^H, ^13^C and ^19^F NMR spectra were recorded using a Bruker AVANCE 400 MHz spectrometer in acetone-*d_6_*, CD_3_CN and CDCl_3_ at 400, 100 and 376 MHz, respectively. Chemical shifts (*δ*) in ppm are reported based on the residual acetone-*d_5_*, CHD_2_CN and chloroform signals (2.04, 1.94 and 7.25 for ^1^H and 29.8, 1.3, 77.0 for ^13^C) as internal references. The ^19^F chemical shifts were referenced to C_6_F_6_, (−162.9 ppm). The coupling constants (*J*) are given in Hertz (Hz). ESI-MS spectra were measured using an Orbitrap Elite instrument. For FT-IR, a spectrometer was employed using consoles of internal reflection iS3 with ATR element from ZnSe and a dip angle of 45 °C. TLC analysis was performed on “Merck 60 F_254_” plates. Column chromatography was performed usig silica gel “Macherey-Nagel 0.063–0.2 nm (Silica 60)”. In all cases, gravity column chromatography was used. All reagents were of reagent grade and were used as such or were distilled prior to use. CF_3_-ynones **1** [68] were prepared as reported previously. Melting points were determined using Electrothermal 9100 apparatus.

**Investigation of reaction of CF_3_-ynones with NaN_3_ in various solvents.** A 4 mL vial with a screw cap was charged with CF_3_-ynone **1a** (0.099 g, 0.5 mmol), corresponding solvent (2 mL, see Table 1) and NaN_3_ (0.039 g, 0.6 mmol, 1.2 equiv.). The reaction mixture was stirred overnight using a magnetic stirrer. The composition of the reaction mixture was established via ^19^F NMR using PhCF_3_ as a standard for calculation of the products’ yields. It is important to note that all manipulations with azides demand significant care for safety reasons.

**Synthesis of triazoles 2 (general procedure).** An 8 mL vial with a screw cap was charged with corresponding CF_3_-ynone **1** (1 mmol), ethanol (4 mL) and NaN_3_ (0.078 g, 1.2 mmol). The reaction mixture was stirred overnight using a magnetic stirrer. Next, ethanol was evaporated in vacuo; the residue was suspended in the heptane–ethylacetate mixture (1:1, 0.5–1 mL) and purified via column chromatography using gradient elution with mixtures of heptane–ethylacetate (9:1, 3:1 and 1:1). Evaporation of the solvents produced pure triazoles **2**.

**2,2,2-Trifluoro-1-(5-phenyl-2*H*-1,2,3-triazol-4-yl)ethanone (2a)**. Obtained from **1a** (0.202 g, 1.02 mmol). White crystals, m.p. 105–108 °C, yield 0.199 g (81%). ^1^H NMR (CDCl_3_, 400.1 MHz): δ 12.75 (br.s, 1H), 7.89–7.75 (m, 2H), 7.58–7.45 (m, 3H). ^13^C{^1^H} NMR (CDCl_3_, 100.6 MHz): δ 174.4 (q, ^2^*J*_CF_ = 37.2 Hz), 147.7, 136.1, 131.3, 129.0, 128.9, 125.1, 116.2 (q, ^1^*J*_CF_ = 290.6 Hz). ^19^F NMR (CDCl_3_, 376.5 MHz): δ −75.0 (s, 3F). ^1^H NMR (CD_3_CN, 400.1 MHz): δ 12.75 (br.s, 1H), 7.85–7.74 (m, 2H), 7.61–7.49 (m, 3H). ^13^C{^1^H} NMR (CD_3_CN, 100.6 MHz): δ 175.2 (q, ^2^*J*_CF_ = 36.3 Hz), 148.1, 137.0, 131.7, 130.1, 129.6, 127.1, 117.4 (q, ^1^*J*_CF_ = 290.2 Hz). ^19^F NMR (CD_3_CN, 376.5 MHz): δ −73.0 (s, 3F). ^1^H NMR (acetone-d6, 400.1 MHz): δ, 8.06–7.98 (m, 2H), 7.41–7.28 (m, 3H), 5.14 (br.s, 1H). ^13^C{^1^H} NMR (acetone-d6, 100.6 MHz): δ 174.7 (q, ^2^*J*_CF_ = 33.4 Hz), 152.0, 136.1, 133.5, 129.5, 128.51, 128.45, 118.6 (q, ^1^*J*_CF_ = 291.6 Hz). ^19^F NMR (acetone-d6, 376.5 MHz): δ −70.7 (s, 3F). HRMS (ESI-TOF): *m/z* [M+H]^+^ calcd for C_10_H_7_F_3_N_3_O^+^: 242.0536; found: 242.0536. IR (ν, cm^−1^): 1719 (C=O).

**2,2,2-Trifluoro-1-(5-(4-methoxyphenyl)-2*H*-1,2,3-triazol-4-yl)ethanone (2b)**. Obtained from **1b** (0.228 g, 1 mmol). Pale brown solid, m.p. 100–102 °C, yield 0.216 g (80%). ^1^H NMR (CDCl_3_, 400.1 MHz): δ 13.87 (br.s, 1H), 7.84 (d, 2H, ^3^*J* = 8.8 Hz), 7.97 (d, 2H, ^3^*J* = 8.8 Hz), 3.84 (s, 3H). ^13^C{^1^H} NMR (CDCl_3_, 100.6 MHz): δ 174.2 (q, ^2^*J*_CF_ = 37.0 Hz), 162.0, 146.7, 135.5, 130.7, 116.5, 116.3 (q, ^1^*J*_CF_ = 290.7 Hz), 114.3, 55.4. ^19^F NMR (CDCl_3_, 376.5 MHz): δ −74.7 (s, 3F). ^1^H NMR (acetone-d6, 400.1 MHz): δ 13.41 (br.s, 1H), 7.91 (d, 2H, ^3^*J* = 7.9 Hz), 7.08 (d, 2H, ^3^*J* = 8.0 Hz), 3.88 (s, 3H). ^13^C{^1^H} NMR (acetone-d6, 100.6 MHz): δ 175.0 (q, ^2^*J*_CF_ = 36.0 Hz), 162.4, 147.9, 136.2, 131.5, 127.8, 117.5 (q, ^1^*J*_CF_ = 290.6 Hz), 114.7, 55.7. ^19^F NMR (acetone-d6, 376.5 MHz): δ −72.6 (s, 3F). HRMS (ESI-TOF): *m/z* [M+H]^+^ calcd. for C_11_H_9_F_3_N_3_O_2_^+^: 272.0641; found: 272.0641. IR (ν, cm^−1^): 1719 (C=O).

**2,2,2-Trifluoro-1-(5-(4-methylthio)phenyl)-2*H*-1,2,3-triazol-4-yl)ethanone (2c)**. Obtained from **1c** (0.123 g, 0.504 mmol). Pale yellow-brown solid, m.p. 102–104 °C, yield 0.110 g (76%). ^1^H NMR (CDCl_3_, 400.1 MHz): δ 13.99 (br.s, 1H), 7.75 (d, 2H, ^3^*J* = 8.4 Hz), 7.26 (d, 2H, ^3^*J* = 7.9 Hz), 2.5 (s, 3H). ^13^C{^1^H} NMR (CDCl_3_, 100.6 MHz): δ 174.2 (q, ^2^*J*_CF_ = 37.2 Hz), 147.0, 143.7, 135.7, 129.1, 125.5, 120.7 (d, ^4^*J*_CF_ = 1.6 Hz), 116.2 (q, ^1^*J*_CF_ = 290.8 Hz), 14.7. ^19^F NMR (CDCl_3_, 376.5 MHz): δ −74.8 (s, 3F). HRMS (ESI-TOF): *m/z* [M+H]^+^ calcd. for C_11_H_9_F_3_N_3_OS^+^: 288.0413; found: 288.0417. IR (ν, cm^−1^): 1722 (C=O).

**2,2,2-Trifluoro-1-(5-(*p*-tolyl)-2*H*-1,2,3-triazol-4-yl)ethanone (2d)**. Obtained from **1d** (0.424 g, 2 mmol). Pale beige powder, m.p. 128–130 °C, yield 0.395 g (77%). ^1^H NMR (CDCl_3_, 400.1 MHz): δ 13.41 (br.s, 1H), 7.72 (d, 2H, ^3^*J* = 7.9 Hz), 7.28 (d, 2H, ^3^*J* = 8.0 Hz), 2.41 (s, 3H). ^13^C{^1^H} NMR (CDCl_3_, 100.6 MHz): δ 174.2 (q, ^2^*J*_CF_ = 37.1 Hz), 146.9 (q, ^4^*J*_CF_ = 2.3 Hz), 142.0, 135.6, 129.5, 128.9, 121.4, 116.2 (q, ^1^*J*_CF_ = 290.7 Hz), 21.4. ^19^F NMR (CDCl_3_, 376.5 MHz): δ −75.0 (s, 3F). HRMS (ESI-TOF): *m/z* [M+H]^+^ calcd. for C_11_H_9_F_3_N_3_O^+^: 256.0692; found: 256.0692. IR (ν, cm^−1^): 1719 (C=O).

**2,2,2-Trifluoro-1-(5-(4-fluorophenyl)-2*H*-1,2,3-triazol-4-yl)ethanone (2e)**. Obtained from **1e** (0.110 g, 0.509 mmol). Pale beige solid, m.p. 85–87 °C, yield 0.092 g (70%). ^1^H NMR (CDCl_3_, 400.1 MHz): δ 11.12 (br.s, 1H), 7.90–7.79 (m, 2H), 7.15 (t, 2H, ^3^*J* = 8.3 Hz). ^13^C{^1^H} NMR (CDCl_3_, 100.6 MHz): δ 174.3 (q, ^2^*J*_CF_ = 37.4 Hz), 164.3 (d, ^1^*J*_CF_ = 252.8 Hz), 147.5, 136.0, 131.3 (d, ^3^*J*_CF_ = 8.8 Hz), 121.7, 116.2 (q, ^1^*J*_CF_ = 290.5 Hz), 116.0 (d, ^2^*J*_CF_ = 22.1 Hz). ^19^F NMR (CDCl_3_, 376.5 MHz): δ −75.0 (s, 3F), 109.1 (s, 1F). HRMS (ESI-TOF): *m/z* [M+H]^+^ calcd. for C_10_H_6_F_4_N_3_O^+^: 260.0442; found: 260.0447. IR (ν, cm^−1^): 1726 (C=O).

**1-(5-(4-Chlorophenyl)-2*H*-1,2,3-triazol-4-yl)-2,2,2-trifluoroethanone (2f)**. Obtained from **1f** (0.233 g, 1 mmol). Pale beige solid, m.p. 83–85 °C, yield 0.203 g (74%). ^1^H NMR (CDCl_3_, 400.1 MHz): δ 14.05 (br.s, 1H), 7.75 (d, 2H, ^3^*J* = 8.3 Hz), 7.42 (d, 2H, ^3^*J* = 8.2 Hz). ^13^C{^1^H} NMR (CDCl_3_, 100.6 MHz): δ 174.3 (q, ^2^*J*_CF_ = 37.3 Hz), 147.1, 137.5, 136.0, 130.3, 129.1, 123.6, 116.1 (q, ^1^*J*_CF_ = 290.6 Hz). ^19^F NMR (CDCl_3_, 376.5 MHz): δ −74.9 (s, 3F). HRMS (ESI-TOF): *m/z* [M+H]^+^ calcd. for C_10_H_6_[^35^Cl]F_3_N_3_O^+^: 276.0146; found: 276.0151; calcd. for C_10_H_6_[^37^Cl]F_3_N_3_O^+^: 278.0117; found: 278.0121. IR (ν, cm^−1^): 1726 (C=O).

**1-(5-(4-Bromophenyl)-2*H*-1,2,3-triazol-4-yl)-2,2,2-trifluoroethanone (2g)**. Obtained from **1g** (0.186 g, 0.671 mmol). Pale beige solid, m.p. 82–84 °C, yield 0.166 g (77%). ^1^H NMR (CDCl_3_, 400.1 MHz): δ 13.39 (br.s, 1H), 7.69 (d, 2H, ^3^*J* = 8.5 Hz), 7.59 (d, 2H, ^3^*J* = 8.5 Hz). ^13^C{^1^H} NMR (CDCl_3_, 100.6 MHz): δ 174.3 (q, ^2^*J*_CF_ = 37.7 Hz), 147.5, 136.1, 132.1, 130.5, 125.8, 124.4, 116.1 (q, ^1^*J*_CF_ = 290.3 Hz). ^19^F NMR (CDCl_3_, 376.5 MHz): δ −75.0 (s, 3F). HRMS (ESI-TOF): *m/z* [M+H]^+^ calcd. for C_10_H_6_[^79^Br]F_3_N_3_O^+^: 319.9641; found: 319.9646; *m/z* [M+H]^+^ calcd. for C_10_H_6_[^81^Br]F_3_N_3_O^+^: 321.9620; found: 321.9625. IR (ν, cm^−1^): 1723 (C=O).

**1-(5-(4-(*Tret*-butyl)phenyl)-2*H*-1,2,3-triazol-4-yl)-2,2,2-trifluoroethanone (2h)**. Obtained from **1h** (0.130 g, 0.512 mmol). Light brown powder, m.p. 140–143 °C, yield 0.123 g (81%). ^1^H NMR (CDCl_3_, 400.1 MHz): δ 14.12 (br.s, 1H), 7.82 (d, 2Н, ^3^*J* = 8.3 Hz), 7.53 (d, 2Н, ^3^*J* = 8.2 Hz), 1.35 (s, 9H). ^13^C{^1^H} NMR (CDCl_3_, 100.6 MHz): δ 174.3 (q, ^2^*J*_CF_ = 37.1 Hz), 155.1, 147.0, 135.9, 128.8, 126.0, 121.6, 116.3 (q, ^1^*J*_CF_ = 290.7 Hz), 35.0, 31.0. ^19^F NMR (CDCl_3_, 376.5 MHz): δ −74.9 (s, 3F). HRMS (ESI-TOF): *m/z* [M+H]^+^ calcd. for C_14_H_15_F_3_N_3_O^+^: 298.1162; found: 298.1166. IR (ν, cm^−1^): 1712 (C=O).

**2,2,2-Trifluoro-1-(5-(4-trifluoromethyl)phenyl)-2*H*-1,2,3-triazol-4-yl)ethanone (2i)**. Obtained from **1i** (0.268 g, 1.01 mmol). Pale yellow solid, m.p. 67–69 °C, yield 0.230 g (74%). ^1^H NMR (CDCl_3_, 400.1 MHz): δ 10.94 (br.s, 1H), 7.94 (d, 2H, ^3^*J* = 8.2 Hz), 7.71 (d, 2H, ^3^*J* = 8.3 Hz). ^13^C{^1^H} NMR (CDCl_3_, 100.6 MHz): δ 174.5 (q, ^2^*J*_CF_ = 37.8 Hz), 148.2, 136.5, 132.6 (q, ^2^*J*_CF_ = 32.9 Hz), 129.8, 129.5, 125.7 (q, ^3^*J*_CF_ = 3.6 Hz), 123.6 (q, ^1^*J*_CF_ = 272.4 Hz), 116.1 (q, ^1^*J*_CF_ = 290.4 Hz). ^19^F NMR (CDCl_3_, 376.5 MHz): δ −64.2 (s, 3F), −75.1 (s, 3F). HRMS (ESI-TOF): *m/z* [M+H]^+^ calcd. for C_11_H_6_F_6_N_3_O^+^: 310.0410; found: 310.0416. IR (ν, cm^−1^): 1718 (C=O).

**1-(5-(3,4-Dimethylphenyl)-2*H*-1,2,3-triazol-4-yl)-2,2,2-trifluoroethanone (2j)**. Obtained from **1j** (0.118 g, 0.522 mmol). Pale brown solid, m.p. 76–78 °C, yield 0.114 g (81%). ^1^H NMR (CDCl_3_, 400.1 MHz): δ 14.05 (br.s, 1H), 7.63–7.50 (m, 2H), 7.21 (d, 1H, ^3^*J* = 7.7 Hz), 2.30 (s, 3H), 2.28 (s, 3H). ^13^C{^1^H} NMR (CDCl_3_, 100.6 MHz): δ 174.2 (q, ^2^*J*_CF_ = 37.1 Hz), 146.9, 140.7, 137.4, 135.6, 130.1, 129.8, 126.4, 121.8, 116.3 (q, ^1^*J*_CF_ = 290.7 Hz), 19.8, 19.6. ^19^F NMR (CDCl_3_, 376.5 MHz): δ −74.8 (s, 3F). HRMS (ESI-TOF): *m/z* [M+H]^+^ calcd. for C_12_H_11_F_3_N_3_O^+^: 270.0849; found: 270.0856. IR (ν, cm^−1^): 1722 (C=O).

**1-(5-(2,3-dihydrobenzo[*b***][1,4]**dioxin-6-yl)-2*H*-1,2,3-triazol-4-yl)-2,2,2-trifluoroethanone (2k)**. Obtained from **1k** (0.240 g, 1.025 mmol). Slightly yellow oil, yield 0.218 g (71%). ^1^H NMR (CDCl_3_, 400.1 MHz): δ 13.85 (br.s, 1H), 7.35 (s, 1Н), 7.31 (d, 1Н, ^3^*J* = 8.5 Hz), 6.90 (d, 1Н, ^3^*J* = 8.4 Hz), 4.31–4.23 (m, 4H). ^13^C{^1^H} NMR (CDCl_3_, 100.6 MHz): δ 174.3 (q, ^2^*J*_CF_ = 37.0 Hz), 146.9, 146.2, 143.5, 135.6, 122.5, 120.6, 118.1, 117.7, 117.8 (q, ^4^*J*_CF_ = 3.1 Hz), 116.3 (q, ^1^*J*_CF_ = 290.7 Hz), 64.5, 64.1. ^19^F NMR (CDCl_3_, 376.5 MHz): δ −74.8 (s, 3F). HRMS (ESI-TOF): *m/z* [M+H]^+^ calcd. for C_12_H_9_F_3_N_3_O_3_^+^: 300.0591; found: 300.0595. IR (ν, cm^−1^): 1717 (C=O).

**1-(5-(2-Chlorophenyl)-2*H*-1,2,3-triazol-4-yl)-2,2,2-trifluoroethanone (2l)**. Obtained from **1l** (0.237 g, 1.015 mmol). Pale brown viscous oil, yield 0.164 g (59%). ^1^H NMR (CDCl_3_, 400.1 MHz): δ 13.55 (br.s, 1H), 7.80–7.25 (m, 4H). ^13^C{^1^H} NMR (CDCl_3_, 100.6 MHz): δ 173.9 (q, ^2^*J*_CF_ = 38.1 Hz), 145.0, 137.7, 133.5, 131.8, 131.3, 129.9, 126.9, 125.3, 115.8 (q, ^1^*J*_CF_ = 290.2 Hz). ^19^F NMR (CDCl_3_, 376.5 MHz): δ −75.6 (s, 3F). HRMS (ESI-TOF): *m/z* [M+H]^+^ calcd. for C_10_H_6_[^35^Cl]F_3_N_3_O^+^: 276.0146; found: 276.0149; calcd. for C_10_H_6_[^37^Cl]F_3_N_3_O^+^: 278.0117; found: 278.0118. IR (ν, cm^−1^): 1726 (C=O).

**2,2,2-Trifluoro-1-(5-(2-methoxyphenyl)-2*H*-1,2,3-triazol-4-yl)ethanone (2m)**. Obtained from **1m** (0.195 g, 0.867 mmol). Pale brown solid, m.p. 130–132 °C, yield 0.097 g (41%). ^1^H NMR (CDCl_3_, 400.1 MHz): δ 10.67 (br.s, 1H), 7.61 (dd, 1Н, ^3^*J* = 8.5 Hz, ^4^*J* = 8.5 Hz), 7.42–7.38 (m, 1H), 7.00–6.94 (m, 2Н), 3.74 (s, 3H). ^13^C{^1^H} NMR (CDCl_3_, 100.6 MHz): δ 174.6 (q, ^2^*J*_CF_ = 36.5 Hz), 156.4, 142.0, 136.6, 132.1, 130.7, 120.3, 116.0 (q, ^1^*J*_CF_ = 290.6 Hz), 113.7, 110.8, 55.2. ^19^F NMR (CDCl_3_, 376.5 MHz): δ −74.7 (s, 3F). HRMS (ESI-TOF): *m/z* [M+H]^+^ calcd. for C_11_H_9_F_3_N_3_O_2_^+^: 272.0641; found: 272.0625.

**2,2,2-Trifluoro-1-(5-(4-methoxynaphthalen-1-yl)-2*H*-1,2,3-triazol-4-yl)ethanone (2n)**. Obtained from **1n** (0.215 g, 0.773 mmol). Slightly yellow oil, yield 0.127 g (51%). ^1^H NMR (CDCl_3_, 400.1 MHz): δ 13.00 (br.s, 1H), 8.36–8.30 (m, 1Н), 7.53–7.36 (m, 4H), 6.81 (d, 1Н, ^3^*J* = 7.9 Hz), 4.04 (s, 3H). ^13^C{^1^H} NMR (CDCl_3_, 100.6 MHz): δ 173.9 (q, ^2^*J*_CF_ = 37.6 Hz), 157.7, 145.8, 137.8, 131.8, 129.6, 127.9, 125.9, 125.5, 123.7, 122.8, 116.0 (q, ^1^*J*_CF_ = 290.4 Hz), 114.4 103.0, 55.7. ^19^F NMR (CDCl_3_, 376.5 MHz): δ −75.4 (s, 3F). HRMS (ESI-TOF): *m/z* [M+H]^+^ calcd. for C_15_H_11_F_3_N_3_O_2_^+^: 322.0798; found: 322.0805. IR (ν, cm^−1^): 1720 (C=O).

**2,2,2-Trifluoro-1-(5-octyl-2*H*-1,2,3-triazol-4-yl)ethanone (2o)**. Obtained from **1o** (0.122 g, 0.521 mmol). Pale brown solid, m.p. 66–68 °C, yield 0.082 g (56%). ^1^H NMR (CDCl_3_, 400.1 MHz): δ 13.73 (br.s, 1H), 3.21–3.07 (m, 2H), 1.75–1.65 (m, 2H), 1.37–1.19 (m, 10H), 0.87–0.81 (m, 3H). ^13^C{^1^H} NMR (CDCl_3_, 100.6 MHz): δ 174.7 (q, ^2^*J*_CF_ = 37.1 Hz), 149.3, 136.8, 116.1 (q, ^1^*J*_CF_ = 290.4 Hz), 31.7, 29.1, 29.0, 28.3, 24.1, 22.6, 14.0. ^19^F NMR (CDCl_3_, 376.5 MHz): δ −75.4 (s, 3F). HRMS (ESI-TOF): *m/z* [M+H]^+^ calcd. for C_12_H_19_F_3_N_3_O^+^: 278.1475; found: 278.1475. IR (ν, cm^−1^): 1721 (C=O).

**Synthesis of triazoles 2a,b (sub-gram scale, general procedure).** A 25 mL one-neck round-bottomed flask was charged with corresponding CF_3_-ynone **1** (5 mmol) and ethanol (5 mL) and cooled down to 0 °C in an ice bath. Next, NaN_3_ (0.390 g, 6 mmol) was added and the reaction mixture was stirred overnight using a magnetic stirrer. Next, ethanol was evaporated in vacuo. The residue was dispersed in water (4 mL) and washed twice with a heptane–ethylacetate mixture (1:1, 1 mL). The organic phase was thrown away; the water phase was acidified via dropwise addition of concentrated HCl (0.8 mL, ~9–10 mmol) to form a precipitate. The water phase was decanted and the remaining precipitate was dried in vacuo.

**2,2,2-Trifluoro-1-(5-phenyl-2*H*-1,2,3-triazol-4-yl)ethanone (2a), sub-gram-scale synthesis**. Obtained from **1a** (1.019 g, 5.146 mmol). White crystals, m.p. 105–108 °C, yield 0.994 g (80%). Calculation of E-factor: E-factor = (mass of all reagents and solvents used)/(mass of the isolated product) = (1.019 + 0.390 + 5 mL(EtOH)×0.789 + 4 mL(H_2_O)×1 + 2 mL(Heptane)×0.684+ 2 mL(EtOAc)×0.902 + 0.8 mL(HCl)×1.2))//(0.994 g) = 13.509/0.994 = 13.6.

**2,2,2-Trifluoro-1-(5-(4-methoxyphenyl)-2*H*-1,2,3-triazol-4-yl)ethanone (2b)**, sub-gram-scale synthesis. Obtained from 1b (0.674 g, 1 mmol). Pale brown solid, m.p. 100–102 °C, yield 0.681 g (85%).

**Investigation of reaction of CF_3_-ynones with NaN_3_ in various solvents in the presence of acids.** A 4 mL vial with a screw cap was charged with CF_3_-ynone **1a** (0.099 g, 0.5 mmol), the corresponding solvent (2 mL, see Table S2), the corresponding acid (for quantity of acid, see Table S2) and NaN_3_ (for quantity, see Table S2). The reaction mixture was stirred for 1 day using a magnetic stirrer. The composition of the reaction mixture was established via ^19^F NMR using PhCF_3_ as a standard for calculation of the products’ yields.

**Synthesis of isoxazoles 3 in ethanol (general procedure A).** An 8 mL vial with a screw cap was charged with the corresponding CF_3_-ynone **1** (0.5 mmol), ethanol (2 mL), acetic acid (0.120 g, 2 mmol, 4 equiv.) and NaN_3_ (0.065 g, 1 mmol, 2 equiv.). The reaction mixture was stirred overnight using a magnetic stirrer. Next, ethanol was evaporated in vacuo at room temperature (it is important to note that isoxazoles **4a** and **4x** are quite volatile); the residue was suspended in the heptane–ethylacetate mixture (3:1, 0.5) and passed through a short silica gel pad via gradient elution using the heptane and heptane–ethylacetate mixture (9:1). Evaporation of the solvents produced pure isoxazoles **3**.

**Synthesis of isoxazoles 3 in heptane (general procedure B).** An 8 mL vial with a screw cap was charged with corresponding CF_3_-ynone **1** (0.5 mmol), heptane (4 mL), water (0.5 mL), acetic acid (0.120 g, 2 mmol, 4 equiv.) and NaN_3_ (0.065 g, 1 mmol, 2 equiv.). The reaction mixture was stirred overnight using a magnetic stirrer. Next, the heptane phase was separated, the water phase was extracted with a heptane–ethylacetate mixture (9:1, 2 × 0.5 mL) and the combined extract was passed through a short silica gel pad using gradient elution with heptane followed by the heptane–ethylacetate mixtures (9:1). Evaporation of the solvents produced pure isoxazoles **3**.

**3-Phenyl-5-(trifluoromethyl)isoxazole (3a)**. Obtained from **1a** (0.099 g, 0.5 mmol (A); 0.100 g, 0.505 mmol (B)). White crystals, m.p. 74–77 °C (Lit.: 73.3–74 °C [69], 76–78 °C [70]), yield 0.037 g (35%, (A)); 0.039 g (36%, (B)). ^1^H NMR (CDCl_3_, 400.1 MHz): δ 7.85–7.76 (m, 2Н), 7.54–7.44 (m, 3Н), 7.0 (s, 1H). ^13^C{^1^H} NMR (CDCl_3_, 100.6 MHz): δ 162.5, 159.1 (q, ^2^*J*_CF_ = 42.6 Hz), 130.9, 129.2, 127.3, 126.9, 117.9 (q, ^1^*J*_CF_ = 270.3 Hz), 103.4 (q, ^4^*J*_CF_ = 2.0 Hz). ^19^F NMR (CDCl_3_, 376.5 MHz): δ −65.3 (s, 3F). NMR data are in agreement with those in the literature [70].

**3-(4-Methoxyphenyl)-5-(trifluoromethyl)isoxazole (3b)**. Obtained from **1b** (0.114 g, 0.5 mmol (A); 0.110 g, 0.482 mmol (B)). Pale yellow solid, m.p. 72–74 °C (Lit.: 72–75 °C [70]), yield 0.021 g (17%, (A)); 0.019 g (16%, (B)). ^1^H NMR (CDCl_3_, 400.1 MHz): δ 7.74 (d, 2Н, ^3^*J* = 8.9 Hz), 6.99 (d, 2Н, ^3^*J* = 8.9 Hz), 6.94 (*pseudo*-d, 1H, ^4^*J* = 0.8 Hz), 3.86 (s, 3H). ^13^C{^1^H} NMR (CDCl_3_, 100.6 MHz): δ 162.1, 161.7, 158.9 (q, ^2^*J*_CF_ = 42.4 Hz), 128.4, 119.7, 117.9 (q, ^1^*J*_CF_ = 270.0 Hz), 114.6, 103.2 (q, ^4^*J*_CF_ = 1.8 Hz), 55.4. ^19^F NMR (CDCl_3_, 376.5 MHz): δ −65.4 (d, 3F, *J* = 0.9 Hz). NMR data are in agreement with those in the literature [70].

**3-(4-Methylphenyl)-5-(trifluoromethyl)isoxazole (3d)**. Obtained from **1d** (0.106 g, 0.5 mmol (A); 0.115 g, 0.542 mmol (B)). Pale yellow solid, m.p. 78–80 °C (Lit.: 79–80 °C [71]), yield 0.035 g (31%, (A)); 0.036 g (29%, (B)). ^1^H NMR (CDCl_3_, 400.1 MHz): δ 7.70 (d, 2Н, ^3^*J* = 8.0 Hz), 7.29 (d, 2Н, ^3^*J* = 8.0 Hz), 6.97 (*pseudo*-d, 1H, ^4^*J* = 0.9 Hz), 2.41 (s, 3H). ^13^C{^1^H} NMR (CDCl_3_, 100.6 MHz): δ 162.5, 159.0 (q, ^2^*J*_CF_ = 42.4 Hz), 141.2, 129.9, 126.8, 124.4, 117.9 (q, ^1^*J*_CF_ = 270.4 Hz), 103.3 (q, ^4^*J*_CF_ = 2.0 Hz), 21.4. ^19^F NMR (CDCl_3_, 376.5 MHz): δ −65.4 (d, 3F, *J* = 0.9 Hz). NMR data are in agreement with those in the literature [71].

**3-(4-Fluorophenyl)-5-(trifluoromethyl)isoxazole (3e)**. Obtained from **1e** (0.113 g, 0.5 mmol (A); 0.113 g, 0.5 mmol (B)). Pale beige crystals, m.p. 49–50 °C (Lit.: 48 °C [72]), yield 0.036 g (31%, (A)); 0.035 g (30%, (B)). ^1^H NMR (CDCl_3_, 400.1 MHz): δ 7.83–7.78 (m, 2Н), 7.21–7.15 (m, 2Н), 6.97 (*pseudo*-d, 1H, ^4^*J* = 0.9 Hz). ^13^C{^1^H} NMR (CDCl_3_, 100.6 MHz): δ 164.3 (d, ^1^*J*_CF_ = 251.7 Hz), 161.6, 159.4 (q, ^2^*J*_CF_ = 42.7 Hz), 129.0 (d, ^3^*J*_CF_ = 8.7 Hz), 123.5 (d, ^4^*J*_CF_ = 3.5 Hz), 117.8 (q, ^1^*J*_CF_ = 270.5 Hz), 116.4 (d, ^2^*J*_CF_ = 22.1 Hz), 103.3 (q, ^4^*J*_CF_ = 2.2 Hz). ^19^F NMR (CDCl_3_, 376.5 MHz): δ −65.4 (d, 3F, *J* = 0.8 Hz), -109.9--110.0 (m, 1F). NMR data are in agreement with those in the literature [70].

**3-(4-Chlorophenyl)-5-(trifluoromethyl)isoxazole (3f)**. Obtained from **1f** (0.117 g, 0.503 mmol (A); 0.114 g, 0.49 mmol (B)). Pale yellow solid, m.p. 51–53 °C (Lit.: 61–62 °C [71]), yield 0.047 g (38%, (A)); 0.042 g (35%, (B)). ^1^H NMR (CDCl_3_, 400.1 MHz): δ 7.74 (d, 2Н, ^3^*J* = 8.7 Hz), 7.46 (d, 2Н, ^3^*J* = 8.7 Hz), 6.98 (*pseudo*-d, 1H, ^4^*J* = 0.9 Hz). ^13^C{^1^H} NMR (CDCl_3_, 100.6 MHz): δ 161.5, 159.5 (q, ^2^*J*_CF_ = 42.6 Hz), 137.1, 129.5, 128.2, 125.7, 117.7 (q, ^1^*J*_CF_ = 270.3 Hz), 103.3 (q, ^4^*J*_CF_ = 2.0 Hz). ^19^F NMR (CDCl_3_, 376.5 MHz): δ −65.4 (d, 3F, *J* = 0.9 Hz). NMR data are in agreement with those in the literature [71].

**3-(4-Bromophenyl)-5-(trifluoromethyl)isoxazole (3g)**. Obtained from **1g** (0.140 g, 0.505 mmol (A); 0.143 g, 0.516 mmol (B)). Colorless solid, m.p. 71–73 °C (Lit.: 63–64 °C [71]), yield 0.053 g (36%, (A)); 0.051 g (34%, (B)). ^1^H NMR (CDCl_3_, 400.1 MHz): δ 7.68 (d, 2Н, ^3^*J* = 8.6 Hz), 7.62 (d, 2Н, ^3^*J* = 8.6 Hz), 6.98 (*pseudo*-d, 1H, ^4^*J* = 0.8 Hz). ^13^C{^1^H} NMR (CDCl_3_, 100.6 MHz): δ 161.7, 159.5 (q, ^2^*J*_CF_ = 42.8 Hz), 132.5, 128.4, 126.2, 125.4, 117.7 (q, ^1^*J*_CF_ = 270.4 Hz), 103.3 (q, ^4^*J*_CF_ = 2.0 Hz). ^19^F NMR (CDCl_3_, 376.5 MHz): δ −65.4 (d, 3F, *J* = 0.6 Hz). NMR data are in agreement with those in the literature [72].

**3-(4-(*tert*-Butyl)phenyl)-5-(trifluoromethyl)isoxazole (3h)**. Obtained from **1h** (0.121 g, 0.476 mmol (A); 0.122 g, 0.48 mmol (B)). Pale yellow viscous oil, yield 0.041 g (32%, (A)); 0.033 g (26%, (B)). ^1^H NMR (CDCl_3_, 400.1 MHz): δ 7.74 (d, 2Н, ^3^*J* = 8.7 Hz), 7.51 (d, 2Н, ^3^*J* = 8.7 Hz), 6.97 (*pseudo*-d, 1H, ^4^*J* = 0.9 Hz), 2.41 (s, 3H). ^13^C{^1^H} NMR (CDCl_3_, 100.6 MHz): δ 162.4, 159.0 (q, ^2^*J*_CF_ = 42.4 Hz), 154.4, 126.7, 126.2, 124.4, 117.9 (q, ^1^*J*_CF_ = 270.4 Hz), 103.4 (q, ^4^*J*_CF_ = 2.0 Hz), 34.9, 31.1. ^19^F NMR (CDCl_3_, 376.5 MHz): δ −65.4 (d, 3F, *J* = 0.6 Hz). HRMS (ESI-TOF): *m/z* [M+H]^+^ calcd. for C_14_H_15_F_3_NO^+^: 270.1100; found: 270.1102.

**5-(Trifluoromethyl)-3-(4-(trifluoromethyl)phenyl)isoxazole (3i)**. Obtained from **1i** (0.134 g, 0.504 mmol (A); 0.137 g, 0.515 mmol (B)). Colorless solid, m.p. 55–57 °C (Lit.: 63–64 °C [73]), yield 0.067 g (47%, (A)); 0.070 g (48%, (B)). ^1^H NMR (CDCl_3_, 400.1 MHz): δ 7.94 (d, 2Н, ^3^*J* = 8.2 Hz), 7.75 (d, 2Н, ^3^*J* = 8.2 Hz), 7.05 (*pseudo*-d, 1H, ^4^*J* = 0.8 Hz). ^13^C{^1^H} NMR (CDCl_3_, 100.6 MHz): δ 161.5, 159.8 (q, ^2^*J*_CF_ = 42.9 Hz), 132.8 (q, ^2^*J*_CF_ = 32.8 Hz), 130.7, 127.3, 126.2 (q, ^3^*J*_CF_ = 3.9 Hz), 123.6 (q, ^1^*J*_CF_ = 272.4 Hz), 117.7 (q, ^1^*J*_CF_ = 270.5 Hz), 103.5 (q, ^4^*J*_CF_ = 2.0 Hz). ^19^F NMR (CDCl_3_, 376.5 MHz): δ −64.2 (d, 3F), −65.3 (d, 3F, *J* = 0.6 Hz). NMR data are in agreement with those in the literature [73].

**3-(2,3-diHydrobenzo[*b***][1,4]**dioxin-6-yl)-5-(trifluoromethyl)isoxazole (3k)**. Obtained from **1k** (0.128 g, 0.5 mmol (A); 0.126 g, 0.492 mmol (B)). Colorless solid, m.p. 102–104 °C, yield 0.026 g (19%, (A)); 0.021 g (16%, (B)). ^1^H NMR (CDCl_3_, 400.1 MHz): δ 7.34 (d, 1Н, ^4^*J* = 2.1 Hz), 7.30 (dd, 1Н, ^3^*J* = 8.4 Hz, ^4^*J* = 2.1 Hz), 6.96 (d, 1Н, ^3^*J* = 8.4 Hz), 6.91 (*pseudo*-d, 1H, ^4^*J* = 0.9 Hz), 4.34–4.29 m, 4H). ^13^C{^1^H} NMR (CDCl_3_, 100.6 MHz): δ 162.0, 158.9 (q, ^2^*J*_CF_ = 42.4 Hz), 145.9, 144.0, 120.5, 120.3, 118.0, 117.9 (q, ^1^*J*_CF_ = 270.8 Hz), 116.0, 103.3 (q, ^4^*J*_CF_ = 2.0 Hz), 64.5, 64.3. ^19^F NMR (CDCl_3_, 376.5 MHz): δ −65.4 (d, 3F, ^4^*J* = 0.4 Hz). HRMS (ESI-TOF): *m/z* [M+H]^+^ calcd. for C_12_H_9_F_3_NO_3_^+^: 272.0529; found: 272.0531.

**3-(2-Chlorophenyl)-5-(trifluoromethyl)isoxazole (3l)**. Obtained from **1l** (0.119 g, 0.512 mmol (A); 0.123 g, 0.529 mmol (B)). Pale yellow oil, yield 0.037 g (29%, (A)); 0.047 g (36%, (B)). ^1^H NMR (CDCl_3_, 400.1 MHz): δ 7.75 (dd, 1Н, ^3^*J* = 7.7 Hz, ^4^*J* = 1.8 Hz), 7.52 (dd, 1Н, ^3^*J* = 7.7 Hz, ^4^*J* = 1.4 Hz), 7.45 (td, 1H, ^3^*J* = 7.7 Hz, ^4^*J* = 1.8 Hz), 7.39 (td, 1H, ^3^*J* = 7.7 Hz, ^4^*J* = 1.4 Hz), 7.19 (*pseudo*-d, 1H, ^4^*J* = 0.8 Hz). ^13^C{^1^H} NMR (CDCl_3_, 100.6 MHz): δ 161.2, 158.5 (q, ^2^*J*_CF_ = 42.6 Hz), 132.9, 131.7, 131.1, 130.6, 127.4, 126.5, 117.8 (q, ^1^*J*_CF_ = 270.3 Hz), 106.6 (q, ^4^*J*_CF_ = 2.0 Hz). ^19^F NMR (CDCl_3_, 376.5 MHz): δ −65.2 (d, 3F, ^4^*J* = 0.9 Hz). HRMS (ESI-TOF): *m/z* [M+H_3_O]^+^ calcd. for C_10_H_8_[^35^Cl]F_3_NO_2_^+^: 266.0190; found: 266.0190; calcd. for C_10_H_8_[^37^Cl]F_3_NO_2_^+^: 268.0161; found: 268.0156.

**3-(2-Methoxyphenyl)-5-(trifluoromethyl)isoxazole (3m)**. Obtained from **1m** (0.113 g, 0.502 mmol (A); 0.108 g, 0.48 mmol (B)). Colorless oil, yield 0.026 g (21%, (A)); 0.030 g (26%, (B)). ^1^H NMR (CDCl_3_, 400.1 MHz): δ 7.91 (dd, 1Н, ^3^*J* = 7.7 Hz, ^4^*J* = 1.7 Hz), 7.48–7.44 (m, 1Н), 7.22 (s, 1H), 7.06 (*pseudo*-t, 1H, ^3^*J* = 7.5 Hz), 7.02 (d, 1H, ^3^*J* = 8.4 Hz), 3.92 (s, 1H). ^13^C{^1^H} NMR (CDCl_3_, 100.6 MHz): δ 160.1, 157.9 (q, ^2^*J*_CF_ = 42.2 Hz), 157.2, 132.1, 129.4, 121.1, 118.1 (q, ^1^*J*_CF_ = 270.0 Hz), 116.1, 111.4, 106.9 (q, ^4^*J*_CF_ = 2.0 Hz), 55.6. ^19^F NMR (CDCl_3_, 376.5 MHz): δ −65.3 (d, 3F, ^4^*J* = 0.9 Hz). HRMS (ESI-TOF): *m/z* [M+H]^+^ calcd. for C_11_H_9_F_3_NO_2_^+^: 244.0580; found: 244.0581.

**3-Octyl-5-(trifluoromethyl)isoxazole (3o)**. Obtained from **1o** (0.118 g, 0.504 mmol (A); 0.130 g, 0.556 mmol (B)). Colorless oil, yield 0.041 g (32%, (A)); 0.046 g (33%, (B)). ^1^H NMR (CDCl_3_, 400.1 MHz): δ 6.53 (*pseudo*-d, 1H, ^4^*J* = 0.7 Hz), 2.71 (t, 2Н, ^3^*J* = 7.7 Hz), 1.70–1.63 (m, 2H), 1.39–1.19 (m, 10H), 0.88–0.85 (m, 3H). ^13^C{^1^H} NMR (CDCl_3_, 100.6 MHz): δ 164.2, 158.4 (q, ^2^*J*_CF_ = 42.4 Hz), 118.0 (q, ^1^*J*_CF_ = 270.2 Hz), 104.9 (q, ^4^*J*_CF_ = 1.9 Hz), 31.8, 29.14, 29.08, 29.04, 28.0, 25.8, 22.6, 14.1. ^19^F NMR (CDCl_3_, 376.5 MHz): δ −65.4 (d, 3F, ^4^*J* = 0.9 Hz). HRMS (ESI-TOF): *m/z* [M+H]^+^ calcd. for C_12_H_19_F_3_NO^+^: 250.14133; found: 250.141.

**3-(Phenoxymethyl)-5-(trifluoromethyl)isoxazole (3p)**. Obtained from **1p** (0.117 g, 0.52 mmol (A); 0.125 g, 0.556 mmol (B)). Pale yellow oil, yield 0.065 g (51%, (A)); 0.064 g (47%, (B)). ^1^H NMR (CDCl_3_, 400.1 MHz): δ 7.36–7.30 (m, 2Н), 7.05–7.01 (m, 1Н), 6.99–6.96 (m, 2Н), 6.86 (*pseudo*-d, 1H, ^4^*J* = 0.4 Hz), 5.20 (s, 2H). ^13^C{^1^H} NMR (CDCl_3_, 100.6 MHz): δ 161.2, 159.2 (q, ^2^*J*_CF_ = 42.8 Hz), 157.6, 129.7, 122.0, 117.7 (q, ^1^*J*_CF_ = 270.5 Hz), 114.6, 104.9 (q, ^4^*J*_CF_ = 2.0 Hz), 61.0. ^19^F NMR (CDCl_3_, 376.5 MHz): δ −65.2 (d, 3F, ^4^*J* = 0.6 Hz). HRMS (ESI-TOF): *m/z* [M+H]^+^ calcd. for C_11_H_9_F_3_NO_2_^+^: 244.0580; found: 244.0585.

**Synthesis of amides 4–6 (general procedure).** A 4 mL vial with a screw cap was charged with 2,2,2-trifluoro-1-(5-phenyl-2*H*-1,2,3-triazol-4-yl)ethanone (**2a**). (0.054–0.059 g, 0.224–0.245 mmol) and the corresponding amine (0.189–0.230 g, ~2.7 mmol, ~12 equiv.) The reaction mixture was heated at 90 °C for 2.5–8 h using a magnetic stirrer while heating, and then, the volatiles were evaporated in vacuo. The residue was passed through a short silica gel pad using CH_2_Cl_2_ followed by CH_2_Cl_2_ -MeOH (100:1) as eluents. The evaporation of volatiles produced corresponding pure amide **4–6**.

**(5-Phenyl-2*H*-1,2,3-triazol-4-yl)(pyrrolidin-1-yl)methanone (4)**. Obtained from **2a** (0.054 g, 0.224 mmol) and pyrrolidine (0.189 g, 2.66 mmol) by heating for 2.5 h. Pale brown solid, m.p. 162–164 °C, yield 0.046 g (85%). ^1^H NMR (CDCl_3_, 400.1 MHz): δ 7.76–7.65 (m, 2H), 7.35–7.25 (m, 3H), 3.67 (t, 2H, ^3^*J* = 6.9 Hz), 3.38 (t, 2H, ^3^*J* = 6.9 Hz), 1.92–1.85 (m, 2H), 1.85–1.74 (m, 2H). ^13^C{^1^H} NMR (CDCl_3_, 100.6 MHz): δ 162.5, 142.8, 138.0, 128.9, 128.6, 127.6, 48.6, 46.4, 25.9, 24.2. HRMS (ESI-TOF): *m/z* [M+Na]^+^ calcd. for C_13_H_14_N_4_ONa^+^: 265.1060; found: 265.1068. IR (ν, cm^−1^): 1595 (C=O).

**(5-Phenyl-2*H*-1,2,3-triazol-4-yl)(piperidin-1-yl)methanone (5)**. Obtained from **2a** (0.053 g, 0.220 mmol) and piperidine (0.228 g, 2.68 mmol) by heating for 8 h. Pale brown solid, m.p. 123–125 °C, yield 0.033 g (58%). ^1^H NMR (CDCl_3_, 400.1 MHz): δ 7.66 (dd, 2H, ^3^*J* = 6.9 Hz, ^4^*J* = 1.9 Hz), 7.41–7.38 (m, 3H), 3.82–3.62 (m, 2H), 3.28–3.10 (m, 2H), 1.68–1.48 (m, 4H), 1.34–1.21 (m, 2H). ^13^C{^1^H} NMR (CDCl_3_, 100.6 MHz): δ 163.0, 142.9, 137.4, 128.9, 128.8, 128.5, 127.3, 48.3, 43.3, 26.0, 25.4, 24.3. HRMS (ESI-TOF): *m/z* [M+H]^+^ calcd. for C_14_H_17_N_4_O^+^: 257.1397; found: 257.1403. IR (ν, cm^−1^): 1592 (C=O).

**Morpholino(5-phenyl-2*H*-1,2,3-triazol-4-yl)methanone (6)**. Obtained from **2a** (0.053 g, 0.220 mmol) and morpholine (0.230 g, 2.64 mmol) by heating for 8 h. Pale brown viscous sticky mass, yield 0.027 g (48%). ^1^H NMR (CDCl_3_, 400.1 MHz): δ 7.73–7.59 (m, 2H), 7.44–7.34 (m, 3H), 3.87–3.79 (m, 2H), 3.77–3.70 (m, 2H), 3.47–3.39 (m, 2H), 3.39–3.27 (m, 2H). ^13^C{^1^H} NMR (CDCl_3_, 100.6 MHz): δ 163.0, 141.6, 136.9, 129.3, 128.9, 127.5, 127.3, 66.53, 66.48, 47.5, 42.7. HRMS (ESI-TOF): *m/z* [M+H]^+^ calcd. for C_13_H_15_N_4_O_2_^+^: 259.1190; found: 259.1190. IR (ν, cm^−1^): 1604 (C=O).

**Synthesis of triazoles 7–10 (general procedure).** A 4 mL vial with a screw cap was charged with 2,2,2-trifluoro-1-(5-phenyl-2*H*-1,2,3-triazol-4-yl)ethanone (**2a**) (0.120 g, 0.498 mmol), DMF (1 mL), Na_2_CO_3_ (0.079 g, 0.745 mmol, 1.5 equiv.), and corresponding alkylating reagent (0.548 mmol, ~1.1 equiv.) The reaction mixture was stirred overnight, poured into water (20 mL) and extracted with CH_2_Cl_2_ (3 × 20 mL). The combined organic phase was washed with water (20 mL) and dried over Na_2_SO_4_, and then, the volatiles were evaporated in vacuo. The residue was purified via column chromatography on silica gel using appropriate mixtures of hexane and CH_2_Cl_2_ as eluents.

**1-(2-Benzyl-5-phenyl-2*H*-1,2,3-triazol-4-yl)-2,2,2-trifluoroethanone (7)**. Obtained from **2a** (0.120 g, 0.498 mmol) and benzylbromide (0.094 g, 0.550 mmol). Purified using gradient elution with hexane-CH_2_Cl_2_ (3:1) followed by hexane-CH_2_Cl_2_ (1:1) and CH_2_Cl_2_. Beige oil, yield 0.113 g (69%). ^1^H NMR (CDCl_3_, 400.1 MHz): δ 7.96–7.86 (m, 2H), 7.53–7.44 (m, 5H), 7.44–7.36(m, 3H), 5.71 (s, 2H). ^13^C{^1^H} NMR (CDCl_3_, 100.6 MHz): δ 174.2 (q, ^2^*J*_CF_ = 37.0 Hz), 152.7, 136.4, 133.5, 130.1, 129.1, 129.0, 128.9, 128.40, 128.35, 128.2, 116.3 (q, ^1^*J*_CF_ = 290.7 Hz), 59.9. ^19^F NMR (CDCl_3_, 376.5 MHz): δ −74.9 (s, 3F). HRMS (ESI-TOF): *m/z* [M+H]^+^ calcd. for C_17_H_13_F_3_N_3_O^+^: 332.1005; found: 332.1005. IR (ν, cm^−1^): 1723 (C=O).

**1-(1-Benzyl-5-phenyl-1*H*-1,2,3-triazol-4-yl)-2,2,2-trifluoroethanone (8)**. Obtained from **2a** as an admixture (81:19) in the synthesis of 7. Beige thick oil, yield 0.026 g (15%). ^1^H NMR (CDCl_3_, 400.1 MHz): δ 7.58–7.52 (m, 1H), 7.51–7.46 (m, 2H), 7.31–7.25 (m, 3H), 7.24–7.20 (m, 2H), 7.08–7.00 (m, 2H), 5.46 (s, 2Н). ^13^C{^1^H} NMR (CDCl_3_, 100.6 MHz): δ 174.2 (q, ^2^*J*_CF_ = 37.3 Hz), 143.9, 138.1, 133.9, 130.9, 129.4, 129.0, 128.9, 128.7, 127.7, 124.4, 116.1 (q, ^1^*J*_CF_ = 290.6 Hz), 52.2. ^19^F NMR (CDCl_3_, 376.5 MHz): δ −75.4 (s, 3F). NMR data are in agreement with those in the literature [54].

**1-(2-(2,4-Dinitrophenyl)-5-phenyl-2*H*-1,2,3-triazol-4-yl)-2,2,2-trifluoroethanone (9)**. Obtained from **2a** (0.057 g, 0.237 mmol) and 1-fluoro-2,4-dinitrobenzene (0.049 g, 0.263 mmol). Purified using gradient elution with hexane-CH_2_Cl_2_ (3:1) followed by hexane-CH_2_Cl_2_ (1:1). Pale yellow powder, m.p. 132–134 °C, yield 0.079 g (82%). ^1^H NMR (CDCl_3_, 400.1 MHz): δ 8.67 (d, 1Н, ^4^*J* = 2.4 Hz), 8.56 (dd, 1Н, ^3^*J* = 8.9 Hz, ^4^*J* = 2.4 Hz), 8.31 (d, 1Н, ^3^*J* = 8.9 Hz), 7.83 (dd, 2H, ^3^*J* = 7.7 Hz, ^4^*J* = 1.7 Hz), 7.49–7.40 (m, 3H). ^13^C{^1^H} NMR (CDCl_3_, 100.6 MHz): δ 173.7 (q, ^2^*J*_CF_ = 38.2 Hz), 153.8, 147.1, 142.8, 138.7, 134.1, 130.8, 129.0, 128.4, 127.4, 126.4, 120.7, 115.6 (q, ^1^*J*_CF_ = 290.5 Hz). ^19^F NMR (CDCl_3_, 376.5 MHz): δ −75.0 (s, 3F). HRMS (ESI-TOF): *m/z* [M+H]^+^ calcd. for C_16_H_9_F_3_N_5_O_5_^+^: 408.0550; found: 408.0549. IR (ν, cm^−1^): 1738 (C=O); 1545, 1540, 1349, 1335 (NO_2_).

**2,2,2-Trifluoro-1-(5-phenyl-2-tosyl-2*H*-1,2,3-triazol-4-yl)ethanone (10)**. Obtained from **2a** (0.060 g, 0.249 mmol) and 4-toluenesulfonyl chloride (0.052 g, 0.274 mmol). Purified using gradient elution with hexane-CH_2_Cl_2_ (1:1) followed by CH_2_Cl_2_.White crystals, m.p. 156–160 °C, yield 0.073 g (74%). ^1^H NMR (CDCl_3_, 400.1 MHz): δ 8.08 (d, 2Н, ^3^*J* = 8.4 Hz), 7.83 (dd, 2Н, ^3^*J* = 7.9 Hz, ^4^*J* = 1.4 Hz) 7.52–7.39 (m, 5H), 2.46 (s, 3H). ^13^C{^1^H} NMR (CDCl_3_, 100.6 MHz): δ 174.5 (q, ^2^*J*_CF_ = 37.9 Hz), 153.4, 148.1, 138.6, 131.6, 130.8, 130.6, 129.6, 129.3, 128.5, 126.9, 115.8 (q, ^1^*J*_CF_ = 290.5 Hz), 21.9. ^19^F NMR (CDCl_3_, 376.5 MHz): δ −75.2 (s, 3F). HRMS (ESI-TOF): *m/z* [M+H]^+^ calcd. for C_17_H_13_F_3_N_3_O_3_S^+^: 396.0324; found: 396.0626. IR (ν, cm^−1^): 1731 (C=O).

**Synthesis of 1-(2,5-diphenyl-2*H*-1,2,3-triazol-4-yl)-2,2,2-trifluoroethanone (11)**. A 25 mL round-bottomed flask was charged with 2,2,2-trifluoro-1-(5-phenyl-2*H*-1,2,3-triazol-4-yl)ethanone (**2a**) (0.062 g, 0.257 mmol), DMSO (1.5 mL), PhB(OH)_2_ (0.047 g, 0.385 mmol, 1.5 equiv.) and Cu(OAc)_2_×H_2_O (0.0051, 0.257 mmol, 0.1 equiv.) The reaction mixture was heated at 100 °C for 5 h in air using a magnetic stirrer while heating. The reaction mixture was poured into water (20 mL) and extracted with CH_2_Cl_2_ (3 × 20 mL). Combined organic phase was washed with water (20 mL) and dried over Na_2_SO_4_, and then, the volatiles were evaporated in vacuo. The residue was purified via column chromatography on silica gel using gradient elution with hexane-CH_2_Cl_2_ (3:1) followed by hexane-CH_2_Cl_2_ (1:1) as eluents. Colorless crystals, m.p. 93–95 °C, yield 0.032 g (50%). ^1^H NMR (CDCl_3_, 400.1 MHz): δ 8.28–8.18 (m, 2H), 8.03–7.95 (m, 2H), 7.59–7.46 (m, 6H). ^13^C{^1^H} NMR (CDCl_3_, 100.6 MHz): δ 174.4 (q, ^2^*J*_CF_ = 37.5 Hz), 152.9, 138.8, 137.1, 130.4, 129.6, 129.4, 129.2, 128.5, 128.1, 119.7, 116.3 (q, ^1^*J*_CF_ = 290.9 Hz). ^19^F NMR (CDCl_3_, 376.5 MHz): δ −74.9 (s, 3F). HRMS (ESI-TOF): *m/z* [M+H]^+^ calcd. for C_16_H_11_F_3_N_3_O^+^: 318.0849; found: 318.0851. IR (ν, cm^−1^): 1716 (C=O).

## 4. Conclusions

In conclusion, we determined a green approach towards 4-trifluoroacetyltriazoles or 5-CF_3_-isoxazoles based on the reaction of CF_3_-ynones with NaN_3_ in EtOH. The reaction pathway could be totally controlled by the addition of acid to the reaction mixture. Without the addition of any acids, the reaction gave 4-trifluoroacetyltriazoles in isolated yields of up to 85%. In the presence of acid, the reaction of CF_3_-ynones with NaN_3_ in EtOH or heptane led to 5-CF_3_-isoxazoles in good yields. The utility of the elaborated method was confirmed by gram-scale synthesis. A remarkably low E-factor was demonstrated. Several reactions of prepared 4-trifluoroacetyltriazoles were investigated, and the high synthetic utility of these products was demonstrated.

## Figures and Tables

**Table 1:**
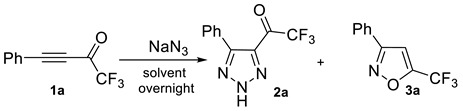
Investigation of the solvents’ nature in the reaction of sodium azide with CF_3_-ynone **1**.

Entry	Solvent	Yield of 2a, % ^a^	Yield of 3a, %
1	PhMe	2	12
2	Acetone-H_2_O	12	5
3	MeCN	26	7
4	THF	19	12
5	EtOAc-H_2_O	23	14
6	DMF	49	2
7	DMSO	48	1
8	NMP	52	2
9	MeOH	77	4
10	EtOH	85(81 ^b^)	3

^a^—by ^19^F NMR; ^b^—isolated yield.

## Data Availability

Not applicable.

## Supplementary Materials

The following supporting information can be downloaded at: https://www.mdpi.com/article/10.3390/ijms232314522/s1. Copies of all ^1^H, ^13^C and ^19^F NMR spectra, and computational details.
