# Supplementary material for: Acid-Switchable Synthesis of Trifluoromethylated Triazoles and Isoxazoles via Reaction of CF3-Ynones with NaN3: DFT Study of the Reaction Mechanism
Source: Int J Mol Sci. 2022 Nov 22;23(23):14522. doi: 10.3390/ijms232314522 (PMC9735682; doi:10.3390/ijms232314522)
Supplement: Supplementary file 1 [file ijms-23-14522-s001.zip › ijms-2014652-supplementary.pdf]

**A green, acid switchable approach to trifluoromethylated triazoles and isoxazoles via reaction of $CF_3$-ynones with sodium azide**

Vasiliy M. Muzalevskiy,[a] Zoia A. Sizova,[a] Mikhail S. Nechaev,[a,b] and Valentine G. Nenajdenko[a,*]

[a] *M. V. Lomonosov Moscow State University, Department of Chemistry, Leninskie Gory 1, Moscow, 119991 Russia*

[b] *A. V. Topchiev Institute of Petrochemical Synthesis, Russian Academy of Sciences, 119991 Moscow, Russian Federation*

**Table of contents**

**Experimental section. General remarks** **S2**

**Investigation of reaction of $CF_3$-ynones with $NaN_3$ in various solvents** **S2**

**Synthesis of triazoles 2 (general procedure)** **S3**

**Characterization data of triazoles 2** **S3-S7**

**Synthesis of triazoles 2a,b (sub-gram scale, general procedure)** **S7**

**Investigation of reaction of $CF_3$-ynones with $NaN_3$ in various solvents in the presence of acids** **S8**

**Synthesis of isoxazoles 3 in ethanol (general procedure A)** **S9**

**Synthesis of isoxazoles 3 in heptane (general procedure B)** **S9**

**Characterization data of isoxazoles 3** **S9-S12**

**Synthesis of amides 4-6 (general procedure)** **S13**

**Characterization data of amides 4-6** **S13**

**Synthesis of triazoles 7-10 (general procedure)** **S14**

**Characterization data of triazoles 7-10** **S14-S15**

**Synthesis of 1-(2,5-diphenyl-2*H*-1,2,3-triazol-4-yl)-2,2,2-trifluoroethanone (11)** **S16**

**Copies of all NMR spectra** **S17-S130**

**Copies of FT-IR spectra** **S131-S151**

**Computational Details** **S152-S154**

**Experimental section**

**General remarks.** $^{1}H$, $^{13}C$ and $^{19}F$ NMR spectra were recorded on Bruker AVANCE 400 MHz spectrometer in acetone-*d*$_6$, $CD_3CN$ and $CDCl_3$ at 400, 100 and 376 MHz respectively. Chemical shifts ($\delta$) in ppm are reported with the use of the residual acetone-*d*$_5$, $CHD_2CN$ and chloroform signals (2.04, 1.94 and 7.25 for $^{1}H$ and 29.8, 1.3, 77.0 for $^{13}C$) as internal reference. The $^{19}F$ chemical shifts were referenced to $C_6F_6$, (-162.9 ppm). The coupling constants ($J$) are given in Hertz (Hz). ESI-MS spectra were measured with an Orbitrap Elite instrument. One FT-IR spectrometer using consoles of internal reflection iS3 with ATR element from ZnSe, dip angle 45 °C. TLC analysis was performed on “Merck 60 $F_{254}$” plates. Column chromatography was performed on silica gel “Macherey-Nagel 0.063-0.2nm (Silica 60)”. In all cases gravity column chromatography was used.. All reagents were of reagent grade and were used as such or were distilled prior to use. $CF_3$-ynones **1** were prepared as reported previously. Melting points were determined on an Electrothermal 9100 apparatus.

**Investigation of reaction of $CF_3$-ynones with $NaN_3$ in various solvents.** A 4 mL vial with a screw cap was charged with $CF_3$-ynone **1a** (0.099 g, 0.5 mmol), corresponding solvent (2 mL, see Table 1) and $NaN_3$ (0.039 g, 0.6 mmol, 1.2 equiv.). The reaction mixture was stirred overnight using magnetic stirrer. Composition of the reaction mixture was established by $^{19}F$ NMR using $PhCF_3$ as a standard for calculation of the products yields. Attention! All manipulations with any azides demand significant care due to safety reasons!

**Table S1.** Investigation of the solvent nature in the reaction of sodium azide with $CF_3$-ynone **1**.

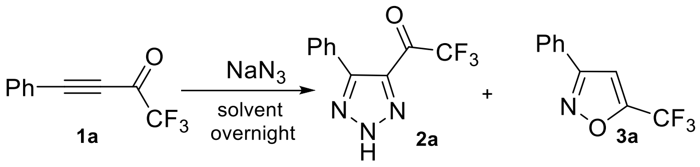

| Entry | Solvent | Yield of **2a**, %[a] | Yield of **3a**, % |
|---|---|---|---|
| 1 | PhMe | 2 | 12 |
| 2 | Acetone-$H_2O$ | 12 | 5 |
| 3 | MeCN | 26 | 7 |
| 4 | THF | 19 | 12 |
| 5 | EtOAc-$H_2O$ | 23 | 14 |
| 6 | DMF | 49 | 2 |
| 7 | DMSO | 48 | 1 |
| 8 | NMP | 52 | 2 |
| 9 | MeOH | 77 | 4 |
| 10 | EtOH | 85(81[b]) | 3 |

[a]- by $^{19}$F NMR; [b]-isolated yield.

**Synthesis of triazoles 2 (general procedure).** A 8 mL vial with a screw cap was charged with corresponding $CF_3$-ynone **1** (1 mmol), ethanol (4 mL) and $NaN_3$ (0.078 g, 1.2 mmol). The reaction mixture was stirred overnight using magnetic stirrer. Next, ethanol was evaporated in vacuo; the residue was suspended in the mixture of heptane-ethylacetate (1:1, 0.5-1 mL) and purified by column chromatography using gradient eluation by mixtures of heptane-ethylacetate (9:1, 3:1 and 1:1). Evaporation of the solvents afforded pure triazoles **2**.

**2,2,2-Trifluoro-1-(5-phenyl-2*H*-1,2,3-triazol-4-yl)ethanone (2a)**. Obtained from **1a** (0.202 g, 1.02 mmol). White crystals, m.p. 105-108 °C, yield 0.199 g (81%). $^{1}$H NMR ($CDCl_3$, 400.1 MHz): δ 12.75 (br.s, 1H), 7.89-7.75 (m, 2H), 7.58-7.45 (m, 3H). $^{13}$C{$^{1}$H} NMR ($CDCl_3$, 100.6 MHz): δ 174.4 (q, $^2J_{CF}$ = 37.2 Hz), 147.7, 136.1, 131.3, 129.0, 128.9, 125.1, 116.2 (q, $^1J_{CF}$ = 290.6 Hz). $^{19}$F NMR ($CDCl_3$, 376.5 MHz): δ -75.0 (s, 3F). $^{1}$H NMR ($CD_3CN$, 400.1 MHz): δ 12.75 (br.s, 1H), 7.85-7.74 (m, 2H), 7.61-7.49 (m, 3H). $^{13}$C{$^{1}$H} NMR ($CD_3CN$, 100.6 MHz): δ 175.2 (q, $^2J_{CF}$ = 36.3 Hz), 148.1, 137.0, 131.7, 130.1, 129.6, 127.1, 117.4 (q, $^1J_{CF}$ = 290.2 Hz). $^{19}$F NMR ($CD_3CN$, 376.5 MHz): δ -73.0 (s, 3F). $^{1}$H NMR (Acetone-d6,

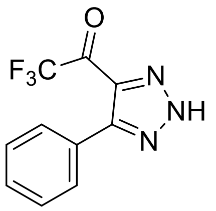

400.1 MHz): δ, 8.06-7.98 (m, 2H), 7.41-7.28 (m, 3H), 5.14 (br.s, 1H). $^{13}C\{^1H\}$ NMR (Acetone-d6, 100.6 MHz): δ 174.7 (q, $^2J_{CF}$ = 33.4 Hz), 152.0, 136.1, 133.5, 129.5, 128.51, 128.45, 118.6 (q, $^1J_{CF}$ = 291.6 Hz). $^{19}F$ NMR (Acetone-d6, 376.5 MHz): δ -70.7 (s, 3F).

HRMS (ESI-TOF): m/z $[M+H]^+$ Calcd for $C_{10}H_7F_3N_3O^+$: 242.0536; found: 242.0536.

IR (ν, $cm^{-1}$): 1719 (C=O).

**2,2,2-Trifluoro-1-(5-(4-methoxyphenyl)-2*H*-1,2,3-triazol-4-yl)ethanone (2b)**. Obtained from **1b** (0.228 g, 1 mmol). Pale brown solid, m.p. 100-102 °C, yield 0.216 g (80%). $^1H$ NMR ($CDCl_3$, 400.1 MHz): δ 13.87 (br.s, 1H), 7.84 (d, 2H, $^3J$ = 8.8 Hz), 7.97 (d, 2H, $^3J$ = 8.8 Hz), 3.84 (s, 3H). $^{13}C\{^1H\}$ NMR ($CDCl_3$, 100.6 MHz): δ 174.2 (q, $^2J_{CF}$ = 37.0 Hz), 162.0, 146.7, 135.5, 130.7, 116.5, 116.3 (q, $^1J_{CF}$ = 290.7 Hz), 114.3, 55.4. $^{19}F$ NMR ($CDCl_3$, 376.5 MHz): δ -74.7 (s, 3F). $^1H$ NMR (Acetone-d6, 400.1 MHz): δ 13.41 (br.s, 1H), 7.91 (d, 2H, $^3J$ = 7.9 Hz), 7.08 (d, 2H, $^3J$ = 8.0 Hz), 3.88 (s, 3H). $^{13}C\{^1H\}$ NMR (Acetone-d6, 100.6 MHz): δ 175.0 (q, $^2J_{CF}$ = 36.0 Hz), 162.4, 147.9, 136.2, 131.5, 127.8, 117.5 (q, $^1J_{CF}$ = 290.6 Hz), 114.7, 55.7. $^{19}F$ NMR (Acetone-d6, 376.5 MHz): δ -72.6 (s, 3F).

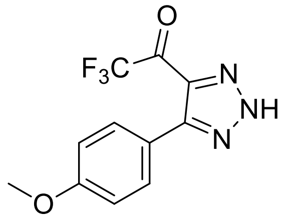


HRMS (ESI-TOF): m/z $[M+H]^+$ Calcd for $C_{11}H_9F_3N_3O_2^+$: 272.0641; found: 272.0641.

IR (ν, $cm^{-1}$): 1719 (C=O).

**2,2,2-Trifluoro-1-(5-(4-methylthio)phenyl)-2*H*-1,2,3-triazol-4-yl)ethanone (2c)**. Obtained from **1c** (0.123 g, 0.504 mmol). Pale yellow-brown solid, m.p. 102-104 °C, yield 0.110 g (76%). $^1H$ NMR ($CDCl_3$, 400.1 MHz): δ 13.99 (br.s, 1H), 7.75 (d, 2H, $^3J$ = 8.4 Hz), 7.26 (d, 2H, $^3J$ = 7.9 Hz), 2.5 (s, 3H). $^{13}C\{^1H\}$ NMR ($CDCl_3$, 100.6 MHz): δ 174.2 (q, $^2J_{CF}$ = 37.2 Hz), 147.0, 143.7, 135.7, 129.1, 125.5, 120.7 (d, $^4J_{CF}$ = 1.6 Hz), 116.2 (q, $^1J_{CF}$ = 290.8 Hz), 14.7. $^{19}F$ NMR ($CDCl_3$, 376.5 MHz): δ -74.8 (s, 3F).

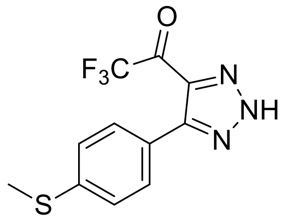


HRMS (ESI-TOF): m/z $[M+H]^+$ Calcd for $C_{11}H_9F_3N_3OS^+$: 288.0413; found: 288.0417.

IR (ν, $cm^{-1}$): 1722 (C=O).

**2,2,2-Trifluoro-1-(5-(*p*-tolyl)-2*H*-1,2,3-triazol-4-yl)ethanone (2d)**. Obtained from **1d** (0.424 g, 2 mmol). Pale beige powder, m.p. 128-130 °C, yield 0.395 g (77%). $^{1}$H NMR ($CDCl_3$, 400.1 MHz): δ 13.41 (br.s, 1H), 7.72 (d, 2H, $^{3}J$ = 7.9 Hz), 7.28 (d, 2H, $^{3}J$ = 8.0 Hz), 2.41 (s, 3H). $^{13}$C{$^{1}$H} NMR ($CDCl_3$, 100.6 MHz): δ 174.2 (q, $^{2}J_{CF}$ = 37.1 Hz), 146.9 (q, $^{4}J_{CF}$ = 2.3 Hz), 142.0, 135.6, 129.5, 128.9, 121.4, 116.2 (q, $^{1}J_{CF}$ = 290.7 Hz), 21.4. $^{19}$F NMR ($CDCl_3$, 376.5 MHz): δ -75.0 (s, 3F).

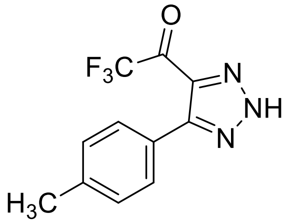


HRMS (ESI-TOF): m/z [M+H]$^+$ Calcd for $C_{11}H_9F_3N_3O^+$: 256.0692; found: 256.0692.

IR (ν, cm$^{-1}$): 1719 (C=O).

**2,2,2-Trifluoro-1-(5-(4-fluorophenyl)-2*H*-1,2,3-triazol-4-yl)ethanone (2e)**. Obtained from **1e** (0.110 g, 0.509 mmol). Pale beige solid, m.p. 85-87 °C, yield 0.092 g (70%). $^{1}$H NMR ($CDCl_3$, 400.1 MHz): δ 11.12 (br.s, 1H), 7.90-7.79 (m, 2H), 7.15 (t, 2H, $^{3}J$ = 8.3 Hz). $^{13}$C{$^{1}$H} NMR ($CDCl_3$, 100.6 MHz): δ 174.3 (q, $^{2}J_{CF}$ = 37.4 Hz), 164.3 (d, $^{1}J_{CF}$ = 252.8 Hz), 147.5, 136.0, 131.3 (d, $^{3}J_{CF}$ = 8.8 Hz), 121.7, 116.2 (q, $^{1}J_{CF}$ = 290.5 Hz), 116.0 (d, $^{2}J_{CF}$ = 22.1 Hz). $^{19}$F NMR ($CDCl_3$, 376.5 MHz): δ -75.0 (s, 3F), 109.1 (s, 1F).

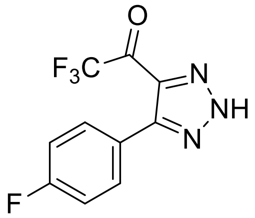


HRMS (ESI-TOF): m/z [M+H]$^+$ Calcd for $C_{10}H_6F_4N_3O^+$: 260.0442; found: 260.0447.

IR (ν, cm$^{-1}$): 1726 (C=O).

**1-(5-(4-Chlorophenyl)-2*H*-1,2,3-triazol-4-yl)-2,2,2-trifluoroethanone (2f)**. Obtained from **1f** (0.233 g, 1 mmol). Pale beige solid, m.p. 83-85 °C, yield 0.203 g (74%). $^{1}$H NMR ($CDCl_3$, 400.1 MHz): δ 14.05 (br.s, 1H), 7.75 (d, 2H, $^{3}J$ = 8.3 Hz), 7.42 (d, 2H, $^{3}J$ = 8.2 Hz). $^{13}$C{$^{1}$H} NMR ($CDCl_3$, 100.6 MHz): δ 174.3 (q, $^{2}J_{CF}$ = 37.3 Hz), 147.1, 137.5, 136.0, 130.3, 129.1, 123.6, 116.1 (q, $^{1}J_{CF}$ = 290.6 Hz). $^{19}$F NMR ($CDCl_3$, 376.5 MHz): δ -74.9 (s, 3F).

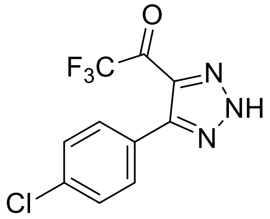


HRMS (ESI-TOF): m/z [M+H]$^+$ Calcd for $C_{10}H_6[^{35}Cl]F_3N_3O^+$: 276.0146; found: 276.0151; Calcd for $C_{10}H_6[^{37}Cl]F_3N_3O^+$: 278.0117; found: 278.0121.

IR (ν, cm$^{-1}$): 1726 (C=O).

**1-(5-(4-Bromophenyl)-2*H*-1,2,3-triazol-4-yl)-2,2,2-trifluoroethanone (2g)**. Obtained from **1g** (0.186 g, 0.671 mmol). Pale beige solid, m.p. 82-84 °C, yield 0.166 g (77%). $^1$H NMR ($CDCl_3$, 400.1 MHz): δ 13.39 (br.s, 1H), 7.69 (d, 2H, $^3J$ = 8.5 Hz), 7.59 (d, 2H, $^3J$ = 8.5 Hz). $^{13}$C{$^1$H} NMR ($CDCl_3$, 100.6 MHz): δ 174.3 (q, $^2J_{CF}$ = 37.7 Hz), 147.5, 136.1, 132.1, 130.5, 125.8, 124.4, 116.1 (q, $^1J_{CF}$ = 290.3 Hz). $^{19}$F NMR ($CDCl_3$, 376.5 MHz): δ -75.0 (s, 3F).

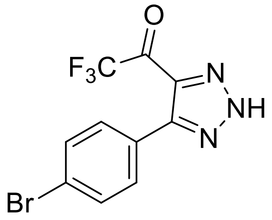


HRMS (ESI-TOF): m/z [M+H]$^+$ Calcd for $C_{10}H_6[^{79}Br]F_3N_3O^+$: 319.9641; found: 319.9646; m/z [M+H]$^+$ Calcd for $C_{10}H_6[^{81}Br]F_3N_3O^+$: 321.9620; found: 321.9625.

IR (ν, cm$^{-1}$): 1723 (C=O).

**1-(5-(4-(*Tret*-butyl)phenyl)-2*H*-1,2,3-triazol-4-yl)-2,2,2-trifluoroethanone (2h)**. Obtained from **1h** (0.130 g, 0.512 mmol). Light brown powder, m.p. 140-143 °C, yield 0.123 g (81%). $^1$H NMR ($CDCl_3$, 400.1 MHz): δ 14.12 (br.s, 1H), 7.82 (d, 2H, $^3J$ = 8.3 Hz), 7.53 (d, 2H, $^3J$ = 8.2 Hz), 1.35 (s, 9H). $^{13}$C{$^1$H} NMR ($CDCl_3$, 100.6 MHz): δ 174.3 (q, $^2J_{CF}$ = 37.1 Hz), 155.1, 147.0, 135.9, 128.8, 126.0, 121.6, 116.3 (q, $^1J_{CF}$ = 290.7 Hz), 35.0, 31.0. $^{19}$F NMR ($CDCl_3$, 376.5 MHz): δ -74.9 (s, 3F).

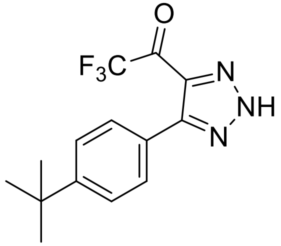


HRMS (ESI-TOF): m/z [M+H]$^+$ Calcd for $C_{14}H_{15}F_3N_3O^+$: 298.1162; found: 298.1166.

IR (ν, cm$^{-1}$): 1713 (C=O).

**2,2,2-Trifluoro-1-(5-(4-trifluoromethyl)phenyl)-2*H*-1,2,3-triazol-4-yl)ethanone (2i)**. Obtained from **1i** (0.268 g, 1.01 mmol). Pale yellow solid, m.p. 67-69 °C, yield 0.230 g (74%). $^1$H NMR ($CDCl_3$, 400.1 MHz): δ 10.94 (br.s, 1H), 7.94 (d, 2H, $^3J$ = 8.2 Hz), 7.71 (d, 2H, $^3J$ = 8.3 Hz). $^{13}$C{$^1$H} NMR ($CDCl_3$, 100.6 MHz): δ 174.5 (q, $^2J_{CF}$ = 37.8 Hz), 148.2, 136.5, 132.6 (q, $^2J_{CF}$ = 32.9 Hz), 129.8, 129.5, 125.7 (q, $^3J_{CF}$ = 3.6 Hz), 123.6 (q, $^1J_{CF}$ = 272.4 Hz), 116.1 (q, $^1J_{CF}$ = 290.4 Hz). $^{19}$F NMR ($CDCl_3$, 376.5 MHz): δ -64.2 (s, 3F), -75.1 (s, 3F).

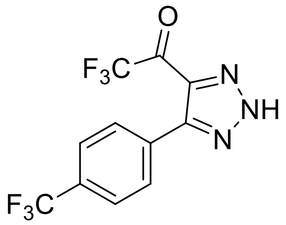


HRMS (ESI-TOF): m/z [M+H]$^+$ Calcd for $C_{11}H_6F_6N_3O^+$: 310.0410; found: 310.0416.

IR (ν, cm$^{-1}$): 1718 (C=O).

**1-(5-(3,4-Dimethylphenyl)-2*H*-1,2,3-triazol-4-yl)-2,2,2-trifluoroethanone (2j)**. Obtained from **1j** (0.118 g, 0.522 mmol). Pale brown solid, m.p. 76-78 °C, yield 0.114 g (81%). $^{1}$H NMR ($CDCl_3$, 400.1 MHz): δ 14.05 (br.s, 1H), 7.63-7.50 (m, 2H), 7.21 (d, 1H, $^{3}J$ = 7.7 Hz), 2.30 (s, 3H), 2.28 (s, 3H). $^{13}$C{$^{1}$H} NMR ($CDCl_3$, 100.6 MHz): δ 174.2 (q, $^{2}J_{CF}$ = 37.1 Hz), 146.9, 140.7, 137.4, 135.6, 130.1, 129.8, 126.4, 121.8, 116.3 (q, $^{1}J_{CF}$ = 290.7 Hz), 19.8, 19.6. $^{19}$F NMR ($CDCl_3$, 376.5 MHz): δ -74.8 (s, 3F).

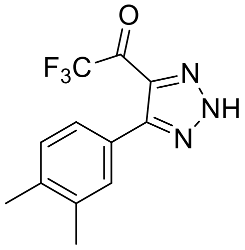


HRMS (ESI-TOF): m/z [M+H]$^+$ Calcd for $C_{12}H_{11}F_3N_3O^+$: 270.0849; found: 270.0856.

IR (ν, cm$^{-1}$): 1722 (C=O).

**1-(5-(2,3-dihydrobenzo[*b*][1,4]dioxin-6-yl)-2*H*-1,2,3-triazol-4-yl)-2,2,2-trifluoroethanone (2k)**. Obtained from **1k** (0.240 g, 1.025 mmol). Slightly yellow oil, yield 0.218 g (71%). $^{1}$H NMR ($CDCl_3$, 400.1 MHz): δ 13.85 (br.s, 1H), 7.35 (s, 1H), 7.31 (d, 1H, $^{3}J$ = 8.5 Hz), 6.90 (d, 1H, $^{3}J$ = 8.4 Hz), 4.31-4.23 (m, 4H). $^{13}$C{$^{1}$H} NMR ($CDCl_3$, 100.6 MHz): δ 174.3 (q, $^{2}J_{CF}$ = 37.0 Hz), 146.9, 146.2, 143.5, 135.6, 122.5, 120.6, 118.1, 117.7, 117.8 (q, $^{4}J_{CF}$ = 3.1 Hz), 116.3 (q, $^{1}J_{CF}$ = 290.7 Hz), 64.5, 64.1. $^{19}$F NMR ($CDCl_3$, 376.5 MHz): δ -74.8 (s, 3F).

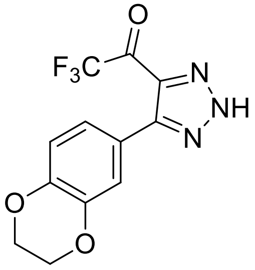


HRMS (ESI-TOF): m/z [M+H]$^+$ Calcd for $C_{12}H_9F_3N_3O_3^+$: 300.0591; found: 300.0595.

IR (ν, cm$^{-1}$): 1717 (C=O).

**1-(5-(2-Chlorophenyl)-2*H*-1,2,3-triazol-4-yl)-2,2,2-trifluoroethanone (2l)**. Obtained from **1l** (0.237 g, 1.015 mmol). Pale brown viscous oil, yield 0.164 g (59%). $^{1}$H NMR ($CDCl_3$, 400.1 MHz): δ 13.55 (br.s, 1H), 7.80-7.25 (m, 4H). $^{13}$C{$^{1}$H} NMR ($CDCl_3$, 100.6 MHz): δ 173.9 (q, $^{2}J_{CF}$ = 38.1 Hz), 145.0, 137.7, 133.5, 131.8, 131.3, 129.9, 126.9, 125.3, 115.8 (q, $^{1}J_{CF}$ = 290.2 Hz). $^{19}$F NMR ($CDCl_3$, 376.5 MHz): δ -75.6 (s, 3F).

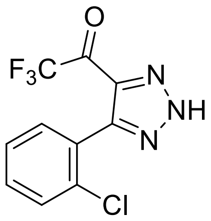


HRMS (ESI-TOF): m/z [M+H]$^+$ Calcd for $C_{10}H_6[^{35}Cl]F_3N_3O^+$: 276.0146; found: 276.0149; Calcd for $C_{10}H_6[^{37}Cl]F_3N_3O^+$: 278.0117; found: 278.0118.

IR (ν, cm$^{-1}$): 1726 (C=O).

**2,2,2-Trifluoro-1-(5-(2-methoxyphenyl)-2*H*-1,2,3-triazol-4-yl)ethanone (2m)**. Obtained from **1m** (0.195 g, 0.867 mmol). Pale brown solid, m.p. 130-132 °C, yield 0.097 g (41%). $^1$H NMR ($CDCl_3$, 400.1 MHz): δ 10.67 (br.s, 1H), 7.61 (dd, 1H, $^3J$ = 8.5 Hz, $^4J$ = 8.5 Hz), 7.42-7.38 (m, 1H), 7.00-6.94 (m, 2H), 3.74 (s, 3H). $^{13}C\{^1H\}$ NMR ($CDCl_3$, 100.6 MHz): δ 174.6 (q, $^2J_{CF}$ = 36.5 Hz), 156.4, 142.0, 136.6, 132.1, 130.7, 120.3, 116.0 (q, $^1J_{CF}$ = 290.6 Hz), 113.7, 110.8, 55.2. $^{19}$F NMR ($CDCl_3$, 376.5 MHz): δ -74.7 (s, 3F).

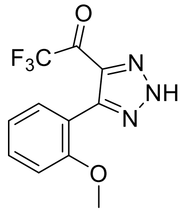


HRMS (ESI-TOF): m/z [M+H]$^+$ Calcd for $C_{11}H_9F_3N_3O_2^+$: 272.0641; found: 272.0625.

**2,2,2-Trifluoro-1-(5-(4-methoxynaphthalen-1-yl)-2*H*-1,2,3-triazol-4-yl)ethanone (2n)**. Obtained from **1n** (0.215 g, 0.773 mmol). Slightly yellow oil, yield 0.127 g (51%). $^1$H NMR ($CDCl_3$, 400.1 MHz): δ 13.00 (br.s, 1H), 8.36-8.30 (m, 1H), 7.53-7.36 (m, 4H), 6.81 (d, 1H, $^3J$ = 7.9 Hz), 4.04 (s, 3H). $^{13}C\{^1H\}$ NMR ($CDCl_3$, 100.6 MHz): δ 173.9 (q, $^2J_{CF}$ = 37.6 Hz), 157.7, 145.8, 137.8, 131.8, 129.6, 127.9, 125.9, 125.5, 123.7, 122.8, 116.0 (q, $^1J_{CF}$ = 290.4 Hz), 114.4 103.0, 55.7. $^{19}$F NMR ($CDCl_3$, 376.5 MHz): δ -75.4 (s, 3F).

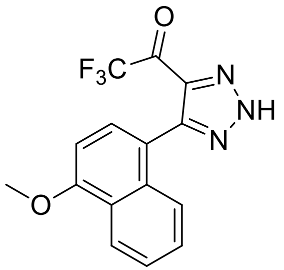


HRMS (ESI-TOF): m/z [M+H]$^+$ Calcd for $C_{15}H_{11}F_3N_3O_2^+$: 322.0798; found: 322.0805.

IR (ν, cm$^{-1}$): 1720 (C=O).

**2,2,2-Trifluoro-1-(5-octyl-2*H*-1,2,3-triazol-4-yl)ethanone (2o)**. Obtained from **1o** (0.122 g, 0.521 mmol). Pale brown solid, m.p. 66-68 °C, yield 0.082 g (56%). $^1$H NMR ($CDCl_3$, 400.1 MHz): δ 13.73 (br.s, 1H), 3.21-3.07 (m, 2H), 1.75-1.65 (m, 2H), 1.37-1.19 (m, 10H), 0.87-0.81 (m, 3H). $^{13}C\{^1H\}$ NMR ($CDCl_3$, 100.6 MHz): δ 174.7 (q, $^2J_{CF}$ = 37.1 Hz), 149.3, 136.8, 116.1 (q, $^1J_{CF}$ = 290.4 Hz), 31.7, 29.1, 29.0, 28.3, 24.1, 22.6, 14.0. $^{19}$F NMR ($CDCl_3$, 376.5 MHz): δ -75.4 (s, 3F).

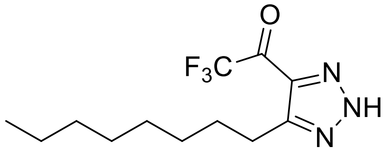


HRMS (ESI-TOF): m/z [M+H]$^+$ Calcd for $C_{12}H_{19}F_3N_3O^+$: 278.1475; found: 278.1475.

IR (ν, cm$^{-1}$): 1721 (C=O).

**Synthesis of triazoles 2a,b (sub-gram scale, general procedure).** A 25 mL one-neck round-bottom flask was charged with corresponding $CF_3$-ynone **1** (5 mmol), ethanol (5 mL) and cooled down to 0 ºC at ice bath. Next, $NaN_3$ (0.390 g, 6 mmol) was added and the reaction mixture was stirred overnight using magnetic stirrer. Next, ethanol was evaporated *in vacuo*. The residue was dispersed in water (4 mL) and washed twice with a mixture heptane-ethylacetate

(1:1, 1 mL). Organic phase was thrown away; water phase was acidified by dropwise addition of concentrated HCl (0.8 mL, ~9-10 mmol) to form a precipitate. Water phase was decanted and the precipitate left was dried *in vacuo*.

**2,2,2-Trifluoro-1-(5-phenyl-2*H*-1,2,3-triazol-4-yl)ethanone (2a), sub-gram scale synthesis**. Obtained from **1a** (1.019 g, 5.146 mmol). White crystals, m.p. 105-108 °C, yield 0.994 g (80%). Calculation of E-factor: E-factor=(mass of all reagents and solvents used)/(mass of the isolated product)=(1.019+0.390+5mL(EtOH)*0.789+4mL($H_2O$)*1+ 2mL(Heptane)*0.684+2mL(EtOAc)*0.902+0.8 mL (HCl)*1.2))/(0.994 g)=13.509/0.994=13.6.

**2,2,2-Trifluoro-1-(5-(4-methoxyphenyl)-2*H*-1,2,3-triazol-4-yl)ethanone (2b), sub-gram scale synthesis**. Obtained from **1b** (0.674 g, 1 mmol). Pale brown solid, m.p. 100-102 °C, yield 0.681 g (85%).

**Investigation of reaction of $CF_3$-ynones with $NaN_3$ in various solvents in the presence of acids.** A 4 mL vial with a screw cap was charged with $CF_3$-ynone **1a** (0.099 g, 0.5 mmol), corresponding solvent (2 mL, see Table SI2), corresponding acid (for quantity of acid see Table SI2) and $NaN_3$ (for quantity see Table SI2). The reaction mixture was stirred for 1 day using magnetic stirrer. Composition of the reaction mixture was established by $^{19}$F NMR using $PhCF_3$ as a standard for calculation of the products yields.

**Table S2.** Investigation of the solvent and acid nature in the reaction of sodium azide with $CF_3$-ynone **1a**.

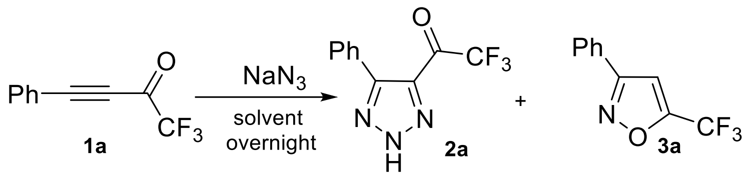


| Entry | Solvents | Reagents | Yield of **2a**, %[a] | Yield of **3a**, %[a] |
|---|---|---|---|---|
| 1 | EtOH | $NaN_3$(2 eq)/ $HCO_2H$(4 eq.) | 3 | 42 |
| 2 | EtOH | $NaN_3$(2 eq)/ AcOH(4 eq.) | 7 | 42 (34[b]) |
| 3 | EtOH(dry) | $NaN_3$(2 eq)/ AcOH(4 eq.) | 4 | 42 |
| 4 | EtOH | $NaN_3$(2 eq)/ $ClCH_2CO_2H$(4 eq.) | 4 | 41 |
| 5 | EtOH | $NaN_3$(2 eq)/ TFA(4 eq.) | 10 | 36 |
| 6 | EtOH | $NaN_3$(2 eq)/ $MeSO_3H$(2.1 eq.) | 9 | 9 |
| 7 | EtOH | $NaN_3$(1.2 eq)/ HCl (~1 eq) | 2 | 3 |
| 8 | Heptane-$H_2O$ (10/1) | $NaN_3$(2 eq)/ AcOH(4 eq.) | 2 | 35 |
| 9 | Heptane-$H_2O$ (10/1) | $NaN_3$(5 eq)/ AcOH(6 eq.) | 2 | 40(36[b]) |
| 10 | Heptane-$H_2O$ (10/1) | $NaN_3$(2 eq)/ $HCO_2H$(4 eq.) | 2 | 35 |
| 11 | Heptane-$H_2O$ (10/1) | $NaN_3$(2 eq)/ $ClCH_2CO_2H$(4 eq.) | 1 | 35 |
| 12 | Heptane-$H_2O$ (10/1) | $NaN_3$(4 eq)/ TEBAC (1 eq.) | - | traces |
| 13 | MeOH | $NaN_3$(2 eq)/ AcOH(4 eq.) | 5 | 35 |

| | | | | |
|---|---|---|---|---|
| 14 | dioxane | $NaN_3$(5 eq), AcOH (5 eq.) | 1 | 41 |
| 15 | TCE | $NaN_3$(2 eq)/ AcOH(4 eq.) | - | 34 |
| 16 | toluene | $NaN_3$(5 eq), AcOH (5 eq.) | traces | 41 (30[b]) |
| 17 | EtOAc | $NaN_3$(5 eq), AcOH (6 eq.) | 2 | 40 |
| 18 | $CF_3CH_2OH$ | $NaN_3$(1.2 eq) | - | 37 |
| 19 | AcOH | $NaN_3$(5 eq) | traces | 36 |
| 20 | AcOH | $NaN_3$(1.2 eq) | traces | 35 |
| 21 | Heptane-$H_2O$ (10/1) | $NaN_3$(2 eq)/ $MeSO_3H$(2.1 eq.) | 2 | 4 (conversion 7%) |
| 22 | dioxane | $NaN_3$(1.2 eq) $MeSO_3H$ (1 eq) | 5 | 3 (conversion 11%) |

[a]- by $^{19}F$ NMR; [b]-isolated yield.

**Synthesis of isoxazoles 3 in ethanol (general procedure A).** A 8 mL vial with a screw cap was charged with corresponding $CF_3$-ynone **1** (0.5 mmol), ethanol (2 mL), acetic acid (0.120 g, 2 mmol, 4 equiv.) and $NaN_3$ (0.065 g, 1 mmol, 2 equiv.). The reaction mixture was stirred overnight using magnetic stirrer. Next, ethanol was evaporated in vacuo at room temperature (careful! isoxazoles 4a, 4x are quite volatile); the residue was suspended in the mixture of heptane-ethylacetate (3:1, 0.5) and passed through a short silica gel pad using gradient eluating by heptane and mixture of heptane-ethylacetate (9:1). Evaporation of the solvents afforded pure isoxazoles **3**.

**Synthesis of isoxazoles 3 in heptane (general procedure B).** A 8 mL vial with a screw cap was charged with corresponding $CF_3$-ynone **1** (0.5 mmol), heptane (4 mL), water (0.5 mL)acetic acid (0.120 g, 2 mmol, 4 equiv.) and $NaN_3$ (0.065 g, 1 mmol, 2 equiv.). The reaction mixture was stirred overnight using magnetic stirrer. Next, heptane phase was separated, water phase was extracted with a mixture of heptane-ethylacetate (9:1, 2x0.5 mL) combined extract was passed through a short silica gel pad using gradient eluating by heptane followed by mixtures of heptane-ethylacetate (9:1). Evaporation of the solvents afforded pure isoxazoles **3**.

**3-Phenyl-5-(trifluoromethyl)isoxazole (3a)**. Obtained from **1a** (0.099 g, 0.5 mmol (A); 0.100 g, 0.505 mmol (B)). White crystals, m.p. 74-77 °C (Lit.: 73.3-74 °C, 76-78 °C), yield 0.037 g (35%, (A)); 0.039 g (36%, (B)). $^{1}$H NMR ($CDCl_3$, 400.1 MHz): δ 7.85-7.76 (m, 2H), 7.54-7.44 (m, 3H), 7.0 (s, 1H). $^{13}$C{$^{1}$H} NMR ($CDCl_3$, 100.6 MHz): δ 162.5, 159.1 (q, $^{2}J_{CF}$ = 42.6 Hz), 130.9, 129.2, 127.3, 126.9, 117.9 (q, $^{1}J_{CF}$ = 270.3 Hz), 103.4 (q, $^{4}J_{CF}$ = 2.0 Hz). $^{19}$F NMR ($CDCl_3$, 376.5 MHz): δ -65.3 (s, 3F). NMR data are in agreement with those in the literature[Error! Bookmark not defined.].

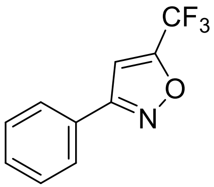

**3-(4-Methoxyphenyl)-5-(trifluoromethyl)isoxazole (3b)**. Obtained from **1b** (0.114 g, 0.5 mmol (A); 0.110 g, 0.482 mmol (B)). Pale yellow solid, m.p. 72-74 °C (Lit.: 72-75 °C[Error! Bookmark not defined.]), yield 0.021 g (17%, (A)); 0.019 g (16%, (B)). $^{1}$H NMR ($CDCl_3$, 400.1 MHz): δ 7.74 (d, 2H, $^{3}J$ = 8.9 Hz), 6.99 (d, 2H, $^{3}J$ = 8.9 Hz), 6.94 (*pseudo*-d, 1H, $^{4}J$ = 0.8 Hz), 3.86 (s, 3H). $^{13}$C{$^{1}$H} NMR ($CDCl_3$, 100.6 MHz): δ 162.1, 161.7, 158.9 (q, $^{2}J_{CF}$ = 42.4 Hz), 128.4, 119.7, 117.9 (q, $^{1}J_{CF}$ = 270.0 Hz), 114.6, 103.2 (q, $^{4}J_{CF}$ = 1.8 Hz), 55.4. $^{19}$F NMR ($CDCl_3$, 376.5 MHz): δ -65.4 (d, 3F, $J$ = 0.9 Hz). NMR data are in agreement with those in the literature[Error! Bookmark not defined.].

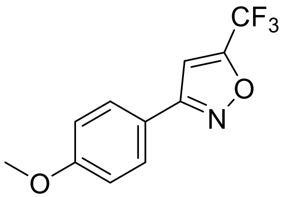

**3-(4-Methylphenyl)-5-(trifluoromethyl)isoxazole (3d)**. Obtained from **1d** (0.106 g, 0.5 mmol (A); 0.115 g, 0.542 mmol (B)). Pale yellow solid, m.p. 78-80 °C (Lit.: 79-80 °C), yield 0.035 g (31%, (A)); 0.036 g (29%, (B)). $^{1}$H NMR ($CDCl_3$, 400.1 MHz): δ 7.70 (d, 2H, $^{3}J$ = 8.0 Hz), 7.29 (d, 2H, $^{3}J$ = 8.0 Hz), 6.97 (*pseudo*-d, 1H, $^{4}J$ = 0.9 Hz), 2.41 (s, 3H). $^{13}$C{$^{1}$H} NMR ($CDCl_3$, 100.6 MHz): δ 162.5, 159.0 (q, $^{2}J_{CF}$ = 42.4 Hz), 141.2, 129.9, 126.8, 124.4, 117.9 (q, $^{1}J_{CF}$ = 270.4 Hz), 103.3 (q, $^{4}J_{CF}$ = 2.0 Hz), 21.4. $^{19}$F NMR ($CDCl_3$, 376.5 MHz): δ -65.4 (d, 3F, $J$ = 0.9 Hz). NMR data are in agreement with those in the literature[Error! Bookmark not defined.].

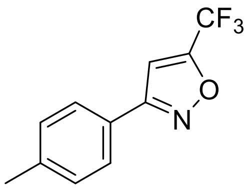

**3-(4-Fluorophenyl)-5-(trifluoromethyl)isoxazole (3e)**. Obtained from **1e** (0.113 g, 0.5 mmol (A); 0.113 g, 0.5 mmol (B)). Pale beige crystals, m.p. 49-50 °C (Lit.: 48 °C), yield 0.036 g (31%, (A)); 0.035 g (30%, (B)). $^{1}$H NMR ($CDCl_3$, 400.1 MHz): δ 7.83-7.78 (m, 2H), 7.21-7.15 (m, 2H), 6.97 (*pseudo*-d, 1H, $^{4}J$ = 0.9 Hz). $^{13}$C{$^{1}$H} NMR ($CDCl_3$, 100.6 MHz): δ 164.3 (d, $^{1}J_{CF}$ = 251.7 Hz), 161.6, 159.4 (q, $^{2}J_{CF}$ = 42.7 Hz), 129.0 (d, $^{3}J_{CF}$ = 8.7 Hz), 123.5 (d, $^{4}J_{CF}$ = 3.5 Hz), 117.8 (q, $^{1}J_{CF}$ = 270.5 Hz), 116.4 (d, $^{2}J_{CF}$ = 22.1 Hz), 103.3 (q, $^{4}J_{CF}$ = 2.2 Hz). $^{19}$F NMR ($CDCl_3$, 376.5 MHz): δ -65.4 (d, 3F, $J$ = 0.8 Hz), -109.9--110.0 (m, 1F). NMR data are in agreement with those in the literature[Error! Bookmark not defined.].

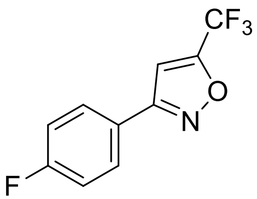

**3-(4-Chlorophenyl)-5-(trifluoromethyl)isoxazole (3f)**. Obtained from **1f** (0.117 g, 0.503 mmol (A); 0.114 g, 0.49 mmol (B)). Pale yellow solid, m.p. 51-53 °C (Lit.: 61-62 °C[Error! Bookmark not defined.]), yield 0.047 g (38%, (A)); 0.042 g (35%, (B)). $^1$H NMR ($CDCl_3$, 400.1 MHz): δ 7.74 (d, 2H, $^3J$ = 8.7 Hz), 7.46 (d, 2H, $^3J$ = 8.7 Hz), 6.98 (*pseudo*-d, 1H, $^4J$ = 0.9 Hz). $^{13}$C{$^1$H} NMR ($CDCl_3$, 100.6 MHz): δ 161.5, 159.5 (q, $^2J_{CF}$ = 42.6 Hz), 137.1, 129.5, 128.2, 125.7, 117.7 (q, $^1J_{CF}$ = 270.3 Hz), 103.3 (q, $^4J_{CF}$ = 2.0 Hz). $^{19}$F NMR ($CDCl_3$, 376.5 MHz): δ -65.4 (d, 3F, $J$ = 0.9 Hz). NMR data are in agreement with those in the literature[Error! Bookmark not defined.].

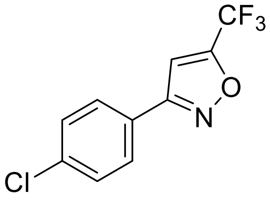


**3-(4-Bromophenyl)-5-(trifluoromethyl)isoxazole (3g)**. Obtained from **1g** (0.140 g, 0.505 mmol (A); 0.143 g, 0.516 mmol (B)). Colorless solid, m.p. 71-73 °C (Lit.: 63-64 °C[Error! Bookmark not defined.]), yield 0.053 g (36%, (A)); 0.051 g (34%, (B)). $^1$H NMR ($CDCl_3$, 400.1 MHz): δ 7.68 (d, 2H, $^3J$ = 8.6 Hz), 7.62 (d, 2H, $^3J$ = 8.6 Hz), 6.98 (*pseudo*-d, 1H, $^4J$ = 0.8 Hz). $^{13}$C{$^1$H} NMR ($CDCl_3$, 100.6 MHz): δ 161.7, 159.5 (q, $^2J_{CF}$ = 42.8 Hz), 132.5, 128.4, 126.2, 125.4, 117.7 (q, $^1J_{CF}$ = 270.4 Hz), 103.3 (q, $^4J_{CF}$ = 2.0 Hz). $^{19}$F NMR ($CDCl_3$, 376.5 MHz): δ -65.4 (d, 3F, $J$ = 0.6 Hz). NMR data are in agreement with those in the literature[Error! Bookmark not defined.].

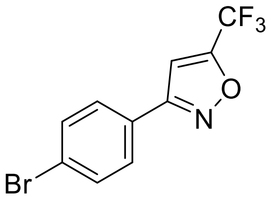


**3-(4-(*tert*-Butyl)phenyl)-5-(trifluoromethyl)isoxazole (3h)**. Obtained from **1h** (0.121 g, 0.476 mmol (A); 0.122 g, 0.48 mmol (B)). Pale yellow viscous oil, yield 0.041 g (32%, (A)); 0.033 g (26%, (B)). $^1$H NMR ($CDCl_3$, 400.1 MHz): δ 7.74 (d, 2H, $^3J$ = 8.7 Hz), 7.51 (d, 2H, $^3J$ = 8.7 Hz), 6.97 (*pseudo*-d, 1H, $^4J$ = 0.9 Hz), 2.41 (s, 3H). $^{13}$C{$^1$H} NMR ($CDCl_3$, 100.6 MHz): δ 162.4, 159.0 (q, $^2J_{CF}$ = 42.4 Hz), 154.4, 126.7, 126.2, 124.4, 117.9 (q, $^1J_{CF}$ = 270.4 Hz), 103.4 (q, $^4J_{CF}$ = 2.0 Hz), 34.9, 31.1. $^{19}$F NMR ($CDCl_3$, 376.5 MHz): δ -65.4 (d, 3F, $J$ = 0.6 Hz).

HRMS (ESI-TOF): m/z $[M+H]^+$ Calcd for $C_{14}H_{15}F_3NO^+$: 270.1100; found: 270.1102.

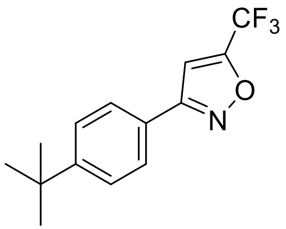


**5-(Trifluoromethyl)-3-(4-(trifluoromethyl)phenyl)isoxazole (3i)**. Obtained from **1i** (0.134 g, 0.504 mmol (A); 0.137 g, 0.515 mmol (B)). Colorless solid, m.p. 55-57 °C (Lit.: 63-64 °C), yield 0.067 g (47%, (A)); 0.070 g (48%, (B)). $^1$H NMR ($CDCl_3$, 400.1 MHz): δ 7.94 (d, 2H, $^3J$ = 8.2 Hz), 7.75 (d, 2H, $^3J$ = 8.2 Hz), 7.05 (*pseudo*-d, 1H, $^4J$ = 0.8 Hz). $^{13}$C{$^1$H} NMR ($CDCl_3$, 100.6 MHz): δ 161.5, 159.8 (q, $^2J_{CF}$ = 42.9 Hz), 132.8 (q, $^2J_{CF}$ = 32.8 Hz), 130.7, 127.3, 126.2 (q, $^3J_{CF}$ = 3.9 Hz), 123.6 (q, $^1J_{CF}$ = 272.4 Hz), 117.7 (q, $^1J_{CF}$ = 270.5 Hz), 103.5 (q, $^4J_{CF}$ = 2.0 Hz). $^{19}$F

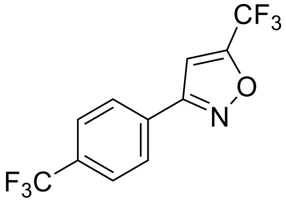

NMR ($CDCl_3$, 376.5 MHz): δ -64.2 (d, 3F), -65.3 (d, 3F, $J$ = 0.6 Hz). NMR data are in agreement with those in the literature[Error! Bookmark not defined.].

**3-(2,3-diHydrobenzo[*b*][1,4]dioxin-6-yl)-5-(trifluoromethyl)isoxazole (3k)**. Obtained from **1k** (0.128 g, 0.5 mmol (A); 0.126 g, 0.492 mmol (B)).

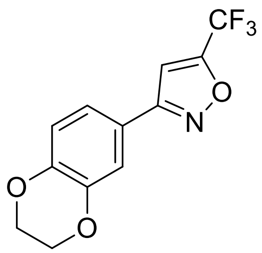


Colorless solid, m.p. 102-104 °C, yield 0.026 g (19%, (A)); 0.021 g (16%, (B)). $^1$H NMR ($CDCl_3$, 400.1 MHz): δ 7.34 (d, 1H, $^4J$ = 2.1 Hz), 7.30 (dd, 1H, $^3J$ = 8.4 Hz, $^4J$ = 2.1 Hz), 6.96 (d, 1H, $^3J$ = 8.4 Hz), 6.91 (*pseudo*-d, 1H, $^4J$ = 0.9 Hz), 4.34-4.29 m, 4H). $^{13}$C{$^1$H} NMR ($CDCl_3$, 100.6 MHz): δ 162.0, 158.9 (q, $^2J_{CF}$ = 42.4 Hz), 145.9, 144.0, 120.5, 120.3, 118.0, 117.9 (q, $^1J_{CF}$ = 270.8 Hz), 116.0, 103.3 (q, $^4J_{CF}$ = 2.0 Hz), 64.5, 64.3. $^{19}$F NMR ($CDCl_3$, 376.5 MHz): δ -65.4 (d, 3F, $^4J$ = 0.4 Hz).

HRMS (ESI-TOF): m/z $[M+H]^+$ Calcd for $C_{12}H_9F_3NO_3^+$: 272.0529; found: 272.0531.

**3-(2-Chlorophenyl)-5-(trifluoromethyl)isoxazole (3l)**. Obtained from **1l** (0.119 g, 0.512 mmol (A); 0.123 g, 0.529 mmol (B)). Pale yellow oil, yield 0.037

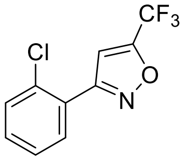


g (29%, (A)); 0.047 g (36%, (B)). $^1$H NMR ($CDCl_3$, 400.1 MHz): δ 7.75 (dd, 1H, $^3J$ = 7.7 Hz, $^4J$ = 1.8 Hz), 7.52 (dd, 1H, $^3J$ = 7.7 Hz, $^4J$ = 1.4 Hz), 7.45 (td, 1H, $^3J$ = 7.7 Hz, $^4J$ = 1.8 Hz), 7.39 (td, 1H, $^3J$ = 7.7 Hz, $^4J$ = 1.4 Hz), 7.19 (*pseudo*-d, 1H, $^4J$ = 0.8 Hz). $^{13}$C{$^1$H} NMR ($CDCl_3$, 100.6 MHz): δ 161.2, 158.5 (q, $^2J_{CF}$ = 42.6 Hz), 132.9, 131.7, 131.1, 130.6, 127.4, 126.5, 117.8 (q, $^1J_{CF}$ = 270.3 Hz), 106.6 (q, $^4J_{CF}$ = 2.0 Hz). $^{19}$F NMR ($CDCl_3$, 376.5 MHz): δ -65.2 (d, 3F, $^4J$ = 0.9 Hz).

HRMS (ESI-TOF): m/z $[M+H_3O]^+$ Calcd for $C_{10}H_8[^{35}Cl]F_3NO_2^+$: 266.0190; found: 266.0190; Calcd for $C_{10}H_8[^{37}Cl]F_3NO_2^+$: 268.0161; found: 268.0156.

**3-(2-Methoxyphenyl)-5-(trifluoromethyl)isoxazole (3m)**. Obtained from **1m** (0.113 g, 0.502 mmol (A); 0.108 g, 0.48 mmol (B)). Colorless oil, yield

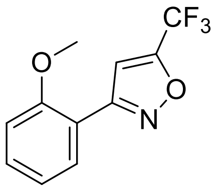


0.026 g (21%, (A)); 0.030 g (26%, (B)). $^1$H NMR ($CDCl_3$, 400.1 MHz): δ 7.91 (dd, 1H, $^3J$ = 7.7 Hz, $^4J$ = 1.7 Hz), 7.48-7.44 (m, 1H), 7.22 (s, 1H), 7.06 (*pseudo*-t, 1H, $^3J$ = 7.5 Hz), 7.02 (d, 1H, $^3J$ = 8.4 Hz), 3.92 (s, 1H). $^{13}$C{$^1$H} NMR ($CDCl_3$, 100.6 MHz): δ 160.1, 157.9 (q, $^2J_{CF}$ = 42.2 Hz), 157.2, 132.1, 129.4, 121.1, 118.1 (q, $^1J_{CF}$ = 270.0 Hz), 116.1, 111.4, 106.9 (q, $^4J_{CF}$ = 2.0 Hz), 55.6. $^{19}$F NMR ($CDCl_3$, 376.5 MHz): δ -65.3 (d, 3F, $^4J$ = 0.9 Hz).

HRMS (ESI-TOF): m/z $[M+H]^+$ Calcd for $C_{11}H_9F_3NO_2^+$: 244.0580; found: 244.0581.

**3-Octyl-5-(trifluoromethyl)isoxazole (3o)**. Obtained from **1o** (0.118 g, 0.504 mmol (A); 0.130 g, 0.556 mmol (B)). Colorless oil, yield 0.041 g (32%, (A)); 0.046 g (33%, (B)). $^1$H NMR ($CDCl_3$, 400.1 MHz): δ 6.53 (*pseudo*-d, 1H, $^4J$ = 0.7 Hz), 2.71 (t, 2H, $^3J$ = 7.7 Hz), 1.70-1.63 (m, 2H), 1.39-1.19 (m, 10H), 0.88-0.85 (m, 3H). $^{13}$C{$^1$H} NMR ($CDCl_3$, 100.6 MHz): δ 164.2, 158.4 (q, $^2J_{CF}$ = 42.4 Hz), 118.0 (q, $^1J_{CF}$ = 270.2 Hz), 104.9 (q, $^4J_{CF}$ = 1.9 Hz), 31.8, 29.14, 29.08, 29.04, 28.0, 25.8, 22.6, 14.1. $^{19}$F NMR ($CDCl_3$, 376.5 MHz): δ -65.4 (d, 3F, $^4J$ = 0.9 Hz).

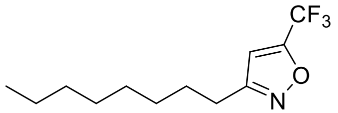


HRMS (ESI-TOF): m/z $[M+H]^+$ Calcd for $C_{12}H_{19}F_3NO^+$: 250.14133; found: 250.141.

**3-(Phenoxymethyl)-5-(trifluoromethyl)isoxazole (3p)**. Obtained from **1p** (0.117 g, 0.52 mmol (A); 0.125 g, 0.556 mmol (B)). Pale yellow oil, yield 0.065 g (51%, (A)); 0.064 g (47%, (B)). $^1$H NMR ($CDCl_3$, 400.1 MHz): δ 7.36-7.30 (m, 2H), 7.05-7.01 (m, 1H), 6.99-6.96 (m, 2H), 6.86 (*pseudo*-d, 1H, $^4J$ = 0.4 Hz), 5.20 (s, 2H). $^{13}$C{$^1$H} NMR ($CDCl_3$, 100.6 MHz): δ 161.2, 159.2 (q, $^2J_{CF}$ = 42.8 Hz), 157.6, 129.7, 122.0, 117.7 (q, $^1J_{CF}$ = 270.5 Hz), 114.6, 104.9 (q, $^4J_{CF}$ = 2.0 Hz), 61.0. $^{19}$F NMR ($CDCl_3$, 376.5 MHz): δ -65.2 (d, 3F, $^4J$ = 0.6 Hz).

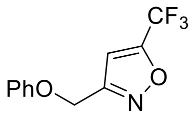


HRMS (ESI-TOF): m/z $[M+H]^+$ Calcd for $C_{11}H_9F_3NO_2^+$: 244.0580; found: 244.0585.

**Synthesis of amides 4-6 (general procedure).** A 4 mL vial with a screw cap was charged with 2,2,2-trifluoro-1-(5-phenyl-2*H*-1,2,3-triazol-4-yl)ethanone (**2a**). (0.054-0.059 g, 0.224-0.245 mmol) and corresponding amine (0.189-0.230 g, ~2.7 mmol, ~12 equiv.) The reaction mixture was heated at 90 °C for 2.5-8 h using magnetic stirrer with heating and then volatiles were evaporated in vacuo. The residue was passed through a short silica gel pad using $CH_2Cl_2$ followed by $CH_2Cl_2$ -MeOH (100:1) as eluents. Evaporation of volatiles afforded corresponding pure amide **4-6**.

**(5-Phenyl-2*H*-1,2,3-triazol-4-yl)(pyrrolidin-1-yl)methanone (4)**. Obtained from **2a** (0.054 g, 0.224 mmol) and pyrrolidine (0.189 g, 2.66 mmol) by heating for 2.5 h. Pale brown solid, m.p. 162-164 °C, yield 0.046 g (85%). $^1$H NMR ($CDCl_3$, 400.1 MHz): δ 7.76-7.65 (m, 2H), 7.35-7.25 (m, 3H), 3.67 (t, 2H, $^3J$ = 6.9 Hz), 3.38 (t, 2H, $^3J$ = 6.9 Hz), 1.92-1.85 (m, 2H), 1.85-1.74 (m, 2H). $^{13}$C{$^1$H} NMR ($CDCl_3$, 100.6 MHz): δ 162.5, 142.8, 138.0, 128.9, 128.6, 127.6, 48.6, 46.4, 25.9, 24.2.

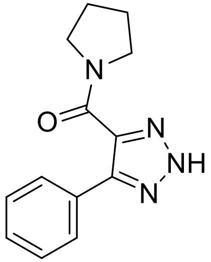


HRMS (ESI-TOF): m/z [M+Na]$^+$ Calcd for $C_{13}H_{14}N_4ONa^+$: 265.1060; found: 265.1068.

IR (ν, cm$^{-1}$): 1595 (C=O).

**(5-Phenyl-2*H*-1,2,3-triazol-4-yl)(piperidin-1-yl)methanone (5)**. Obtained from **2a** (0.053 g, 0.220 mmol) and piperidine (0.228 g, 2.68 mmol) by heating for 8 h. Pale brown solid, m.p. 123-125 °C, yield 0.033 g (58%). $^1$H NMR ($CDCl_3$, 400.1 MHz): δ 7.66 (dd, 2H, $^3J$ = 6.9 Hz, $^4J$ = 1.9 Hz), 7.41-7.38 (m, 3H), 3.82-3.62 (m, 2H), 3.28-3.10 (m, 2H), 1.68-1.48 (m, 4H), 1.34-1.21 (m, 2H). $^{13}$C{$^1$H} NMR ($CDCl_3$, 100.6 MHz): δ 163.0, 142.9, 137.4, 128.9, 128.8, 128.5, 127.3, 48.3, 43.3, 26.0, 25.4, 24.3.

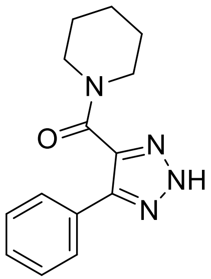


HRMS (ESI-TOF): m/z [M+H]$^+$ Calcd for $C_{14}H_{17}N_4O^+$: 257.1397; found: 257.1403.

IR (ν, cm$^{-1}$): 1592 (C=O).

**Morpholino(5-phenyl-2*H*-1,2,3-triazol-4-yl)methanone (6)**. Obtained from **2a** (0.053 g, 0.220 mmol) and morpholine (0.230 g, 2.64 mmol) by heating for 8 h. Pale brown viscous sticky mass, yield 0.027 g (48%). $^1$H NMR ($CDCl_3$, 400.1 MHz): δ 7.73-7.59 (m, 2H), 7.44-7.34 (m, 3H), 3.87-3.79 (m, 2H), 3.77-3.70 (m, 2H), 3.47-3.39 (m, 2H), 3.39-3.27 (m, 2H). $^{13}$C{$^1$H} NMR ($CDCl_3$, 100.6 MHz): δ 163.0, 141.6, 136.9, 129.3, 128.9, 127.5, 127.3, 66.53, 66.48, 47.5, 42.7.

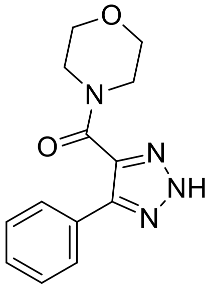

HRMS (ESI-TOF): m/z $[M+H]^+$ Calcd for $C_{13}H_{15}N_4O_2^+$: 259.1190; found: 259.1190.

IR ($\nu$, $cm^{-1}$): 1604 (C=O).

**Synthesis of triazoles 7-10 (general procedure).** A 4 mL vial with a screw cap was charged with 2,2,2-trifluoro-1-(5-phenyl-2*H*-1,2,3-triazol-4-yl)ethanone (**2a**) (0.120 g, 0.498 mmol), DMF (1 mL), $Na_2CO_3$ (0.079 g, 0.745 mmol, 1.5 equiv.), and corresponding alkylating reagent (0.548 mmol, ~1.1 equiv.) The reaction mixture was stirred overnight, poured into water (20 mL) and extracted with $CH_2Cl_2$ (3x20 mL). Combined organic phase was washed with water (20 mL), dried over $Na_2SO_4$ and then volatiles were evaporated in vacuo. The residue was purified by column chromatography on silica gel using appropriate mixtures of hexane and $CH_2Cl_2$ as eluents.

**1-(2-Benzyl-5-phenyl-2*H*-1,2,3-triazol-4-yl)-2,2,2-trifluoroethanone (7)**. Obtained from **2a** (0.120 g, 0.498 mmol) and benzylbromide (0.094 g, 0.550 mmol). Purified using gradient eluating by hexane-$CH_2Cl_2$ (3:1) followed by hexane-$CH_2Cl_2$ (1:1) and $CH_2Cl_2$. Beige oil, yield 0.113 g (69%). $^1$H NMR ($CDCl_3$, 400.1 MHz): δ 7.96-7.86 (m, 2H), 7.53-7.44 (m, 5H), 7.44-7.36(m, 3H), 5.71 (s, 2H). $^{13}$C{$^1$H} NMR ($CDCl_3$, 100.6 MHz): δ 174.2 (q, $^2J_{CF}$ = 37.0 Hz), 152.7, 136.4, 133.5, 130.1, 129.1, 129.0, 128.9, 128.40, 128.35, 128.2, 116.3 (q, $^1J_{CF}$ = 290.7 Hz), 59.9. $^{19}$F NMR ($CDCl_3$, 376.5 MHz): δ -74.9 (s, 3F).

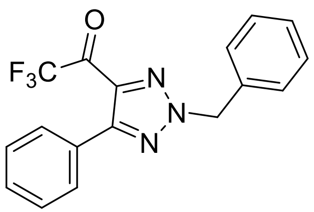


HRMS (ESI-TOF): m/z [M+H]$^+$ Calcd for $C_{17}H_{13}F_3N_3O^+$: 332.1005; found: 332.1005.

IR (ν, cm$^{-1}$): 1723 (C=O).

**1-(1-Benzyl-5-phenyl-1*H*-1,2,3-triazol-4-yl)-2,2,2-trifluoroethanone (8)**. Obtained from **2a** as an admixture (81:19) in the synthesis of 7. Beige thick oil, yield 0.026 g (15%). $^1$H NMR ($CDCl_3$, 400.1 MHz): δ 7.58-7.52 (m, 1H), 7.51-7.46 (m, 2H), 7.31-7.25 (m, 3H), 7.24-7.20 (m, 2H), 7.08-7.00 (m, 2H), 5.46 (s, 2H). $^{13}$C{$^1$H} NMR ($CDCl_3$, 100.6 MHz): δ 174.2 (q, $^2J_{CF}$ = 37.3 Hz), 143.9, 138.1, 133.9, 130.9, 129.4, 129.0, 128.9, 128.7, 127.7, 124.4, 116.1 (q, $^1J_{CF}$ = 290.6 Hz), 52.2. $^{19}$F NMR ($CDCl_3$, 376.5 MHz): δ -75.4 (s, 3F). NMR data are in agreement with those in the literature

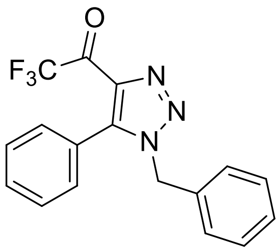


**1-(2-(2,4-Dinitrophenyl)-5-phenyl-2*H*-1,2,3-triazol-4-yl)-2,2,2-trifluoroethanone (9)**. Obtained from **2a** (0.057 g, 0.237 mmol) and 1-fluoro-2,4-dinitrobenzene (0.049 g, 0.263 mmol). Purified using gradient eluating by hexane-$CH_2Cl_2$ (3:1) followed by hexane-$CH_2Cl_2$ (1:1).

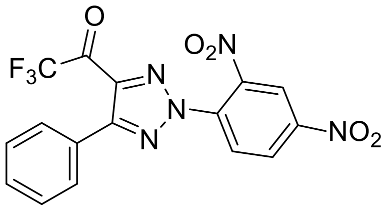

Pale yellow powder, m.p. 132-134 °C, yield 0.079 g (82%). $^1$H NMR (CDCl$_3$, 400.1 MHz): δ 8.67 (d, 1H, $^4J$ = 2.4 Hz), 8.56 (dd, 1H, $^3J$ = 8.9 Hz, $^4J$ = 2.4 Hz), 8.31 (d, 1H, $^3J$ = 8.9 Hz), 7.83 (dd, 2H, $^3J$ = 7.7 Hz, $^4J$ = 1.7 Hz), 7.49-7.40 (m, 3H). $^{13}$C{$^1$H} NMR (CDCl$_3$, 100.6 MHz): δ 173.7 (q, $^2J_{CF}$ = 38.2 Hz), 153.8, 147.1, 142.8, 138.7, 134.1, 130.8, 129.0, 128.4, 127.4, 126.4, 120.7, 115.6 (q, $^1J_{CF}$ = 290.5 Hz). $^{19}$F NMR (CDCl$_3$, 376.5 MHz): δ -75.0 (s, 3F).

HRMS (ESI-TOF): m/z [M+H]$^+$ Calcd for $C_{16}H_9F_3N_5O_5^+$: 408.0550; found: 408.0549.

IR (ν, cm$^{-1}$): 1738 (C=O); 1545, 1540, 1349, 1335 ($NO_2$).

**2,2,2-Trifluoro-1-(5-phenyl-2-tosyl-2*H*-1,2,3-triazol-4-yl)ethanone (10)**. Obtained from **2a** (0.060 g, 0.249 mmol) and 4-toluenesulfonyl chloride (0.052 g, 0.274 mmol). Purified using gradient eluating by hexane-$CH_2Cl_2$ (1:1) followed by $CH_2Cl_2$.White crystals, m.p. 156-160 °C, yield 0.073 g (74%). $^1$H NMR (CDCl$_3$, 400.1 MHz): δ 8.08 (d, 2H, $^3J$ = 8.4 Hz), 7.83 (dd, 2H, $^3J$ = 7.9 Hz, $^4J$ = 1.4 Hz) 7.52-7.39 (m, 5H), 2.46 (s, 3H). $^{13}$C{$^1$H} NMR (CDCl$_3$, 100.6 MHz): δ 174.5 (q, $^2J_{CF}$ = 37.9 Hz), 153.4, 148.1, 138.6, 131.6, 130.8, 130.6, 129.6, 129.3, 128.5, 126.9, 115.8 (q, $^1J_{CF}$ = 290.5 Hz), 21.9. $^{19}$F NMR (CDCl$_3$, 376.5 MHz): δ -75.2 (s, 3F).

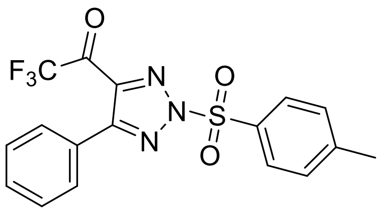


HRMS (ESI-TOF): m/z [M+H]$^+$ Calcd for $C_{17}H_{13}F_3N_3O_3S^+$: 396.0324; found: 396.0626.

IR (ν, cm$^{-1}$): 1604 (C=O).

**Synthesis of 1-(2,5-diphenyl-2*H*-1,2,3-triazol-4-yl)-2,2,2-trifluoroethanone (11)**. A 25 mL round-bottomed flask was charged with 2,2,2-trifluoro-1-(5-

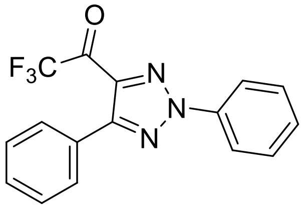

phenyl-2*H*-1,2,3-triazol-4-yl)ethanone (**2a**) (0.062 g, 0.257 mmol), DMSO (1.5 mL), $PhB(OH)_2$ (0.047 g, 0.385 mmol, 1.5 equiv.), and $Cu(OAc)_2xH_2O$ (0.0051, 0.257 mmol, 0.1 equiv.) The reaction mixture was heated at 100 °C for 5 h at air using magnetic stirrer with heating. The reaction mixture was poured into water (20 mL) and extracted with $CH_2Cl_2$ (3x20 mL). Combined organic phase was washed with water (20 mL), dried over $Na_2SO_4$ and then volatiles were evaporated in vacuo. The residue was purified by column chromatography on silica gel using using gradient eluating by hexane-$CH_2Cl_2$ (3:1) followed by hexane-$CH_2Cl_2$ (1:1) as eluents. Colorless crystals, m.p. 93-95 **°**C yield 0.032 g (50%). $^1$H NMR ($CDCl_3$, 400.1 MHz): δ 8.28-8.18 (m, 2H), 8.03-7.95 (m, 2H), 7.59-7.46 (m, 6H). $^{13}$C{$^1$H} NMR ($CDCl_3$, 100.6 MHz): δ 174.4 (q, $^2J_{CF}$ = 37.5 Hz), 152.9, 138.8, 137.1, 130.4, 129.6, 129.4, 129.2, 128.5, 128.1, 119.7, 116.3 (q, $^1J_{CF}$ = 290.9 Hz). $^{19}$F NMR ($CDCl_3$, 376.5 MHz): δ -74.9 (s, 3F).

HRMS (ESI-TOF): m/z $[M+H]^+$ Calcd for $C_{16}H_{11}F_3N_3O^+$: 318.0849; found: 318.0851.

IR (ν, cm$^{-1}$): 1716 (C=O).

13 May 2022

| | | | | | | | |
|---|---|---|---|---|---|---|---|
| ***Acquisition Time (sec)*** | 4.0894 | ***Comment*** | Imported from UXNMR. | | | ***Date*** | 25 Apr 2022 15:23:00 |
| ***File Name*** | C:\DOCS\OUTPUT_301\2022\04.图疱朦\BM-2508-3.H_001001r | | | | | ***Frequency (MHz)*** | 400.13 |
| ***Nucleus*** | 1H | ***Number of Transients*** | 4 | ***Original Points Count*** | 32768 | ***Points Count*** | 131072 |
| ***Pulse Sequence*** | zg30 | ***Solvent*** | CHLOROFORM-D | | | ***Sweep Width (Hz)*** | 8012.82 |
| ***Temperature (degree C)*** | 27.000 | | | | | | |

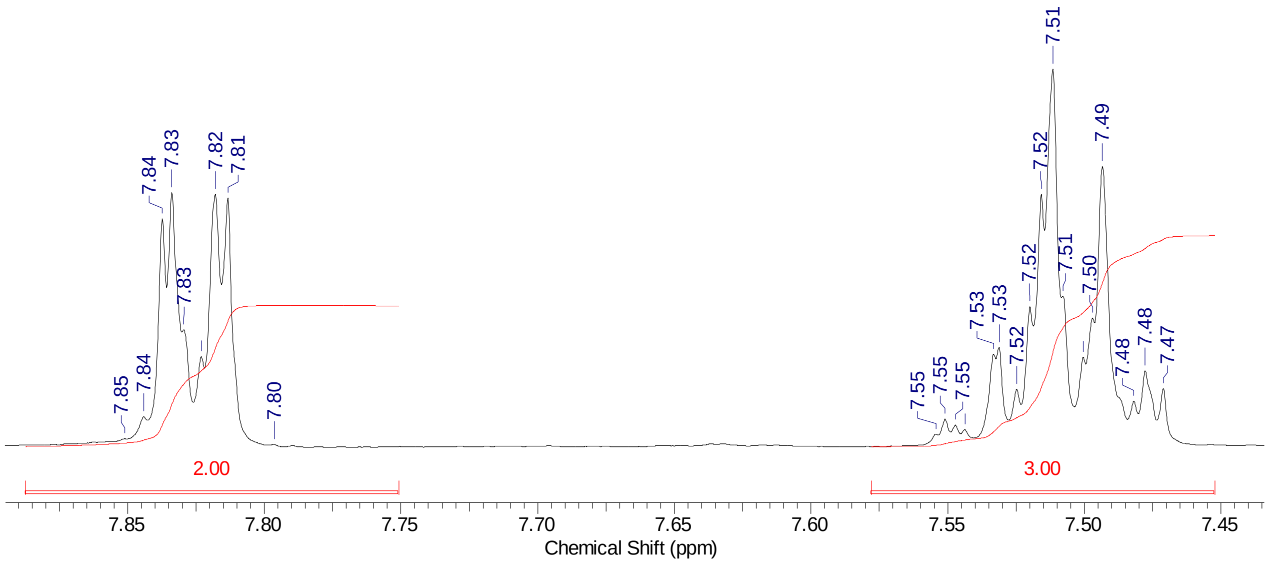


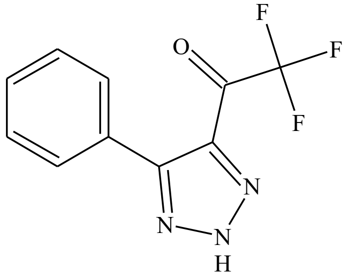

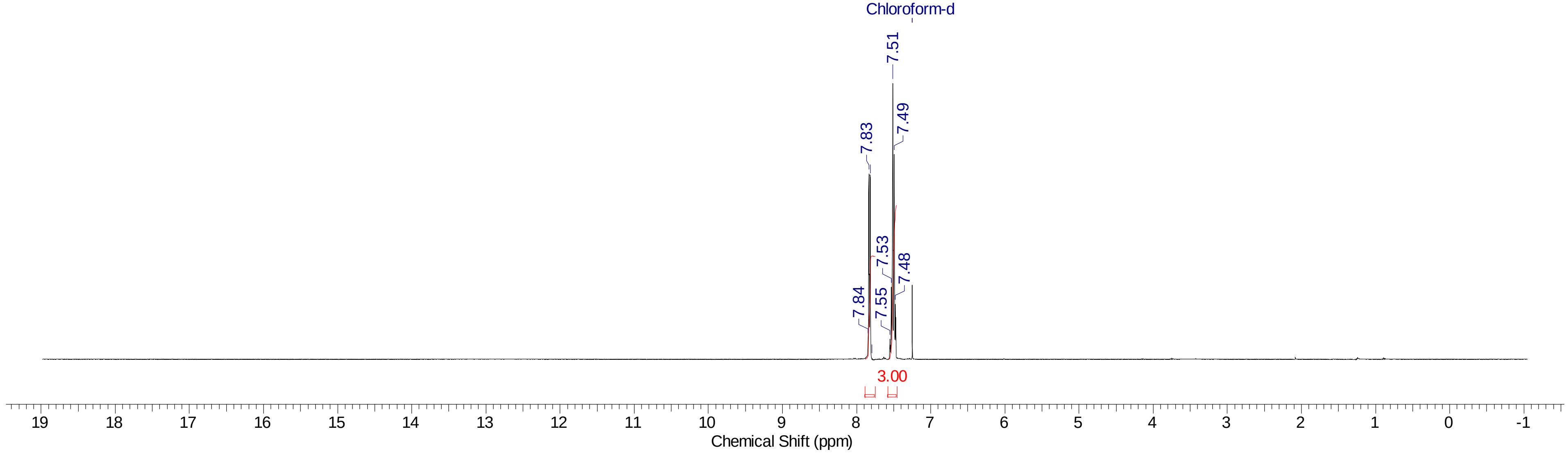

$^{1}H$ NMR spectrum of **2a** (400.1 MHz, $CDCl_3$)

13 May 2022

| | | | | | | | |
|---|---|---|---|---|---|---|---|
| ***Acquisition Time (sec)*** | 1.7433 | ***Comment*** | Imported from UXNMR. | | | ***Date*** | 25 Apr 2022 15:31:28 |
| ***File Name*** | C:\DOCS\OUTPUT_301\2022\04.図疱朦\BM-2508-3.F_005001r | | | | | ***Frequency (MHz)*** | 376.50 |
| ***Nucleus*** | 19F | ***Number of Transients*** | 16 | ***Original Points Count*** | 131072 | ***Points Count*** | 262144 |
| ***Pulse Sequence*** | zgflqn | ***Solvent*** | CHLOROFORM-D | | | ***Sweep Width (Hz)*** | 75187.97 |
| ***Temperature (degree C)*** | 27.000 | | | | | | |

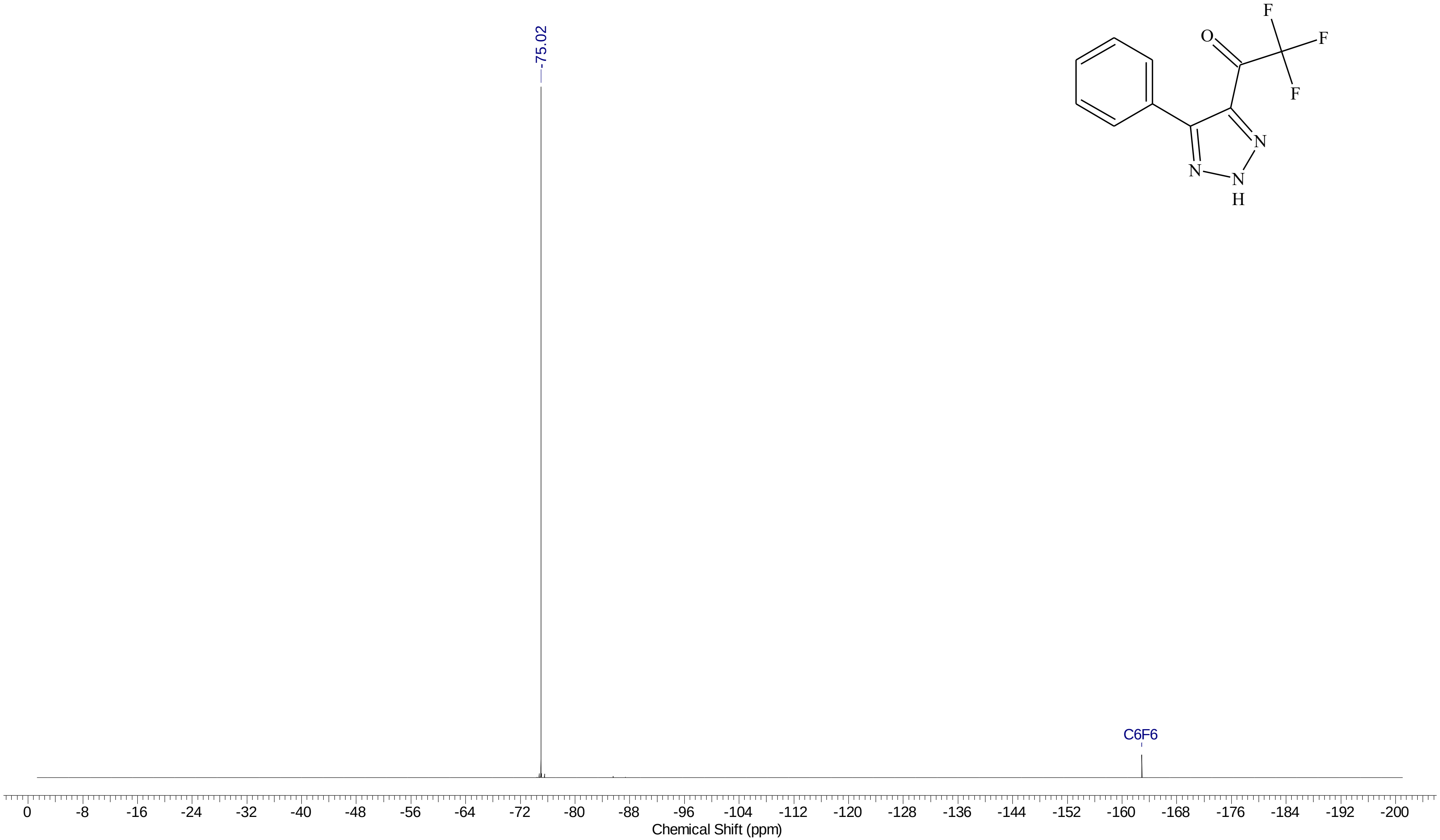

$^{19}$F NMR spectrum of **2a** (376.5 MHz, $CDCl_3$)

16 May 2022

| | | | | | | | |
|---|---|---|---|---|---|---|---|
| ***Acquisition Time (sec)*** | 0.6783 | ***Comment*** | Imported from UXNMR. | | | ***Date*** | 26 Apr 2022 14:36:18 |
| ***File Name*** | C:\DOCS\OUTPUT_301\2022\04.图疱朦\BM-2508-3.C_002001r | | | | | ***Frequency (MHz)*** | 100.61 |
| ***Nucleus*** | 13C | ***Number of Transients*** | 217 | ***Original Points Count*** | 16384 | ***Points Count*** | 131072 |
| ***Pulse Sequence*** | zgpg30 | ***Solvent*** | CHLOROFORM-D | | | ***Sweep Width (Hz)*** | 24154.59 |
| ***Temperature (degree C)*** | 27.000 | | | | | | |

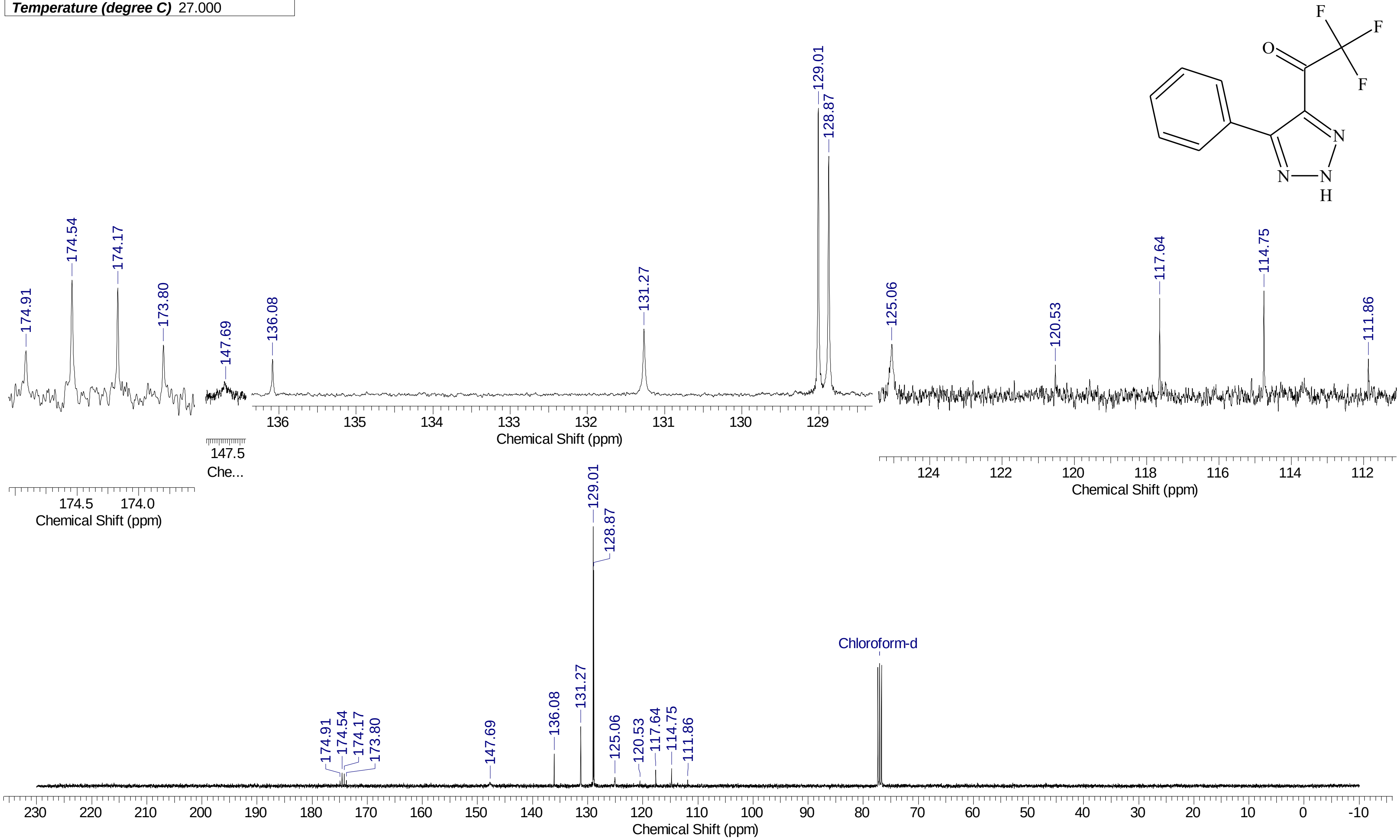

## $^{13}$C NMR spectrum of **2a** (100.6 MHz, $CDCl_3$)

13 May 2022

| | | | | | | | |
|---|---|---|---|---|---|---|---|
| ***Acquisition Time (sec)*** | 4.0894 | ***Comment*** | Imported from UXNMR. | | | ***Date*** | 04 Feb 2022 15:56:08 |
| ***File Name*** | C:\DOCS\OUTPUT_301\2022\02.翦 怵嚯黒SZA-BM-2368-15.H_001001r | | | | | ***Frequency (MHz)*** | 400.13 |
| ***Nucleus*** | 1H | ***Number of Transients*** | 4 | ***Original Points Count*** | 32768 | ***Points Count*** | 131072 |
| ***Pulse Sequence*** | zg30 | ***Solvent*** | ACETONITRILE-D3 | | | ***Sweep Width (Hz)*** | 8012.82 |
| ***Temperature (degree C)*** | 27.000 | | | | | | |

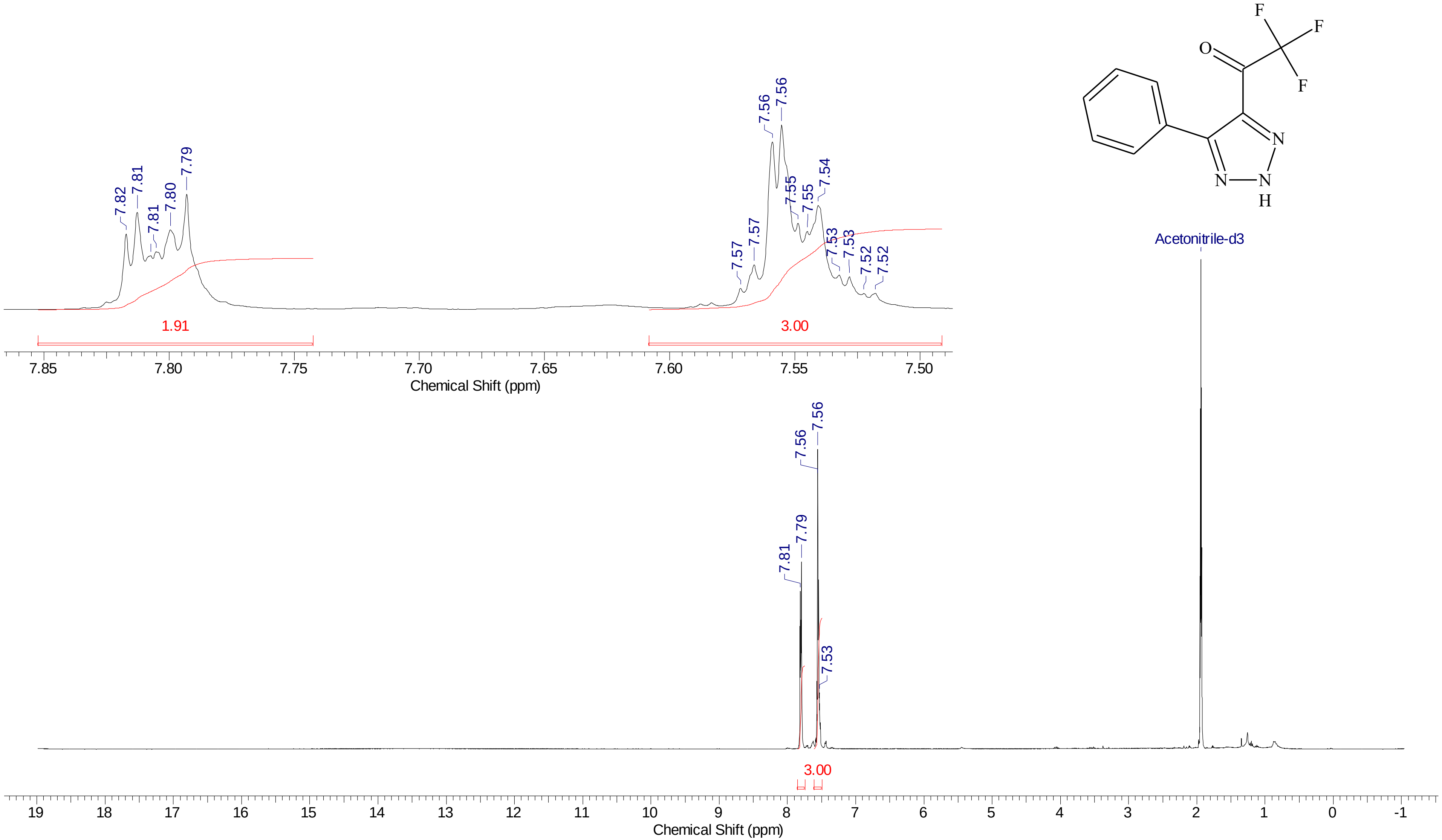

$^1$H NMR spectrum of **2a** (400.1 MHz, $CD_3CN$)

13 May 2022

| | | | | | |
|---|---|---|---|---|---|
| ***Acquisition Time (sec)*** | 1.7433 | ***Comment*** | Imported from UXNMR. | ***Date*** | 07 Feb 2022 15:16:48 |
| ***File Name*** | C:\DOCS\OUTPUT_301\2022\02.翦 怵嚯黑SZA-BM-2368-11.F_005001r | | | ***Frequency (MHz)*** | 376.50 |
| ***Nucleus*** | 19F | ***Number of Transients*** | 16 ***Original Points Count*** 131072 | ***Points Count*** | 262144 |
| ***Pulse Sequence*** | zgflqn | ***Solvent*** | CHLOROFORM-D | ***Sweep Width (Hz)*** | 75187.97 |
| ***Temperature (degree C)*** | 27.000 | | | | |

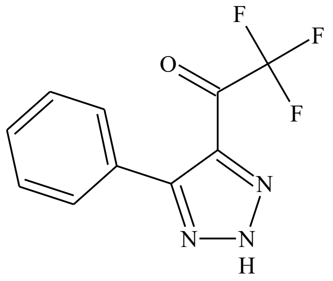


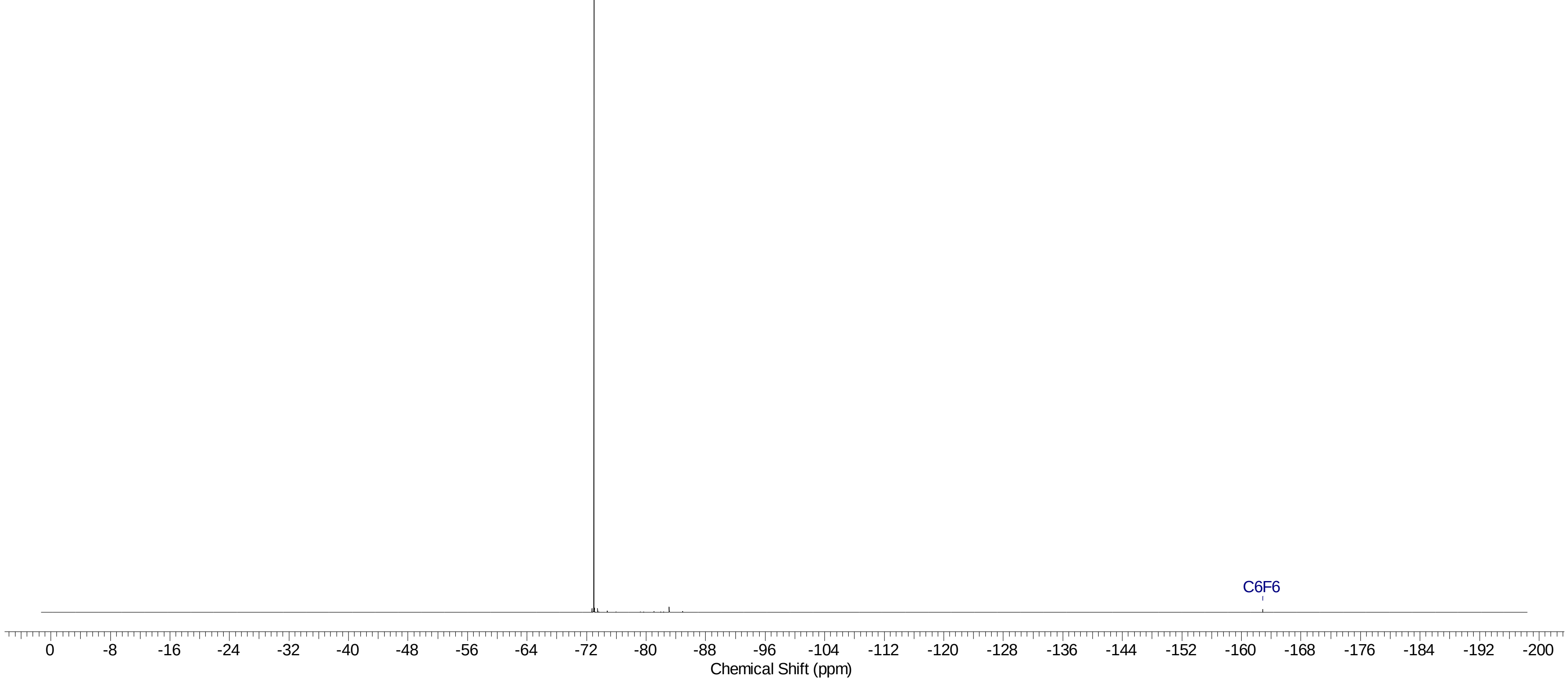

# $^{19}$F NMR spectrum of **2a** (376.5 MHz, $CD_3CN$)

13 May 2022

| | | | | | | | |
|---|---|---|---|---|---|---|---|
| ***Acquisition Time (sec)*** | 0.6783 | ***Comment*** | Imported from UXNMR. | | | ***Date*** | 08 Feb 2022 16:02:02 |
| ***File Name*** | C:\DOCS\OUTPUT_301\2022\02.翦 怺嚾黒SZA-BM-2368-11.C_002001r | | | | | ***Frequency (MHz)*** | 100.61 |
| ***Nucleus*** | 13C | ***Number of Transients*** | 248 | ***Original Points Count*** | 16384 | ***Points Count*** | 131072 |
| ***Pulse Sequence*** | zgpg30 | ***Solvent*** | ACETONITRILE-D3 | | | ***Sweep Width (Hz)*** | 24154.59 |
| ***Temperature (degree C)*** | 27.000 | | | | | | |

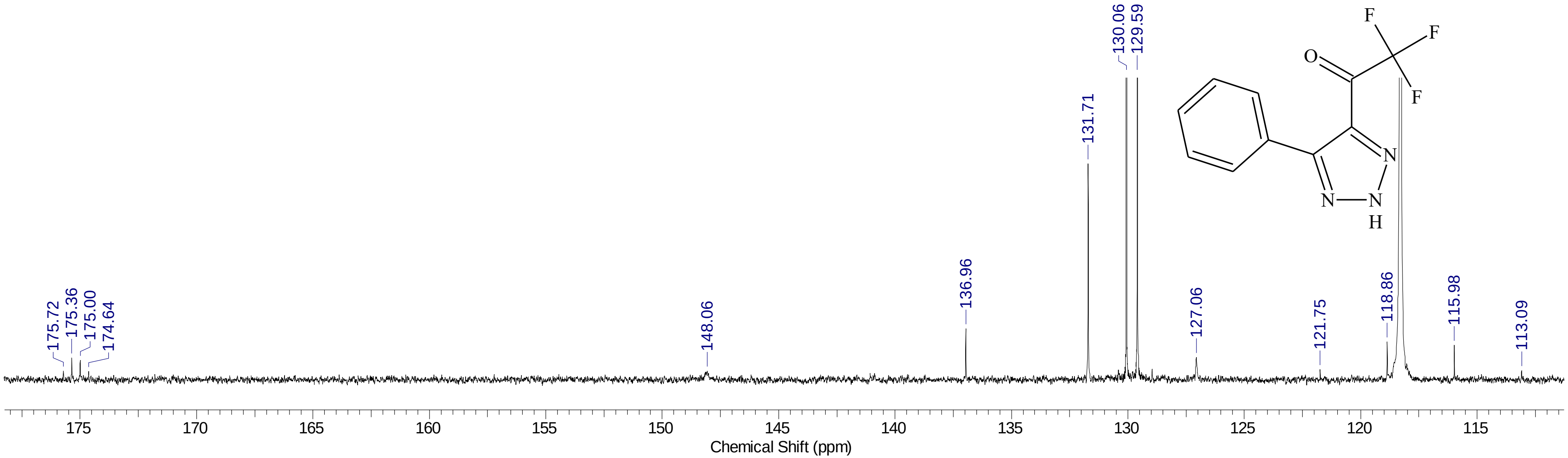


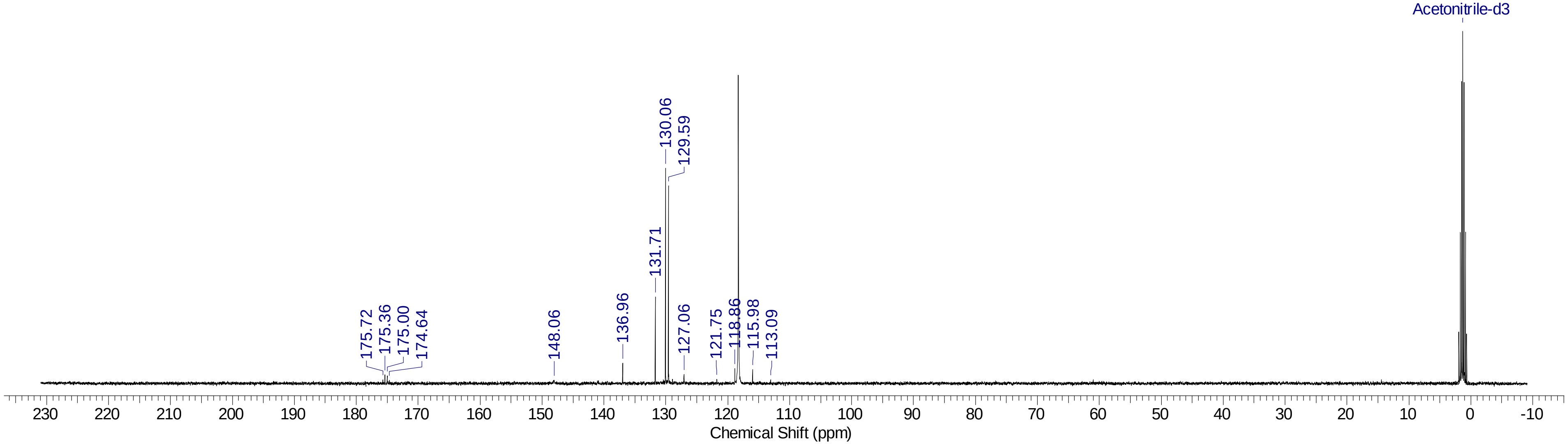

$^{13}C$ NMR spectrum of **2a** (100.6 MHz, $CD_3CN$)

20 May 2022

| | | | | | | | |
|---|---|---|---|---|---|---|---|
| ***Acquisition Time (sec)*** | 4.0894 | ***Comment*** | Imported from UXNMR. | | | ***Date*** | 25 Jan 2022 15:01:30 |
| ***File Name*** | C:\DOCS\OUTPUT_301\2022\01. 礅囵黒BM-2368-C-2.H_001001r | | | | | ***Frequency (MHz)*** | 400.13 |
| ***Nucleus*** | 1H | ***Number of Transients*** | 4 | ***Original Points Count*** | 32768 | ***Points Count*** | 131072 |
| ***Pulse Sequence*** | zg30 | ***Solvent*** | CHLOROFORM-D | | | ***Sweep Width (Hz)*** | 8012.82 |
| ***Temperature (degree C)*** | 27.000 | | | | | | |

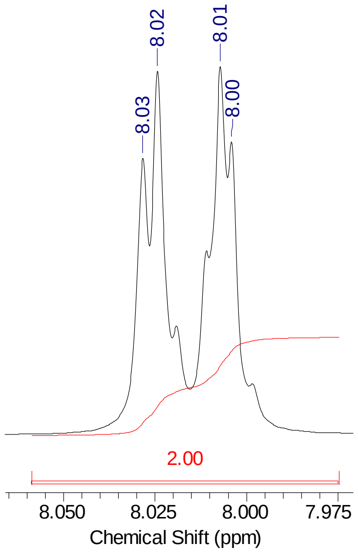


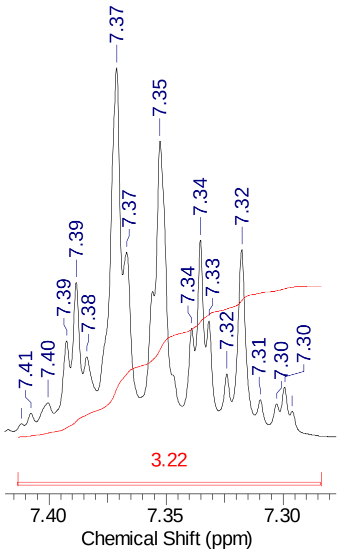


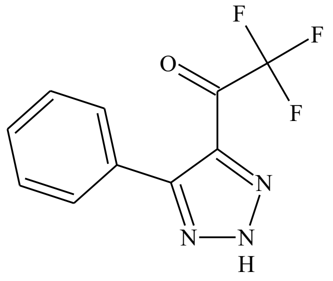


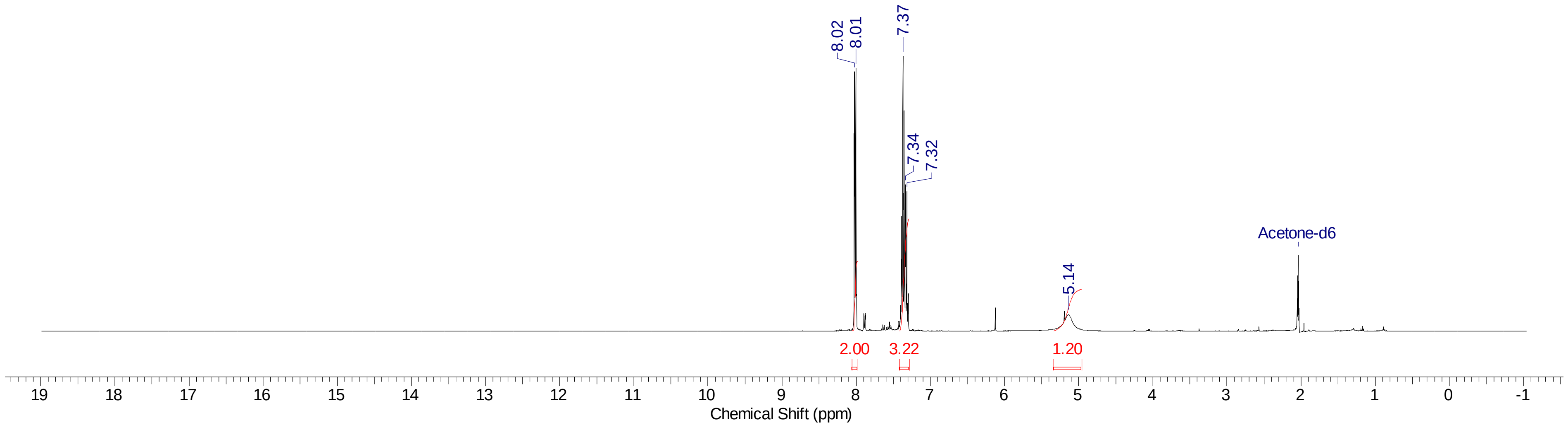

## $^{1}$H NMR spectrum of **2a** (400.1 MHz, acetone-$d_6$)

20 May 2022

| | | | | | | | |
|---|---|---|---|---|---|---|---|
| ***Acquisition Time (sec)*** | 1.7433 | ***Comment*** | Imported from UXNMR. | | | ***Date*** | 18 Jan 2022 15:24:18 |
| ***File Name*** | C:\DOCS\OUTPUT_301\2022\01. 礅囹黒BM-2368-2.F_005001r | | | | | ***Frequency (MHz)*** | 376.50 |
| ***Nucleus*** | 19F | ***Number of Transients*** | 16 | ***Original Points Count*** | 131072 | ***Points Count*** | 262144 |
| ***Pulse Sequence*** | zgflqn | ***Solvent*** | Acetone | ***Sweep Width (Hz)*** | 75187.97 | ***Temperature (degree C)*** | 27.000 |

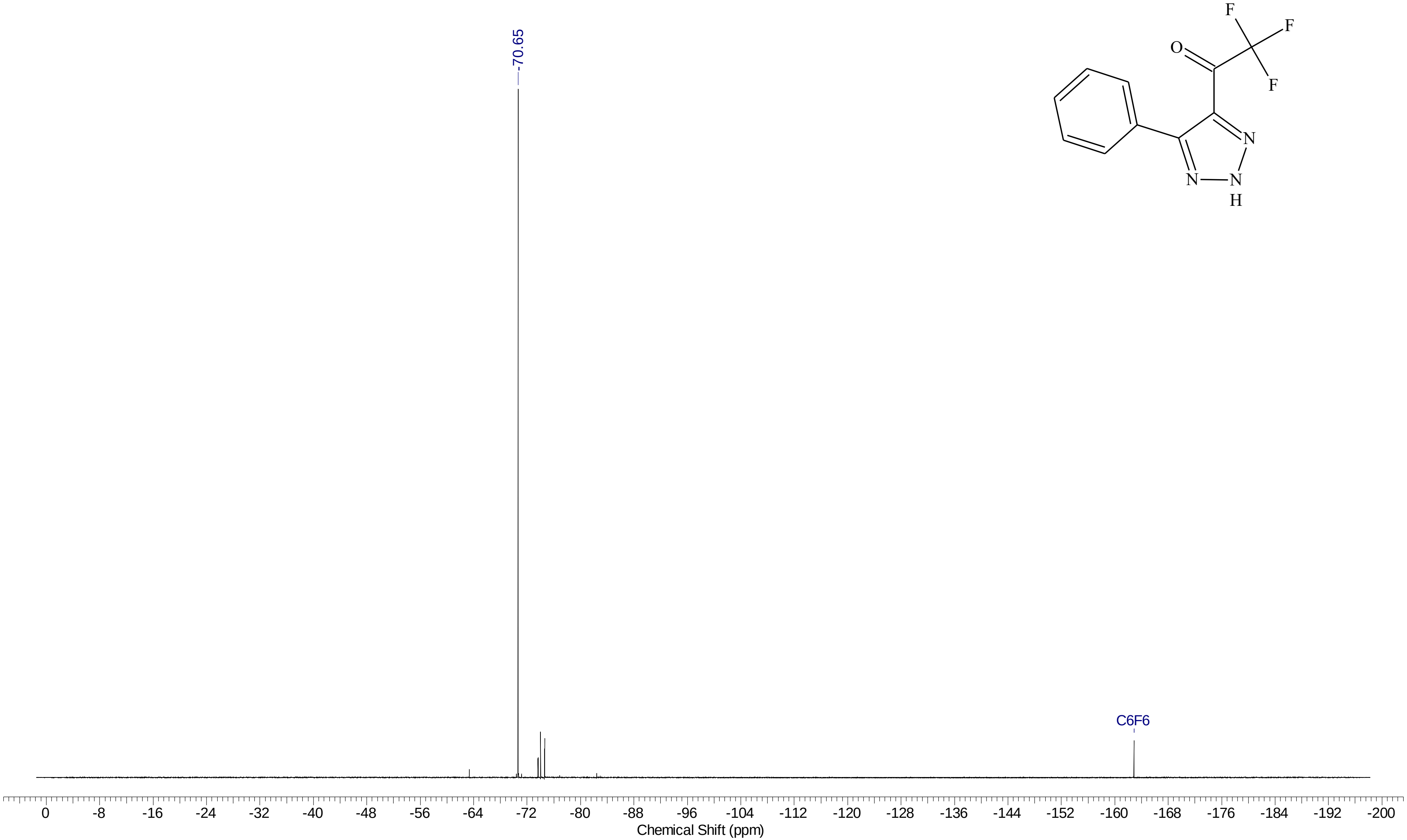

## $^{19}$F NMR spectrum of **2a** (376.5 MHz, acetone-$d_6$)

20 May 2022

| | | | | | |
|---|---|---|---|---|---|
| ***Acquisition Time (sec)*** | 0.6783 | ***Comment*** | Imported from UXNMR. | ***Date*** | 25 Jan 2022 15:05:38 |
| ***File Name*** | C:\DOCS\OUTPUT_301\2022\01. 礅囧黒BM-2368_002001r | ***Frequency (MHz)*** | 100.61 | ***Nucleus*** | 13C |
| ***Number of Transients*** | 57 | ***Original Points Count*** 16384 | ***Points Count*** 131072 | ***Pulse Sequence*** | zgpg30 |
| ***Solvent*** | CHLOROFORM-D | ***Sweep Width (Hz)*** | 24154.59 | ***Temperature (degree C)*** | 27.000 |

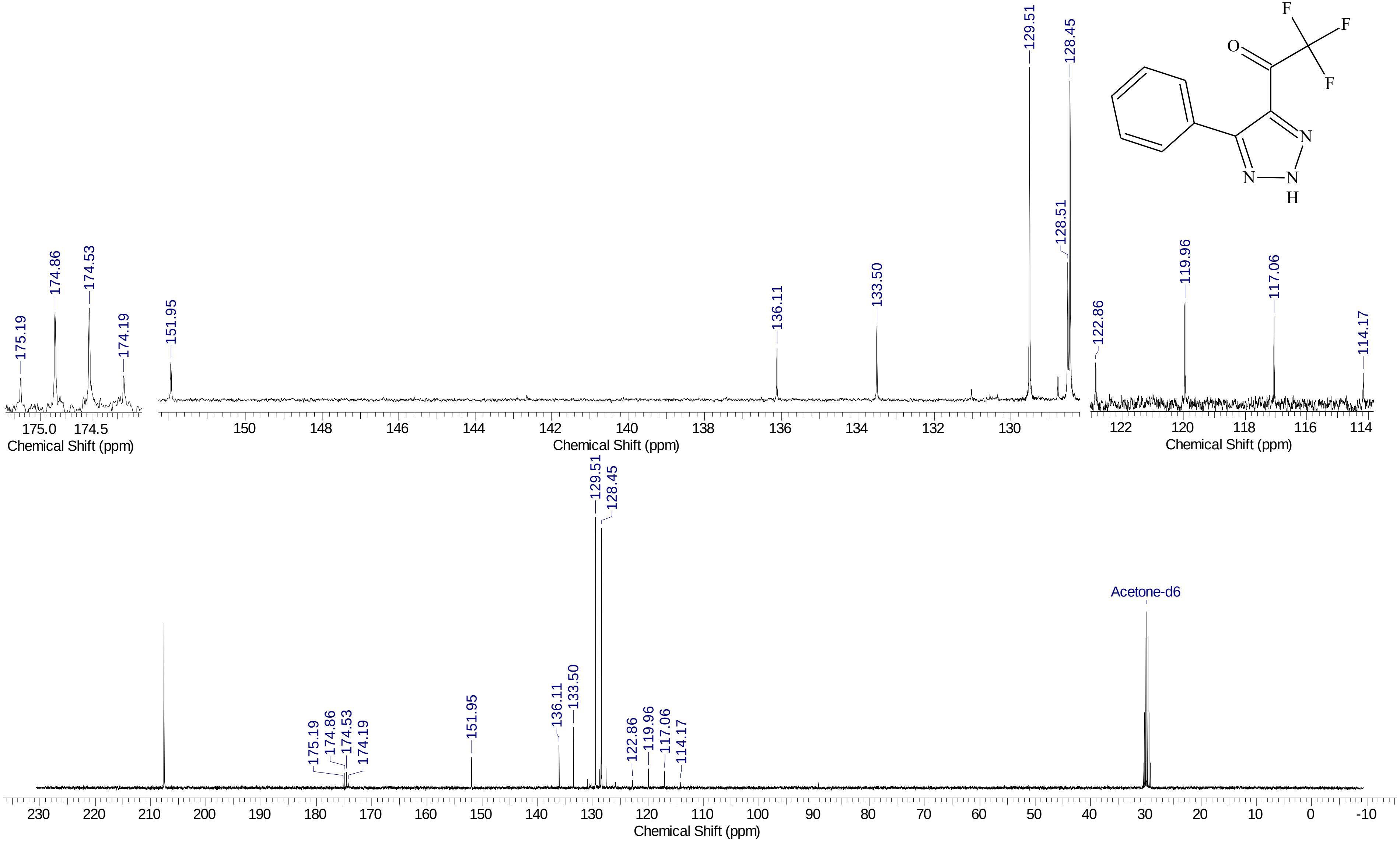

# $^{13}$C NMR spectrum of **2a** (100.6 MHz, acetone-d$_6$)

1 Jun 2022

| | | | | | | | |
|---|---|---|---|---|---|---|---|
| ***Acquisition Time (sec)*** | 4.0894 | ***Comment*** | Imported from UXNMR. | | | ***Date*** | 27 May 2022 15:23:34 |
| ***File Name*** | C:\DOCS\OUTPUT_301\2022\05.爨 閈BM-2511-2.H_001001r | | | ***Frequency (MHz)*** | 400.13 | ***Nucleus*** | 1H |
| ***Number of Transients*** | 4 | ***Original Points Count*** | 32768 | ***Points Count*** | 131072 | ***Pulse Sequence*** | zg30 |
| ***Solvent*** | CHLOROFORM-D | | | ***Sweep Width (Hz)*** | 8012.82 | ***Temperature (degree C)*** | 27.000 |

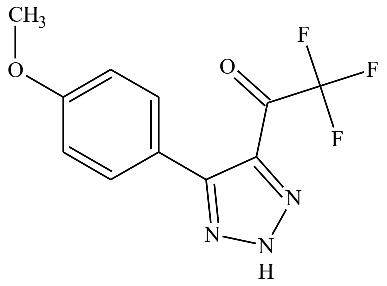


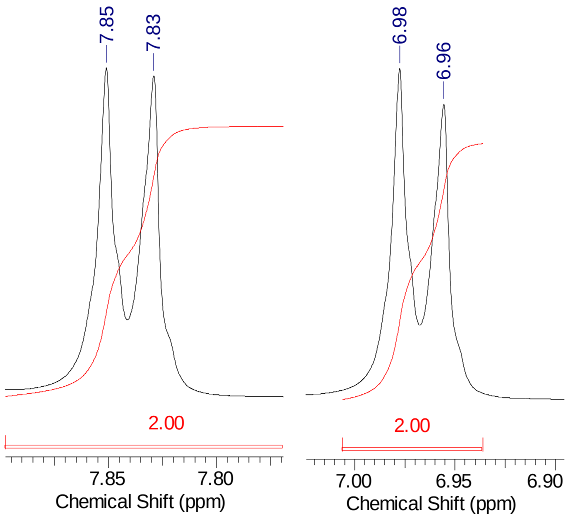


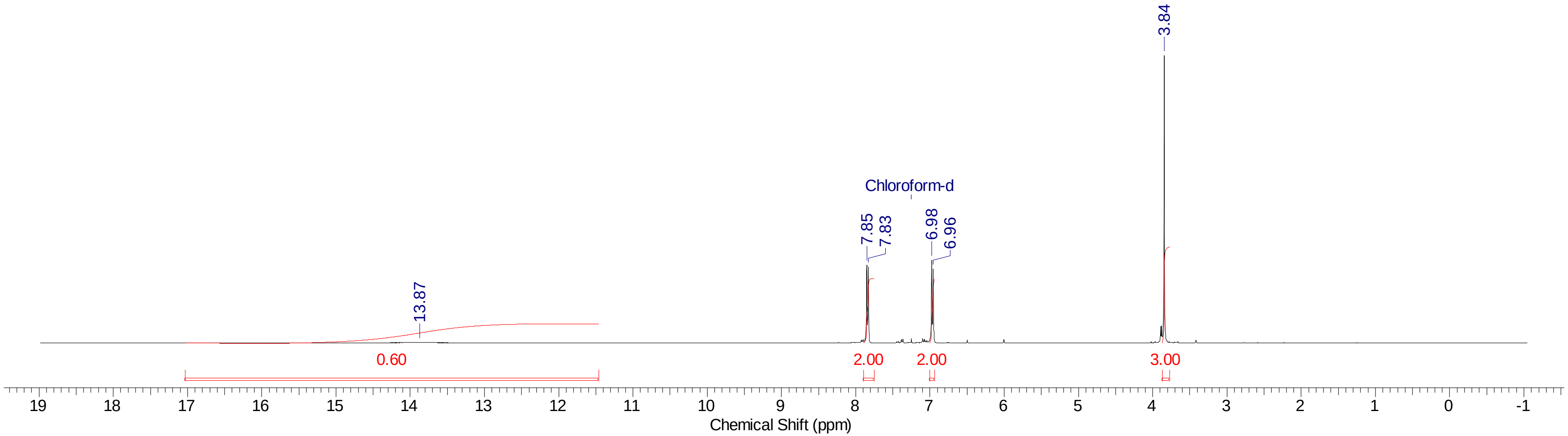

## $^{1}H$ NMR spectrum of **2b** (400.1 MHz, $CDCl_3$)

1 Jun 2022

| | | | | | | | |
|---|---|---|---|---|---|---|---|
| ***Acquisition Time (sec)*** | 1.7433 | ***Comment*** | Imported from UXNMR. | | | ***Date*** | 27 May 2022 15:38:18 |
| ***File Name*** | C:\DOCS\OUTPUT_301\2022\05.爨 閈BM-2511-2.F_005001r | | | ***Frequency (MHz)*** | 376.50 | ***Nucleus*** | 19F |
| ***Number of Transients*** | 16 | ***Original Points Count*** | 131072 | ***Points Count*** | 262144 | ***Pulse Sequence*** | zgflqn |
| ***Solvent*** | CHLOROFORM-D | | | ***Sweep Width (Hz)*** | 75187.97 | ***Temperature (degree C)*** | 27.000 |

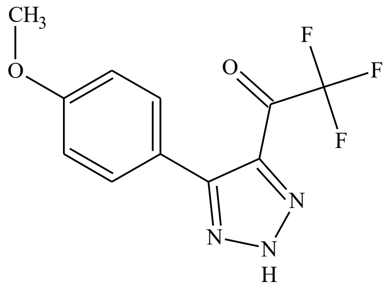


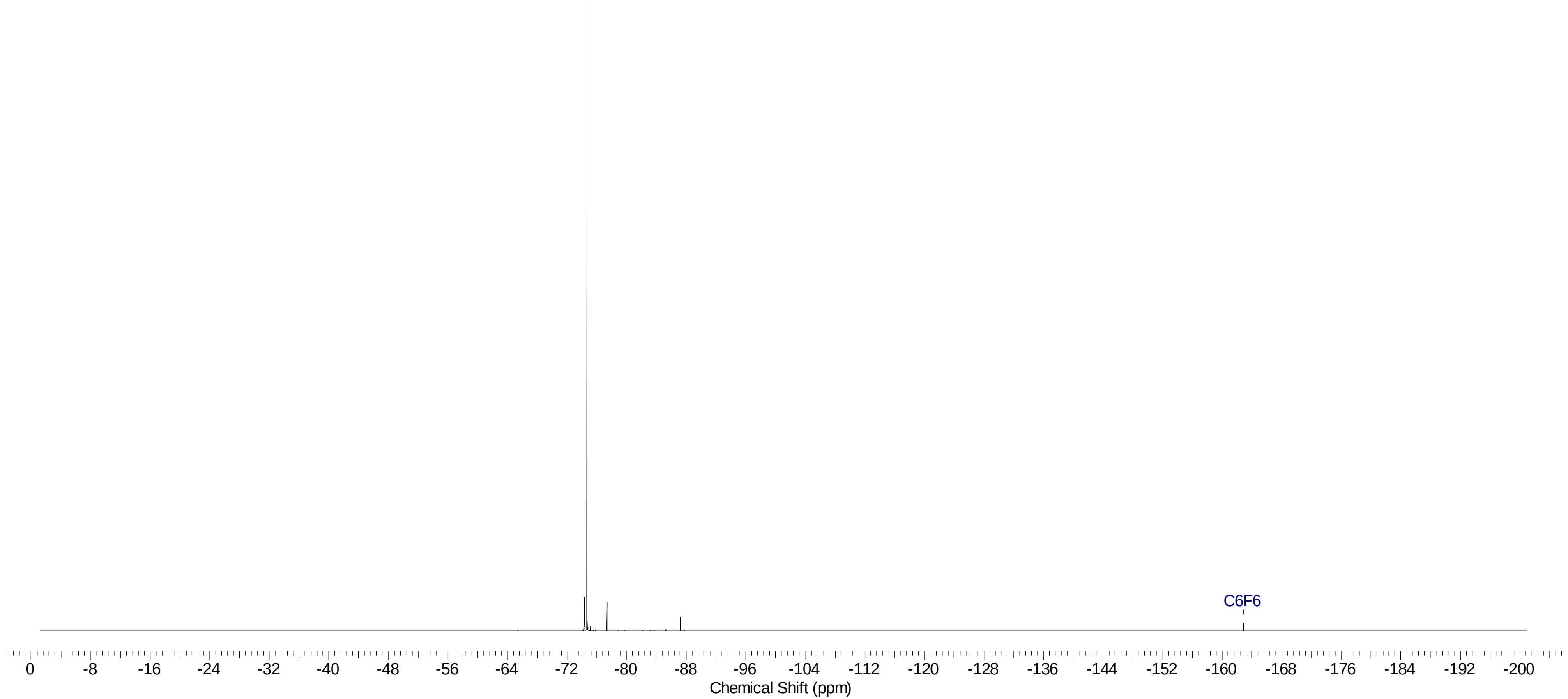

## $^{19}$F NMR spectrum of **2b** (376.5 MHz, $CDCl_3$)

1 Jun 2022

| | | | | | | | |
|---|---|---|---|---|---|---|---|
| ***Acquisition Time (sec)*** | 0.6783 | ***Comment*** | Imported from UXNMR. | | | ***Date*** | 27 May 2022 15:33:52 |
| ***File Name*** | C:\DOCS\OUTPUT_301\2022\05.爨 閈BM-2511-2.C_002001r | | | ***Frequency (MHz)*** | 100.61 | ***Nucleus*** | 13C |
| ***Number of Transients*** | 209 | ***Original Points Count*** | 16384 | ***Points Count*** | 131072 | ***Pulse Sequence*** | zgpg30 |
| ***Solvent*** | CHLOROFORM-D | | | ***Sweep Width (Hz)*** | 24154.59 | ***Temperature (degree C)*** | 27.000 |

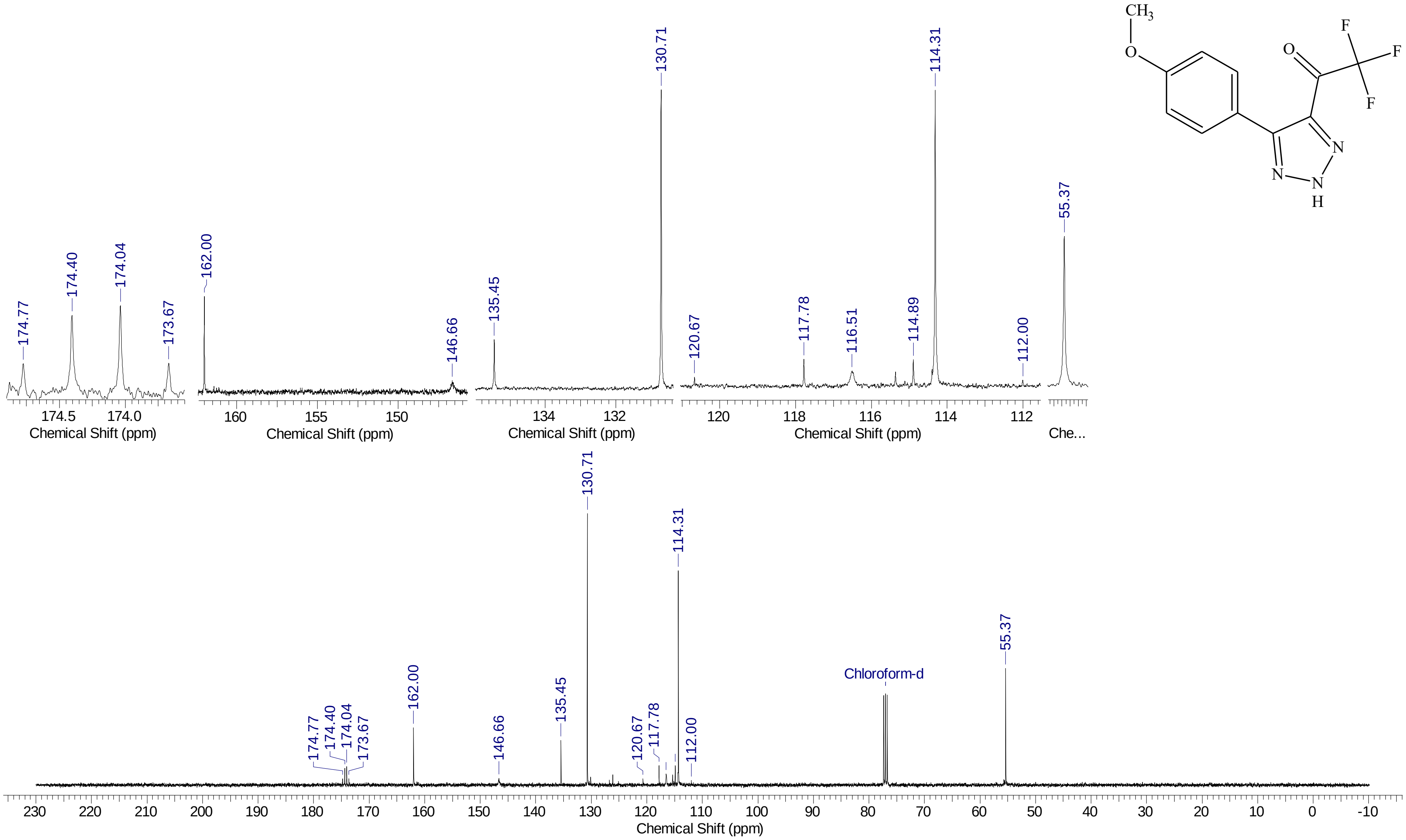

$^{13}$C NMR spectrum of **2b** (100.6 MHz, $CDCl_3$)

12 May 2022

| Acquisition Time (sec) | 2.5559 | Date | Apr 10 2018 | File Name | I:\SPEC_BM_F_2018.12.25\bm1336-2-h_20180410_01\PROTON_01 | | | |
|---|---|---|---|---|---|---|---|---|
| Frequency (MHz) | 399.97 | Nucleus | 1H | Number of Transients | 4 | Original Points Count | 16384 | Points Count 16384 |
| Pulse Sequence | s2pul | Solvent | acetone | Sweep Width (Hz) | 6410.26 | Temperature (degree C) | 22.000 | |

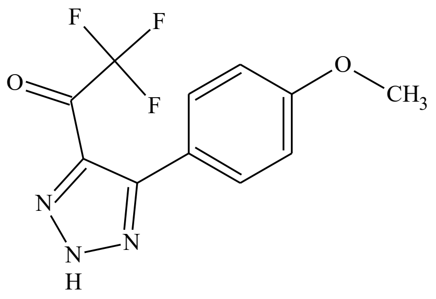


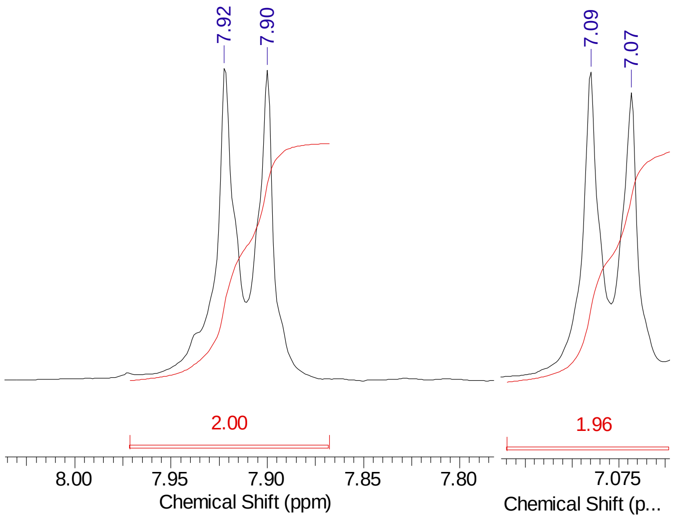


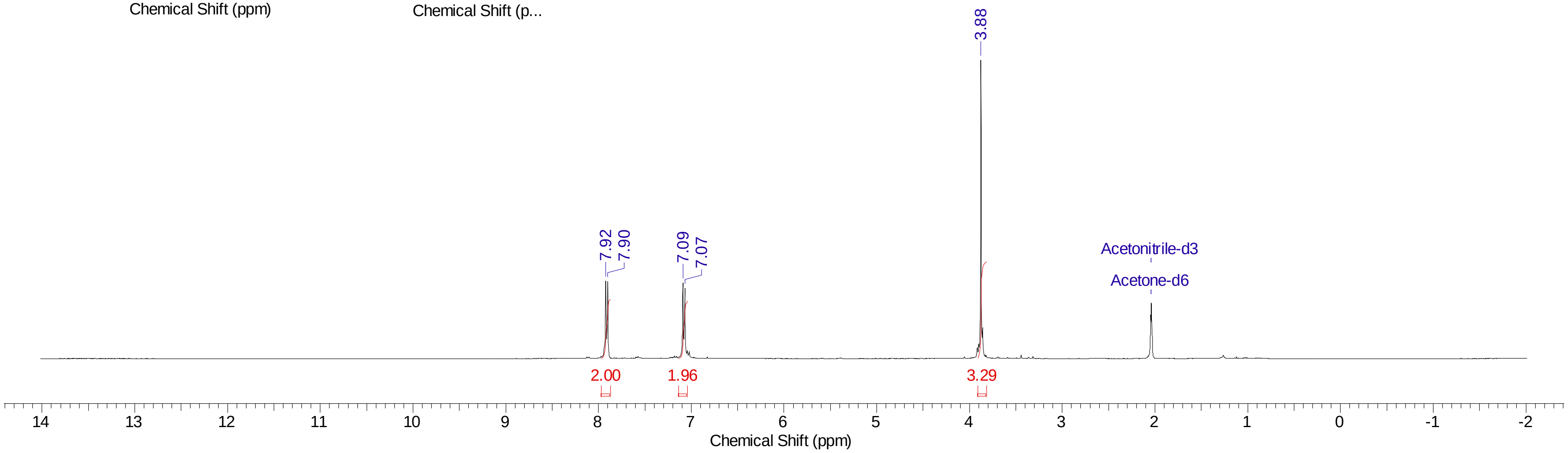

$^1$H NMR spectrum of **2b** (400.1 MHz, acetone-d$_6$)

12 May 2022

| | | | | | | | |
|---|---|---|---|---|---|---|---|
| ***Acquisition Time (sec)*** | 0.7340 | ***Date*** | Apr 10 2018 | ***File Name*** | C:\DOCS\OUTPUT_301\F19\2018.04.10\bm1336-2-f_20180410_01\FLUORINE_01 | | |
| ***Frequency (MHz)*** | 376.31 | ***Nucleus*** | 19F | ***Number of Transients*** | 100 | ***Original Points Count*** | 65536 |
| ***Points Count*** | 65536 | ***Pulse Sequence*** | s2pul | ***Solvent*** | acetone | ***Sweep Width (Hz)*** | 89285.71 |
| ***Temperature (degree C)*** | 22.000 | | | | | | |

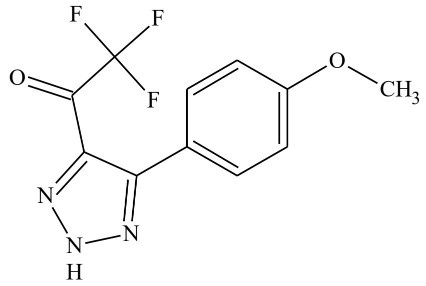


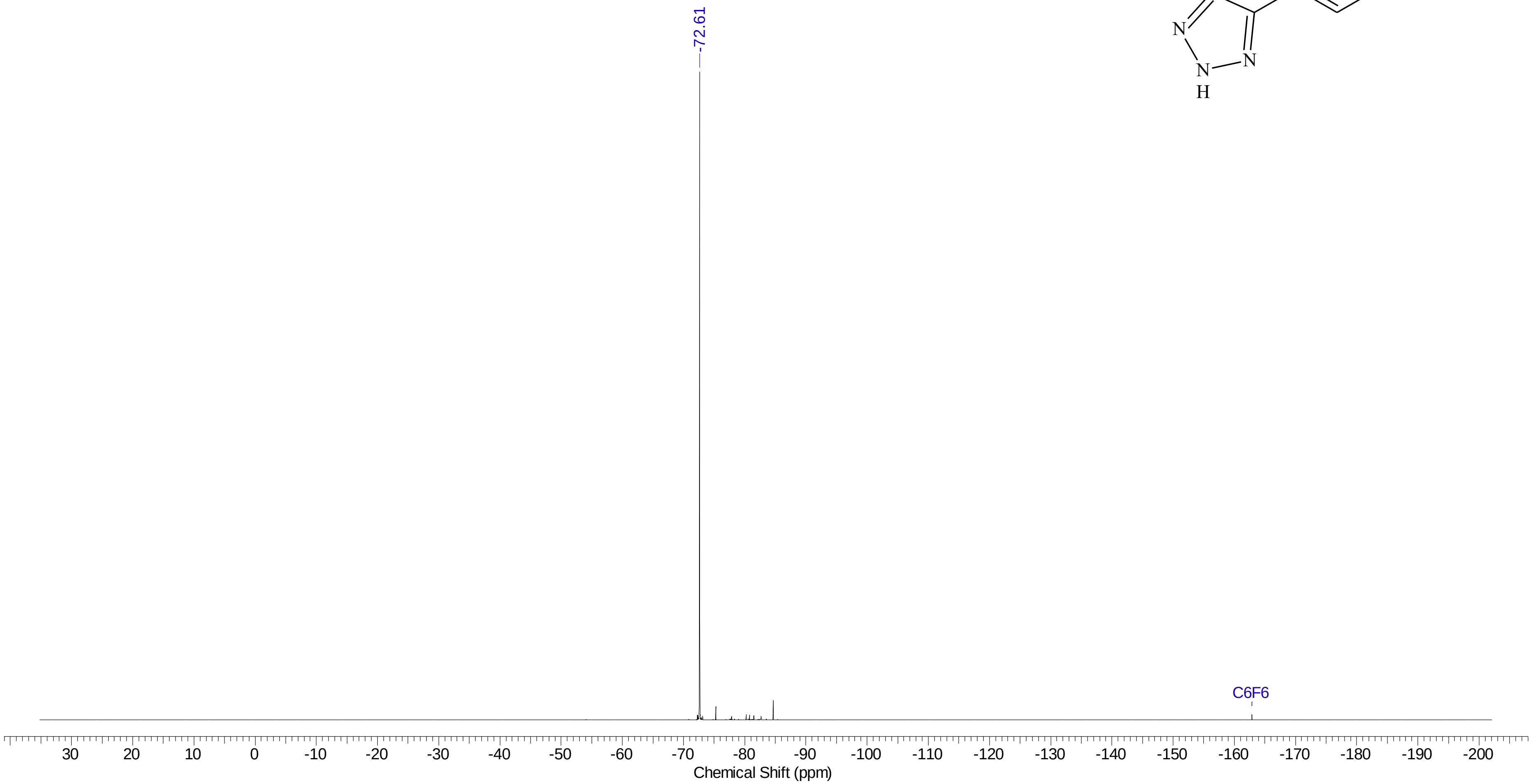

## $^{19}$F NMR spectrum of **2b** (376.5 MHz, acetone-d$_6$)

12 May 2022

| Acquisition Time (sec) | 0.4999 | Comment | Imported from UXNMR. | | | Date | 10 Apr 2018 16:05:28 |
|---|---|---|---|---|---|---|---|
| File Name | C:\BM_DATA\DOCS\SPEC_BM_H,C\BM-1336-2.C_002001r | | | | | Frequency (MHz) | 100.61 |
| Nucleus | 13C | Number of Transients | 1188 | Original Points Count | 12076 | Points Count | 65536 |
| Pulse Sequence | zgpg30 | Solvent | Acetone | Sweep Width (Hz) | 24154.59 | Temperature (degree C) | 27.000 |

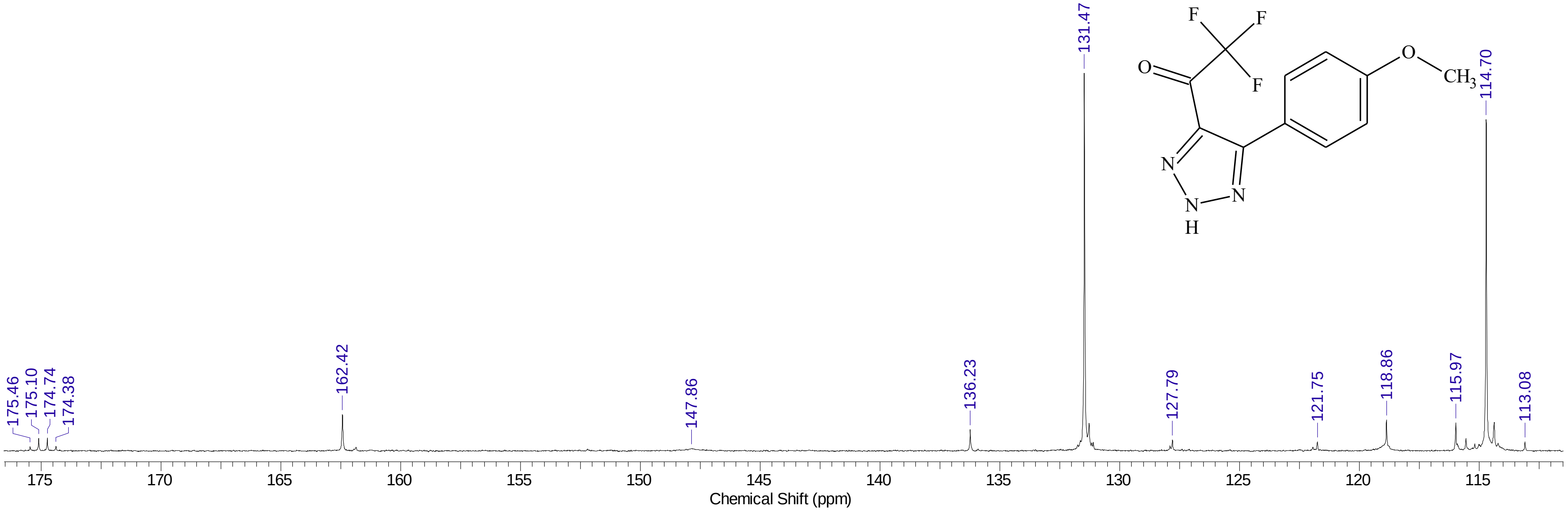


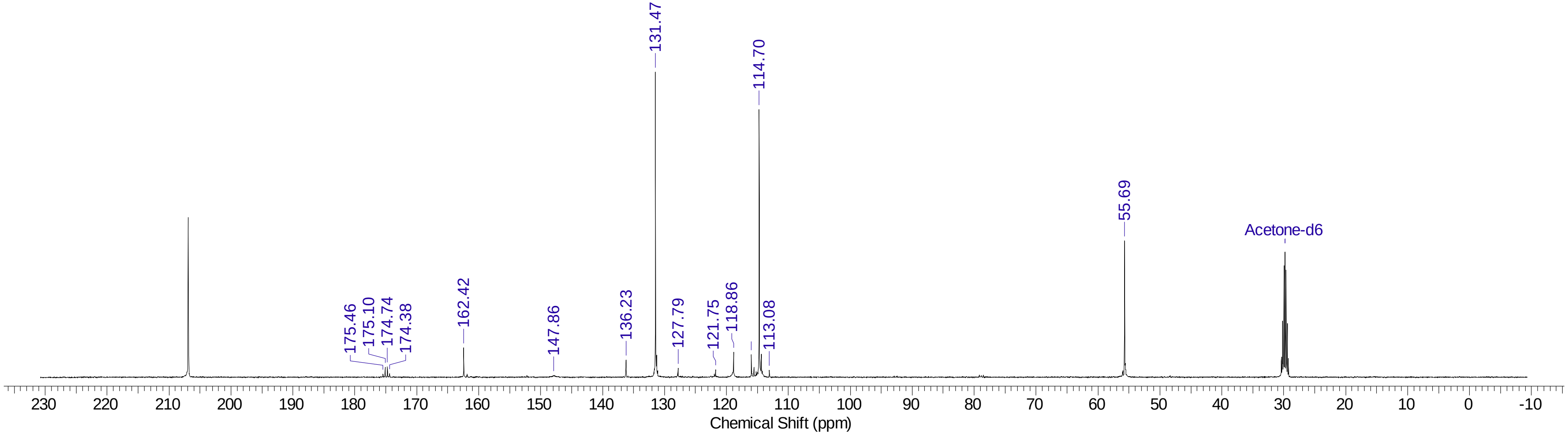

# $^{13}$C NMR spectrum of **2b** (100.6 MHz, acetone-$d_6$)

3 May 2022

| | | | | | | | |
|---|---|---|---|---|---|---|---|
| ***Acquisition Time (sec)*** | 4.0894 | ***Comment*** | Imported from UXNMR. | | | ***Date*** | 16 Feb 2022 17:36:48 |
| ***File Name*** | C:\DOCS\OUTPUT_301\2022\02.翦 怺嚯黒BM-2365-3.H_001001r | | | | | ***Frequency (MHz)*** | 400.13 |
| ***Nucleus*** | 1H | ***Number of Transients*** | 4 | ***Original Points Count*** | 32768 | ***Points Count*** | 131072 |
| ***Pulse Sequence*** | zg30 | ***Solvent*** | DEUTERIUM OXIDE | | | ***Sweep Width (Hz)*** | 8012.82 |
| ***Temperature (degree C)*** | 27.000 | | | | | | |

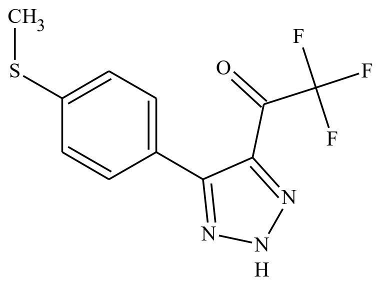


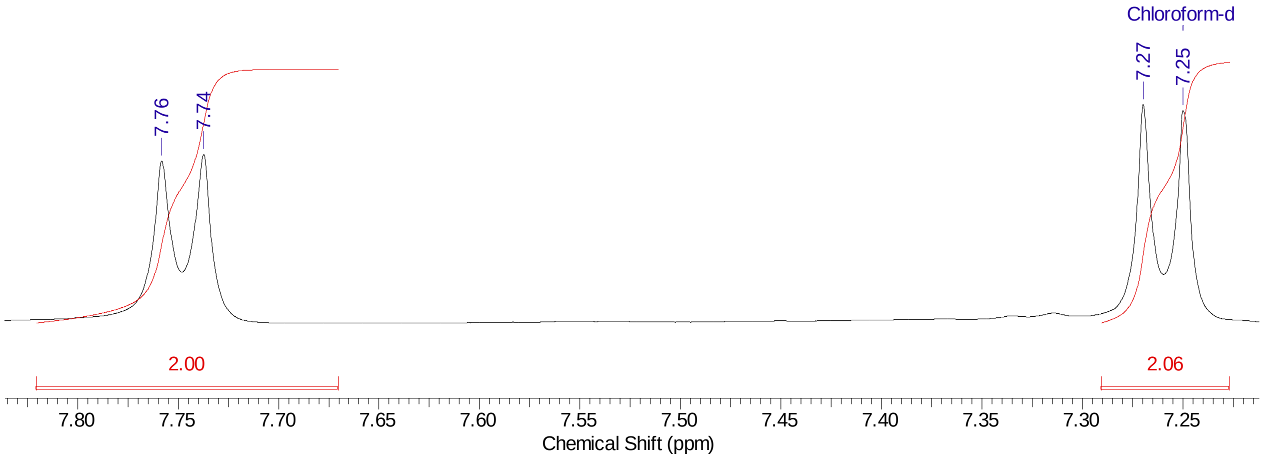


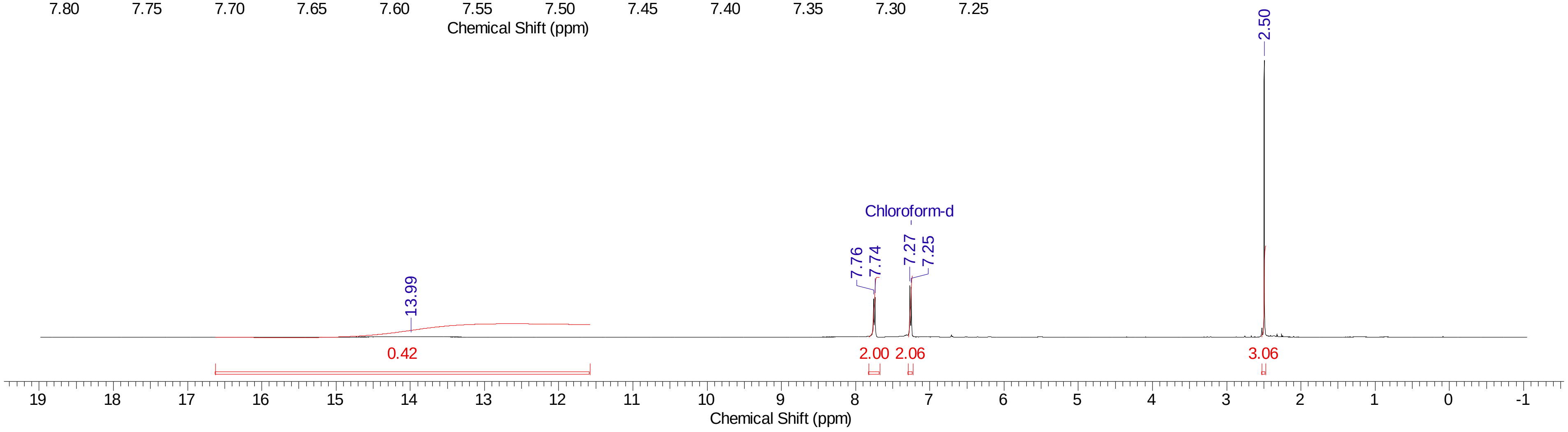

## $^{1}H$ NMR spectrum of **2c** (400.1 MHz, $CDCl_3$)

3 May 2022

| | | | | | |
|---|---|---|---|---|---|
| ***Acquisition Time (sec)*** | 1.7433 | ***Comment*** | Imported from UXNMR. | ***Date*** | 16 Feb 2022 17:51:02 |
| ***File Name*** | C:\DOCS\OUTPUT_301\2022\02.翦 怵嚯黒BM-2365-3.F_005001r | | | ***Frequency (MHz)*** | 376.50 |
| ***Nucleus*** | 19F | ***Number of Transients*** | 16 ***Original Points Count*** 131072 | ***Points Count*** | 262144 |
| ***Pulse Sequence*** | zgflqn | ***Solvent*** | CHLOROFORM-D | ***Sweep Width (Hz)*** | 75187.97 |
| ***Temperature (degree C)*** | 27.000 | | | | |

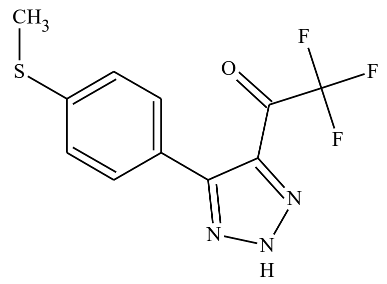


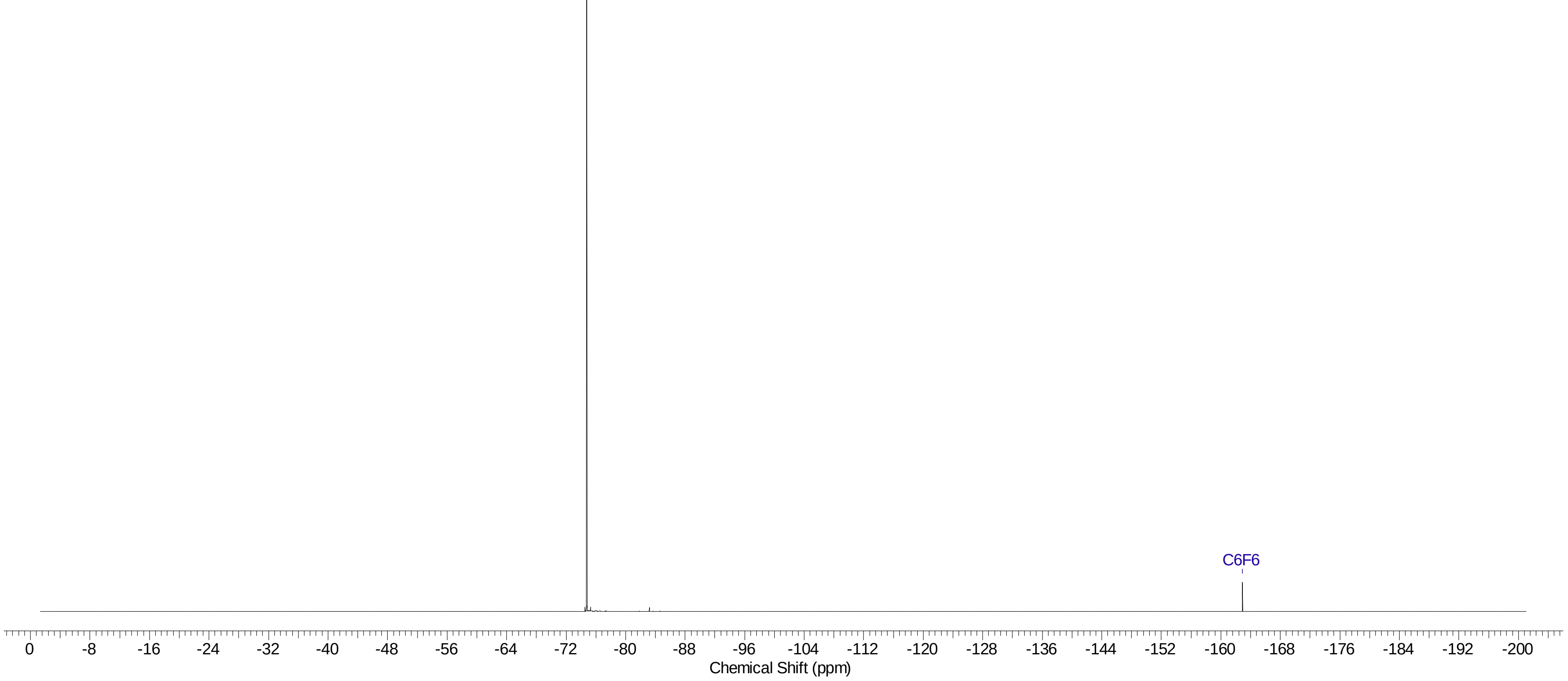

$^{19}$F NMR spectrum of **2c** (376.5 MHz, $CDCl_3$)

3 May 2022

| | | | | | | | |
|---|---|---|---|---|---|---|---|
| ***Acquisition Time (sec)*** | 0.6783 | ***Comment*** | Imported from UXNMR. | | | ***Date*** | 17 Feb 2022 12:45:10 |
| ***File Name*** | C:\BM_DATA\DOCS\咛 峈 耦鈹滂\2022\17_02_2022\BM-2365-3.C_002001r | | | | | ***Frequency (MHz)*** | 100.61 |
| ***Nucleus*** | 13C | ***Number of Transients*** | 161 | ***Original Points Count*** | 16384 | ***Points Count*** | 131072 |
| ***Pulse Sequence*** | zgpg30 | ***Solvent*** | ACETONITRILE-D3 | | | ***Sweep Width (Hz)*** | 24154.59 |
| ***Temperature (degree C)*** | 27.000 | | | | | | |

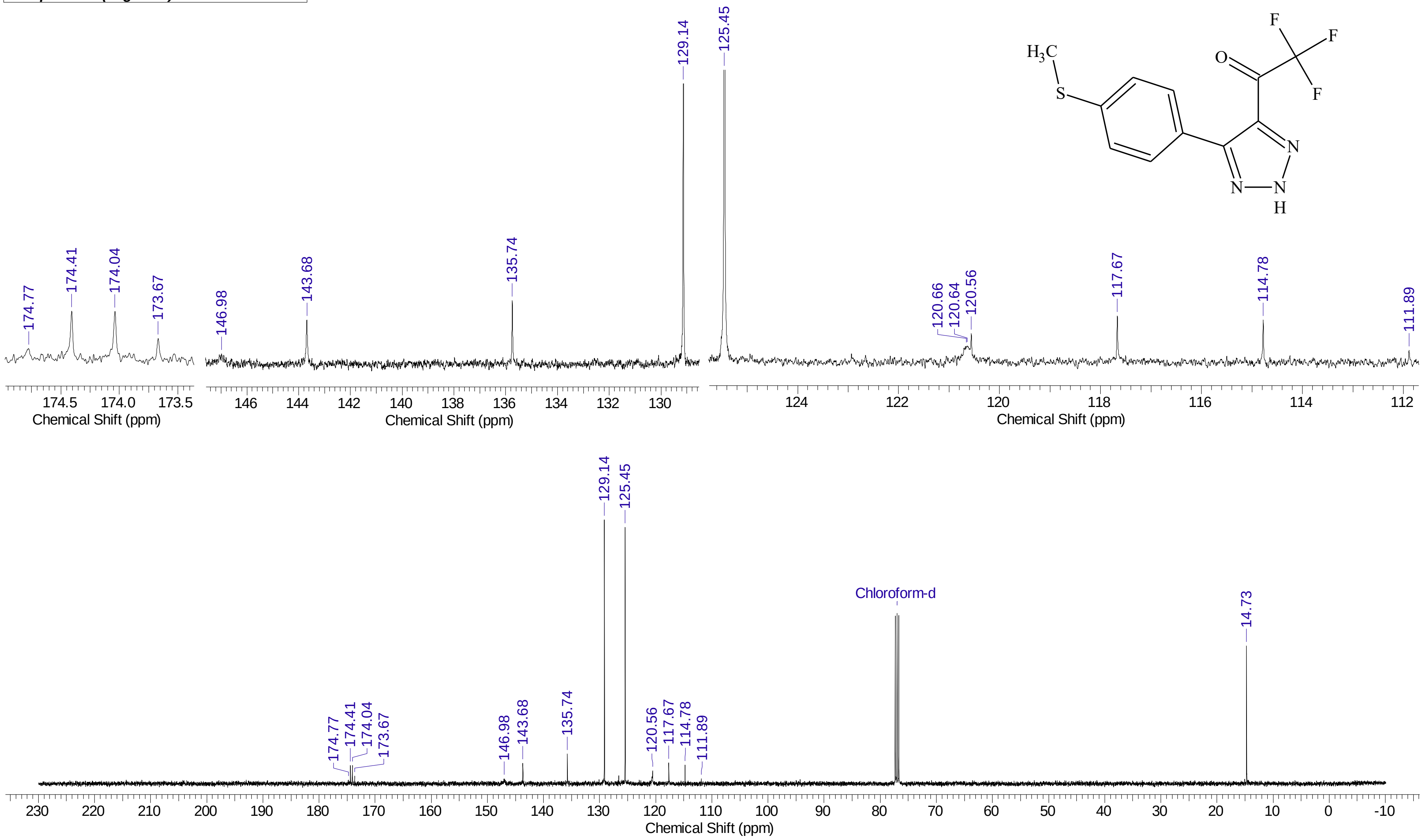

$^{13}C$ NMR spectrum of **2c** (100.6 MHz, $CDCl_3$)

12 May 2022

| | | | | | | | |
|---|---|---|---|---|---|---|---|
| ***Acquisition Time (sec)*** | 2.5559 | ***Comment*** | Imported from UXNMR. | | | ***Date*** | 06 Apr 2018 15:06:24 |
| ***File Name*** | C:\BM_DATA\DOCS\SPEC_BM_H,C\BM-1327-3S.H_001001r | | | | | ***Frequency (MHz)*** | 400.13 |
| ***Nucleus*** | 1H | ***Number of Transients*** | 4 | ***Original Points Count*** | 16384 | ***Points Count*** | 65536 |
| ***Pulse Sequence*** | zg30 | ***Solvent*** | CHLOROFORM-D | | | ***Sweep Width (Hz)*** | 6410.26 |
| ***Temperature (degree C)*** | 27.000 | | | | | | |

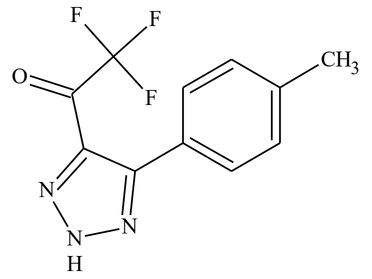


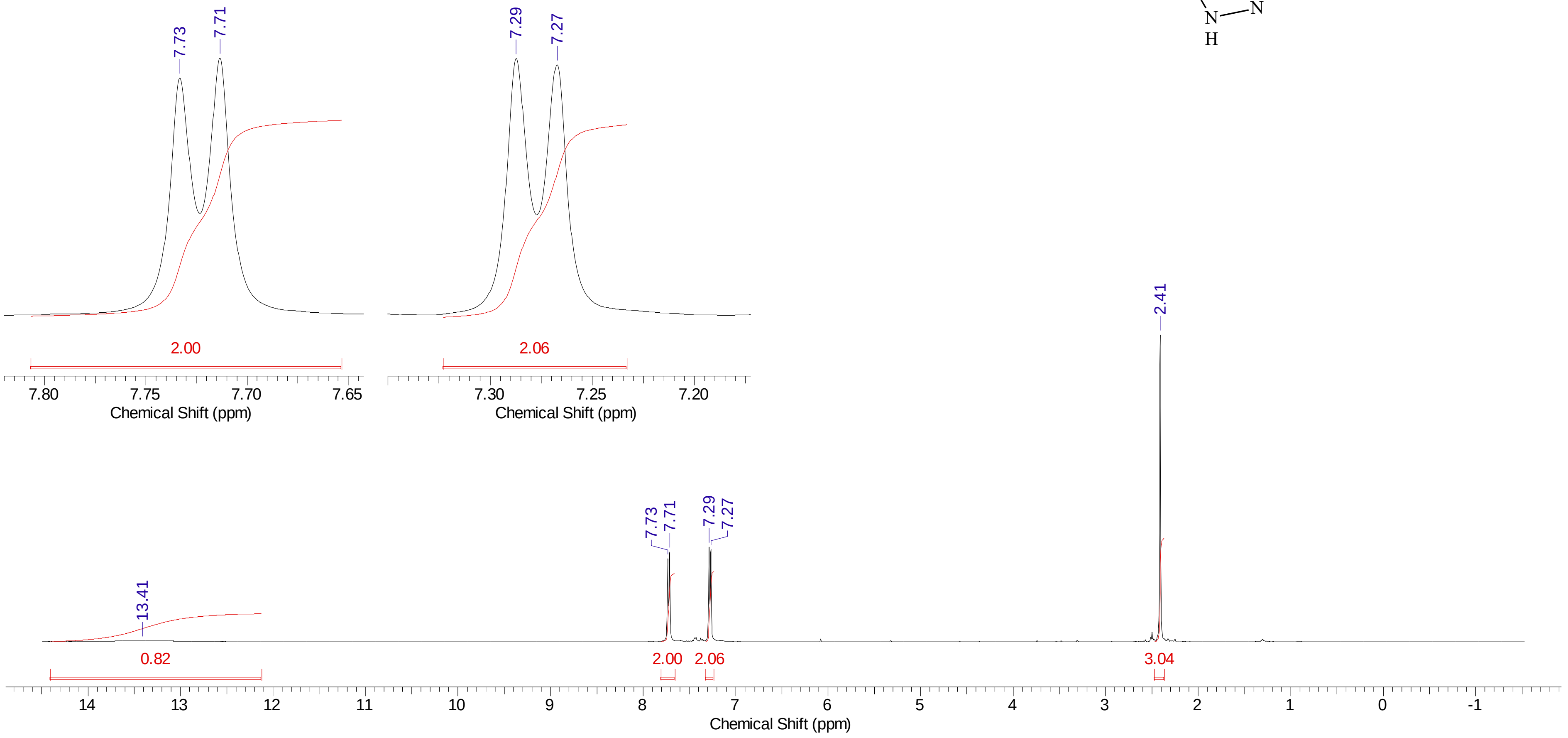

$^{1}$H NMR spectrum of **2d** (400.1 MHz, $CDCl_3$)

12 May 2022

| | | | | | | | |
|---|---|---|---|---|---|---|---|
| ***Acquisition Time (sec)*** | 2.7263 | ***Date*** | Apr 5 2018 | ***File Name*** | I:\SPEC_BM_F_2018.12.25\BM-1327-3S-F_20180405_01\FLUORINE_01 | | |
| ***Frequency (MHz)*** | 376.31 | ***Nucleus*** | 19F | ***Number of Transients*** | 8 | ***Original Points Count*** | 262144 |
| ***Points Count*** | 262144 | ***Pulse Sequence*** | s2pul | ***Solvent*** | CHLOROFORM-D | | |
| ***Sweep Width (Hz)*** | 96153.84 | ***Temperature (degree C)*** | 22.000 | | | | |

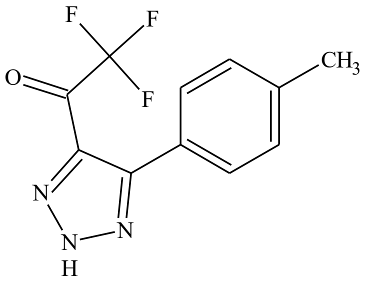


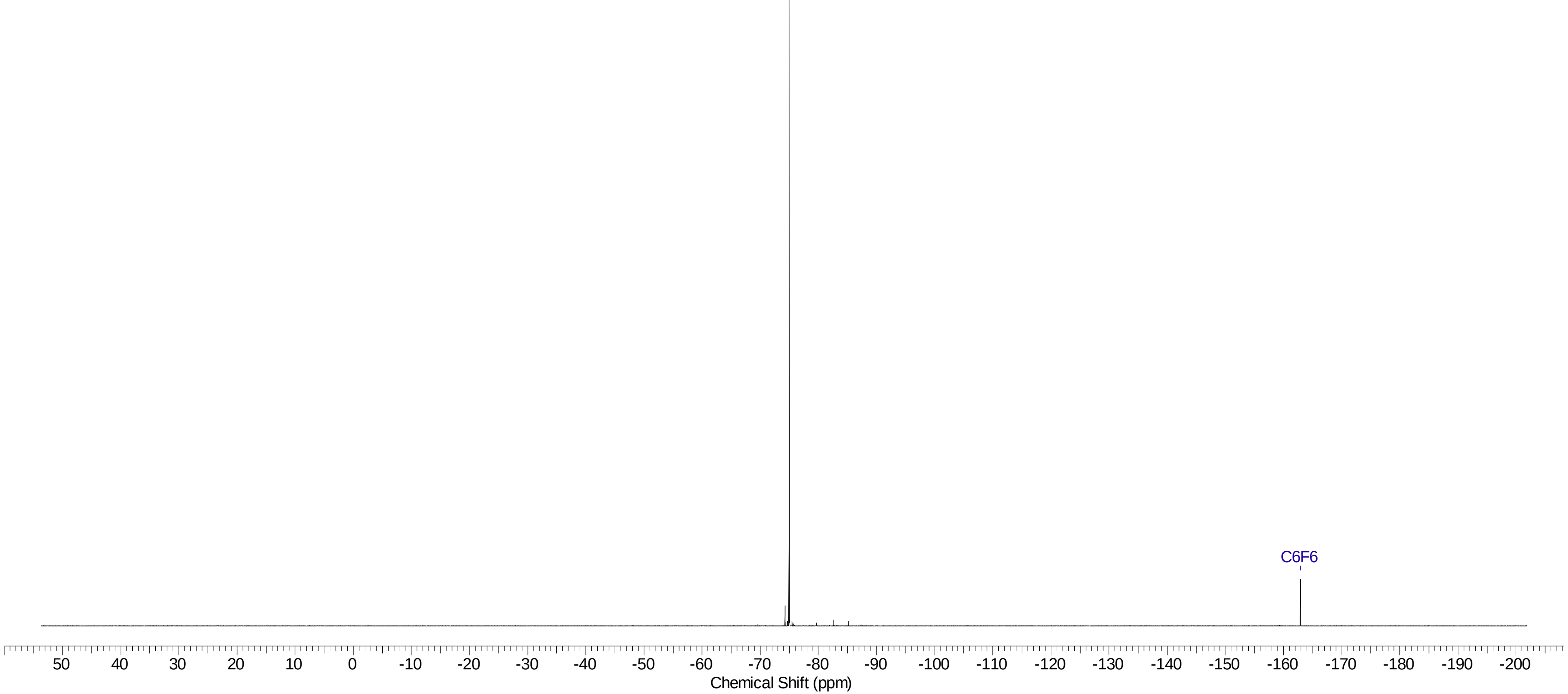

$^{19}$F NMR spectrum of **2d** (376.5 MHz, $CDCl_3$)

12 May 2022

| | | | | | | | |
|---|---|---|---|---|---|---|---|
| ***Acquisition Time (sec)*** | 0.4999 | ***Comment*** | Imported from UXNMR. | | | ***Date*** | 06 Apr 2018 15:09:52 |
| ***File Name*** | C:\BM_DATA\DOCS\SPEC_BM_H,C\BM-1327-3S.C_002001r | | | ***Frequency (MHz)*** | 100.61 | ***Nucleus*** | 13C |
| ***Number of Transients*** | 64 | ***Original Points Count*** | 12076 | ***Points Count*** | 65536 | ***Pulse Sequence*** | zgpg30 |
| ***Solvent*** | DEUTERIUM OXIDE | | | ***Sweep Width (Hz)*** | 24154.59 | ***Temperature (degree C)*** | 27.000 |

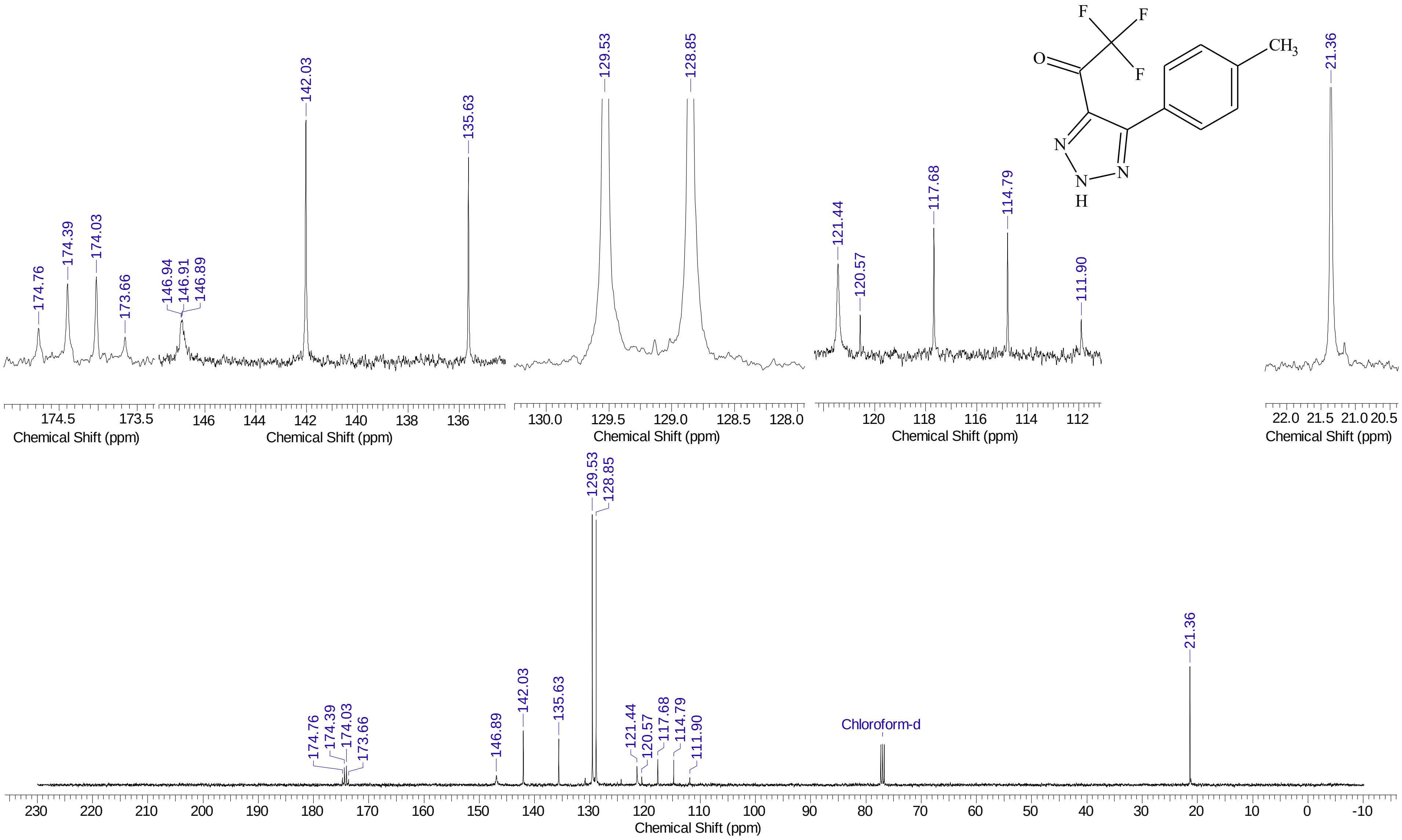

$^{13}$C NMR spectrum of **2d** (100.6 MHz, $CDCl_3$)

3 May 2022

| | | | | | | | |
|---|---|---|---|---|---|---|---|
| ***Acquisition Time (sec)*** | 4.0894 | ***Comment*** | Imported from UXNMR. | | | ***Date*** | 15 Feb 2022 15:08:46 |
| ***File Name*** | C:\DOCS\OUTPUT_301\2022\02.翦 怵嚯黑BM-2214-2.H_001001r | | | | | ***Frequency (MHz)*** | 400.13 |
| ***Nucleus*** | 1H | ***Number of Transients*** | 4 | ***Original Points Count*** | 32768 | ***Points Count*** | 131072 |
| ***Pulse Sequence*** | zg30 | ***Solvent*** | DEUTERIUM OXIDE | | | ***Sweep Width (Hz)*** | 8012.82 |
| ***Temperature (degree C)*** | 27.000 | | | | | | |

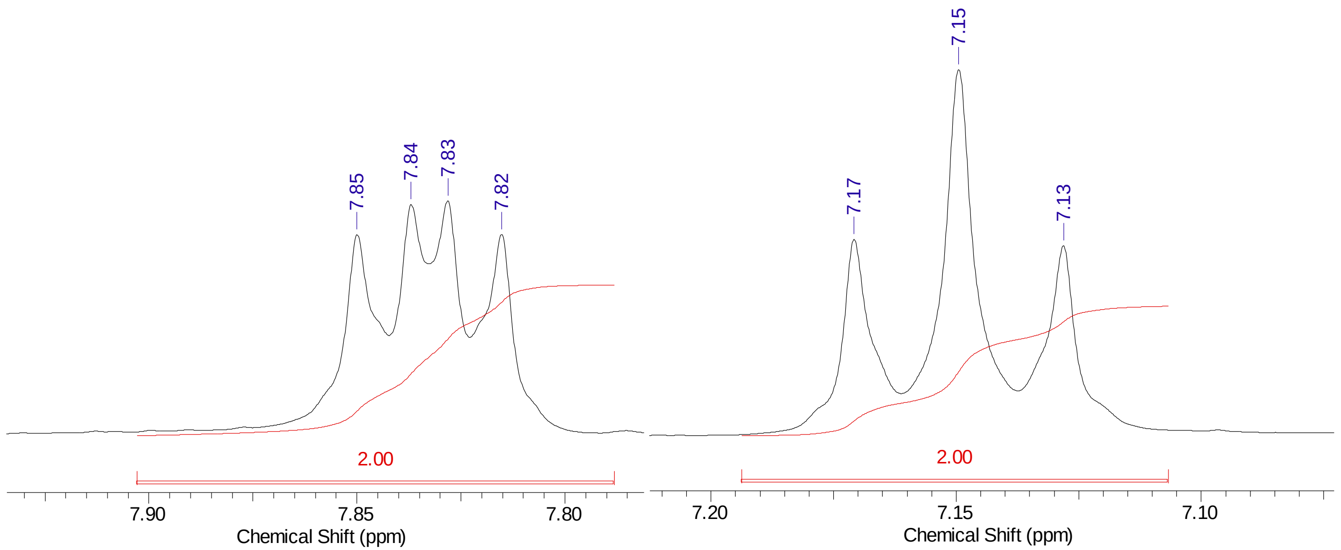


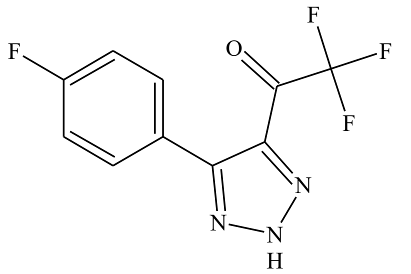


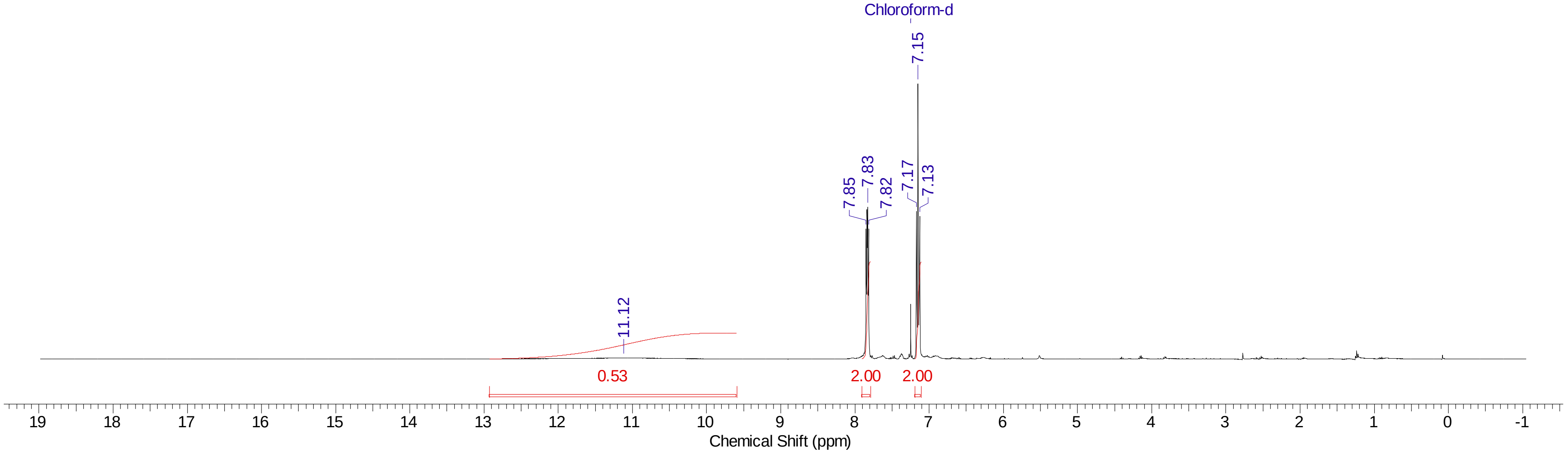

## $^{1}H$ NMR spectrum of **2e** (400.1 MHz, $CDCl_3$)

3 May 2022

| | | | | | | | |
|---|---|---|---|---|---|---|---|
| ***Acquisition Time (sec)*** | 1.7433 | ***Comment*** | Imported from UXNMR. | | | ***Date*** | 15 Feb 2022 15:38:54 |
| ***File Name*** | C:\DOCS\OUTPUT_301\2022\02.翦 怵嚯黒BM-2214-2.F_005001r | | | | | ***Frequency (MHz)*** | 376.50 |
| ***Nucleus*** | 19F | ***Number of Transients*** | 13 | ***Original Points Count*** | 131072 | ***Points Count*** | 262144 |
| ***Pulse Sequence*** | zgflqn | ***Solvent*** | CHLOROFORM-D | | | ***Sweep Width (Hz)*** | 75187.97 |
| ***Temperature (degree C)*** | 27.000 | | | | | | |

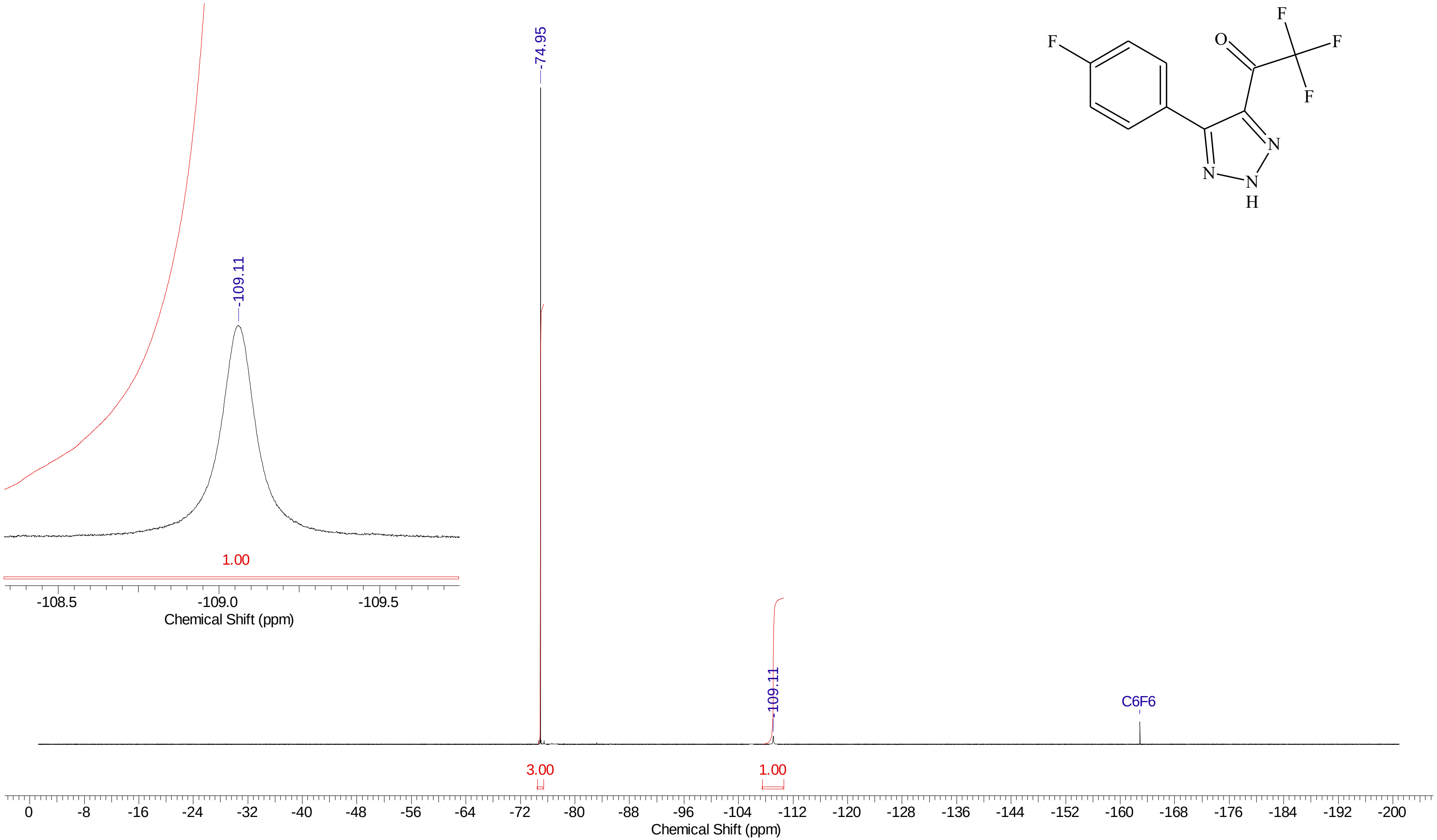

## $^{19}$F NMR spectrum of **2e** (376.5 MHz, $CDCl_3$)

3 May 2022

| | | | | | |
|---|---|---|---|---|---|
| ***Acquisition Time (sec)*** | 0.6783 | ***Comment*** | Imported from UXNMR. | ***Date*** | 15 Feb 2022 15:17:16 |
| ***File Name*** | C:\DOCS\OUTPUT_301\2022\02.翦 怺嚾熏BM-2214-2.C_002001r | | | ***Frequency (MHz)*** | 100.61 |
| ***Nucleus*** | 13C | ***Number of Transients*** | 218 ***Original Points Count*** 16384 | ***Points Count*** | 131072 |
| ***Pulse Sequence*** | zgpg30 | ***Solvent*** | ACETONITRILE-D3 | ***Sweep Width (Hz)*** | 24154.59 |
| ***Temperature (degree C)*** | 27.000 | | | | |

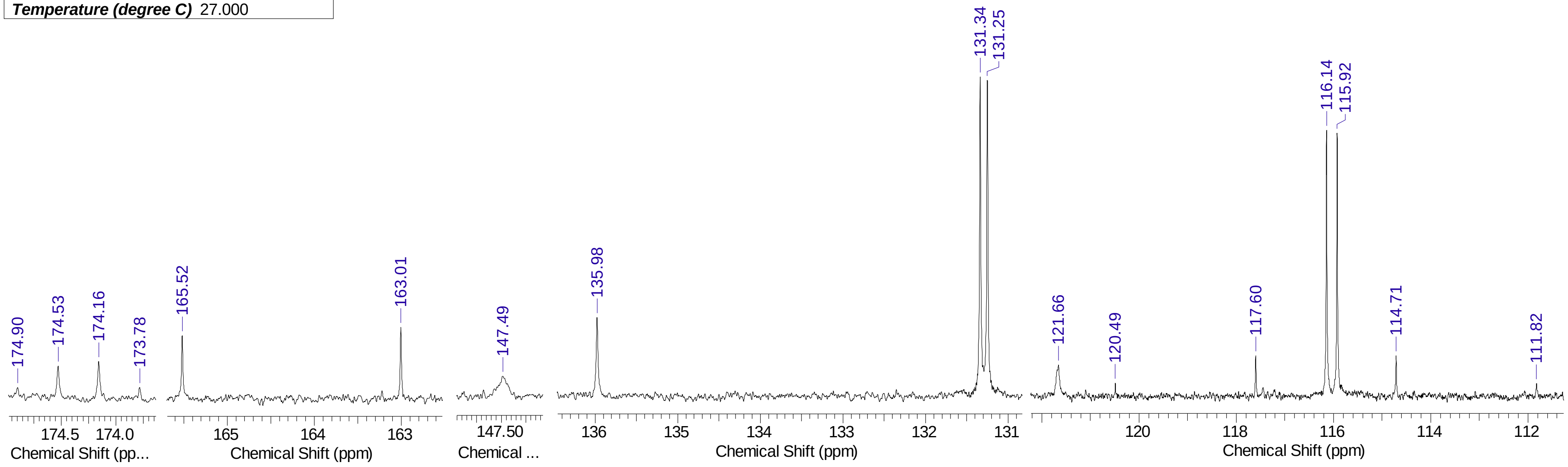


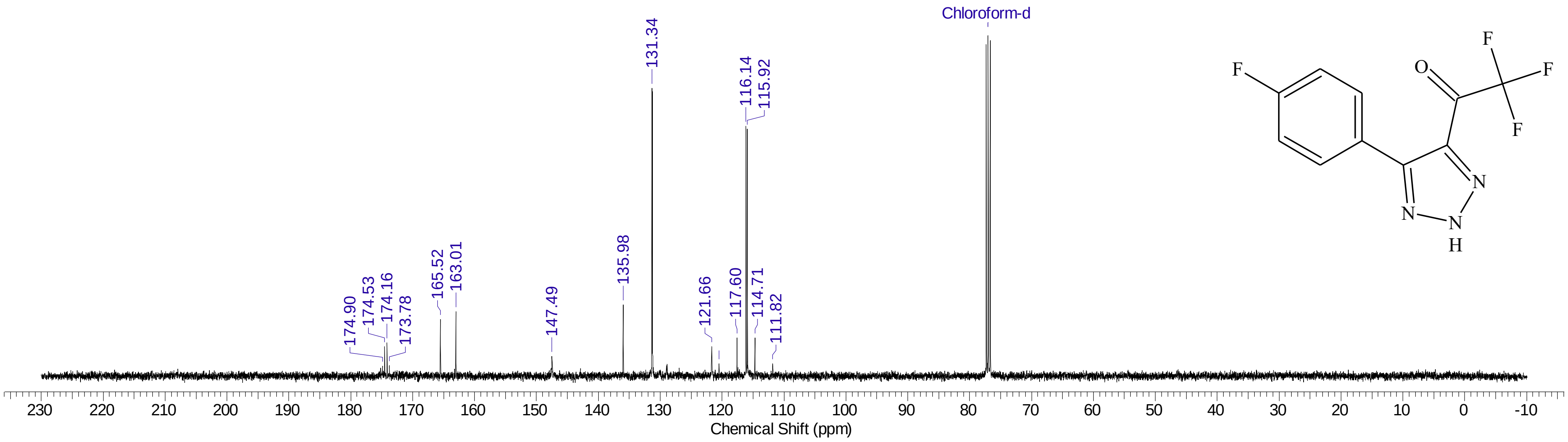

## $^{13}$C NMR spectrum of **2e** (100.6 MHz, $CDCl_3$)

3 May 2022

| | | | | | | | |
|---|---|---|---|---|---|---|---|
| ***Acquisition Time (sec)*** | 4.0894 | ***Comment*** | Imported from UXNMR. | | | ***Date*** | 14 Feb 2022 15:23:32 |
| ***File Name*** | C:\DOCS\OUTPUT_301\2022\02.翦 怵嚯黒BM-2357-3.H_001001r | | | | | ***Frequency (MHz)*** | 400.13 |
| ***Nucleus*** | 1H | ***Number of Transients*** | 4 | ***Original Points Count*** | 32768 | ***Points Count*** | 131072 |
| ***Pulse Sequence*** | zg30 | ***Solvent*** | CHLOROFORM-D | | | ***Sweep Width (Hz)*** | 8012.82 |
| ***Temperature (degree C)*** | 27.000 | | | | | | |

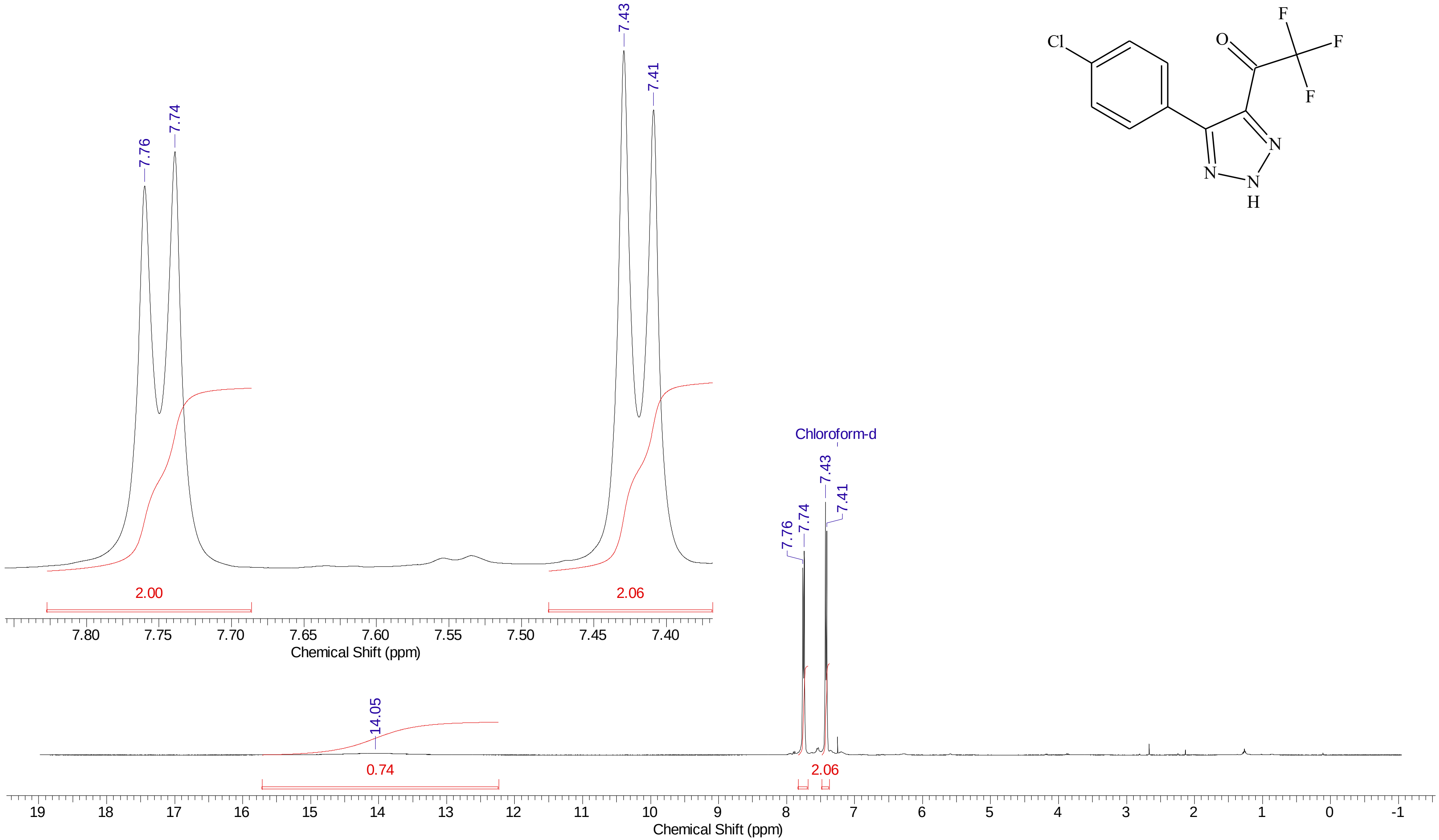

## $^{1}$H NMR spectrum of **2f** (400.1 MHz, $CDCl_3$)

3 May 2022

| | | | | | | | |
|---|---|---|---|---|---|---|---|
| ***Acquisition Time (sec)*** | 1.7433 | ***Comment*** | Imported from UXNMR. | | | ***Date*** | 12 Feb 2022 22:16:28 |
| ***File Name*** | C:\BM_DATA\DOCS\吖 峈耦皱滂\2022\bm220212\BM-2357-3_005001r | | | | | ***Frequency (MHz)*** | 376.50 |
| ***Nucleus*** | 19F | ***Number of Transients*** | 16 | ***Original Points Count*** | 131072 | ***Points Count*** | 262144 |
| ***Pulse Sequence*** | zgflqn | ***Solvent*** | CHLOROFORM-D | | | ***Sweep Width (Hz)*** | 75187.97 |
| ***Temperature (degree C)*** | 27.000 | | | | | | |

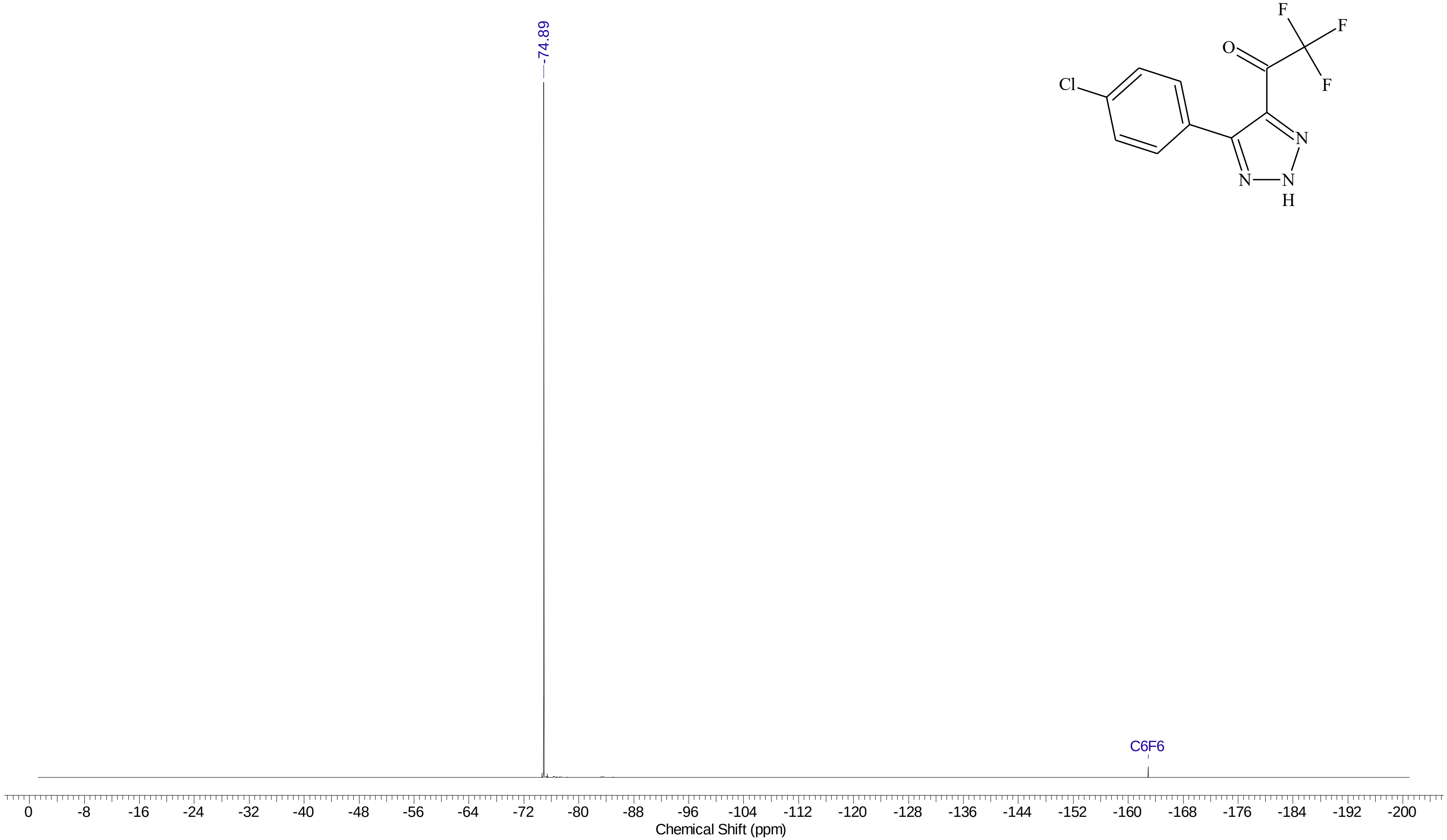

## $^{19}$F NMR spectrum of **2f** (376.5 MHz, $CDCl_3$)

3 May 2022

| | | | | | | | |
|---|---|---|---|---|---|---|---|
| ***Acquisition Time (sec)*** | 0.6783 | ***Comment*** | Imported from UXNMR. | | | ***Date*** | 14 Feb 2022 15:30:14 |
| ***File Name*** | C:\DOCS\OUTPUT_301\2022\02.翦 怵嚯黑BM-2357-3.C_002001r | | | | | ***Frequency (MHz)*** | 100.61 |
| ***Nucleus*** | 13C | ***Number of Transients*** | 129 | ***Original Points Count*** | 16384 | ***Points Count*** | 131072 |
| ***Pulse Sequence*** | zgpg30 | ***Solvent*** | ACETONITRILE-D3 | | | ***Sweep Width (Hz)*** | 24154.59 |
| ***Temperature (degree C)*** | 27.000 | | | | | | |

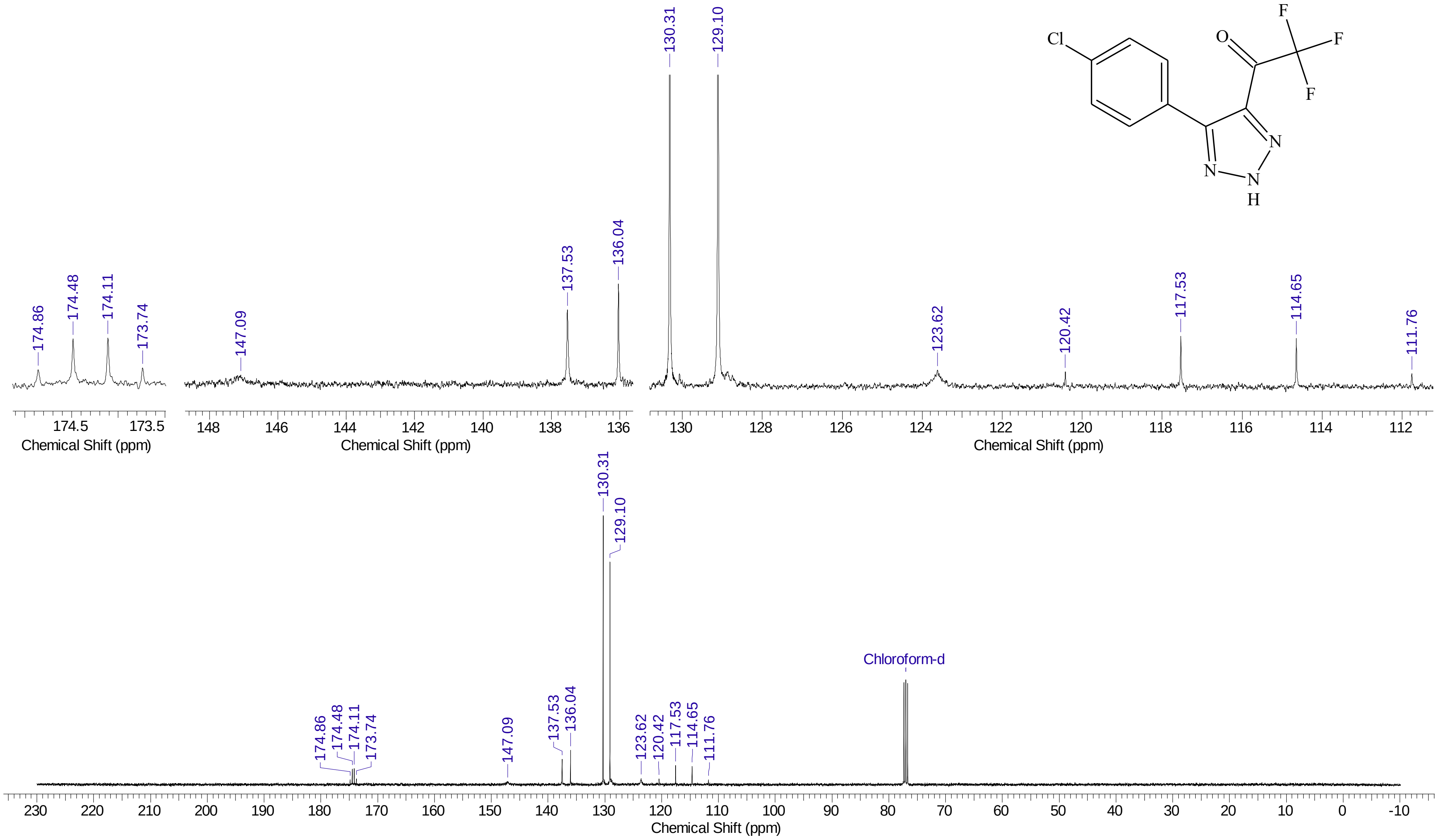

# $^{13}C$ NMR spectrum of **2f** (100.6 MHz, $CDCl_3$)

3 May 2022

| | | | | | |
|---|---|---|---|---|---|
| ***Acquisition Time (sec)*** | 4.0894 | ***Comment*** | Imported from UXNMR. | ***Date*** | 15 Feb 2022 16:05:56 |
| ***File Name*** | C:\DOCS\OUTPUT_301\2022\02.翦 怵嚯黒BM-2363-3.H_001001r | | | ***Frequency (MHz)*** | 400.13 |
| ***Nucleus*** | 1H | ***Number of Transients*** | 4 ***Original Points Count*** 32768 | ***Points Count*** | 131072 |
| ***Pulse Sequence*** | zg30 | ***Solvent*** | DEUTERIUM OXIDE | ***Sweep Width (Hz)*** | 8012.82 |
| ***Temperature (degree C)*** | 27.000 | | | | |

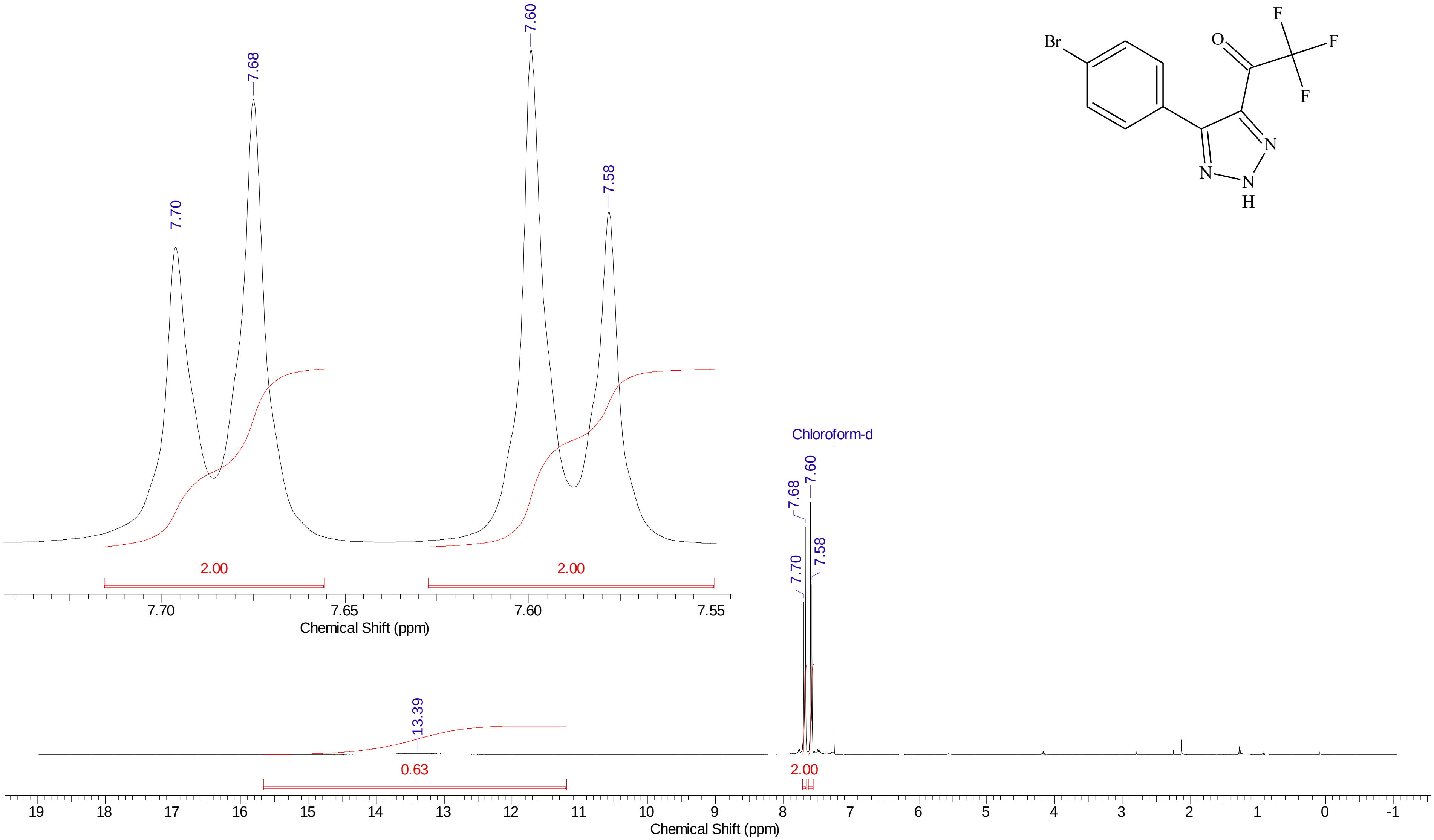

## $^{1}$H NMR spectrum of **2g** (400.1 MHz, $CDCl_3$)

3 May 2022

| | | | | | | | |
|---|---|---|---|---|---|---|---|
| ***Acquisition Time (sec)*** | 1.7433 | ***Comment*** | Imported from UXNMR. | | | ***Date*** | 15 Feb 2022 16:04:44 |
| ***File Name*** | C:\DOCS\OUTPUT_301\2022\02.翦 怺嚯黒BM-2363-3.F_005001r | | | | | ***Frequency (MHz)*** | 376.50 |
| ***Nucleus*** | 19F | ***Number of Transients*** | 6 | ***Original Points Count*** | 131072 | ***Points Count*** | 262144 |
| ***Pulse Sequence*** | zgflqn | ***Solvent*** | CHLOROFORM-D | | | ***Sweep Width (Hz)*** | 75187.97 |
| ***Temperature (degree C)*** | 27.000 | | | | | | |

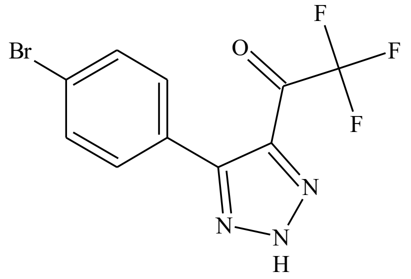

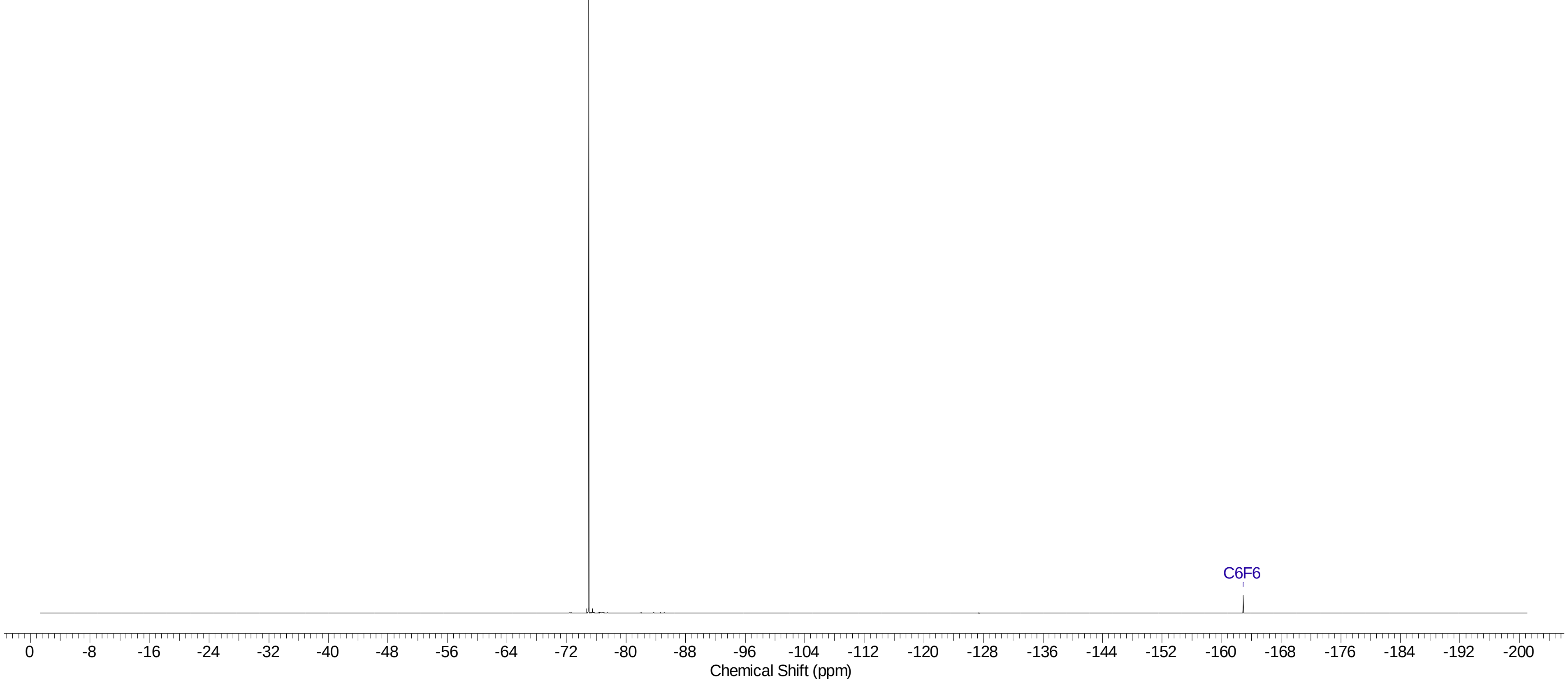

$^{19}$F NMR spectrum of **2g** (376.5 MHz, $CDCl_3$)

3 May 2022

| | | | | | | | |
|---|---|---|---|---|---|---|---|
| ***Acquisition Time (sec)*** | 0.6783 | ***Comment*** | Imported from UXNMR. | | | ***Date*** | 17 Feb 2022 12:37:00 |
| ***File Name*** | C:\BM_DATA\DOCS\吋 峪 耦鈹滂\2022\17_02_2022\BM-2363-3.C_002001r | | | | | ***Frequency (MHz)*** | 100.61 |
| ***Nucleus*** | 13C | ***Number of Transients*** | 81 | ***Original Points Count*** | 16384 | ***Points Count*** | 131072 |
| ***Pulse Sequence*** | zgpg30 | ***Solvent*** | ACETONITRILE-D3 | | | ***Sweep Width (Hz)*** | 24154.59 |
| ***Temperature (degree C)*** | 27.000 | | | | | | |

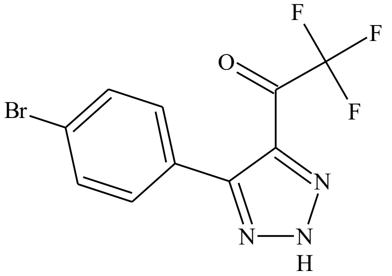


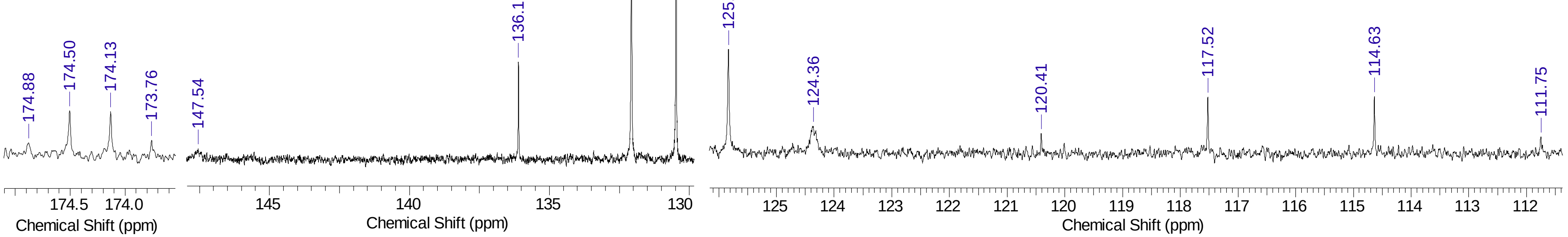


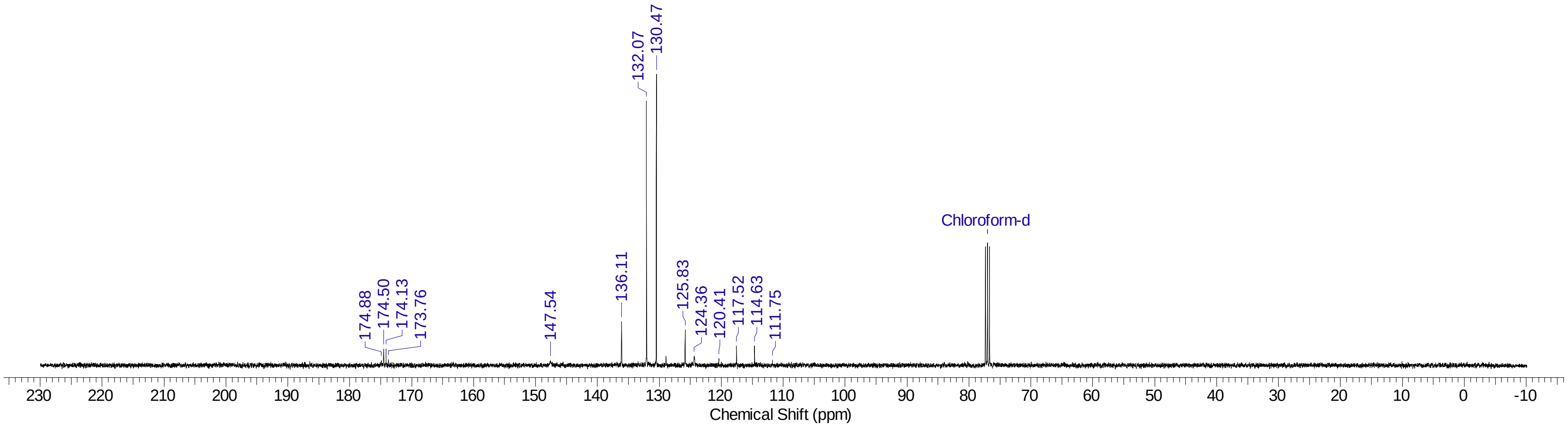

# $^{13}$C NMR spectrum of **2g** (100.6 MHz, $CDCl_3$)

13 May 2022

| | | | | | | | |
|---|---|---|---|---|---|---|---|
| ***Acquisition Time (sec)*** | 4.0894 | ***Comment*** | Imported from UXNMR. | | | ***Date*** | 02 Mar 2022 17:45:38 |
| ***File Name*** | C:\DOCS\OUTPUT_301\2022\03.爨 痱\SZA-BM-2374.H_001001r | | | | | ***Frequency (MHz)*** | 400.13 |
| ***Nucleus*** | 1H | ***Number of Transients*** | 4 | ***Original Points Count*** | 32768 | ***Points Count*** | 131072 |
| ***Pulse Sequence*** | zg30 | ***Solvent*** | DEUTERIUM OXIDE | | | ***Sweep Width (Hz)*** | 8012.82 |
| ***Temperature (degree C)*** | 27.000 | | | | | | |

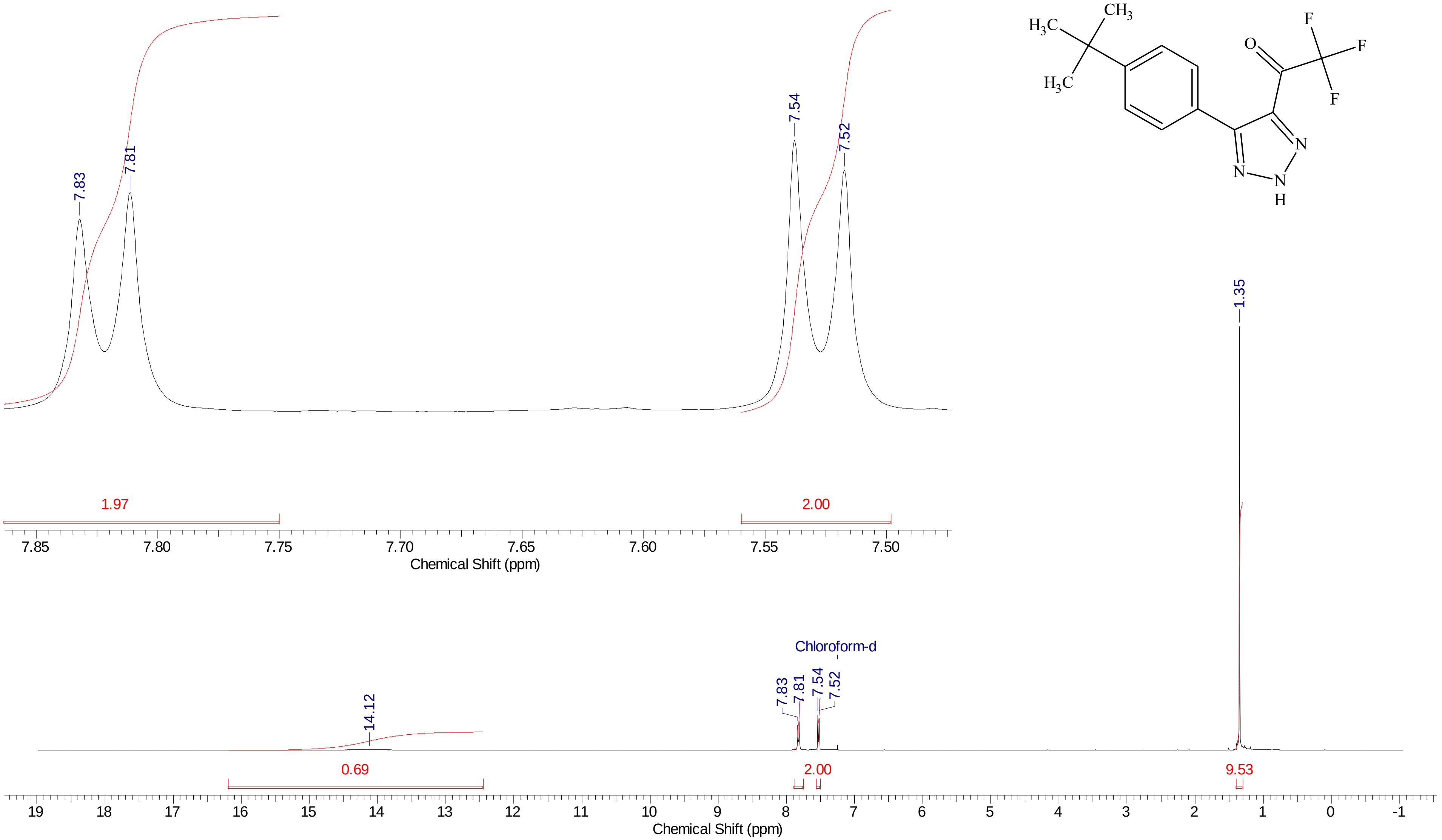

$^1$H NMR spectrum of **2h** (400.1 MHz, $CDCl_3$)

13 May 2022

| | | | | | | | |
|---|---|---|---|---|---|---|---|
| ***Acquisition Time (sec)*** | 1.7433 | ***Comment*** | Imported from UXNMR. | | | ***Date*** | 02 Mar 2022 17:50:18 |
| ***File Name*** | C:\DOCS\OUTPUT_301\2022\03.爨 痱\SZA-BM-2374.F_005001r | | | | | ***Frequency (MHz)*** | 376.50 |
| ***Nucleus*** | 19F | ***Number of Transients*** | 4 | ***Original Points Count*** | 131072 | ***Points Count*** | 262144 |
| ***Pulse Sequence*** | zgflqn | ***Solvent*** | CHLOROFORM-D | | | ***Sweep Width (Hz)*** | 75187.97 |
| ***Temperature (degree C)*** | 27.000 | | | | | | |

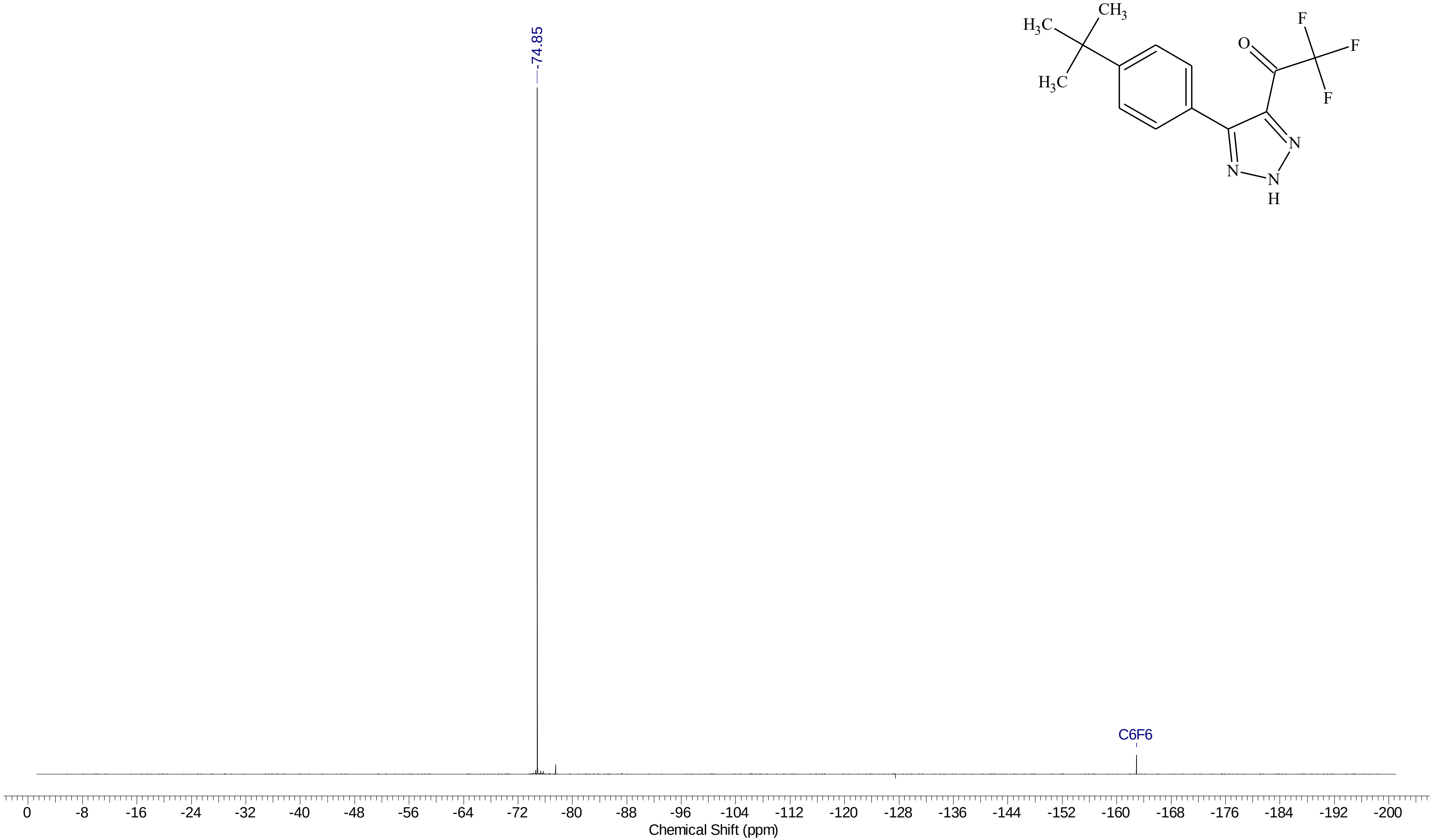

# $^{19}$F NMR spectrum of **2h** (376.5 MHz, $CDCl_3$)

13 May 2022

| | | | | | | | |
|---|---|---|---|---|---|---|---|
| ***Acquisition Time (sec)*** | 0.6783 | ***Comment*** | Imported from UXNMR. | | | ***Date*** | 03 Mar 2022 12:01:12 |
| ***File Name*** | C:\DOCS\OUTPUT_301\2022\03.爨 痱\SZA-BM-2374.C_002001r | | | | | ***Frequency (MHz)*** | 100.61 |
| ***Nucleus*** | 13C | ***Number of Transients*** | 845 | ***Original Points Count*** | 16384 | ***Points Count*** | 131072 |
| ***Pulse Sequence*** | zgpg30 | ***Solvent*** | ACETONITRILE-D3 | | | ***Sweep Width (Hz)*** | 24154.59 |
| ***Temperature (degree C)*** | 27.000 | | | | | | |

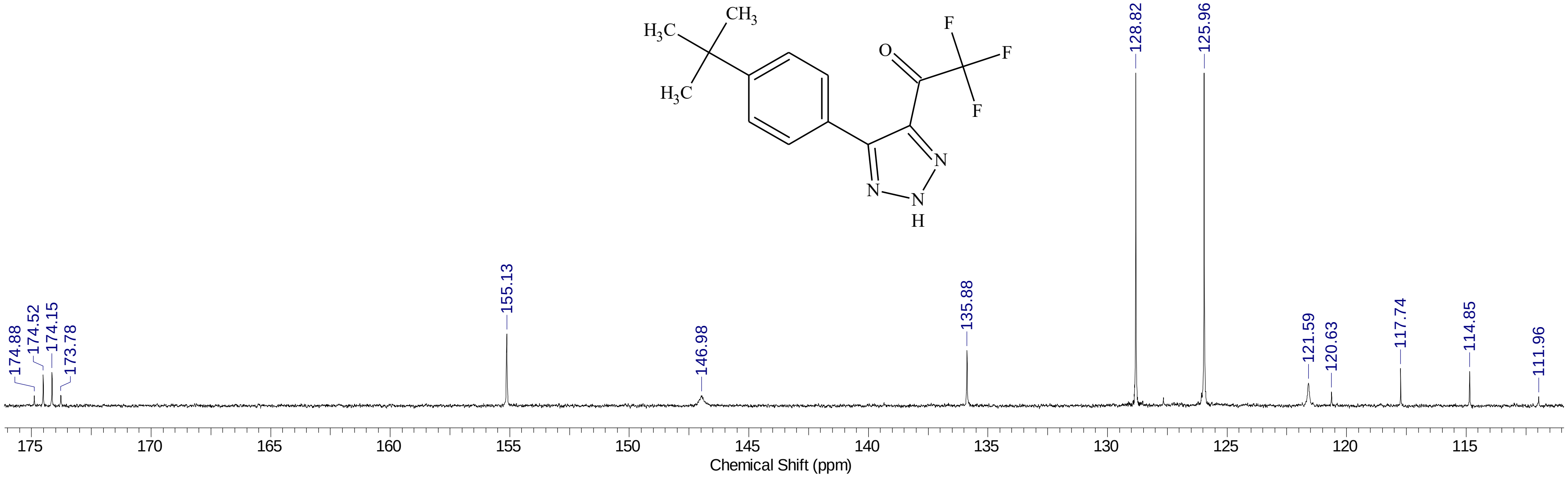


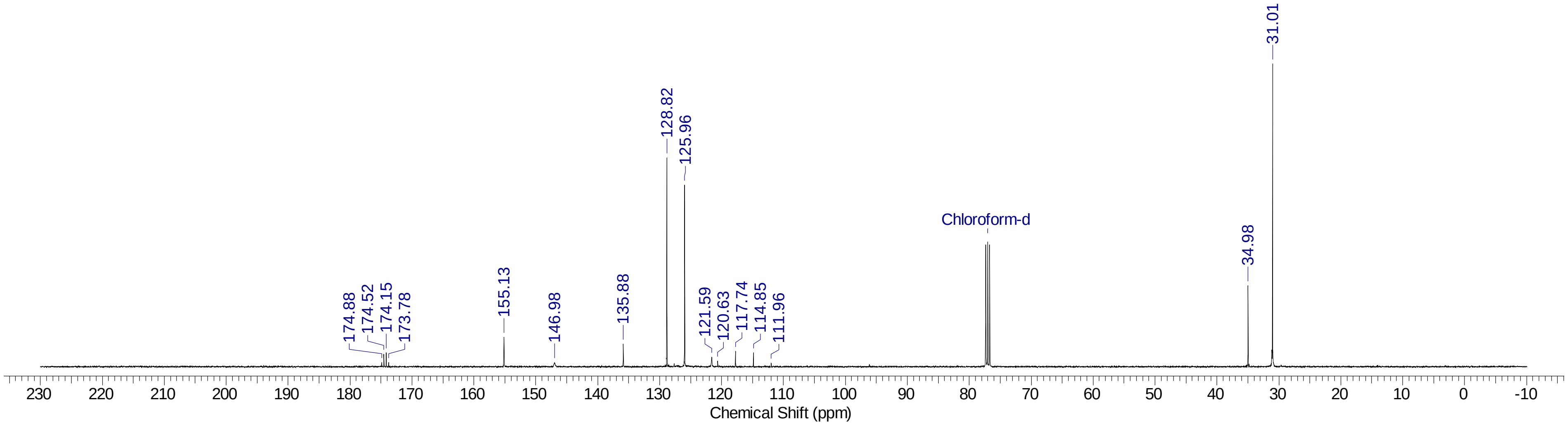

$^{13}C$ NMR spectrum of **2h** (100.6 MHz, $CDCl_3$)

3 May 2022

| | | | | | | | |
|---|---|---|---|---|---|---|---|
| ***Acquisition Time (sec)*** | 4.0894 | ***Comment*** | Imported from UXNMR. | | | ***Date*** | 15 Feb 2022 15:22:14 |
| ***File Name*** | C:\DOCS\OUTPUT_301\2022\02.翦 怵嚾黒BM-2362-3.H_001001r | | | | | ***Frequency (MHz)*** | 400.13 |
| ***Nucleus*** | 1H | ***Number of Transients*** | 4 | ***Original Points Count*** | 32768 | ***Points Count*** | 131072 |
| ***Pulse Sequence*** | zg30 | ***Solvent*** | DEUTERIUM OXIDE | | | ***Sweep Width (Hz)*** | 8012.82 |
| ***Temperature (degree C)*** | 27.000 | | | | | | |

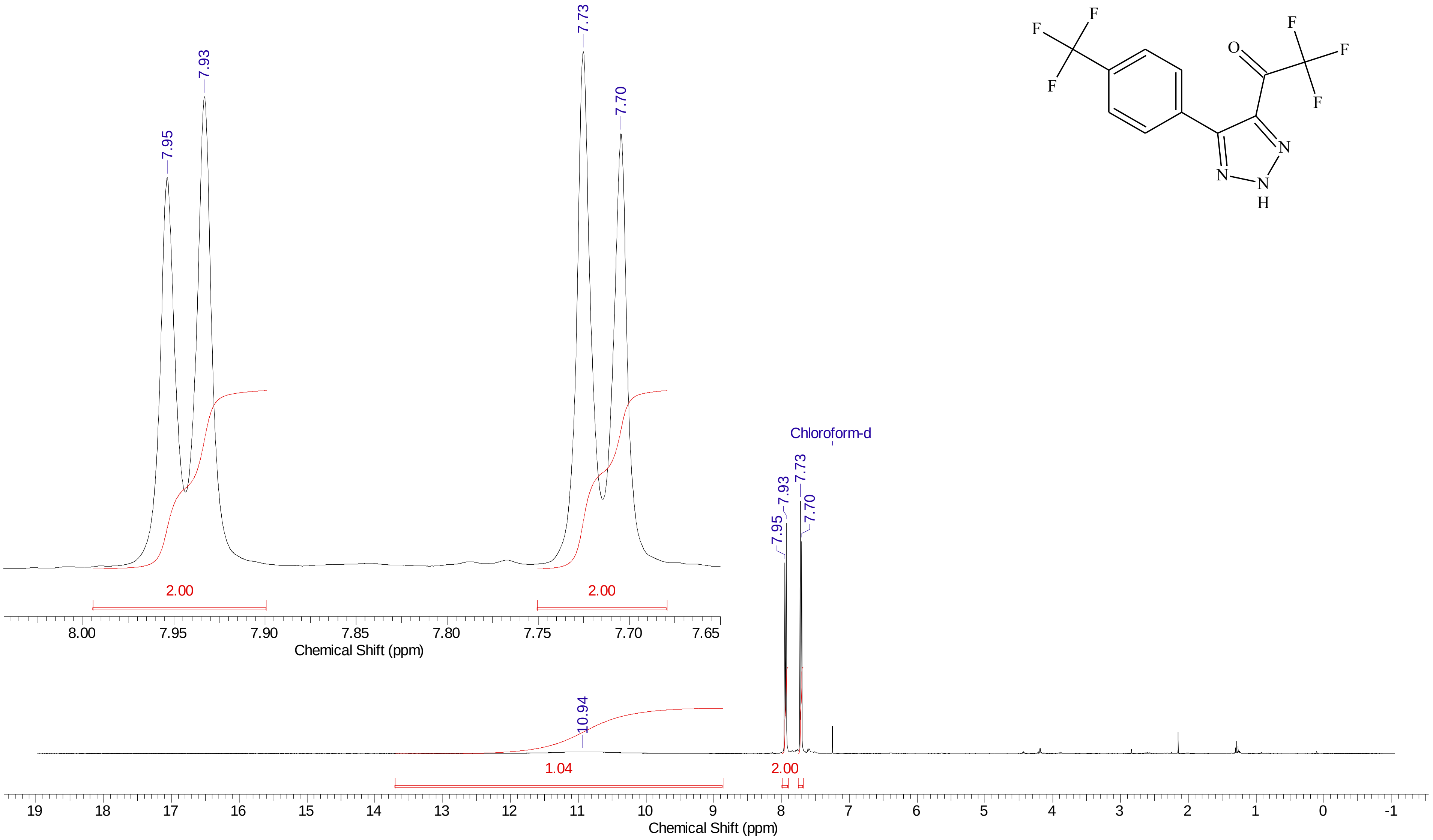

## $^{1}$H NMR spectrum of **2i** (400.1 MHz, $CDCl_3$)

3 May 2022

| | | | | | | | |
|---|---|---|---|---|---|---|---|
| ***Acquisition Time (sec)*** | 1.7433 | ***Comment*** | Imported from UXNMR. | | | ***Date*** | 15 Feb 2022 15:41:38 |
| ***File Name*** | C:\DOCS\OUTPUT_301\2022\02.翦 怵嚁黒BM-2362-2.F_005001r | | | | | ***Frequency (MHz)*** | 376.50 |
| ***Nucleus*** | 19F | ***Number of Transients*** | 16 | ***Original Points Count*** | 131072 | ***Points Count*** | 262144 |
| ***Pulse Sequence*** | zgflqn | ***Solvent*** | CHLOROFORM-D | | | ***Sweep Width (Hz)*** | 75187.97 |
| ***Temperature (degree C)*** | 27.000 | | | | | | |

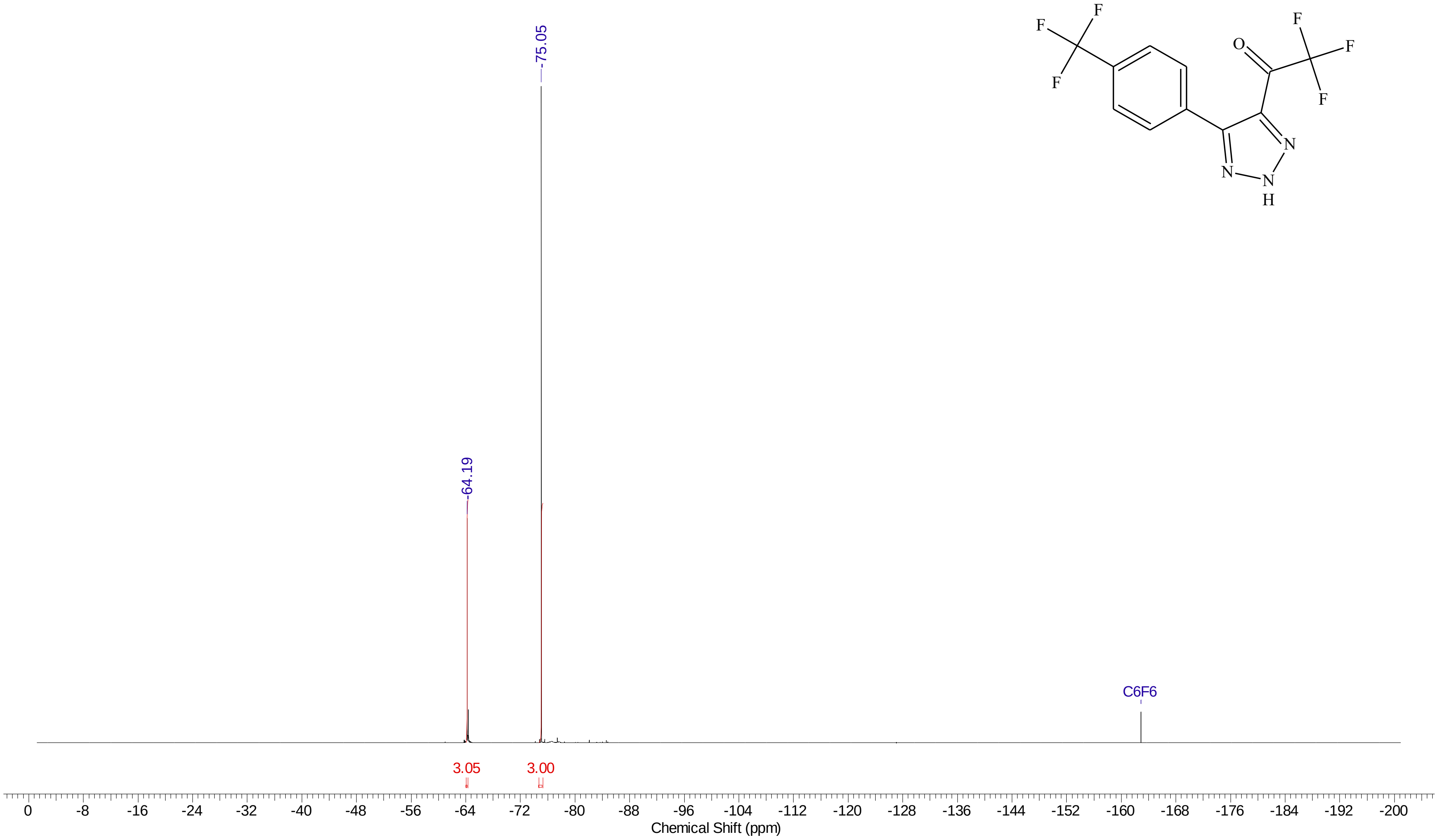

# $^{19}$F NMR spectrum of **2i** (376.5 MHz, $CDCl_3$)

3 May 2022

| | | | | | | | |
|---|---|---|---|---|---|---|---|
| ***Acquisition Time (sec)*** | 0.6783 | ***Comment*** | Imported from UXNMR. | | | ***Date*** | 15 Feb 2022 15:26:58 |
| ***File Name*** | C:\DOCS\OUTPUT_301\2022\02.翦 怺嚯黒BM-2362-3.C_002001r | | | | | ***Frequency (MHz)*** | 100.61 |
| ***Nucleus*** | 13C | ***Number of Transients*** | 105 | ***Original Points Count*** | 16384 | ***Points Count*** | 131072 |
| ***Pulse Sequence*** | zgpg30 | ***Solvent*** | ACETONITRILE-D3 | | | ***Sweep Width (Hz)*** | 24154.59 |
| ***Temperature (degree C)*** | 27.000 | | | | | | |

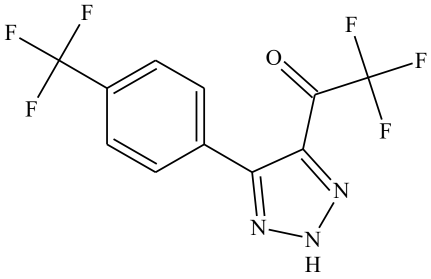


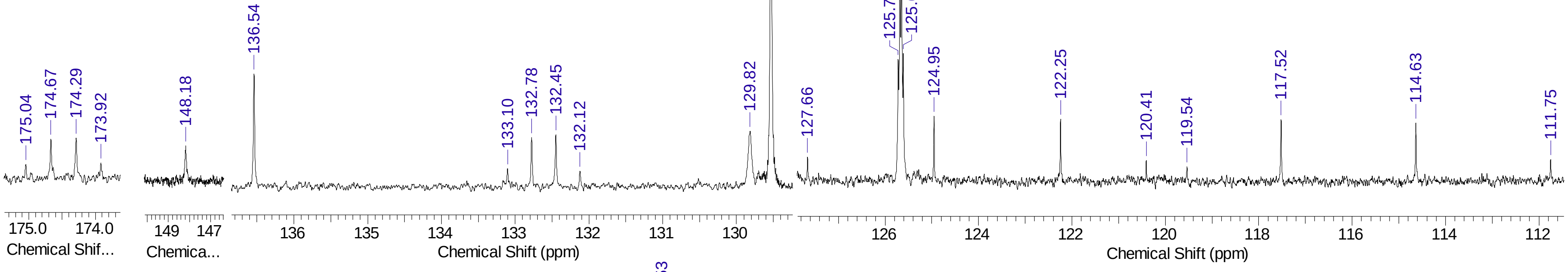


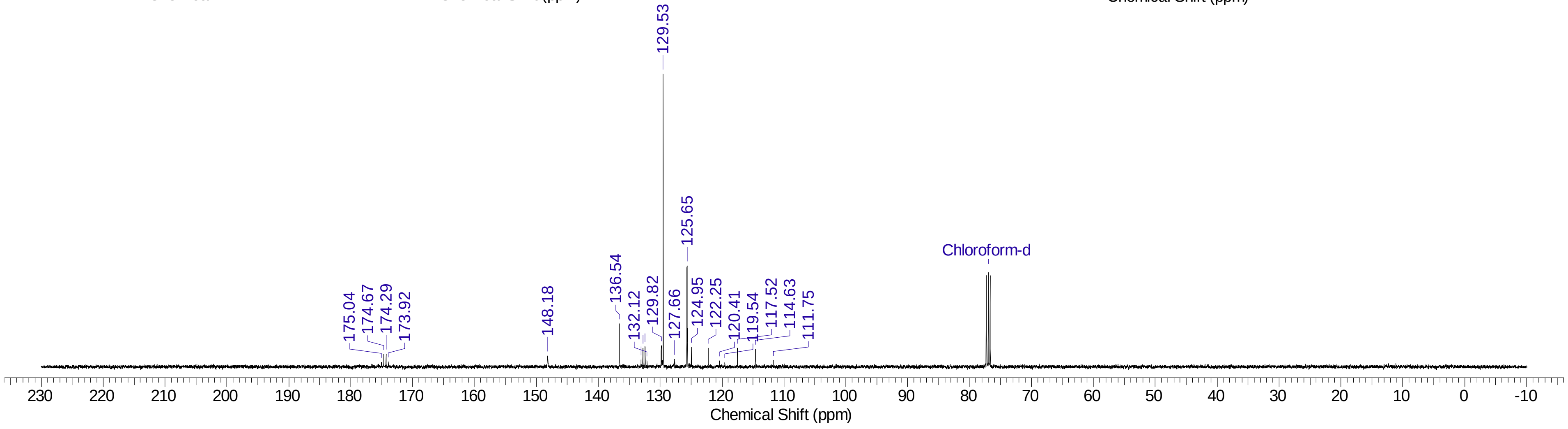

## $^{13}$C NMR spectrum of **2i** (100.6 MHz, $CDCl_3$)

3 May 2022

| | | | | | | | |
|---|---|---|---|---|---|---|---|
| ***Acquisition Time (sec)*** | 4.0894 | ***Comment*** | Imported from UXNMR. | | | ***Date*** | 16 Feb 2022 17:12:58 |
| ***File Name*** | C:\DOCS\OUTPUT_301\2022\02.翦 忕嚯黒BM-2364-3.H_001001r | | | | | ***Frequency (MHz)*** | 400.13 |
| ***Nucleus*** | 1H | ***Number of Transients*** | 4 | ***Original Points Count*** | 32768 | ***Points Count*** | 131072 |
| ***Pulse Sequence*** | zg30 | ***Solvent*** | DEUTERIUM OXIDE | | | ***Sweep Width (Hz)*** | 8012.82 |
| ***Temperature (degree C)*** | 27.000 | | | | | | |

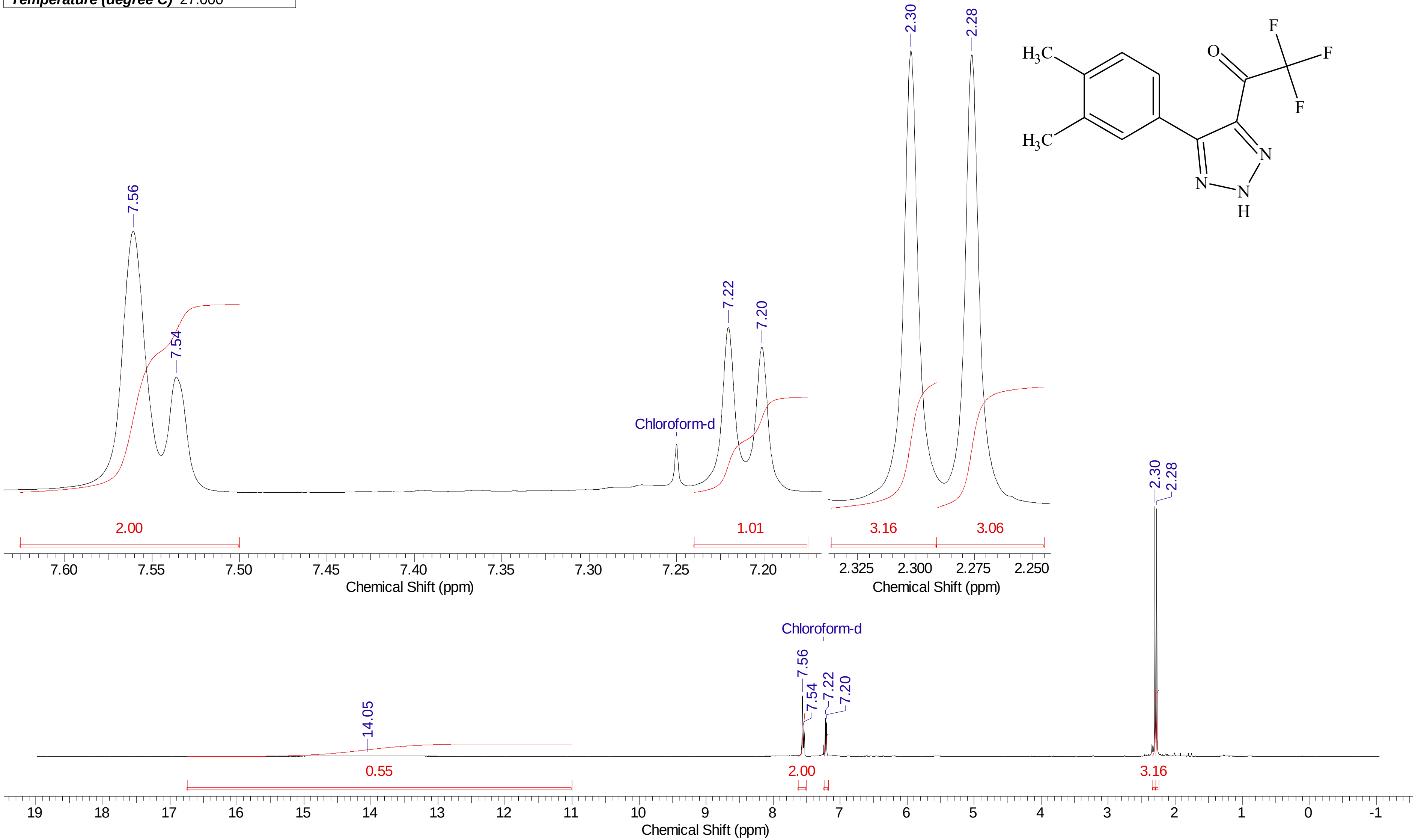

# $^{1}$H NMR spectrum of **2j** (400.1 MHz, $CDCl_3$)

3 May 2022

| | | | | | |
|---|---|---|---|---|---|
| ***Acquisition Time (sec)*** | 1.7433 | ***Comment*** | Imported from UXNMR. | ***Date*** | 16 Feb 2022 17:48:22 |
| ***File Name*** | C:\DOCS\OUTPUT_301\2022\02.翦 怵嚯黒BM-2364-3.F_005001r | | | ***Frequency (MHz)*** | 376.50 |
| ***Nucleus*** | 19F | ***Number of Transients*** | 16 | ***Original Points Count*** | 131072 |
| | | | | ***Points Count*** | 262144 |
| ***Pulse Sequence*** | zgflqn | ***Solvent*** | CHLOROFORM-D | ***Sweep Width (Hz)*** | 75187.97 |
| ***Temperature (degree C)*** | 27.000 | | | | |

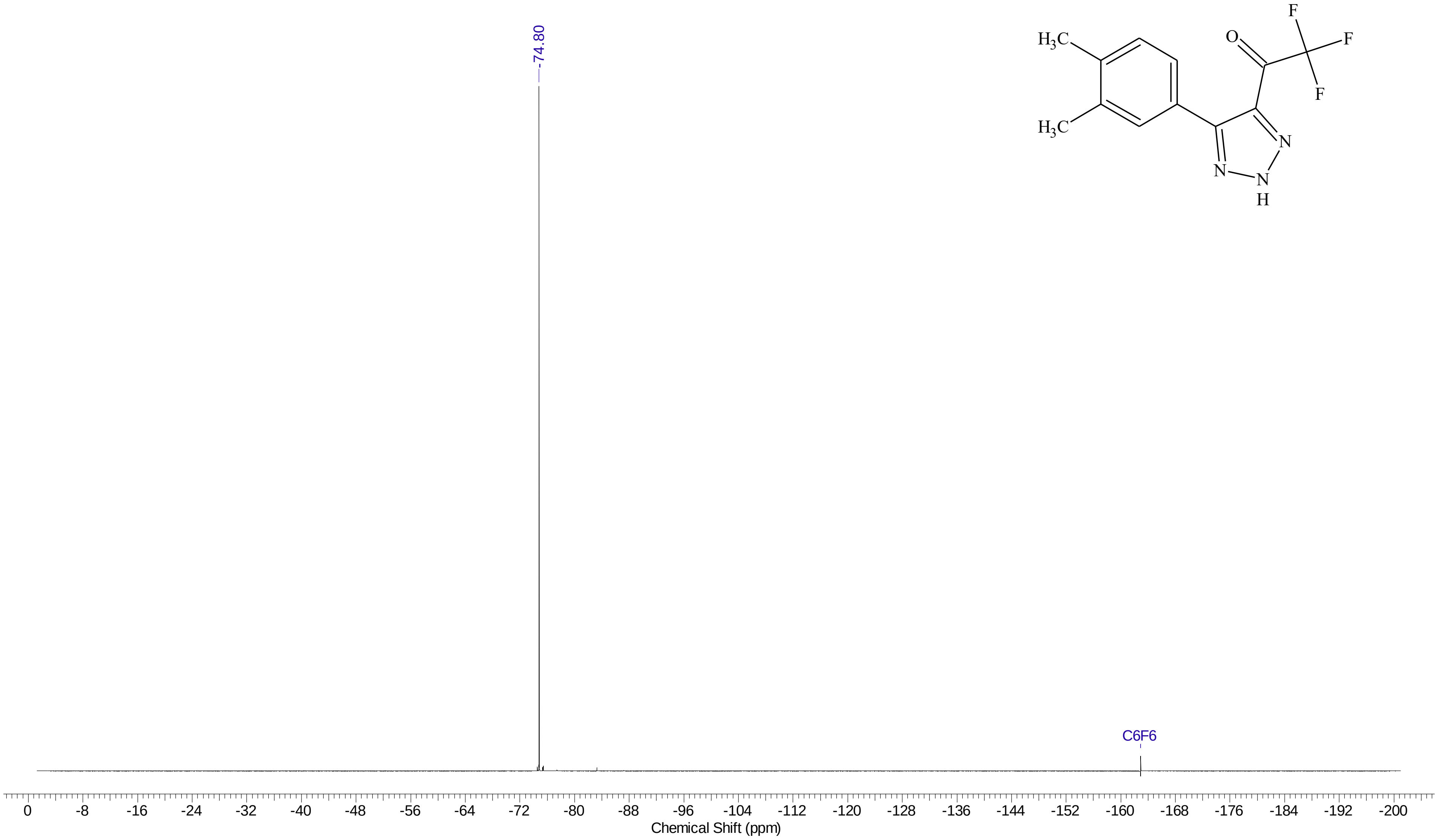

## $^{19}$F NMR spectrum of **2j** (376.5 MHz, $CDCl_3$)

3 May 2022

| | | | | | | | |
|---|---|---|---|---|---|---|---|
| ***Acquisition Time (sec)*** | 0.6783 | ***Comment*** | Imported from UXNMR. | | | ***Date*** | 16 Feb 2022 17:33:44 |
| ***File Name*** | C:\DOCS\OUTPUT_301\2022\02.翦 怺嚯黒BM-2364-3.C_002001r | | | | | ***Frequency (MHz)*** | 100.61 |
| ***Nucleus*** | 13C | ***Number of Transients*** | 489 | ***Original Points Count*** | 16384 | ***Points Count*** | 131072 |
| ***Pulse Sequence*** | zgpg30 | ***Solvent*** | ACETONITRILE-D3 | | | ***Sweep Width (Hz)*** | 24154.59 |
| ***Temperature (degree C)*** | 27.000 | | | | | | |

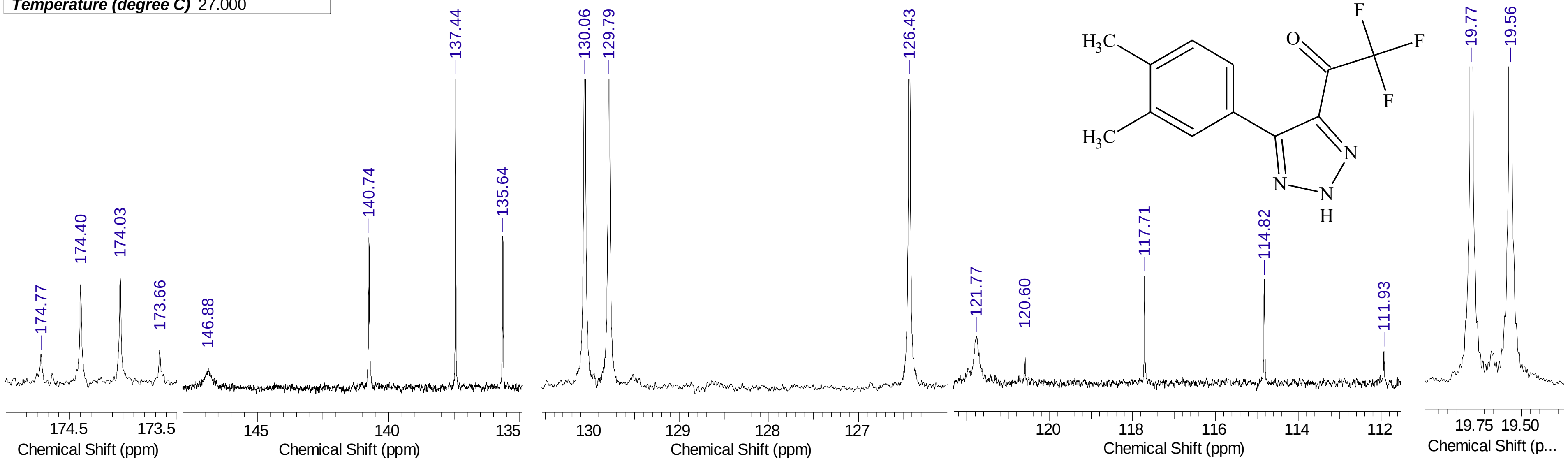


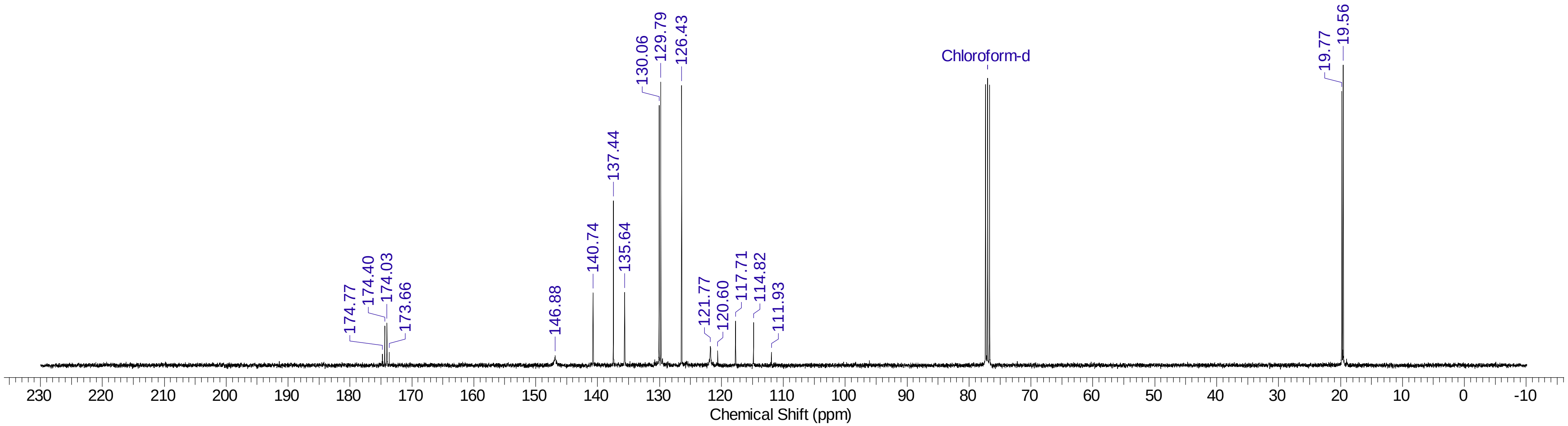

$^{13}$C NMR spectrum of **2j** (100.6 MHz, $CDCl_3$)

13 May 2022

| | | | | | | | |
|---|---|---|---|---|---|---|---|
| ***Acquisition Time (sec)*** | 4.0894 | ***Comment*** | Imported from UXNMR. | | | ***Date*** | 02 Mar 2022 17:24:00 |
| ***File Name*** | C:\DOCS\OUTPUT_301\2022\03.爨 痱\SZA-BM-2359p.H_001001r | | | | | ***Frequency (MHz)*** | 400.13 |
| ***Nucleus*** | 1H | ***Number of Transients*** | 4 | ***Original Points Count*** | 32768 | ***Points Count*** | 131072 |
| ***Pulse Sequence*** | zg30 | ***Solvent*** | CHLOROFORM-D | | | ***Sweep Width (Hz)*** | 8012.82 |
| ***Temperature (degree C)*** | 27.000 | | | | | | |

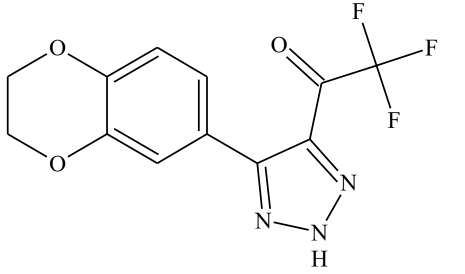

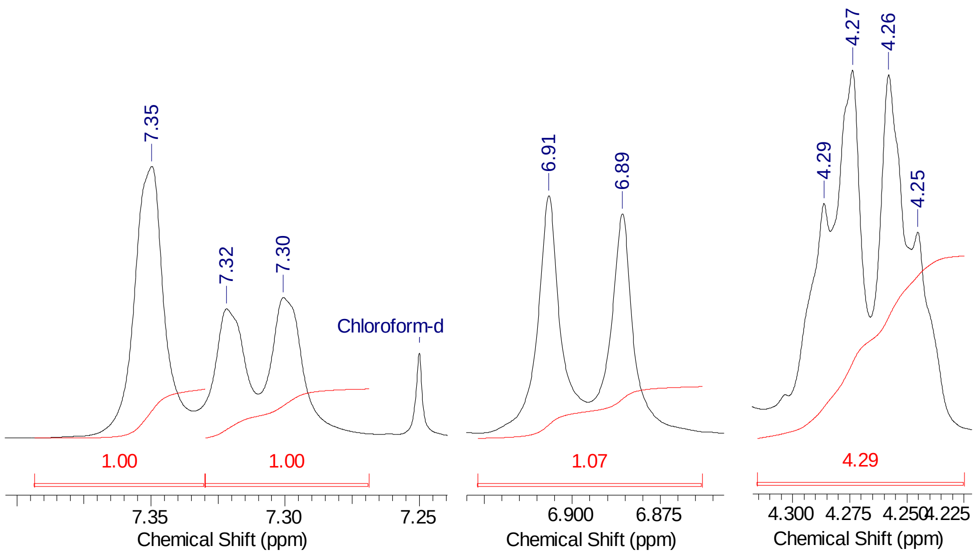


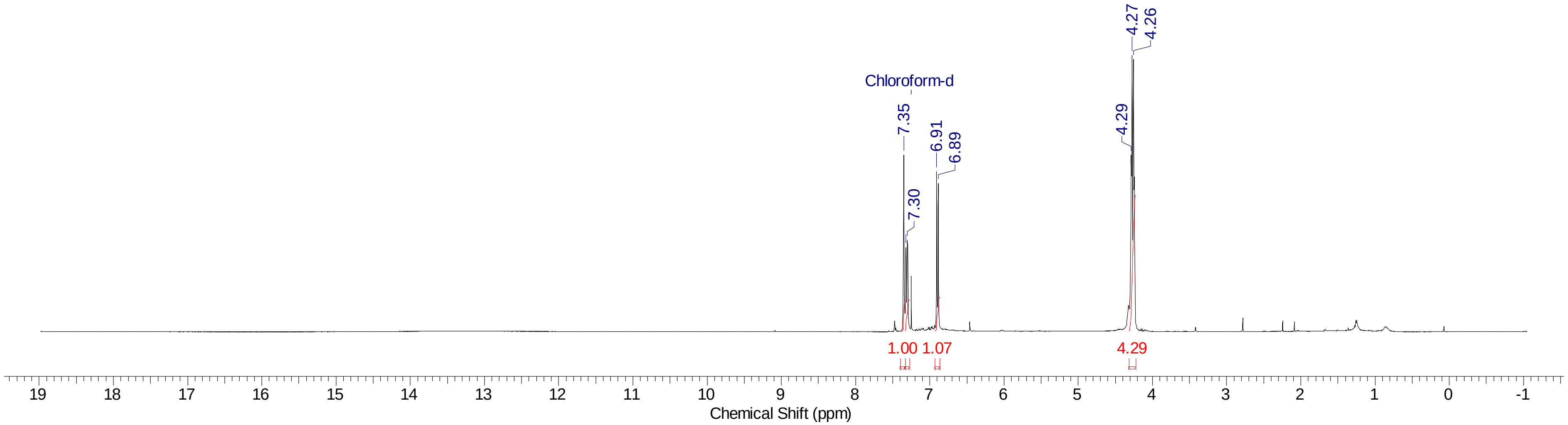

$^{1}$H NMR spectrum of **2k** (400.1 MHz, $CDCl_3$)

13 May 2022

| | | | | | | | |
|---|---|---|---|---|---|---|---|
| ***Acquisition Time (sec)*** | 1.7433 | ***Comment*** | Imported from UXNMR. | | ***Date*** | 01 Mar 2022 15:32:36 | |
| ***File Name*** | H:\吖 桒01.03.2022\SZA-BM-2359.F_005001r | | ***Frequency (MHz)*** | 376.50 | ***Nucleus*** | 19F | ***Number of Transients*** 4 |
| ***Original Points Count*** | 131072 | ***Points Count*** | 262144 | ***Pulse Sequence*** zgflqn | ***Solvent*** | CHLOROFORM-D | |
| ***Sweep Width (Hz)*** | 75187.97 | ***Temperature (degree C)*** | 27.000 | | | | |

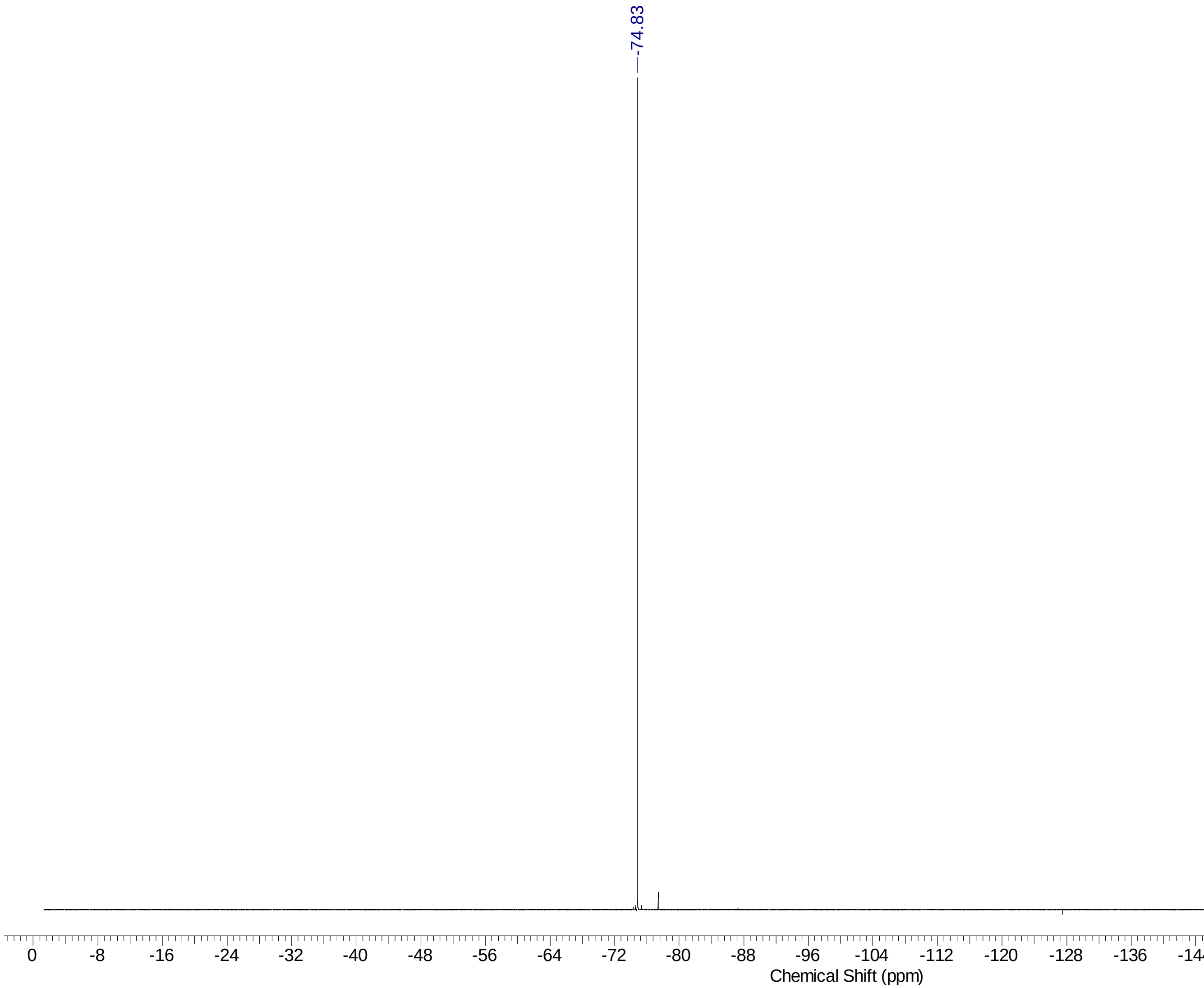


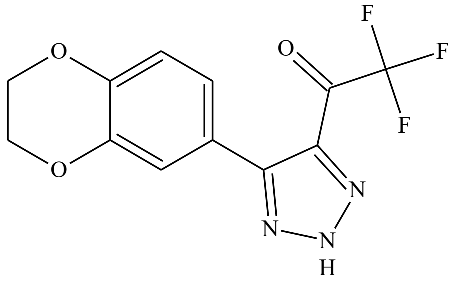

# $^{19}$F NMR spectrum of **2k** (376.5 MHz, $CDCl_3$)

13 May 2022

| | | | | | | | |
|---|---|---|---|---|---|---|---|
| ***Acquisition Time (sec)*** | 0.6783 | ***Comment*** | Imported from UXNMR. | | | ***Date*** | 02 Mar 2022 17:42:18 |
| ***File Name*** | C:\DOCS\OUTPUT_301\2022\03.嬰 痱\SZA-BM-2359p.C_002001r | | | | | ***Frequency (MHz)*** | 100.61 |
| ***Nucleus*** | 13C | ***Number of Transients*** | 417 | ***Original Points Count*** | 16384 | ***Points Count*** | 131072 |
| ***Pulse Sequence*** | zgpg30 | ***Solvent*** | ACETONITRILE-D3 | | | ***Sweep Width (Hz)*** | 24154.59 |
| ***Temperature (degree C)*** | 27.000 | | | | | | |

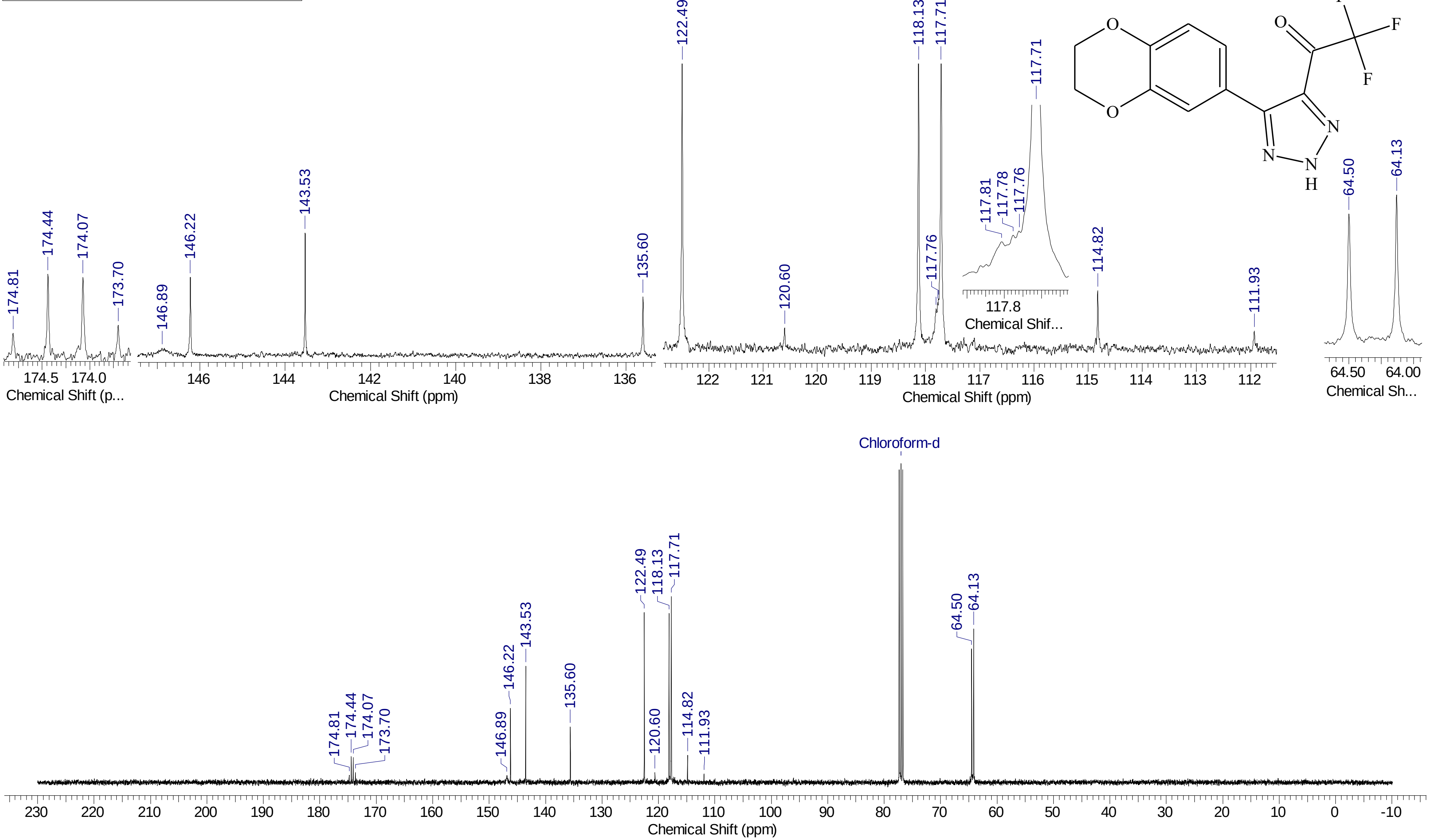

## $^{13}$C NMR spectrum of **2k** (100.6 MHz, $CDCl_3$)

13 May 2022

| | | | | | | | |
|---|---|---|---|---|---|---|---|
| ***Acquisition Time (sec)*** | 4.0894 | ***Comment*** | Imported from UXNMR. | | | ***Date*** | 14 Feb 2022 15:40:12 |
| ***File Name*** | C:\DOCS\OUTPUT_301\2022\02.翦 怵嚯黒BM-2358-3.H_001001r | | | | | ***Frequency (MHz)*** | 400.13 |
| ***Nucleus*** | 1H | ***Number of Transients*** | 4 | ***Original Points Count*** | 32768 | ***Points Count*** | 131072 |
| ***Pulse Sequence*** | zg30 | ***Solvent*** | DMSO-D6 | ***Sweep Width (Hz)*** | 8012.82 | ***Temperature (degree C)*** | 27.000 |

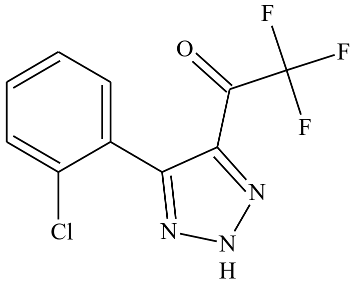


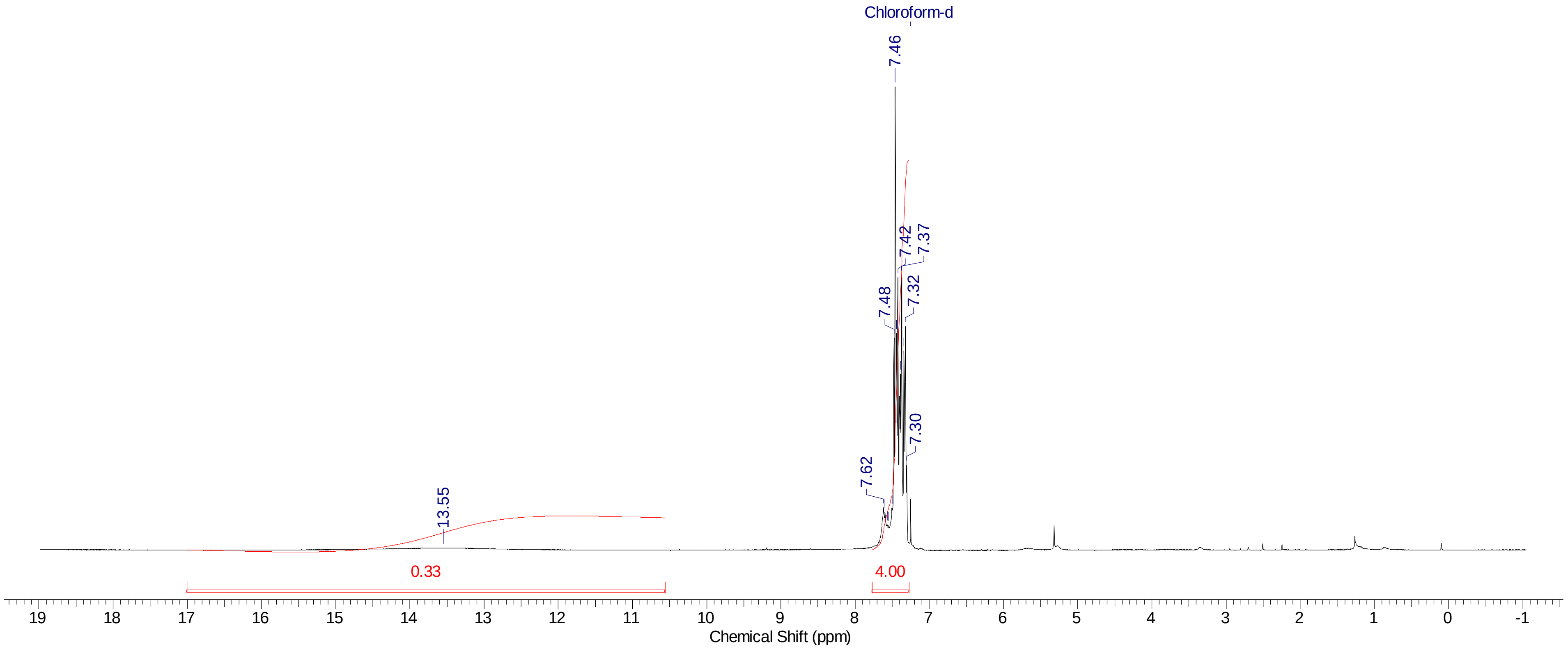

$^{1}$H NMR spectrum of **2l** (400.1 MHz, $CDCl_3$)

13 May 2022

| | | | | | | | |
|---|---|---|---|---|---|---|---|
| ***Acquisition Time (sec)*** | 1.7433 | ***Comment*** | Imported from UXNMR. | | | ***Date*** | 12 Feb 2022 13:39:46 |
| ***File Name*** | C:\DOCS\BM\咛 峪盐 雅 娜 \2022\bm220212\BM-2358-3_005001r | | | | | ***Frequency (MHz)*** | 376.50 |
| ***Nucleus*** | 19F | ***Number of Transients*** | 16 | ***Original Points Count*** | 131072 | ***Points Count*** | 262144 |
| ***Pulse Sequence*** | zgflqn | ***Solvent*** | CHLOROFORM-D | | | ***Sweep Width (Hz)*** | 75187.97 |
| ***Temperature (degree C)*** | 27.000 | | | | | | |

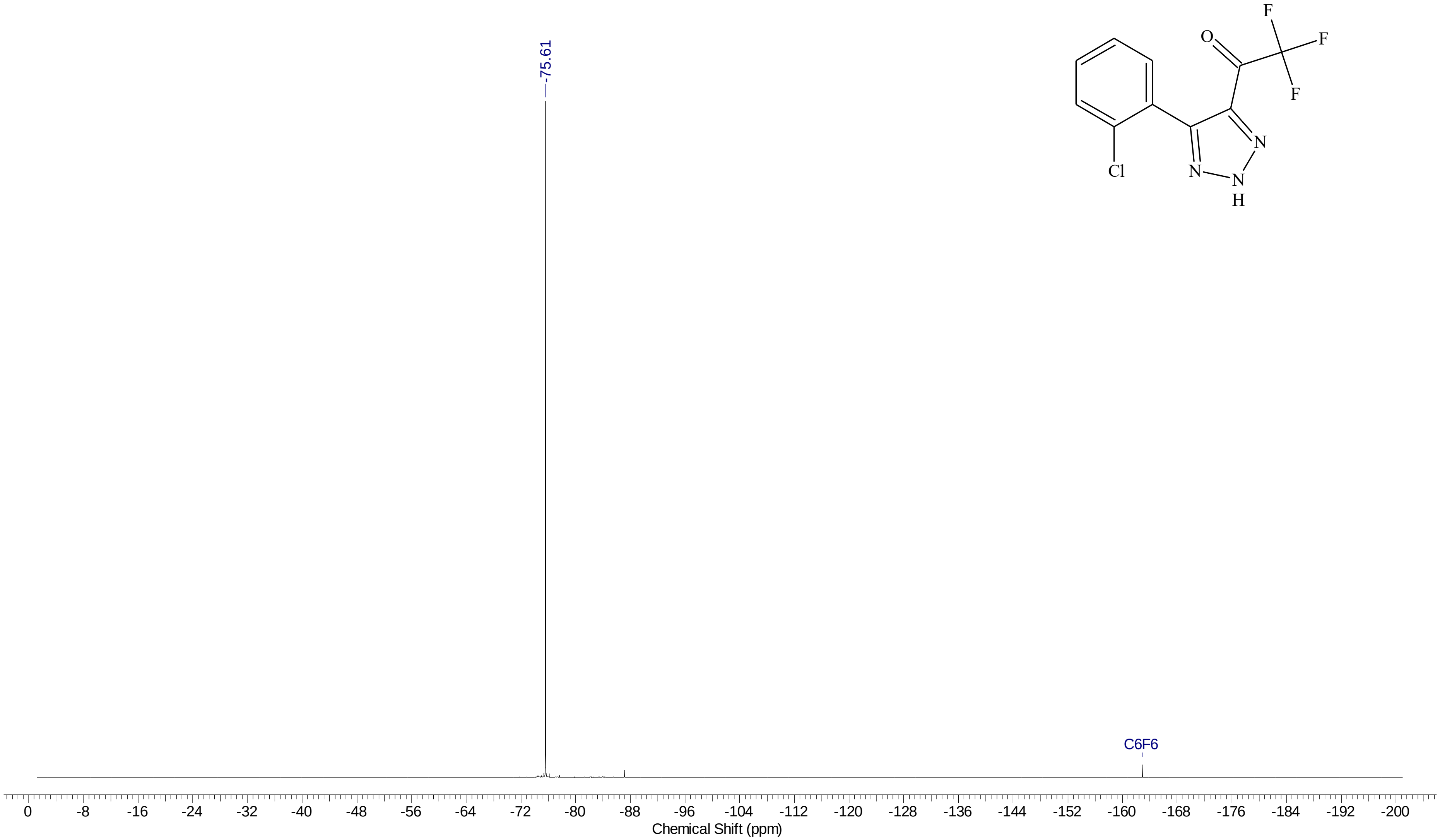

$^{19}$F NMR spectrum of **2l** (376.5 MHz, $CDCl_3$)

13 May 2022

| | | | | | | | |
|---|---|---|---|---|---|---|---|
| ***Acquisition Time (sec)*** | 0.6783 | ***Comment*** | Imported from UXNMR. | | | ***Date*** | 14 Feb 2022 15:37:38 |
| ***File Name*** | C:\DOCS\OUTPUT_301\2022\02.翦 怺嚁熏BM-2358-3.C_002001r | | | | | ***Frequency (MHz)*** | 100.61 |
| ***Nucleus*** | 13C | ***Number of Transients*** | 129 | ***Original Points Count*** | 16384 | ***Points Count*** | 131072 |
| ***Pulse Sequence*** | zgpg30 | ***Solvent*** | ACETONITRILE-D3 | | | ***Sweep Width (Hz)*** | 24154.59 |
| ***Temperature (degree C)*** | 27.000 | | | | | | |

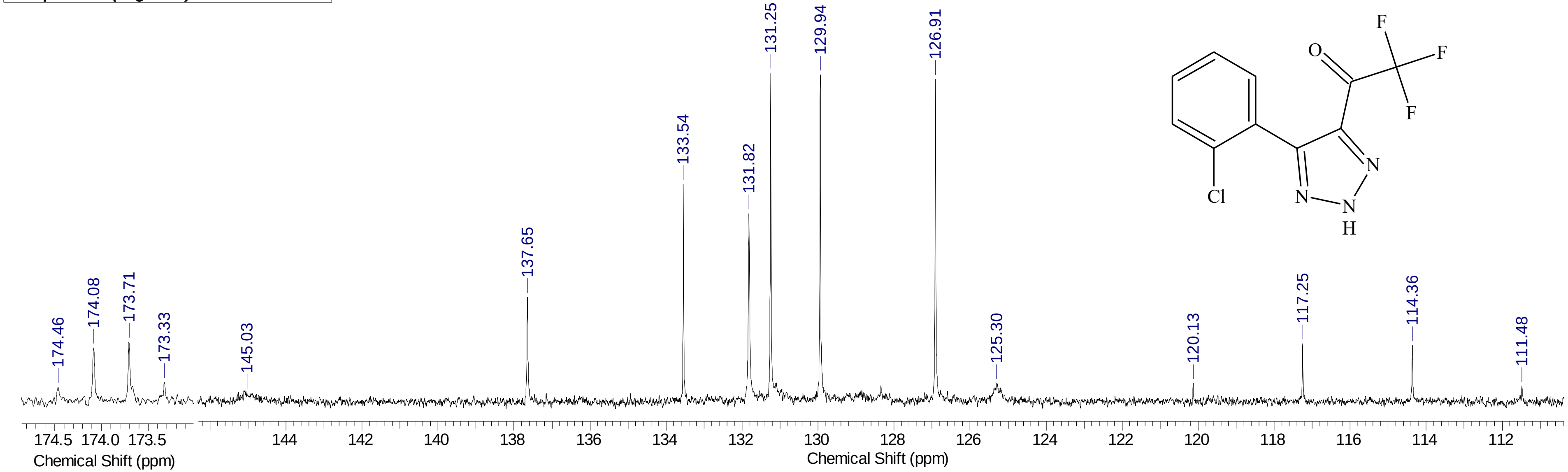


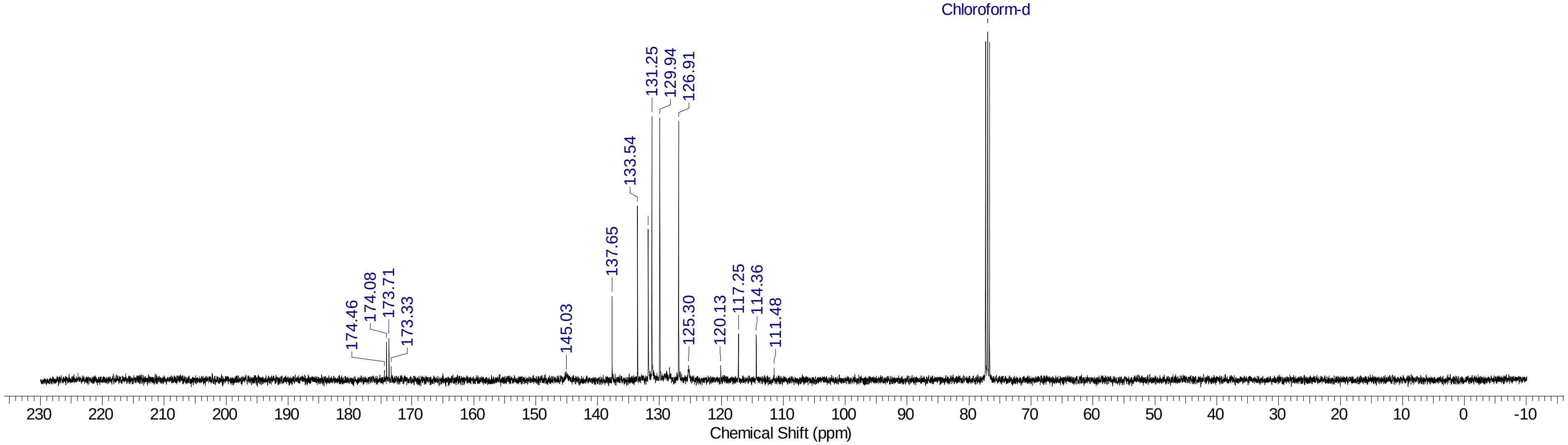

## $^{13}$C NMR spectrum of **2l** (100.6 MHz, $CDCl_3$)

2 Aug 2022

| | | | | | | | |
|---|---|---|---|---|---|---|---|
| ***Acquisition Time (sec)*** | 4.0894 | ***Comment*** | Imported from UXNMR. | | | ***Date*** | 07 Jun 2022 15:29:24 |
| ***File Name*** | C:\DOCS\OUTPUT_301\2022\06.棹 晙\BM-2509-NEW.H_001001r | | | | | ***Frequency (MHz)*** | 400.13 |
| ***Nucleus*** | 1H | ***Number of Transients*** | 8 | ***Original Points Count*** | 32768 | ***Points Count*** | 131072 |
| ***Pulse Sequence*** | zg30 | ***Solvent*** | CHLOROFORM-D | | | ***Sweep Width (Hz)*** | 8012.82 |
| ***Temperature (degree C)*** | 27.000 | | | | | | |

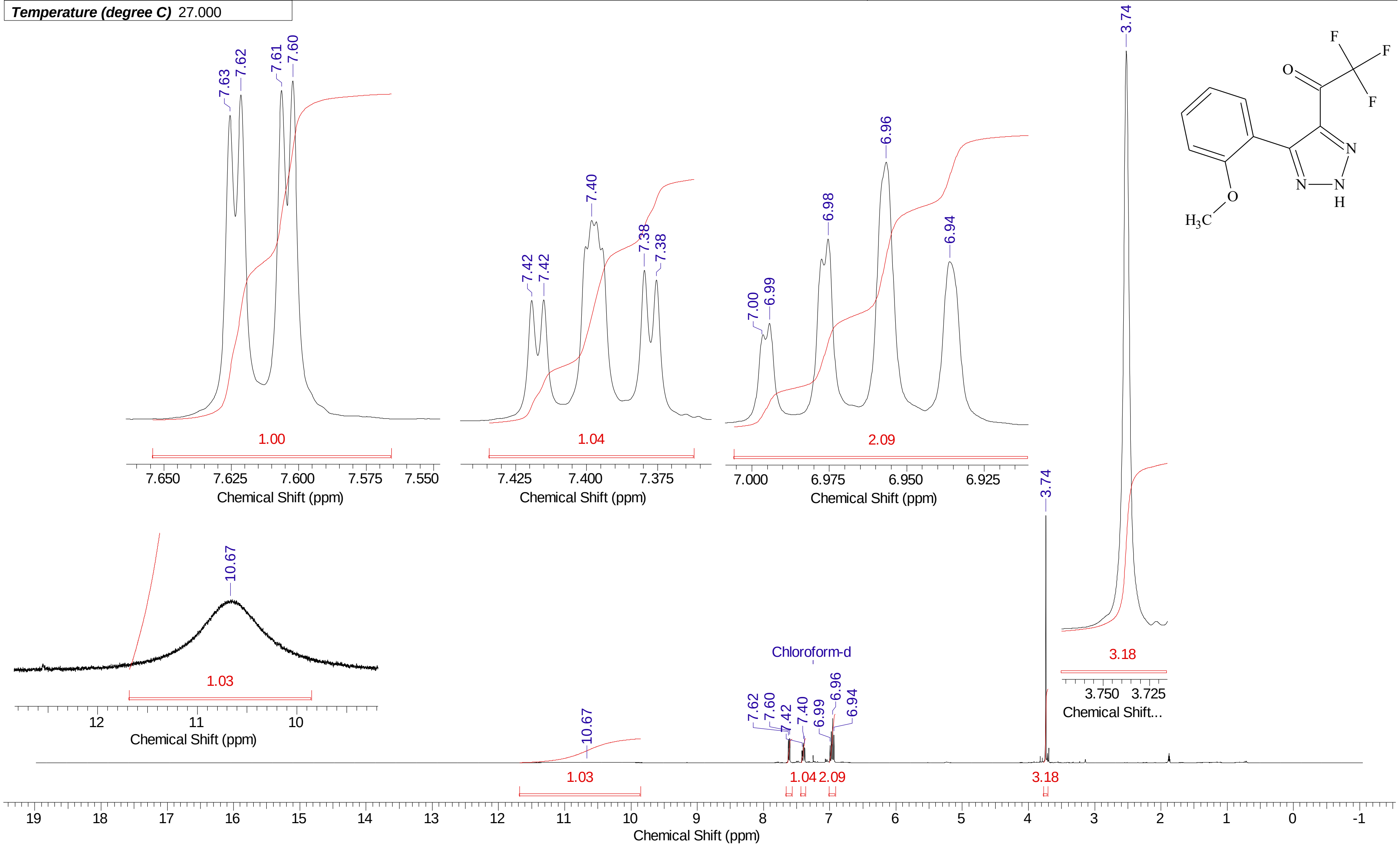

# $^{1}H$ NMR spectrum of **2m** (400.1 MHz, $CDCl_3$)

2 Aug 2022

| | | | | | |
|---|---|---|---|---|---|
| ***Acquisition Time (sec)*** | 1.7433 | ***Comment*** | Imported from UXNMR. | ***Date*** | 07 Jun 2022 15:26:58 |
| ***File Name*** | C:\DOCS\OUTPUT_301\2022\06.棹 晙\BM-2509-NEW.F_005001r | | | ***Frequency (MHz)*** | 376.50 |
| ***Nucleus*** | 19F | ***Number of Transients*** | 16 ***Original Points Count*** 131072 | ***Points Count*** | 262144 |
| ***Pulse Sequence*** | zgflqn | ***Solvent*** | CHLOROFORM-D | ***Sweep Width (Hz)*** | 75187.97 |
| ***Temperature (degree C)*** | 27.000 | | | | |

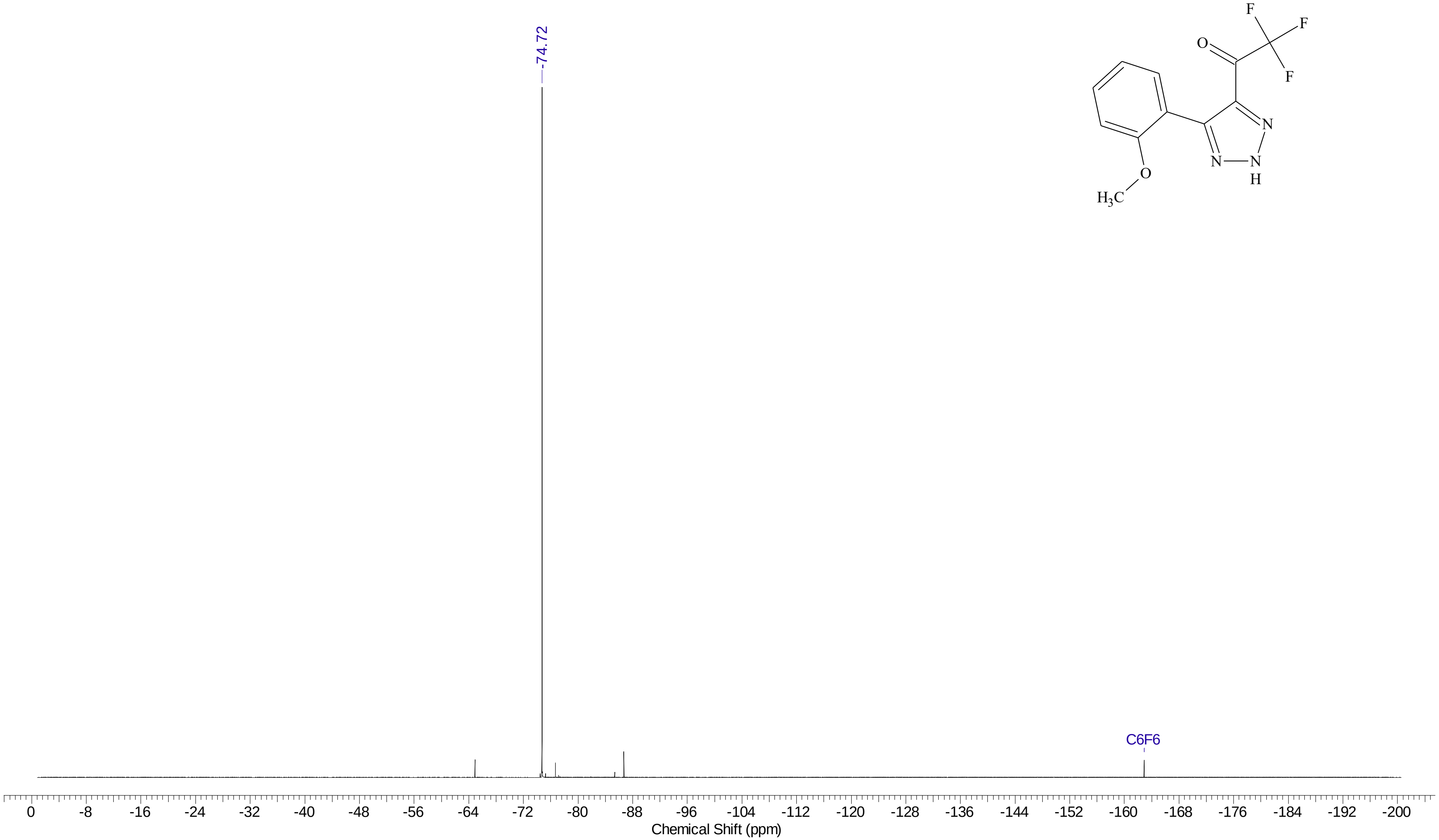

## $^{19}$F NMR spectrum of **2m** (376.5 MHz, $CDCl_3$)

2 Aug 2022

| | | | | | | | |
|---|---|---|---|---|---|---|---|
| **Acquisition Time (sec)** | 0.6783 | **Comment** | Imported from UXNMR. | | | **Date** | 07 Jun 2022 21:30:54 |
| **File Name** | C:\DOCS\OUTPUT_301\2022\06.棹 晙\BM-2509-NEW\BM-2509-NEW_002001r | | | | | **Frequency (MHz)** | 100.61 |
| **Nucleus** | 13C | **Number of Transients** | 256 | **Original Points Count** | 16384 | **Points Count** | 131072 |
| **Pulse Sequence** | zgpg30 | **Solvent** | CHLOROFORM-D | | | **Sweep Width (Hz)** | 24154.59 |
| **Temperature (degree C)** | 27.000 | | | | | | |

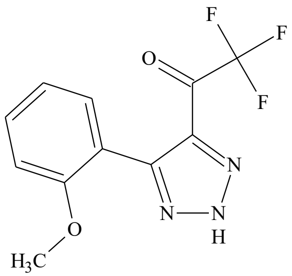


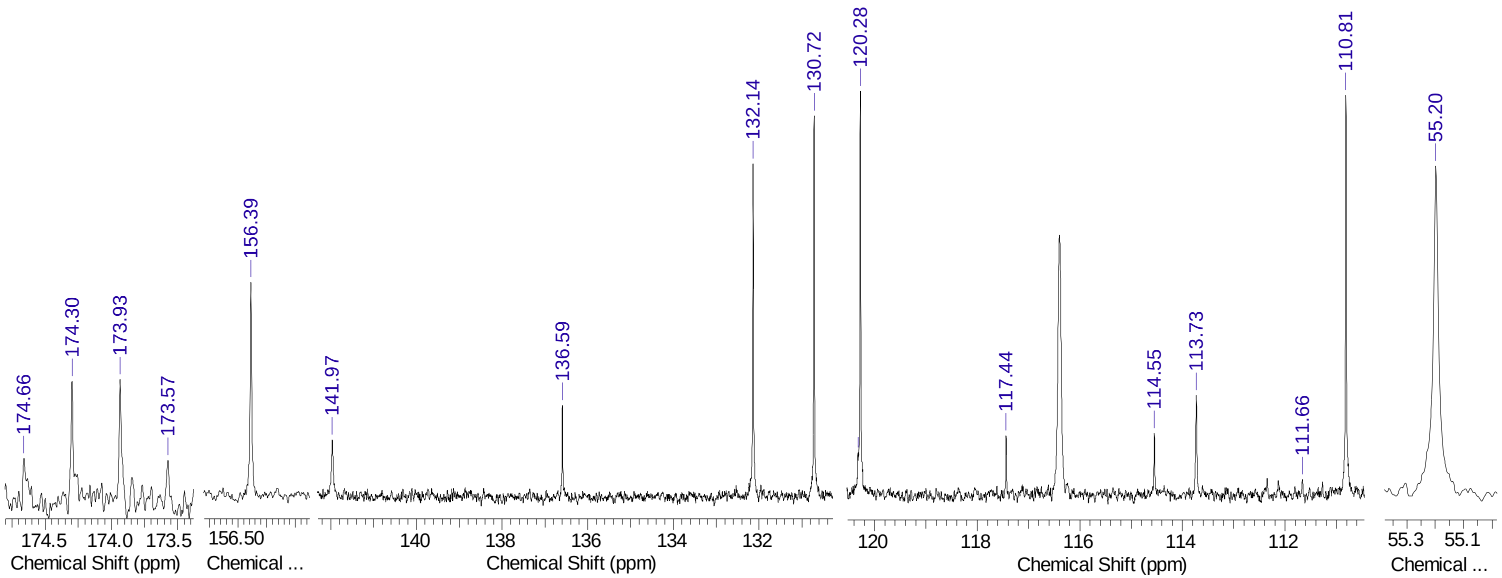


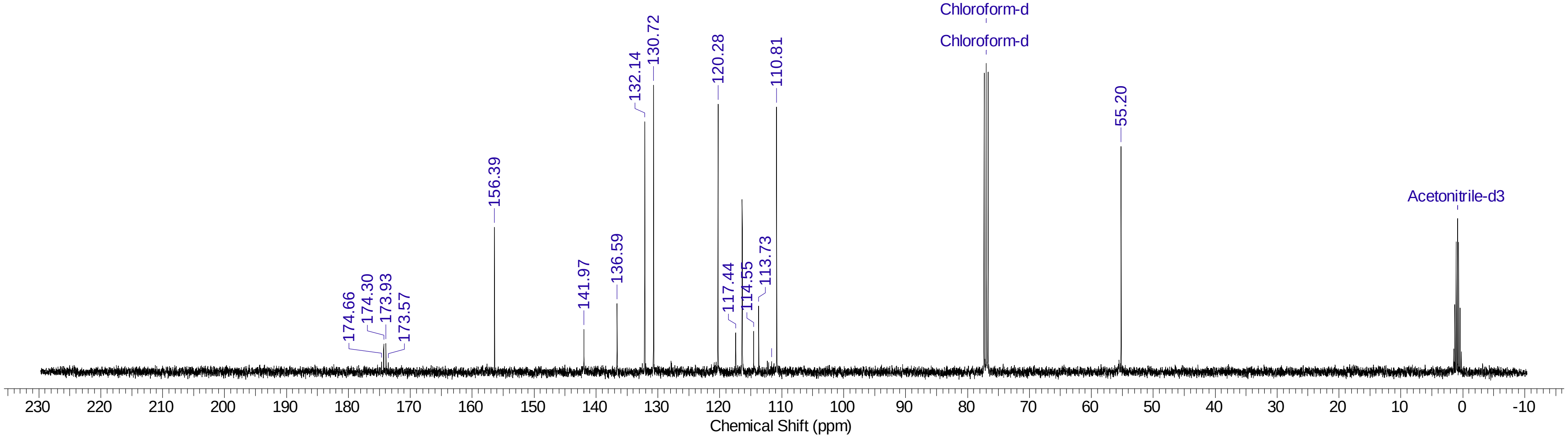

$^{13}$C NMR spectrum of **2m** (100.6 MHz, $CDCl_3$)

13 May 2022

| | | | | | | | |
|---|---|---|---|---|---|---|---|
| ***Acquisition Time (sec)*** | 4.0894 | ***Comment*** | Imported from UXNMR. | | | ***Date*** | 03 Mar 2022 13:40:30 |
| ***File Name*** | C:\DOCS\OUTPUT_301\2022\03.爨 鼐\SZA-BM-2361-h_001001r | | | | | ***Frequency (MHz)*** | 400.13 |
| ***Nucleus*** | 1H | ***Number of Transients*** | 4 | ***Original Points Count*** | 32768 | ***Points Count*** | 131072 |
| ***Pulse Sequence*** | zg30 | ***Solvent*** | CHLOROFORM-D | | | ***Sweep Width (Hz)*** | 8012.82 |
| ***Temperature (degree C)*** | 27.000 | | | | | | |

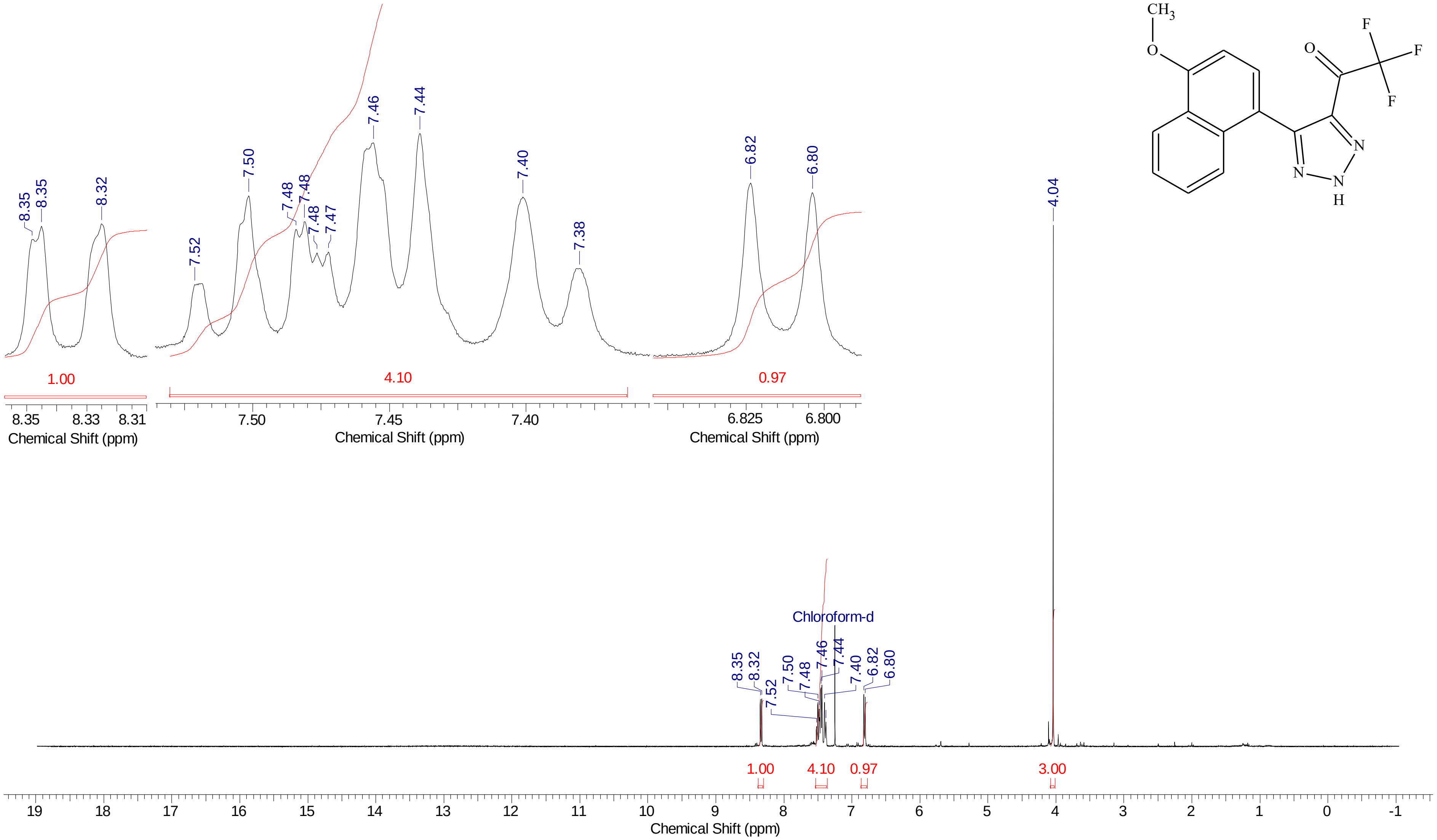

# $^{1}$H NMR spectrum of **2n** (400.1 MHz, $CDCl_3$)

13 May 2022

| | | | | | | | |
|---|---|---|---|---|---|---|---|
| ***Acquisition Time (sec)*** | 1.7433 | ***Comment*** | Imported from UXNMR. | | | ***Date*** | 04 Mar 2022 15:30:46 |
| ***File Name*** | C:\DOCS\OUTPUT_301\2022\03.爨 痱\SZA-BM-2361.F_005001r | | | | | ***Frequency (MHz)*** | 376.50 |
| ***Nucleus*** | 19F | ***Number of Transients*** | 4 | ***Original Points Count*** | 131072 | ***Points Count*** | 262144 |
| ***Pulse Sequence*** | zgflqn | ***Solvent*** | CHLOROFORM-D | | | ***Sweep Width (Hz)*** | 75187.97 |
| ***Temperature (degree C)*** | 27.000 | | | | | | |

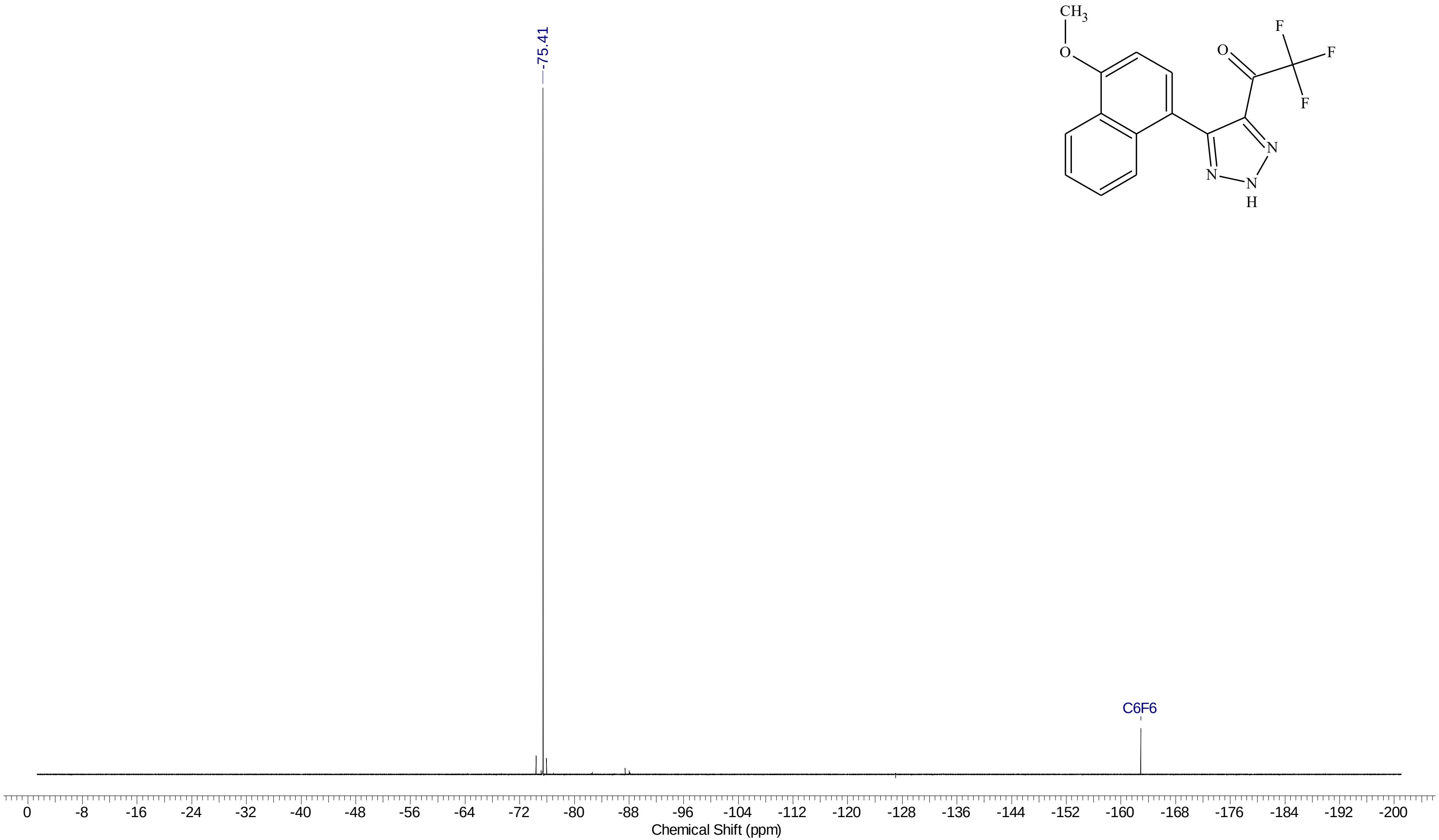

## $^{19}$F NMR spectrum of **2n** (376.5 MHz, $CDCl_3$)

13 May 2022

| | | | | | | | |
|---|---|---|---|---|---|---|---|
| ***Acquisition Time (sec)*** | 0.6783 | ***Comment*** | Imported from UXNMR. | | | ***Date*** | 10 Mar 2022 14:52:34 |
| ***File Name*** | C:\DOCS\OUTPUT_301\2022\03.爨 痱\SZA-BM-2361.C_002001r | | | | | ***Frequency (MHz)*** | 100.61 |
| ***Nucleus*** | 13C | ***Number of Transients*** | 894 | ***Original Points Count*** | 16384 | ***Points Count*** | 131072 |
| ***Pulse Sequence*** | zgpg30 | ***Solvent*** | DMSO-D6 | ***Sweep Width (Hz)*** | 24154.59 | ***Temperature (degree C)*** | 27.000 |

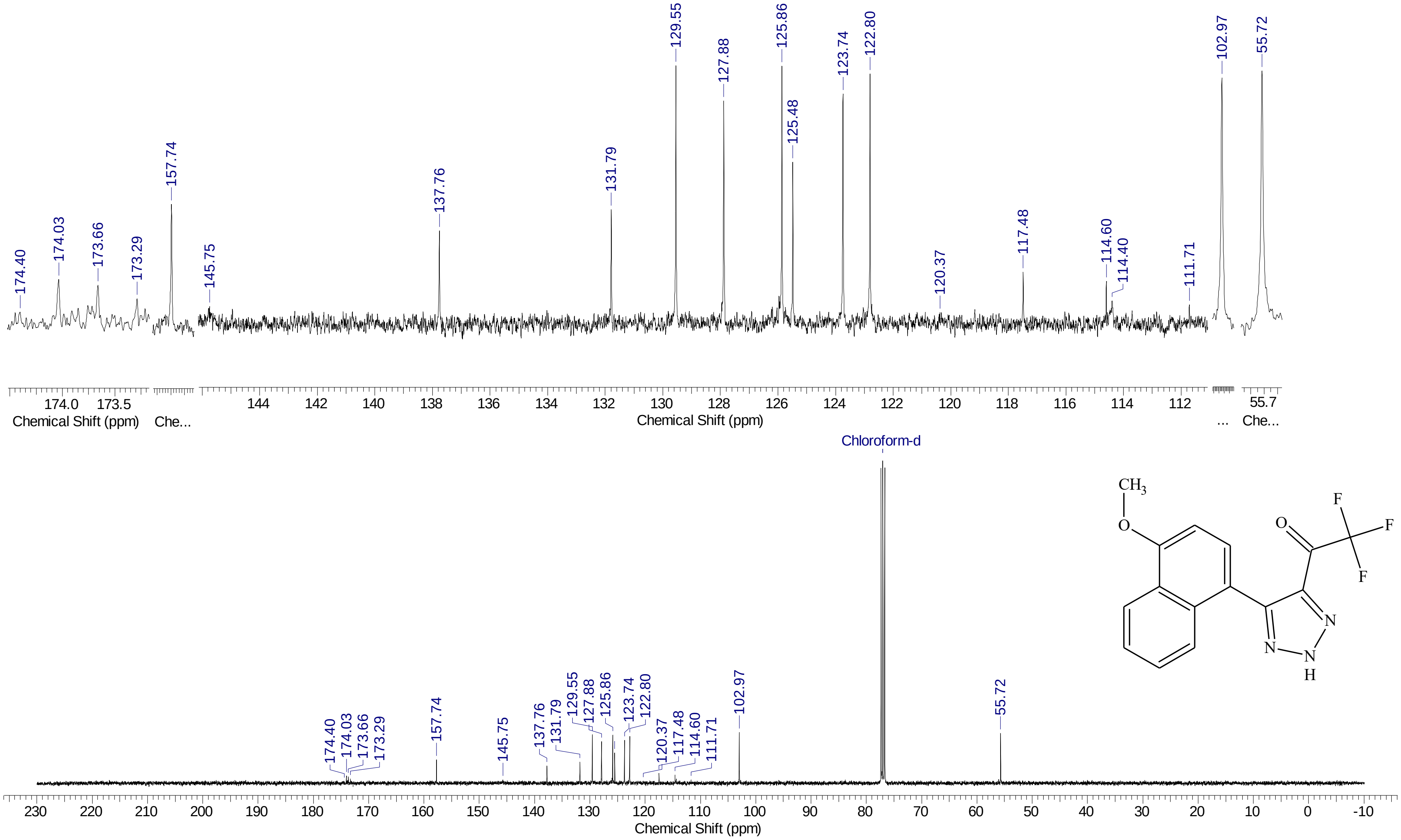

## $^{13}$C NMR spectrum of **2n** (100.6 MHz, $CDCl_3$)

13 May 2022

| | | | | | |
|---|---|---|---|---|---|
| ***Acquisition Time (sec)*** | 4.0894 | ***Comment*** | Imported from UXNMR. | ***Date*** | 03 Dec 2021 15:15:22 |
| ***File Name*** | C:\DOCS\OUTPUT_301\2021\12.溴赅狃黒BM-2356-3.H_001001r | | | ***Frequency (MHz)*** | 400.13 |
| ***Nucleus*** | 1H | ***Number of Transients*** | 4 ***Original Points Count*** 32768 | ***Points Count*** | 131072 |
| ***Pulse Sequence*** | zg30 | ***Solvent*** | CHLOROFORM-D | ***Sweep Width (Hz)*** | 8012.82 |
| ***Temperature (degree C)*** | 27.000 | | | | |

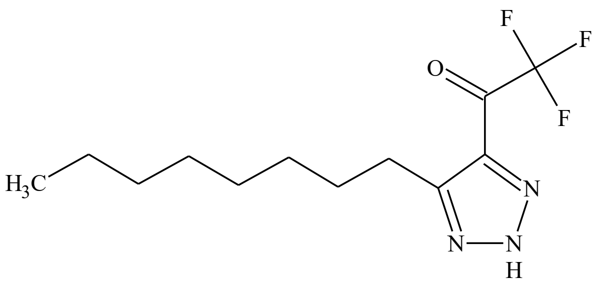


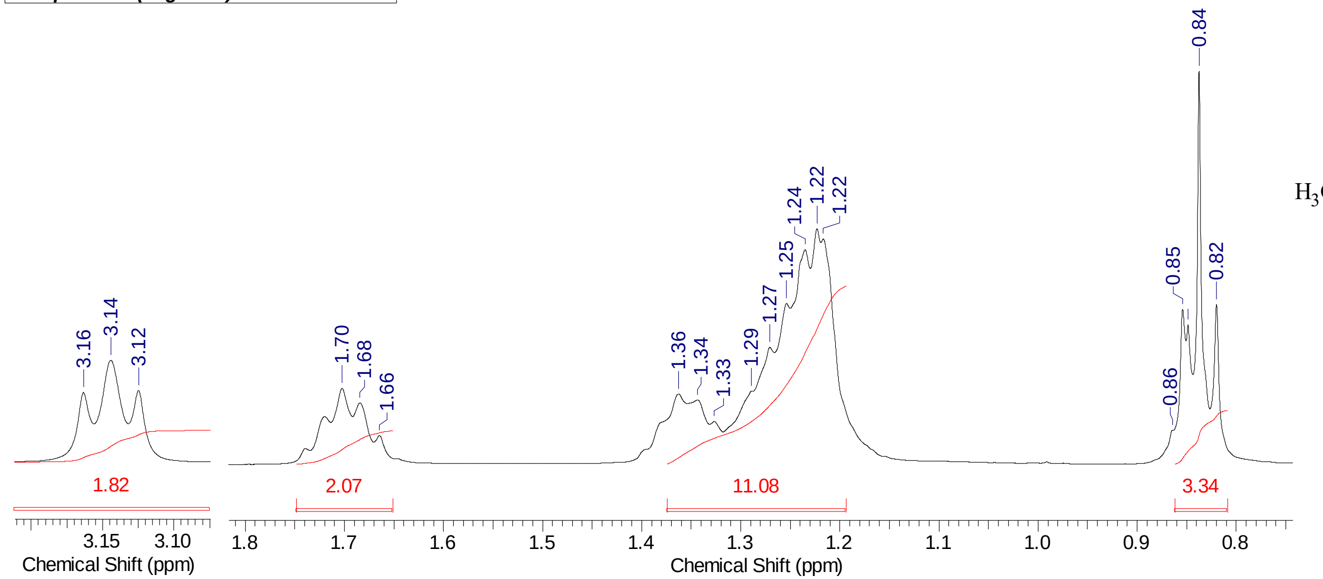


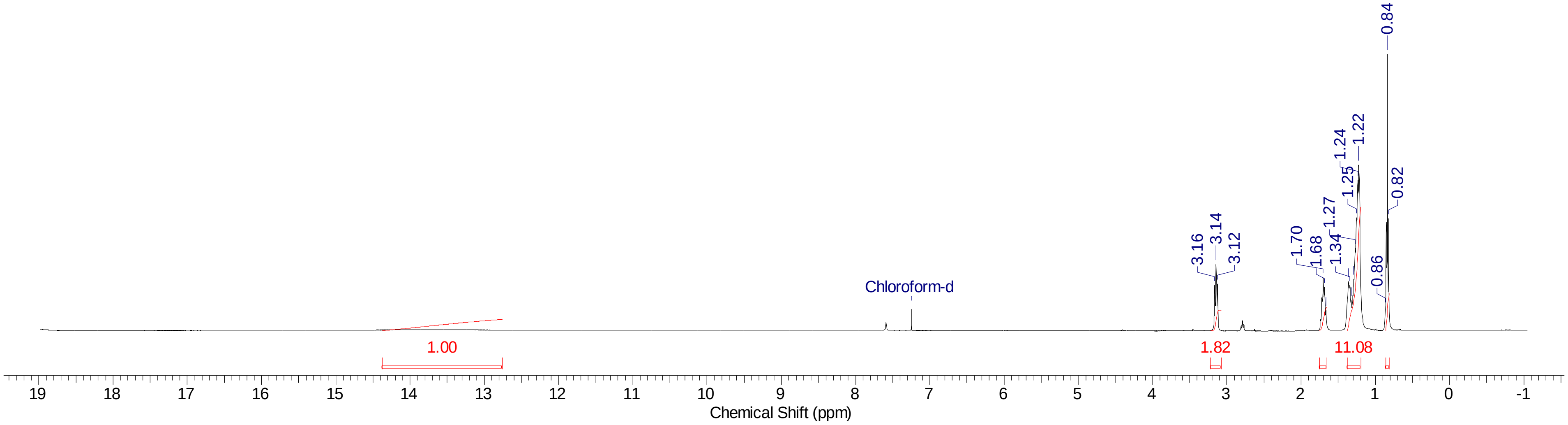

## $^{1}H$ NMR spectrum of **2o** (400.1 MHz, $CDCl_3$)

13 May 2022

| | | | | | | | |
|---|---|---|---|---|---|---|---|
| ***Acquisition Time (sec)*** | 1.7433 | ***Comment*** | Imported from UXNMR. | | | ***Date*** | 03 Dec 2021 15:41:32 |
| ***File Name*** | C:\DOCS\OUTPUT_301\2021\12.溴赅狃黒BM-2356-3.F_005001r | | | | | ***Frequency (MHz)*** | 376.50 |
| ***Nucleus*** | 19F | ***Number of Transients*** | 11 | ***Original Points Count*** | 131072 | ***Points Count*** | 262144 |
| ***Pulse Sequence*** | zgflqn | ***Solvent*** | CHLOROFORM-D | | | ***Sweep Width (Hz)*** | 75187.97 |
| ***Temperature (degree C)*** | 27.000 | | | | | | |

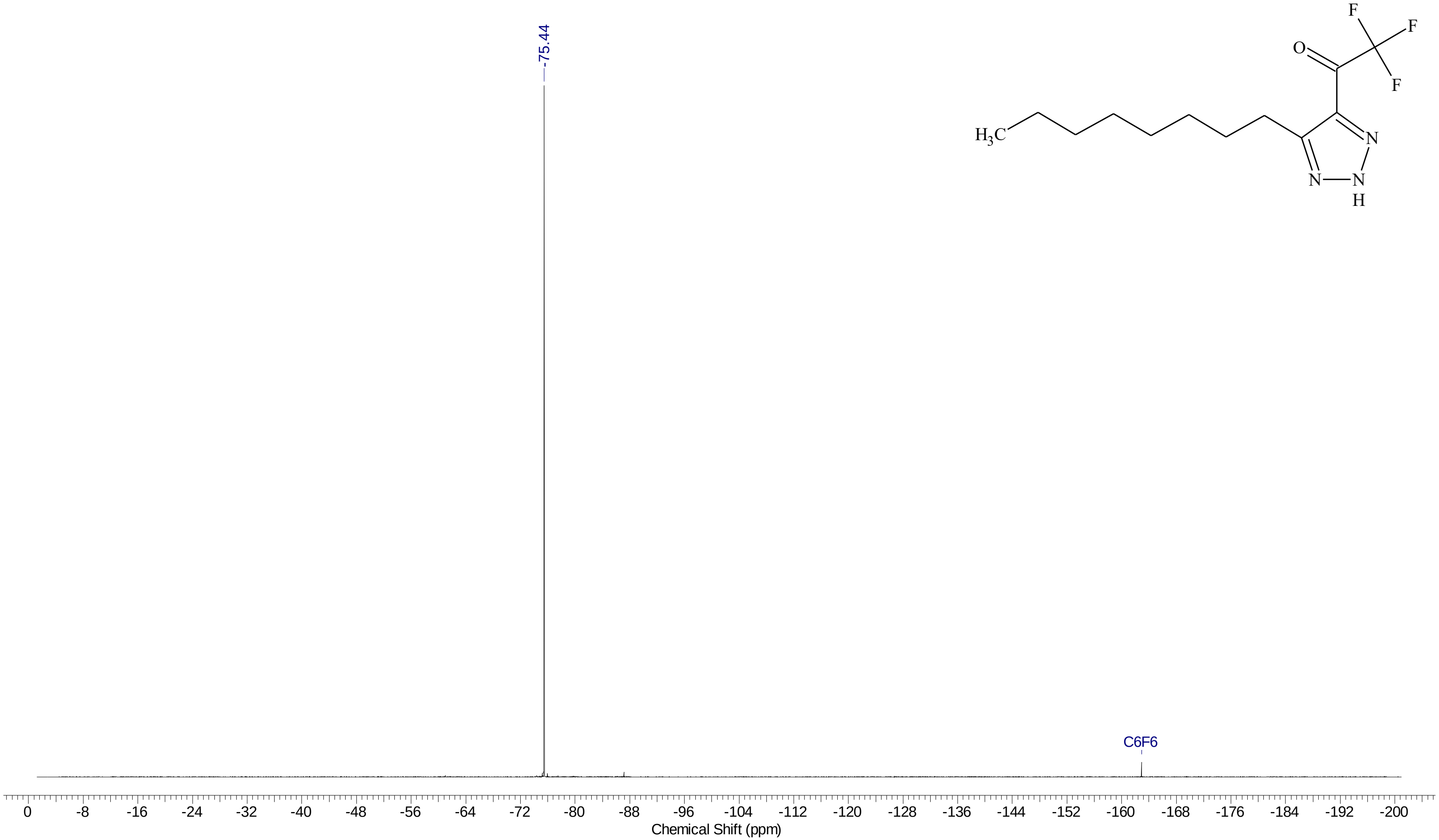

$^{19}$F NMR spectrum of **2o** (376.5 MHz, $CDCl_3$)

13 May 2022

| | | | | | | | |
|---|---|---|---|---|---|---|---|
| ***Acquisition Time (sec)*** | 0.6783 | ***Comment*** | Imported from UXNMR. | | | ***Date*** | 04 Dec 2021 13:47:24 |
| ***File Name*** | C:\DOCS\OUTPUT_301\2021\12.溴赅狃黑BM-2356-3.C_002001r | | | | | ***Frequency (MHz)*** | 100.61 |
| ***Nucleus*** | 13C | ***Number of Transients*** | 201 | ***Original Points Count*** | 16384 | ***Points Count*** | 131072 |
| ***Pulse Sequence*** | zgpg30 | ***Solvent*** | CHLOROFORM-D | | | ***Sweep Width (Hz)*** | 24154.59 |
| ***Temperature (degree C)*** | 27.000 | | | | | | |

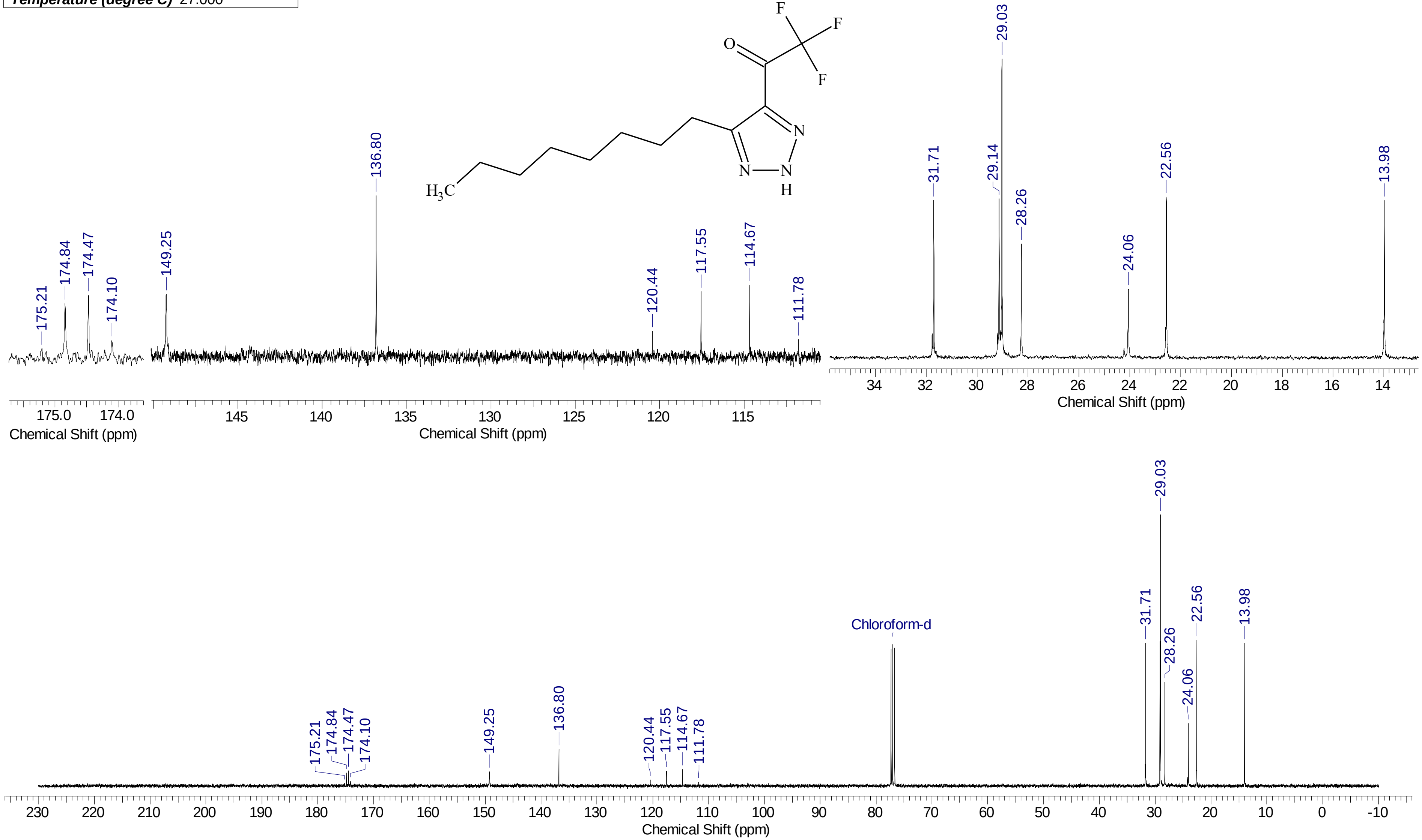

## $^{13}$C NMR spectrum of **2o** (100.6 MHz, $CDCl_3$)

3 Aug 2022

| | | | | | | | |
|---|---|---|---|---|---|---|---|
| ***Acquisition Time (sec)*** | 4.0894 | ***Comment*** | Imported from UXNMR. | | | ***Date*** | 30 Nov 2021 14:27:30 |
| ***File Name*** | C:\DOCS\OUTPUT_301\2021\11.眍 狃黒SZA-BM-2208.H_001001r | | | | | ***Frequency (MHz)*** | 400.13 |
| ***Nucleus*** | 1H | ***Number of Transients*** | 4 | ***Original Points Count*** | 32768 | ***Points Count*** | 131072 |
| ***Pulse Sequence*** | zg30 | ***Solvent*** | CHLOROFORM-D | | | ***Sweep Width (Hz)*** | 8012.82 |
| ***Temperature (degree C)*** | 27.000 | | | | | | |

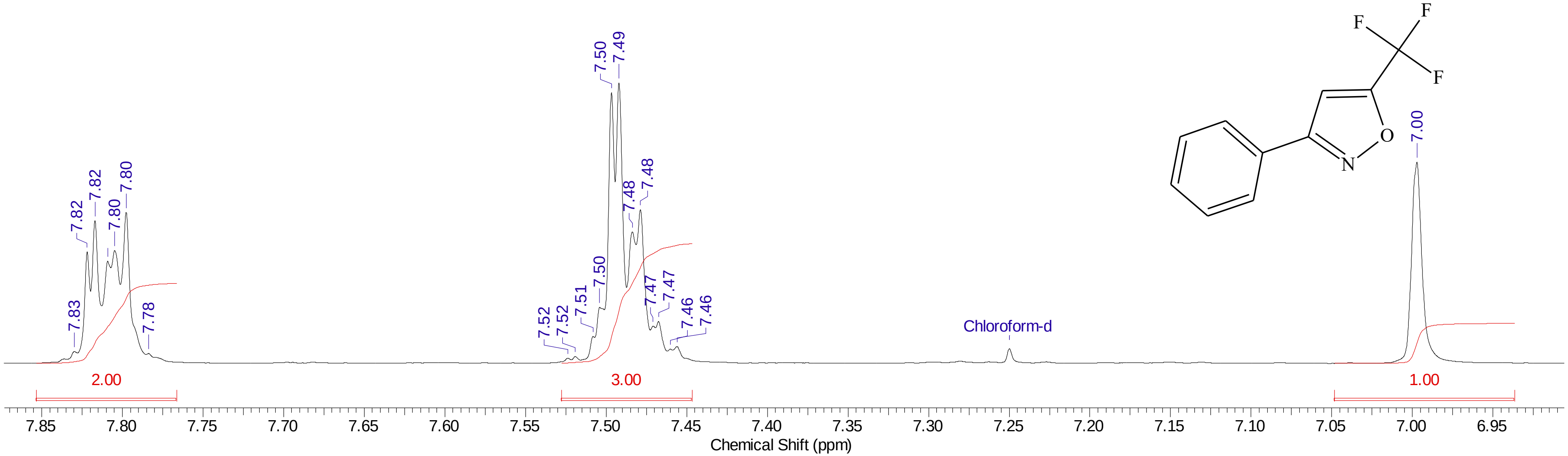


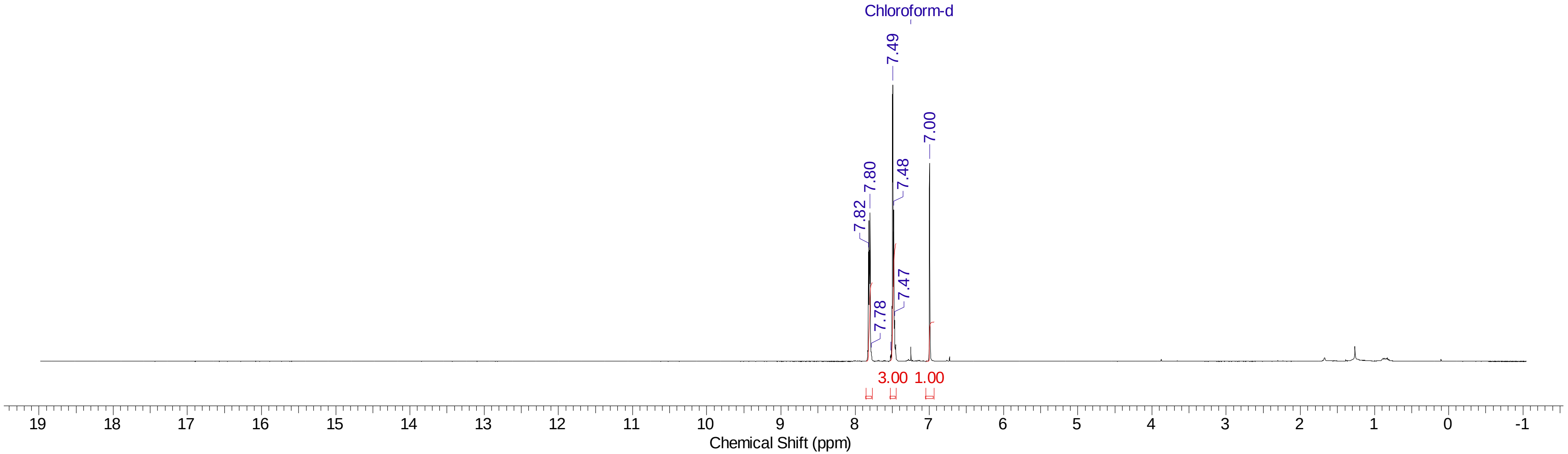

$^{1}$H NMR spectrum of **3a** (400.1 MHz, $CDCl_3$)

3 Aug 2022

| | | | | | |
|---|---|---|---|---|---|
| ***Acquisition Time (sec)*** | 1.7433 | ***Comment*** | Imported from UXNMR. | ***Date*** | 30 Nov 2021 14:57:04 |
| ***File Name*** | C:\DOCS\OUTPUT_301\2021\11.朊 狃黑SZA-BM-2208.F_005001r | | | ***Frequency (MHz)*** | 376.50 |
| ***Nucleus*** | 19F | ***Number of Transients*** | 16 ***Original Points Count*** 131072 | ***Points Count*** | 262144 |
| ***Pulse Sequence*** | zgflqn | ***Solvent*** | CHLOROFORM-D | ***Sweep Width (Hz)*** | 75187.97 |
| ***Temperature (degree C)*** | 27.000 | | | | |

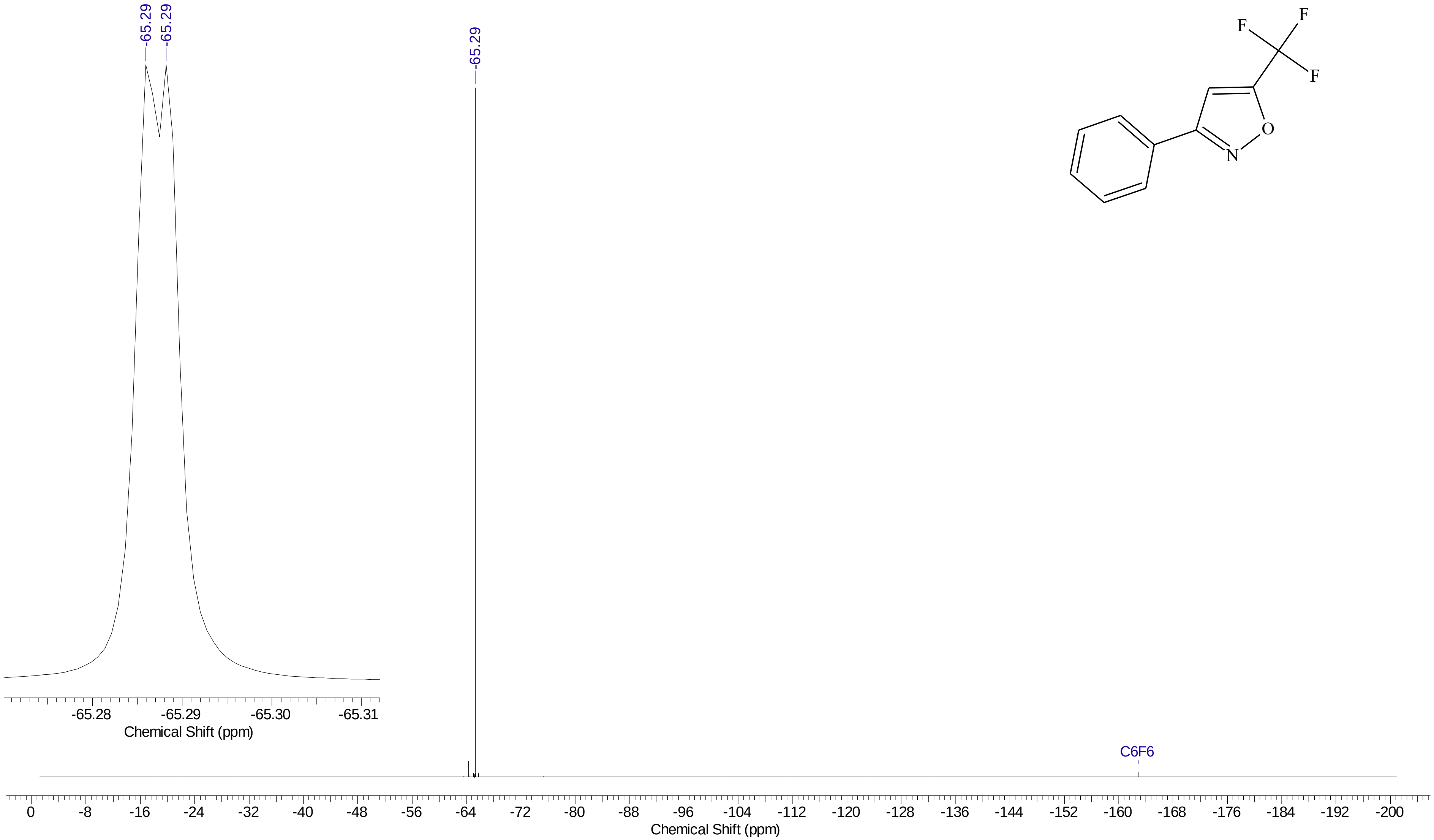

## $^{19}$F NMR spectrum of **3a** (376.5 MHz, $CDCl_3$)

3 Aug 2022

| | | | | | | | |
|---|---|---|---|---|---|---|---|
| ***Acquisition Time (sec)*** | 0.6783 | ***Comment*** | Imported from UXNMR. | | | ***Date*** | 02 Dec 2021 12:00:48 |
| ***File Name*** | C:\DOCS\OUTPUT_301\2021\12.溴贬狃黑SZA-BM-2208.C.C_002001r | | | | | ***Frequency (MHz)*** | 100.61 |
| ***Nucleus*** | 13C | ***Number of Transients*** | 116 | ***Original Points Count*** | 16384 | ***Points Count*** | 131072 |
| ***Pulse Sequence*** | zgpg30 | ***Solvent*** | CHLOROFORM-D | | | ***Sweep Width (Hz)*** | 24154.59 |
| ***Temperature (degree C)*** | 27.000 | | | | | | |

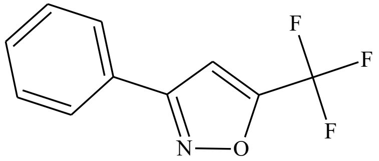


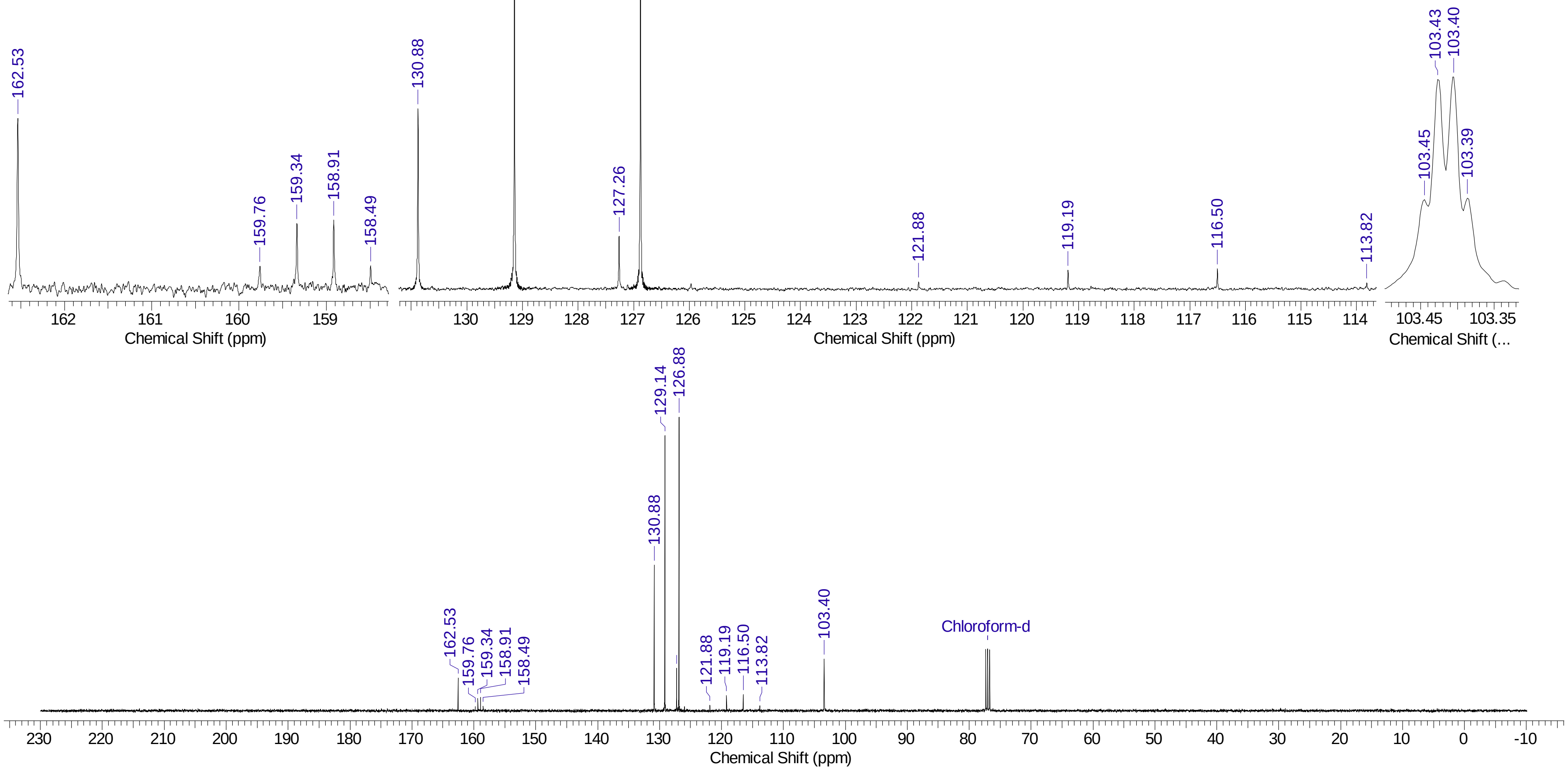

## $^{13}$C NMR spectrum of **3a** (100.6 MHz, $CDCl_3$)

3 Aug 2022

| | | | | | | | |
|---|---|---|---|---|---|---|---|
| ***Acquisition Time (sec)*** | 4.0894 | ***Comment*** | Imported from UXNMR. | | | ***Date*** | 17 Jan 2022 15:09:44 |
| ***File Name*** | C:\DOCS\OUTPUT_301\2022\01. 磤図黒BM-2399.H_001001r | | | | | ***Frequency (MHz)*** | 400.13 |
| ***Nucleus*** | 1H | ***Number of Transients*** | 4 | ***Original Points Count*** | 32768 | ***Points Count*** | 131072 |
| ***Pulse Sequence*** | zg30 | ***Solvent*** | BENZENE-D6 | ***Sweep Width (Hz)*** | 8012.82 | ***Temperature (degree C)*** | 27.000 |

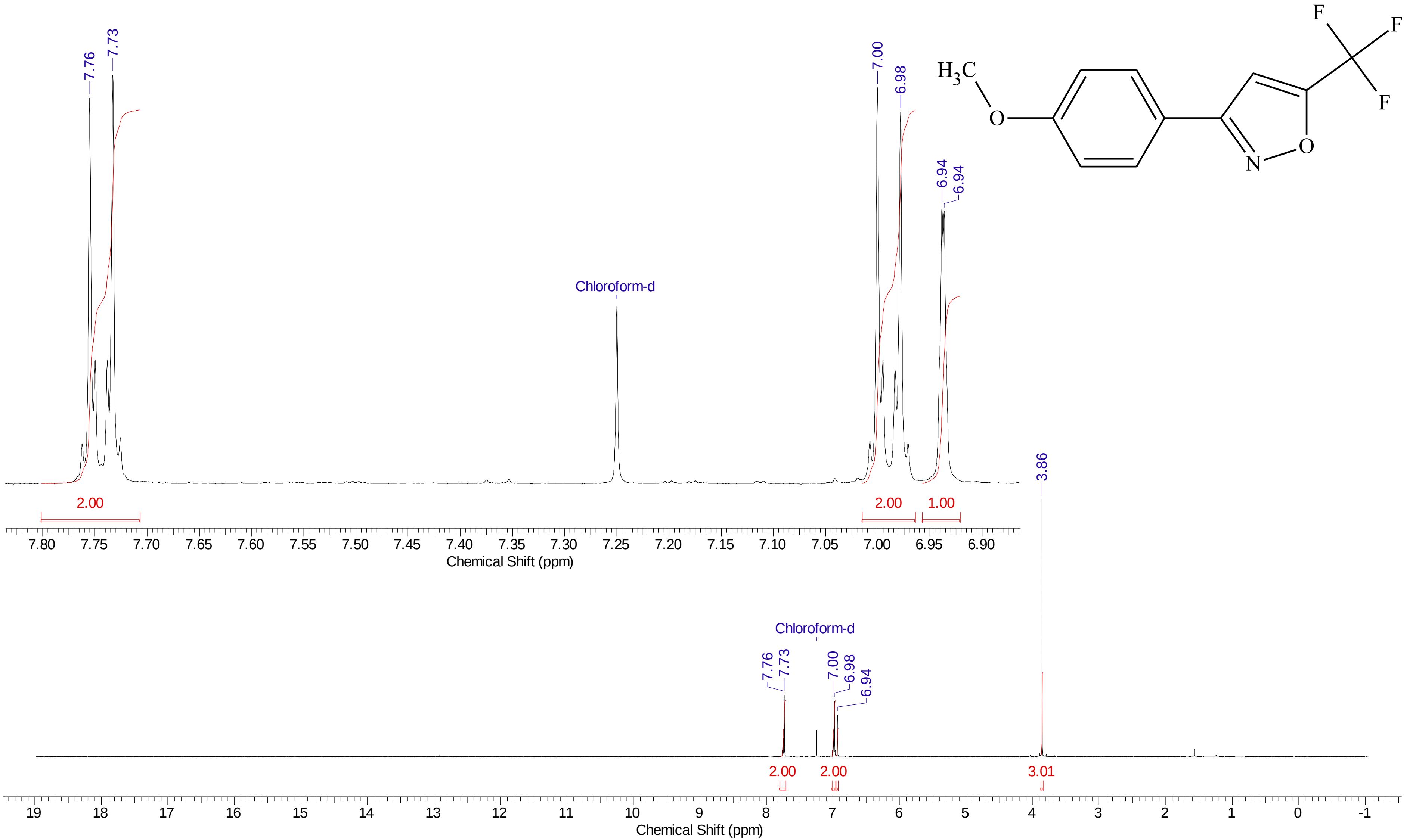

$^{1}H$ NMR spectrum of **3b** (400.1 MHz, $CDCl_3$)

3 Aug 2022

| | | | | | | | |
|---|---|---|---|---|---|---|---|
| ***Acquisition Time (sec)*** | 1.7433 | ***Comment*** | Imported from UXNMR. | | | ***Date*** | 17 Jan 2022 15:26:40 |
| ***File Name*** | C:\DOCS\OUTPUT_301\2022\01. 礅囹黒BM-2399.F_005001r | | | ***Frequency (MHz)*** | 376.50 | ***Nucleus*** | 19F |
| ***Number of Transients*** | 15 | ***Original Points Count*** | 131072 | ***Points Count*** | 262144 | ***Pulse Sequence*** | zgflqn |
| ***Solvent*** | CHLOROFORM-D | | | ***Sweep Width (Hz)*** | 75187.97 | ***Temperature (degree C)*** | 27.000 |

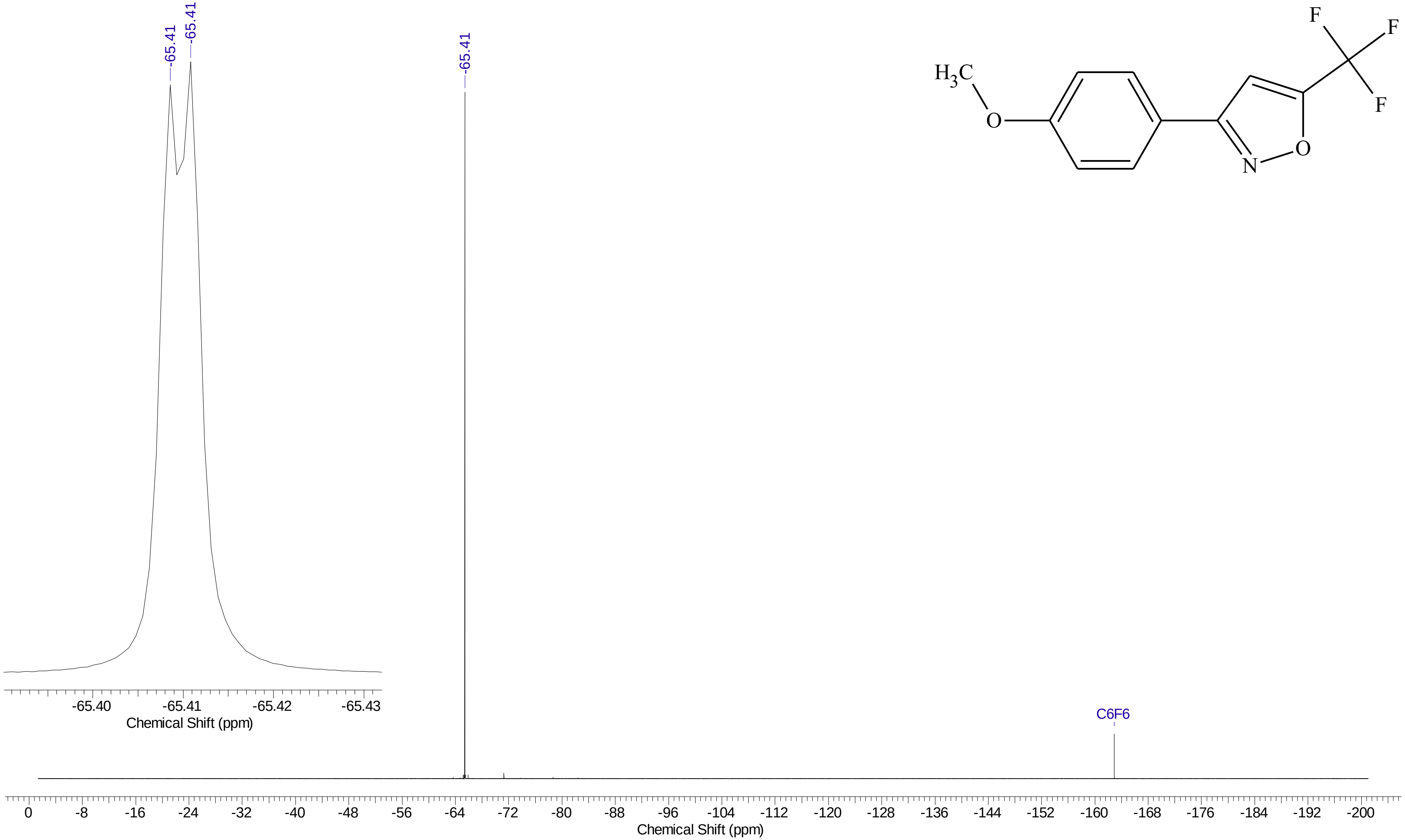

## $^{19}$F NMR spectrum of **3b** (376.5 MHz, $CDCl_3$)

3 Aug 2022

| | | | | | | | |
|---|---|---|---|---|---|---|---|
| ***Acquisition Time (sec)*** | 0.6783 | ***Comment*** | Imported from UXNMR. | | | ***Date*** | 08 Jul 2022 16:02:16 |
| ***File Name*** | C:\DOCS\OUTPUT_301\2022\07.棹 滕\BM-2399-1.C_002001r | | | ***Frequency (MHz)*** | 100.61 | ***Nucleus*** | 13C |
| ***Number of Transients*** | 685 | ***Original Points Count*** | 16384 | ***Points Count*** | 131072 | ***Pulse Sequence*** | zgpg30 |
| ***Solvent*** | CHLOROFORM-D | | | ***Sweep Width (Hz)*** | 24154.59 | ***Temperature (degree C)*** | 27.000 |

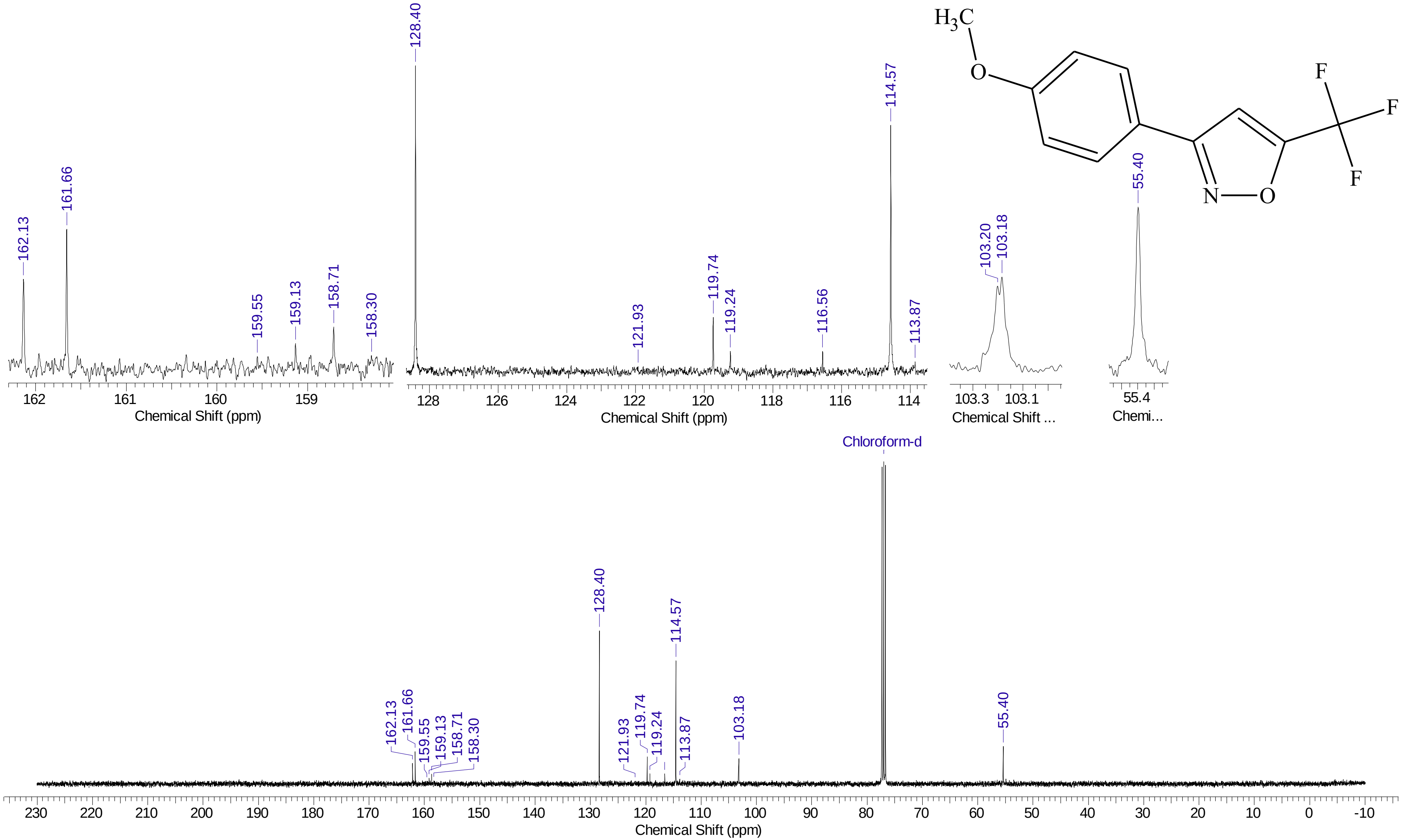

## $^{13}C$ NMR spectrum of **3b** (100.6 MHz, $CDCl_3$)

3 Aug 2022

| | | | | | | | |
|---|---|---|---|---|---|---|---|
| ***Acquisition Time (sec)*** | 4.0894 | ***Comment*** | Imported from UXNMR. | | | ***Date*** | 21 Jan 2022 14:58:44 |
| ***File Name*** | C:\DOCS\OUTPUT_301\2022\01. 磡囹黑BM-2408.H_001001r | | | | | ***Frequency (MHz)*** | 400.13 |
| ***Nucleus*** | 1H | ***Number of Transients*** | 4 | ***Original Points Count*** | 32768 | ***Points Count*** | 131072 |
| ***Pulse Sequence*** | zg30 | ***Solvent*** | CHLOROFORM-D | | | ***Sweep Width (Hz)*** | 8012.82 |
| ***Temperature (degree C)*** | 27.000 | | | | | | |

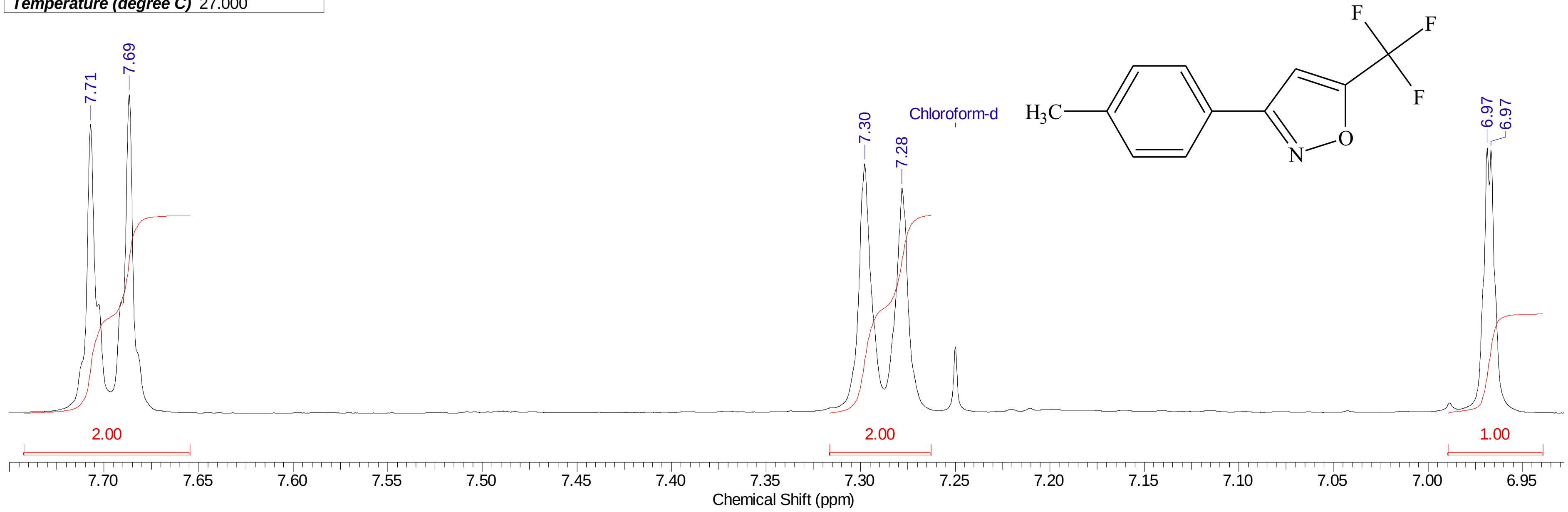


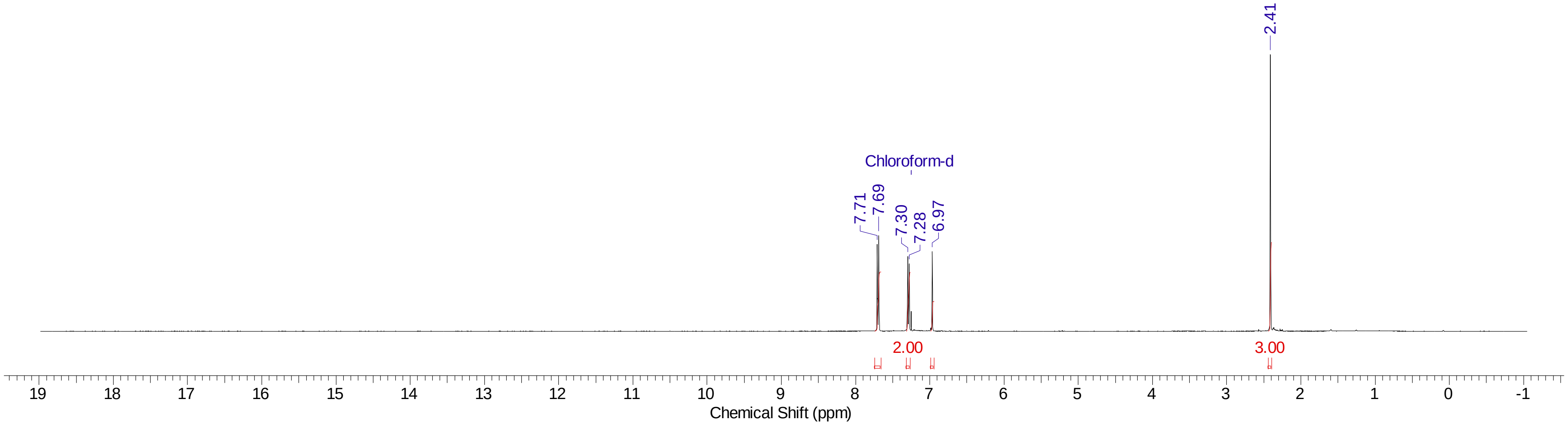

## $^{1}H$ NMR spectrum of **3d** (400.1 MHz, $CDCl_3$)

3 Aug 2022

| | | | | | | | |
|---|---|---|---|---|---|---|---|
| ***Acquisition Time (sec)*** | 1.7433 | ***Comment*** | Imported from UXNMR. | | | ***Date*** | 21 Jan 2022 15:22:12 |
| ***File Name*** | C:\DOCS\OUTPUT_301\2022\01. 磤囹黒BM-2408.F_005001r | | | | | ***Frequency (MHz)*** | 376.50 |
| ***Nucleus*** | 19F | ***Number of Transients*** | 10 | ***Original Points Count*** | 131072 | ***Points Count*** | 262144 |
| ***Pulse Sequence*** | zgflqn | ***Solvent*** | Acetone | ***Sweep Width (Hz)*** | 75187.97 | ***Temperature (degree C)*** | 27.000 |

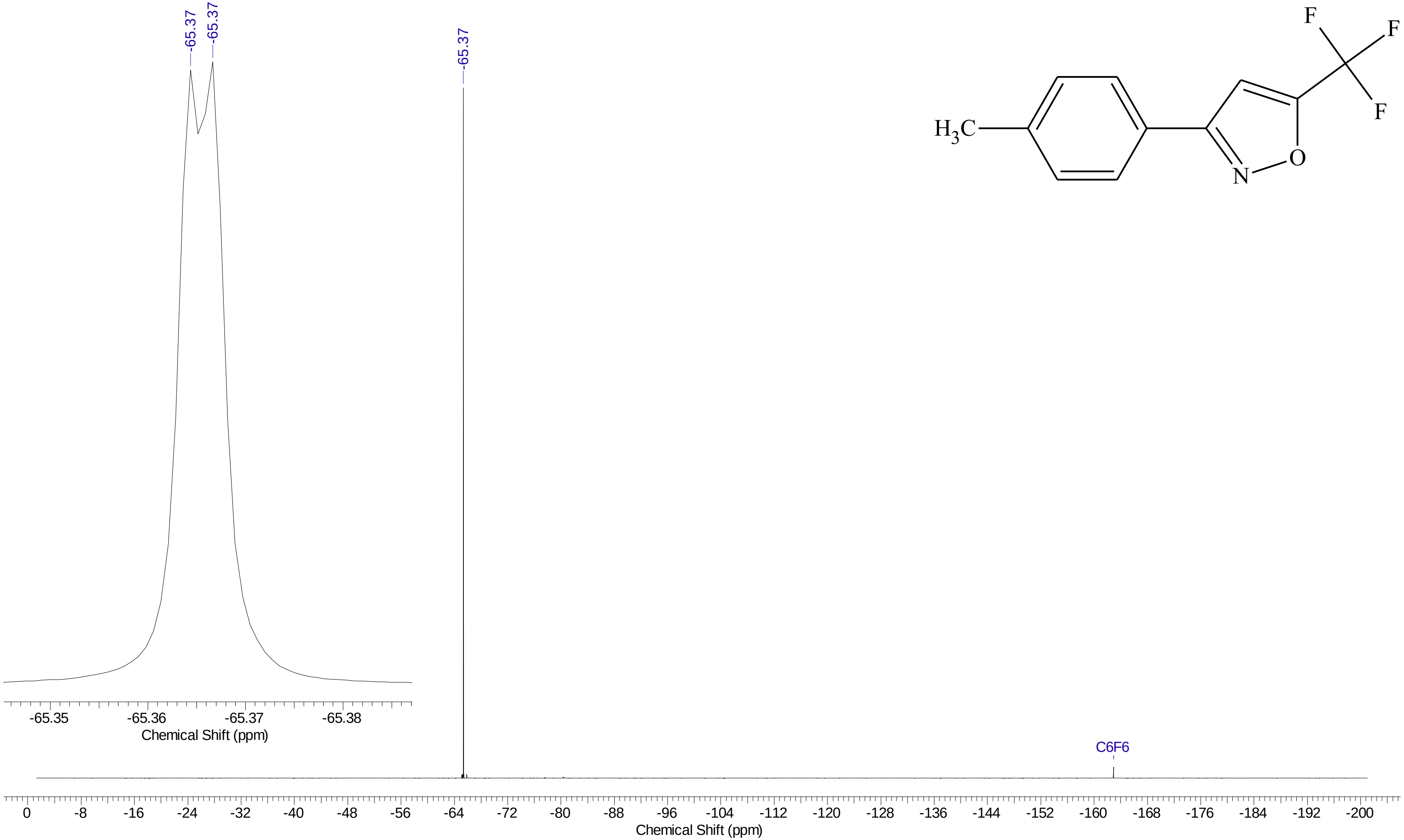

# $^{19}$F NMR spectrum of **3d** (376.5 MHz, $CDCl_3$)

3 Aug 2022

| | | | | | | | |
|---|---|---|---|---|---|---|---|
| ***Acquisition Time (sec)*** | 0.6783 | ***Comment*** | Imported from UXNMR. | | | ***Date*** | 24 Jan 2022 14:37:04 |
| ***File Name*** | C:\DOCS\OUTPUT_301\2022\01. 礅図黒BM-2408.C_002001r | | | ***Frequency (MHz)*** | 100.61 | ***Nucleus*** | 13C |
| ***Number of Transients*** | 201 | ***Original Points Count*** | 16384 | ***Points Count*** | 131072 | ***Pulse Sequence*** | zgpg30 |
| ***Solvent*** | CHLOROFORM-D | | | ***Sweep Width (Hz)*** | 24154.59 | ***Temperature (degree C)*** | 27.000 |

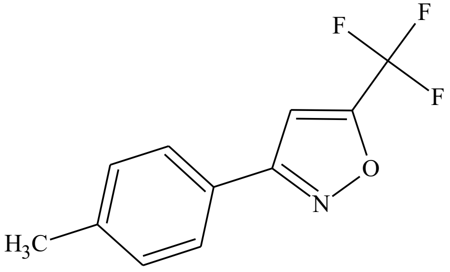


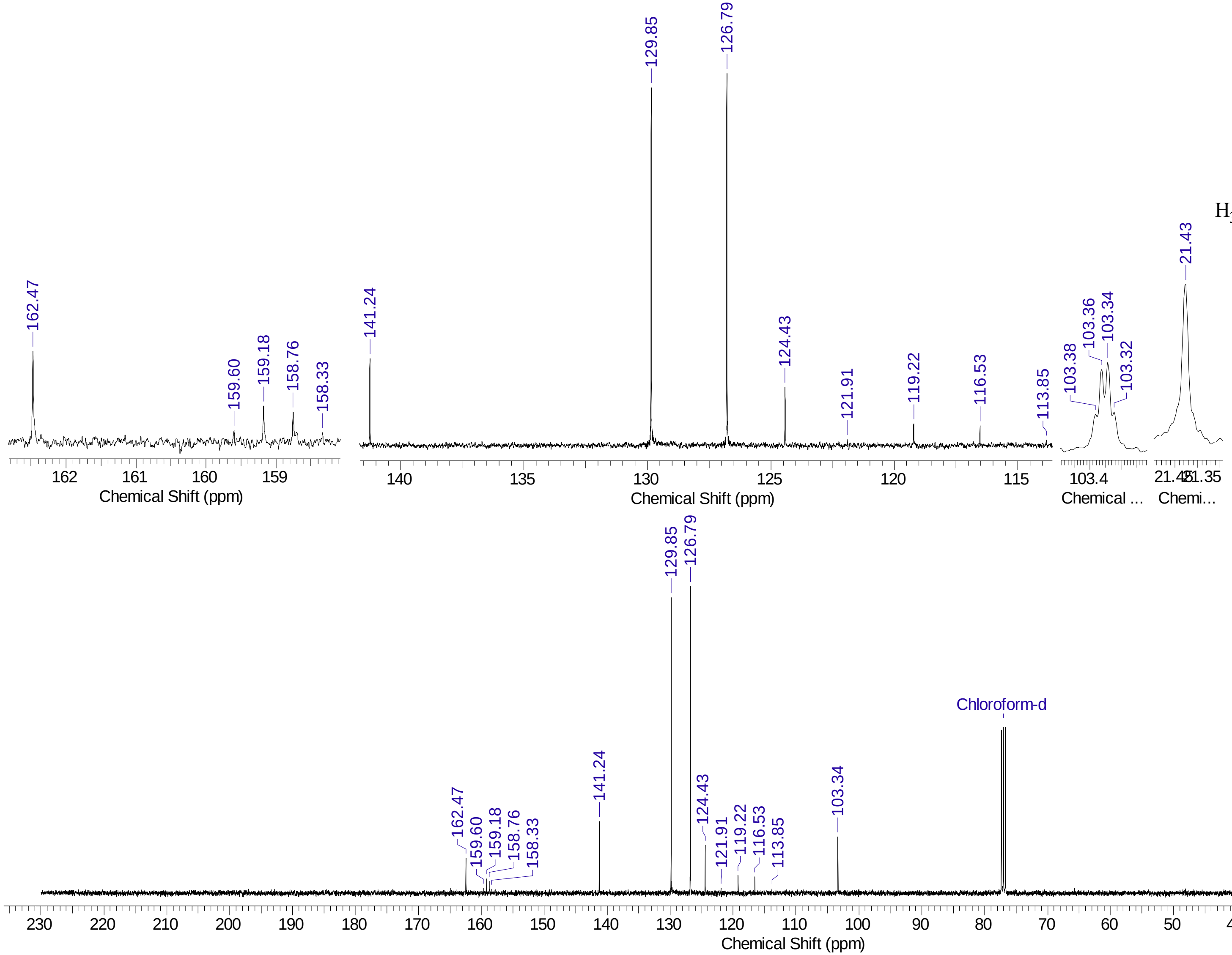

## $^{13}$C NMR spectrum of **3d** (100.6 MHz, $CDCl_3$)

3 Aug 2022

| | | | | | | | |
|---|---|---|---|---|---|---|---|
| ***Acquisition Time (sec)*** | 4.0894 | ***Comment*** | Imported from UXNMR. | | | ***Date*** | 24 Jan 2022 15:19:32 |
| ***File Name*** | C:\DOCS\OUTPUT_301\2022\01. 磤図黒BM-2215.H_001001r | | | | | ***Frequency (MHz)*** | 400.13 |
| ***Nucleus*** | 1H | ***Number of Transients*** | 4 | ***Original Points Count*** | 32768 | ***Points Count*** | 131072 |
| ***Pulse Sequence*** | zg30 | ***Solvent*** | CHLOROFORM-D | | | ***Sweep Width (Hz)*** | 8012.82 |
| ***Temperature (degree C)*** | 27.000 | | | | | | |

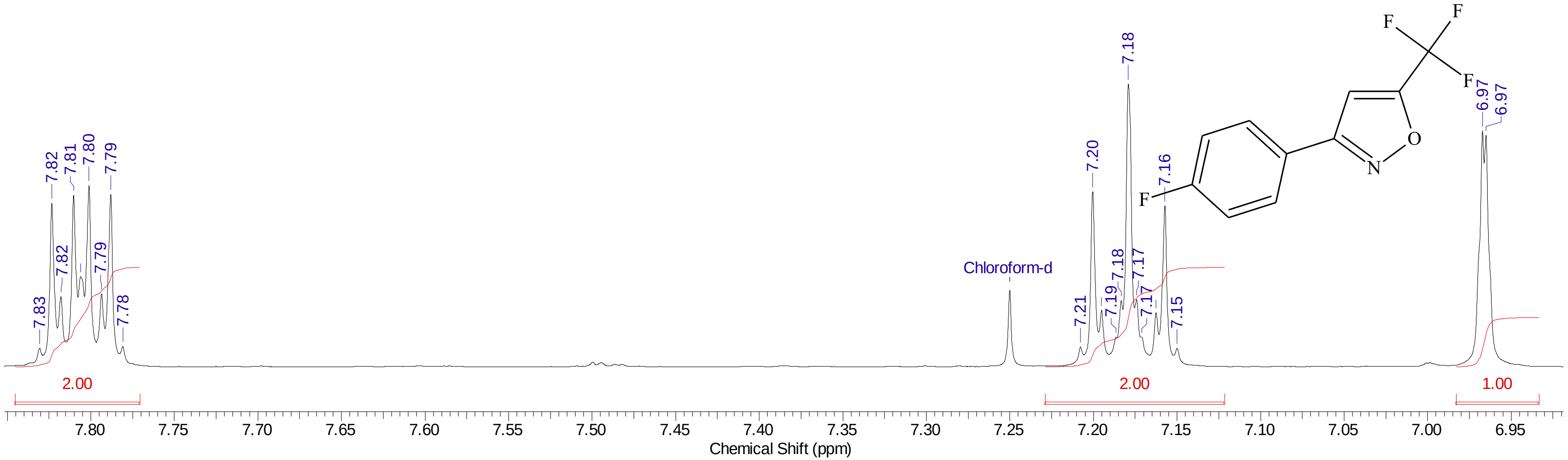


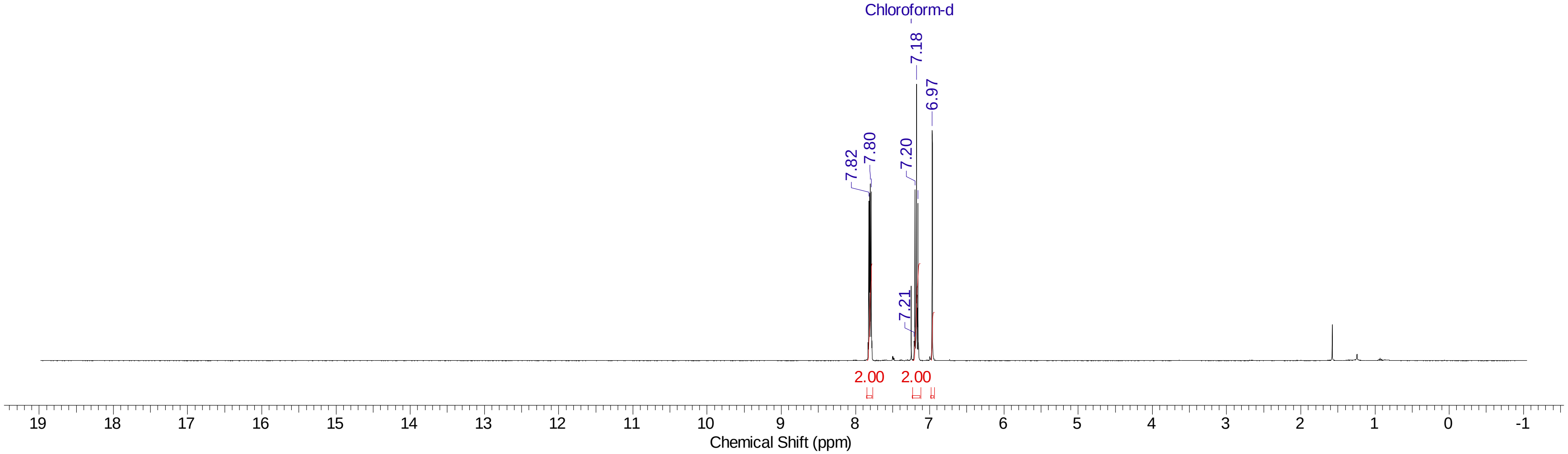

## $^{1}$H NMR spectrum of **3e** (400.1 MHz, $CDCl_3$)

3 Aug 2022

| | | | | | | | |
|---|---|---|---|---|---|---|---|
| ***Acquisition Time (sec)*** | 1.7433 | ***Comment*** | Imported from UXNMR. | | | ***Date*** | 24 Jan 2022 15:27:48 |
| ***File Name*** | C:\DOCS\OUTPUT_301\2022\01. 磡図黒BM-2215.F_005001r | | | | | ***Frequency (MHz)*** | 376.50 |
| ***Nucleus*** | 19F | ***Number of Transients*** | 16 | ***Original Points Count*** | 131072 | ***Points Count*** | 262144 |
| ***Pulse Sequence*** | zgflqn | ***Solvent*** | Acetone | ***Sweep Width (Hz)*** | 75187.97 | ***Temperature (degree C)*** | 27.000 |

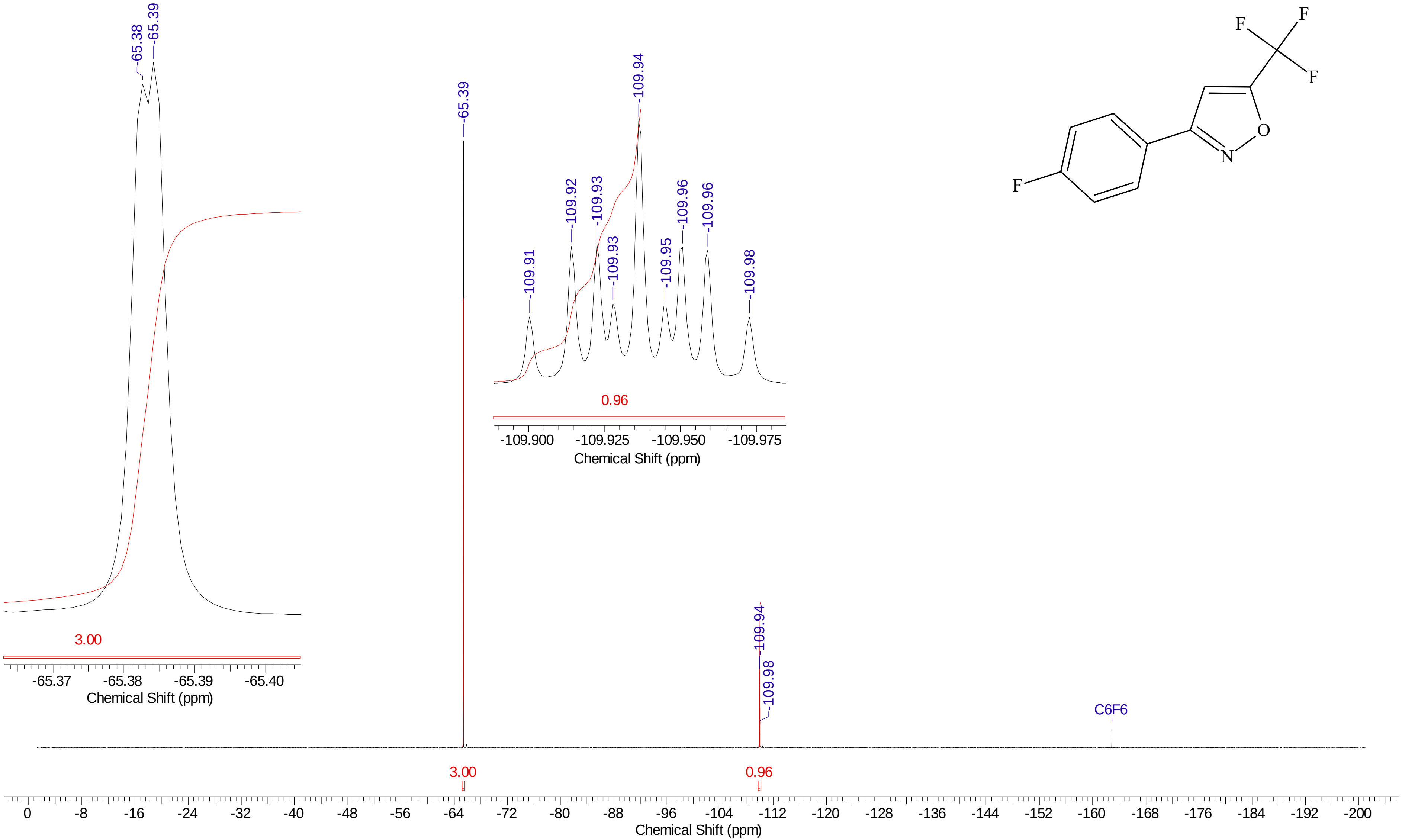

## $^{19}$F NMR spectrum of **3e** (376.5 MHz, $CDCl_3$)

3 Aug 2022

| | | | | | | | |
|---|---|---|---|---|---|---|---|
| ***Acquisition Time (sec)*** | 0.6783 | ***Comment*** | Imported from UXNMR. | | | ***Date*** | 25 Jan 2022 16:04:58 |
| ***File Name*** | C:\DOCS\OUTPUT_301\2022\01. 磝囹黒BM-2215.C_002001r | | | ***Frequency (MHz)*** | 100.61 | ***Nucleus*** | 13C |
| ***Number of Transients*** | 273 | ***Original Points Count*** | 16384 | ***Points Count*** | 131072 | ***Pulse Sequence*** | zgpg30 |
| ***Solvent*** | CHLOROFORM-D | | | ***Sweep Width (Hz)*** | 24154.59 | ***Temperature (degree C)*** | 27.000 |

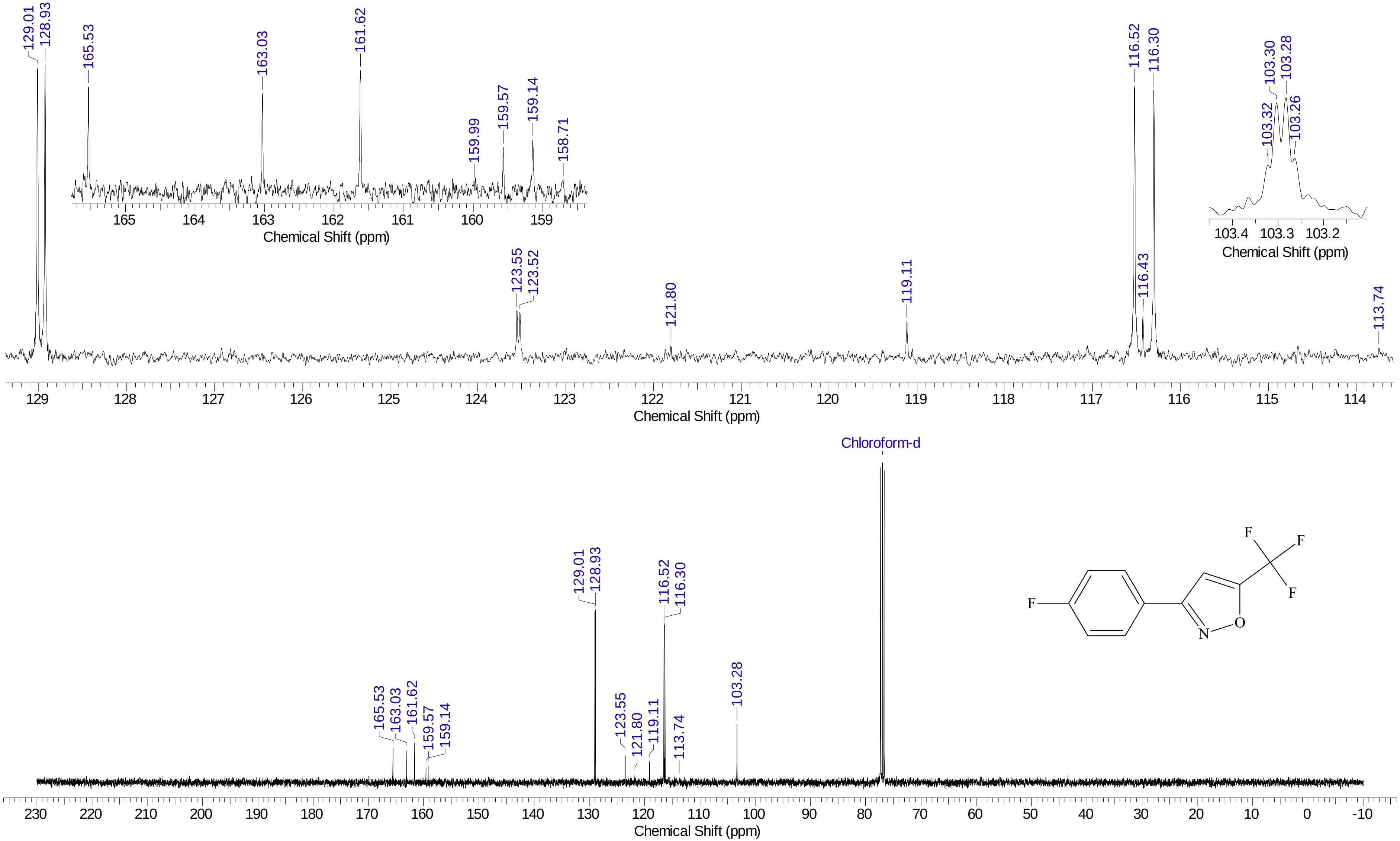

$^{13}C$ NMR spectrum of **3e** (100.6 MHz, $CDCl_3$)

3 Aug 2022

| | | | | | | | |
|---|---|---|---|---|---|---|---|
| ***Acquisition Time (sec)*** | 4.0894 | ***Comment*** | Imported from UXNMR. | | | ***Date*** | 17 Jan 2022 15:14:56 |
| ***File Name*** | C:\DOCS\OUTPUT_301\2022\01. 礅図黒BM-2402.H_001001r | | | | | ***Frequency (MHz)*** | 400.13 |
| ***Nucleus*** | 1H | ***Number of Transients*** | 4 | ***Original Points Count*** | 32768 | ***Points Count*** | 131072 |
| ***Pulse Sequence*** | zg30 | ***Solvent*** | BENZENE-D6 | ***Sweep Width (Hz)*** | 8012.82 | ***Temperature (degree C)*** | 27.000 |

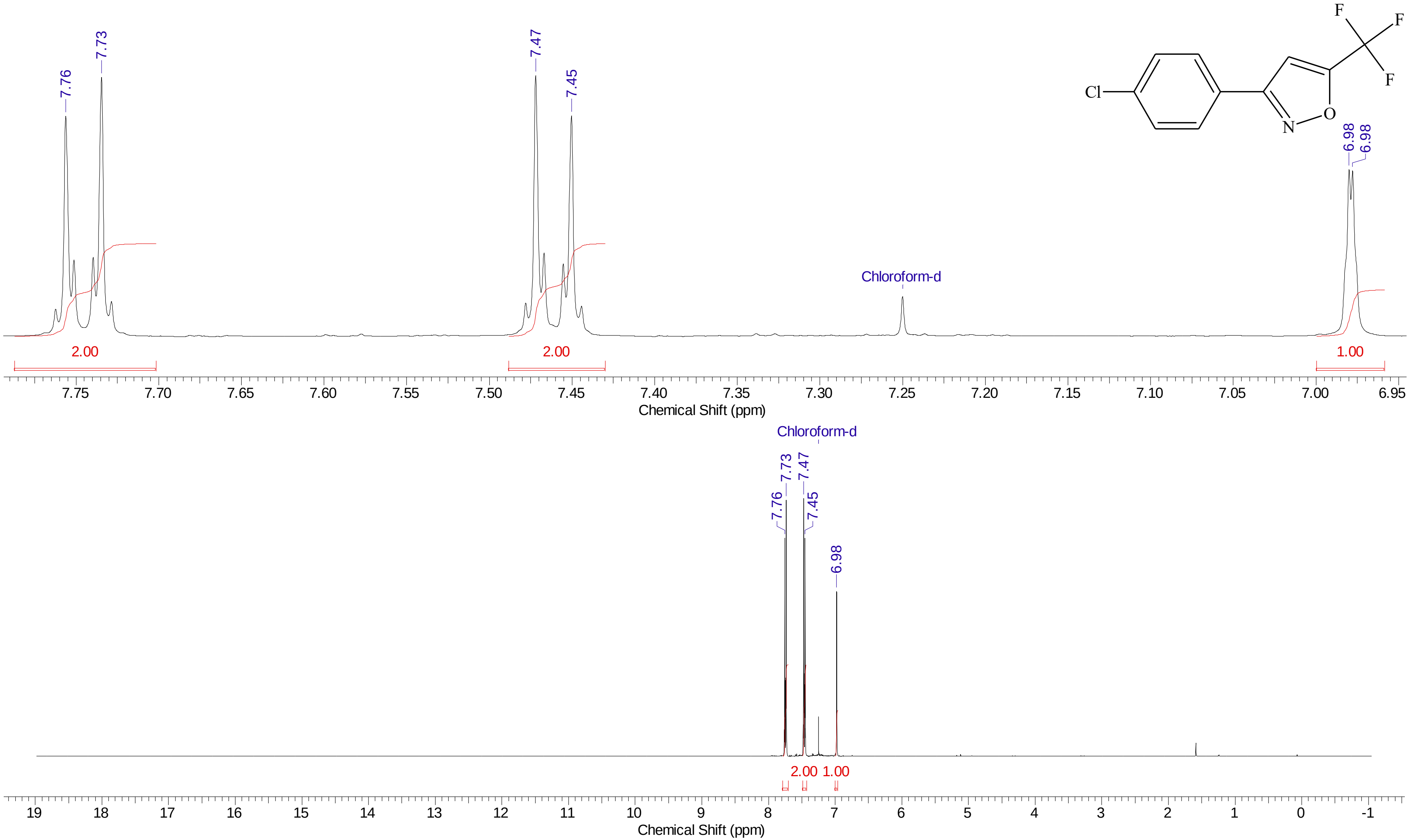

## $^{1}$H NMR spectrum of **3f** (400.1 MHz, $CDCl_3$)

3 Aug 2022

| | | | | | |
|---|---|---|---|---|---|
| ***Acquisition Time (sec)*** | 1.7433 | ***Comment*** | Imported from UXNMR. | ***Date*** | 17 Jan 2022 15:30:42 |
| ***File Name*** | C:\DOCS\OUTPUT_301\2022\01. 礅囹黑BM-2402.F_005001r | ***Frequency (MHz)*** | 376.50 | ***Nucleus*** | 19F |
| ***Number of Transients*** | 9 | ***Original Points Count*** 131072 | ***Points Count*** 262144 | ***Pulse Sequence*** | zgflqn |
| ***Solvent*** | CHLOROFORM-D | ***Sweep Width (Hz)*** | 75187.97 | ***Temperature (degree C)*** | 27.000 |

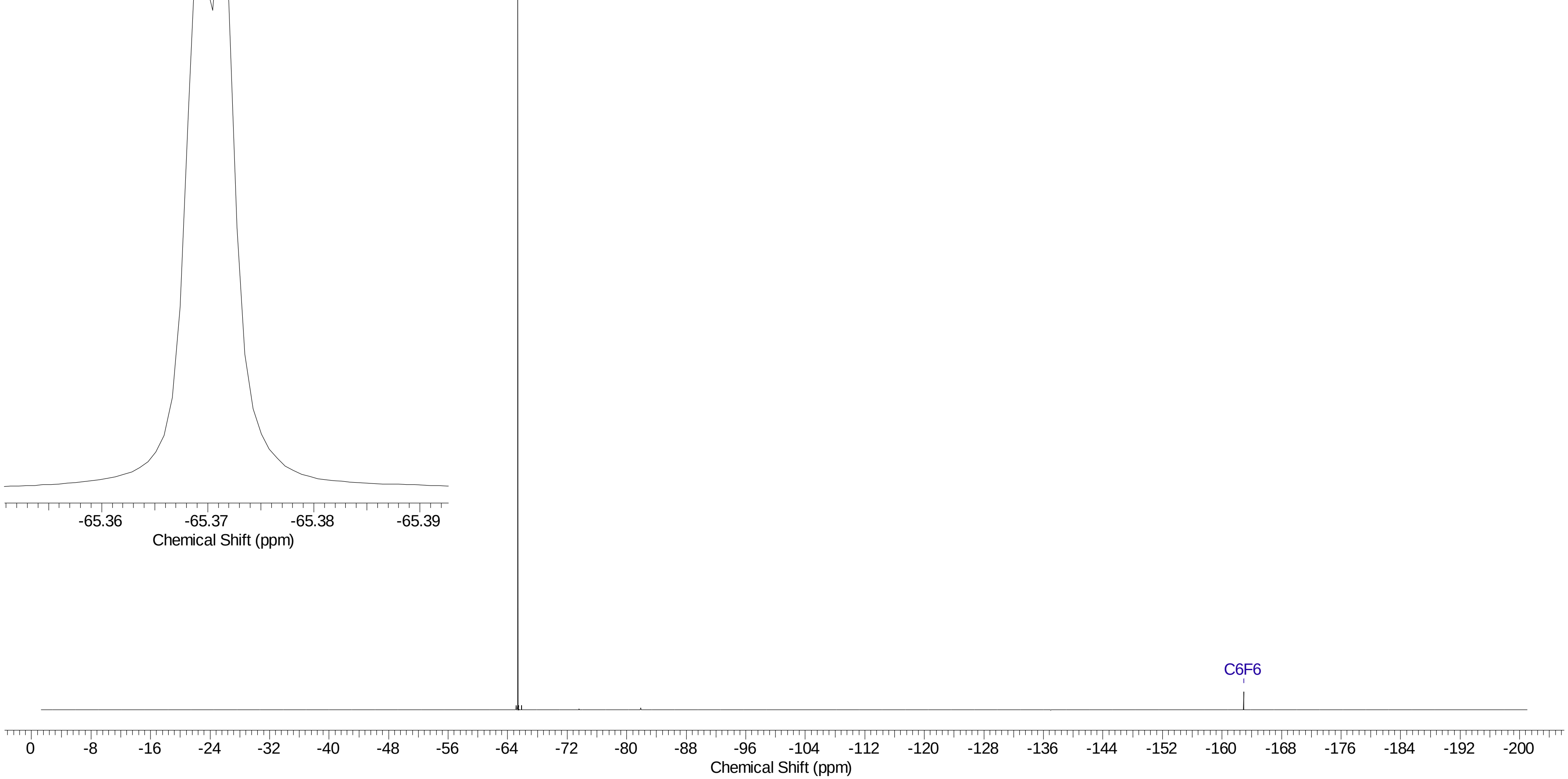


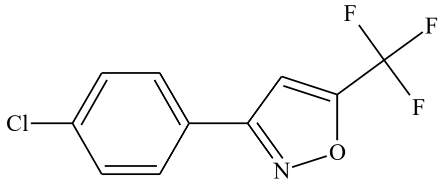

$^{19}$F NMR spectrum of **3f** (376.5 MHz, $CDCl_3$)

3 Aug 2022

| | | | | | | | |
|---|---|---|---|---|---|---|---|
| ***Acquisition Time (sec)*** | 0.6783 | ***Comment*** | Imported from UXNMR. | | | ***Date*** | 18 Jan 2022 15:06:44 |
| ***File Name*** | C:\DOCS\OUTPUT_301\2022\01. 磡図黒BM-2402.C_002001r | | | ***Frequency (MHz)*** | 100.61 | ***Nucleus*** | 13C |
| ***Number of Transients*** | 121 | ***Original Points Count*** | 16384 | ***Points Count*** | 131072 | ***Pulse Sequence*** | zgpg30 |
| ***Solvent*** | CHLOROFORM-D | | | ***Sweep Width (Hz)*** | 24154.59 | ***Temperature (degree C)*** | 27.000 |

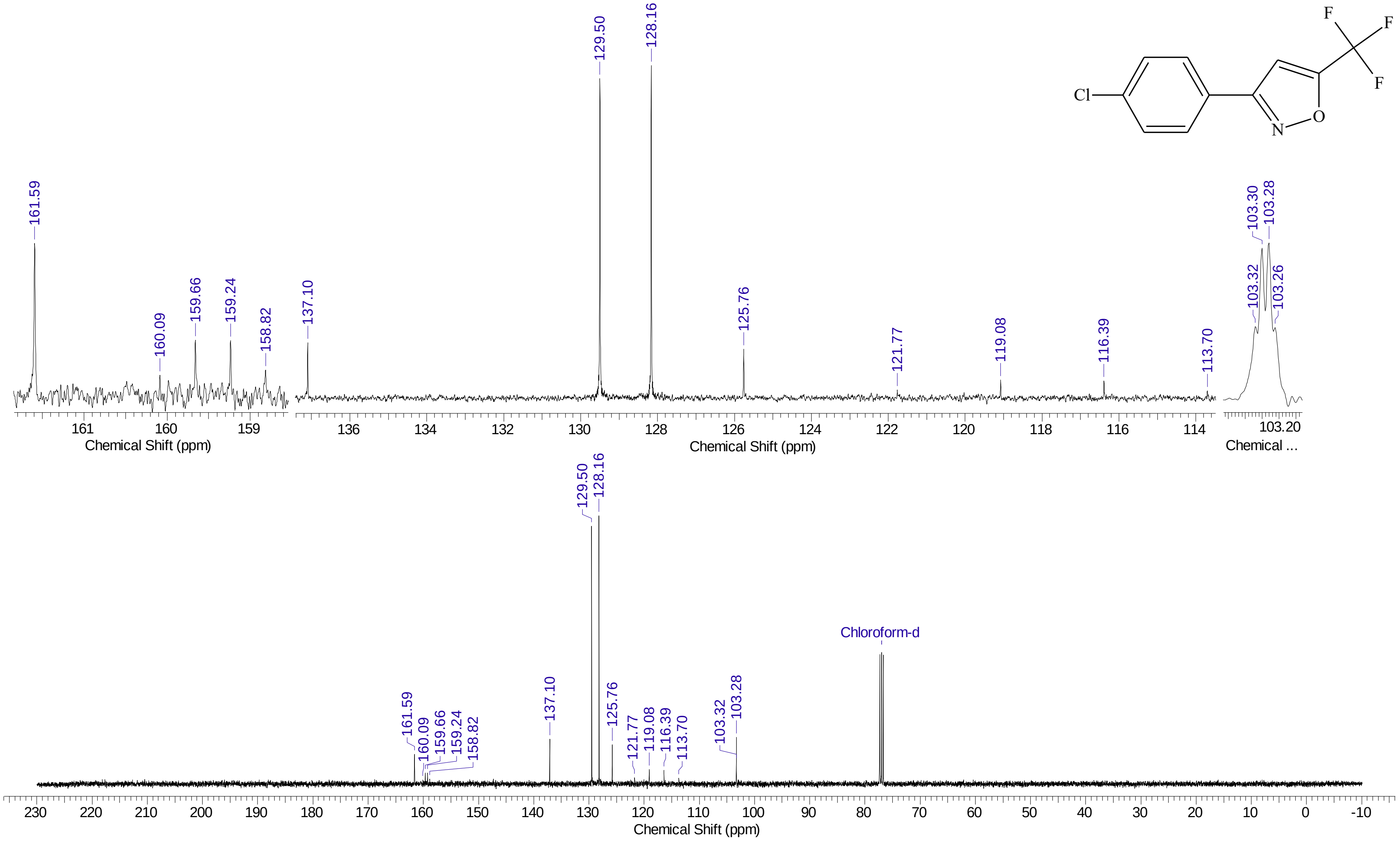

^13C NMR spectrum of **3f** (100.6 MHz, $CDCl_3$)

3 Aug 2022

| | | | | | | | |
|---|---|---|---|---|---|---|---|
| ***Acquisition Time (sec)*** | 4.0894 | ***Comment*** | Imported from UXNMR. | | | ***Date*** | 17 Jan 2022 15:18:40 |
| ***File Name*** | C:\DOCS\OUTPUT_301\2022\01. 礅図黒BM-2405.H_001001r | | | | | ***Frequency (MHz)*** | 400.13 |
| ***Nucleus*** | 1H | ***Number of Transients*** | 4 | ***Original Points Count*** | 32768 | ***Points Count*** | 131072 |
| ***Pulse Sequence*** | zg30 | ***Solvent*** | BENZENE-D6 | ***Sweep Width (Hz)*** | 8012.82 | ***Temperature (degree C)*** | 27.000 |

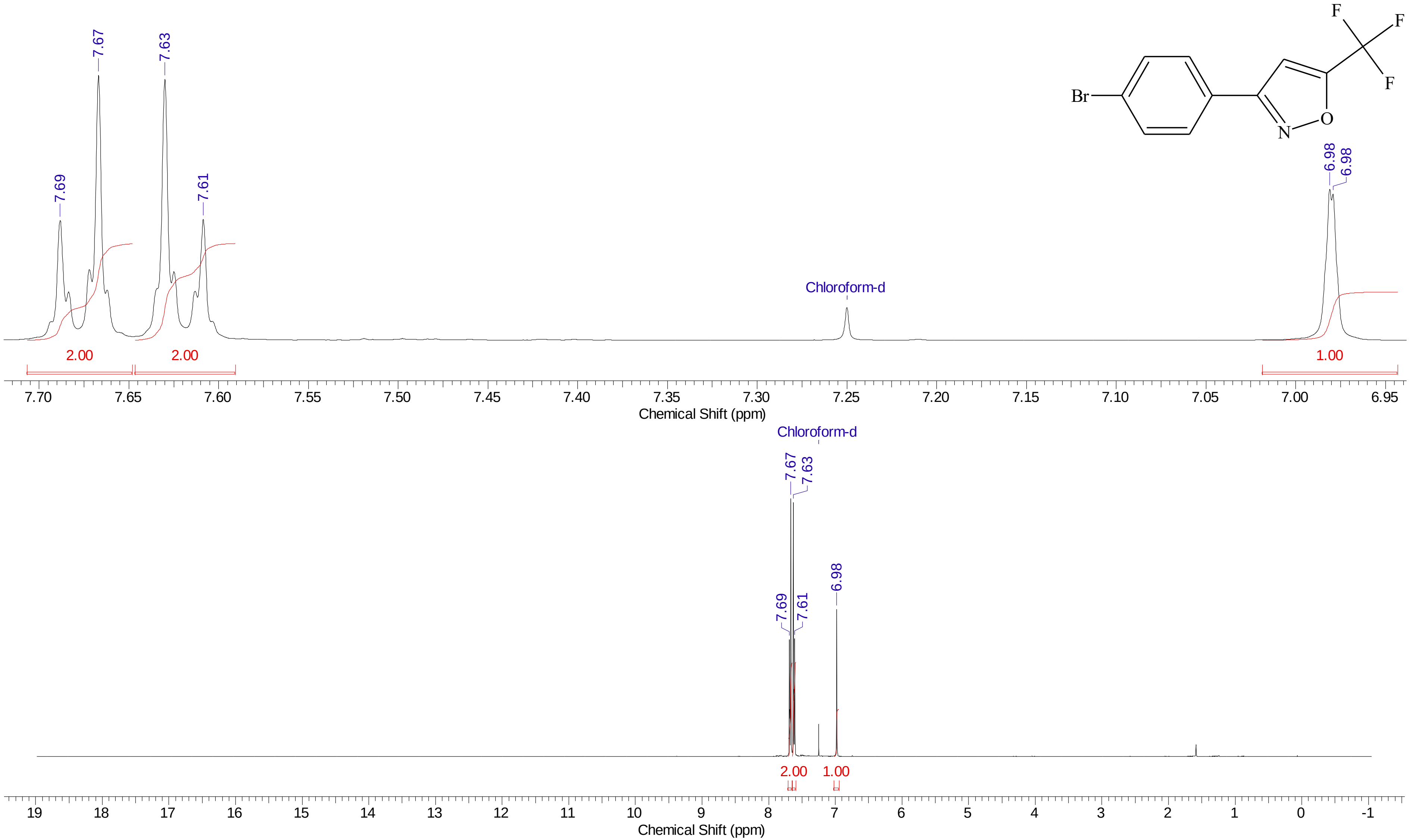

$^{1}$H NMR spectrum of **3g** (400.1 MHz, $CDCl_3$)

3 Aug 2022

| | | | | | | | |
|---|---|---|---|---|---|---|---|
| ***Acquisition Time (sec)*** | 1.7433 | ***Comment*** | Imported from UXNMR. | | | ***Date*** | 17 Jan 2022 15:34:48 |
| ***File Name*** | C:\DOCS\OUTPUT_301\2022\01. 礅図黒BM-2405.F_005001r | | | ***Frequency (MHz)*** | 376.50 | ***Nucleus*** | 19F |
| ***Number of Transients*** | 11 | ***Original Points Count*** | 131072 | ***Points Count*** | 262144 | ***Pulse Sequence*** | zgflqn |
| ***Solvent*** | CHLOROFORM-D | | | ***Sweep Width (Hz)*** | 75187.97 | ***Temperature (degree C)*** | 27.000 |

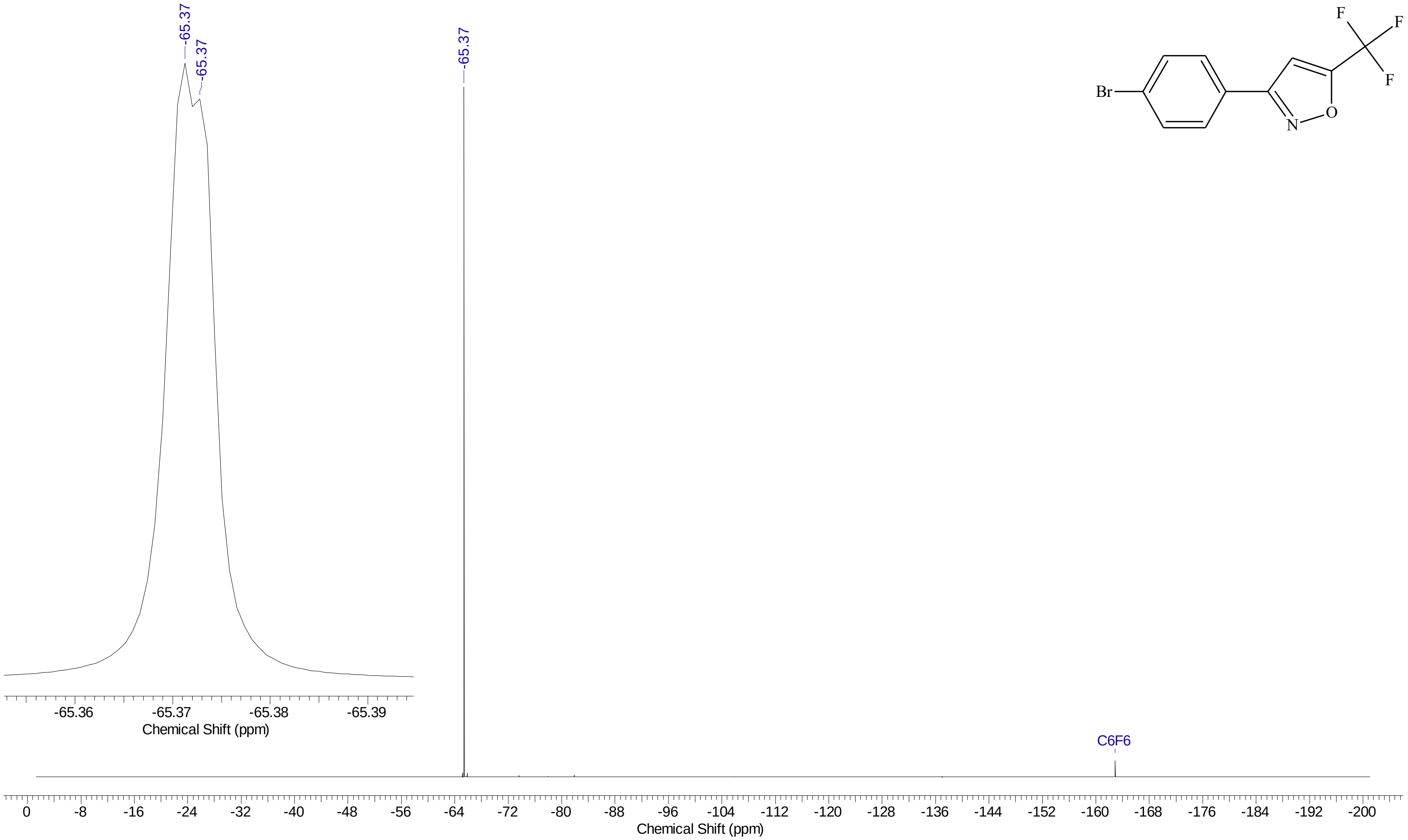

## $^{19}$F NMR spectrum of **3g** (376.5 MHz, $CDCl_3$)

3 Aug 2022

| | | | | | | | |
|---|---|---|---|---|---|---|---|
| ***Acquisition Time (sec)*** | 0.6783 | ***Comment*** | Imported from UXNMR. | | | ***Date*** | 18 Jan 2022 15:36:44 |
| ***File Name*** | C:\DOCS\OUTPUT_301\2022\01. 礅囨黒BM-2405.C_002001r | | | ***Frequency (MHz)*** | 100.61 | ***Nucleus*** | 13C |
| ***Number of Transients*** | 177 | ***Original Points Count*** | 16384 | ***Points Count*** | 131072 | ***Pulse Sequence*** | zgpg30 |
| ***Solvent*** | CHLOROFORM-D | | | ***Sweep Width (Hz)*** | 24154.59 | ***Temperature (degree C)*** | 27.000 |

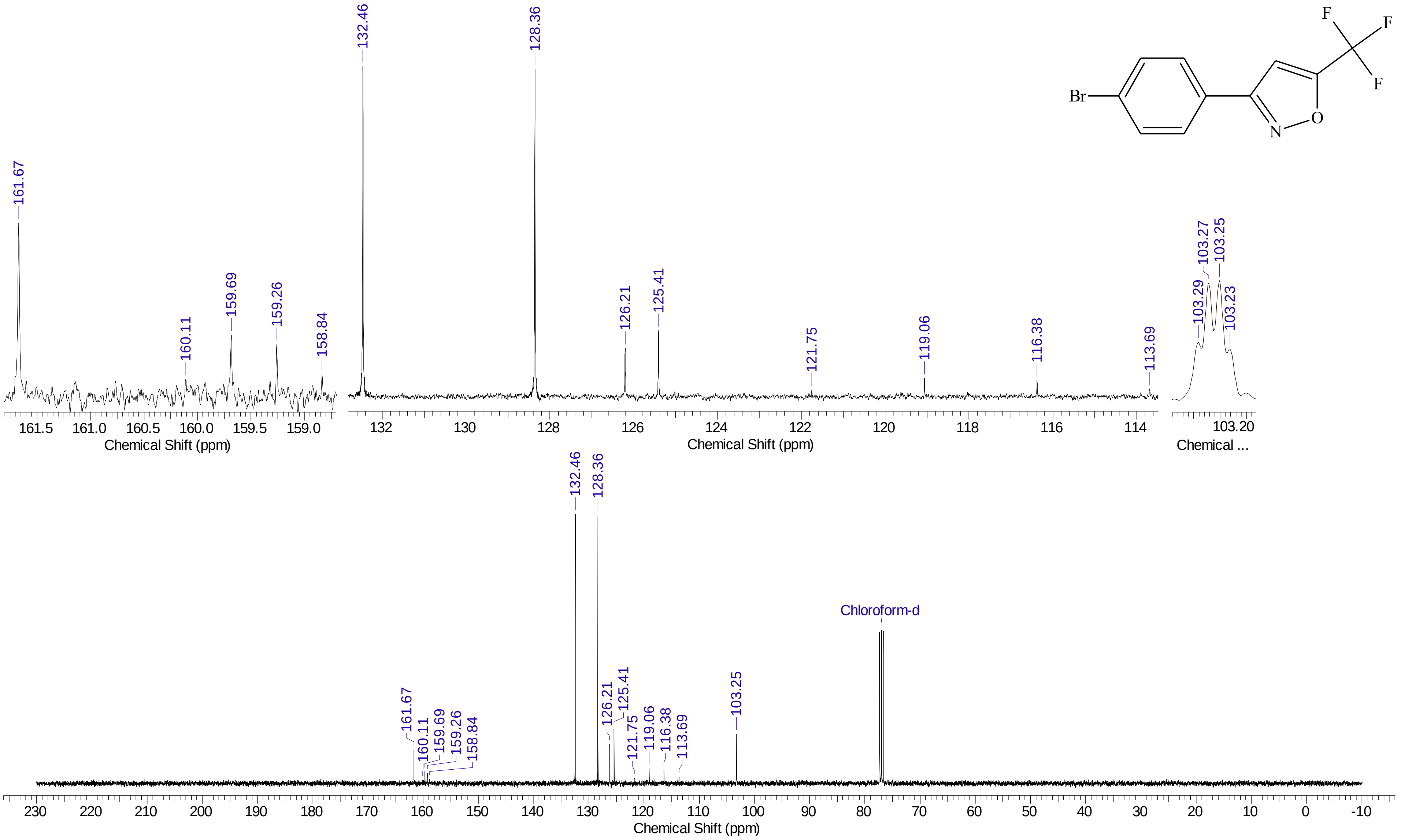

$^{13}$C NMR spectrum of **3g** (100.6 MHz, $CDCl_3$)

3 Aug 2022

| | | | | | | | |
|---|---|---|---|---|---|---|---|
| ***Acquisition Time (sec)*** | 4.0894 | ***Comment*** | Imported from UXNMR. | | | ***Date*** | 18 Jan 2022 15:00:28 |
| ***File Name*** | C:\DOCS\OUTPUT_301\2022\01. 礅図黒BM-2404-3.H_001001r | | | | | ***Frequency (MHz)*** | 400.13 |
| ***Nucleus*** | 1H | ***Number of Transients*** | 4 | ***Original Points Count*** | 32768 | ***Points Count*** | 131072 |
| ***Pulse Sequence*** | zg30 | ***Solvent*** | BENZENE-D6 | ***Sweep Width (Hz)*** | 8012.82 | ***Temperature (degree C)*** | 27.000 |

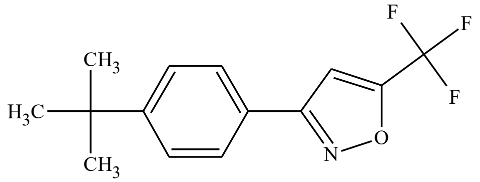


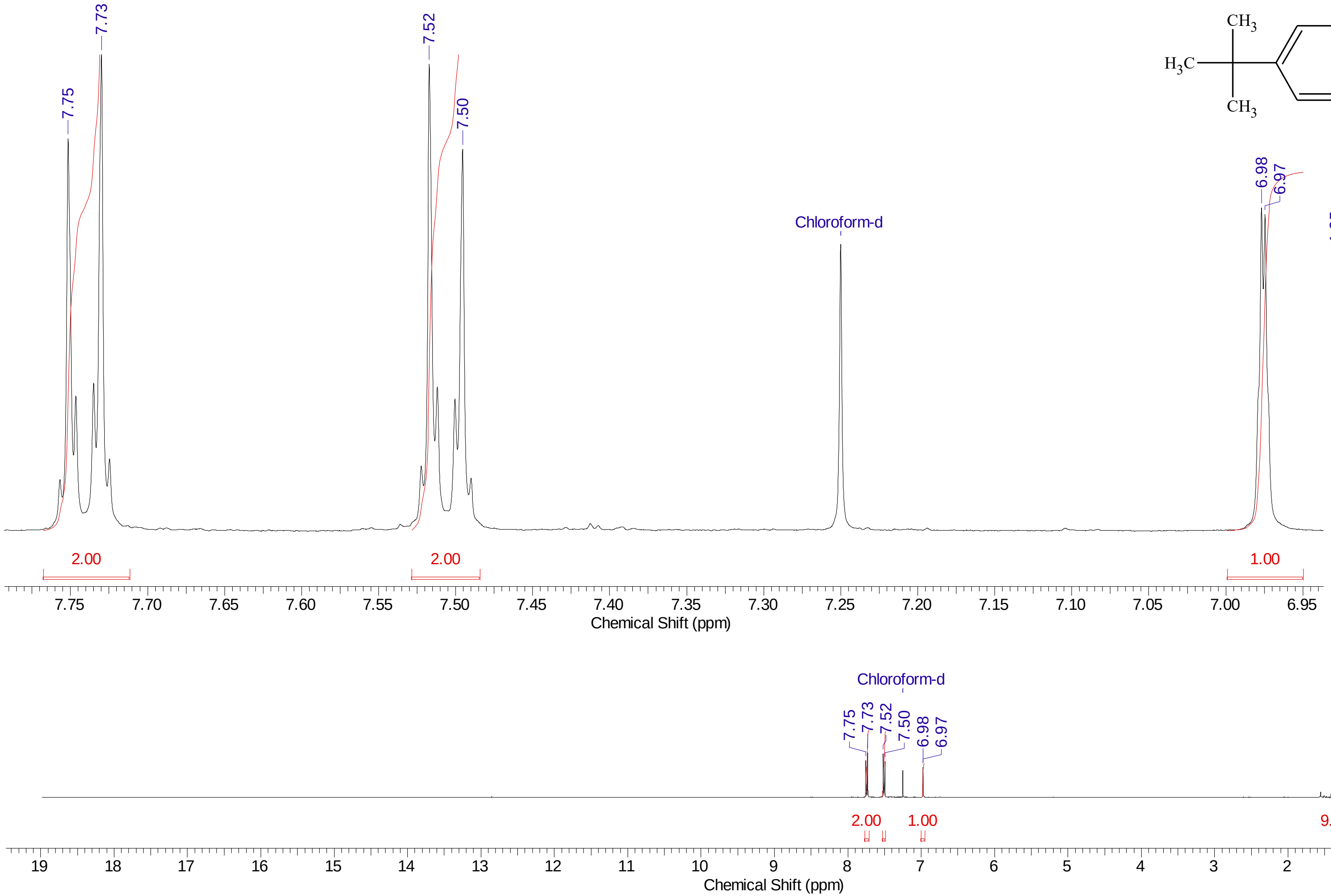

# $^{1}$H NMR spectrum of **3h** (400.1 MHz, $CDCl_3$)

3 Aug 2022

| | | | | | | | |
|---|---|---|---|---|---|---|---|
| ***Acquisition Time (sec)*** | 1.7433 | ***Comment*** | Imported from UXNMR. | | | ***Date*** | 18 Jan 2022 15:10:48 |
| ***File Name*** | C:\DOCS\OUTPUT_301\2022\01. 磡囹黑BM-2404-4.F_005001r | | | | | ***Frequency (MHz)*** | 376.50 |
| ***Nucleus*** | 19F | ***Number of Transients*** | 16 | ***Original Points Count*** | 131072 | ***Points Count*** | 262144 |
| ***Pulse Sequence*** | zgflqn | ***Solvent*** | CHLOROFORM-D | | | ***Sweep Width (Hz)*** | 75187.97 |
| ***Temperature (degree C)*** | 27.000 | | | | | | |

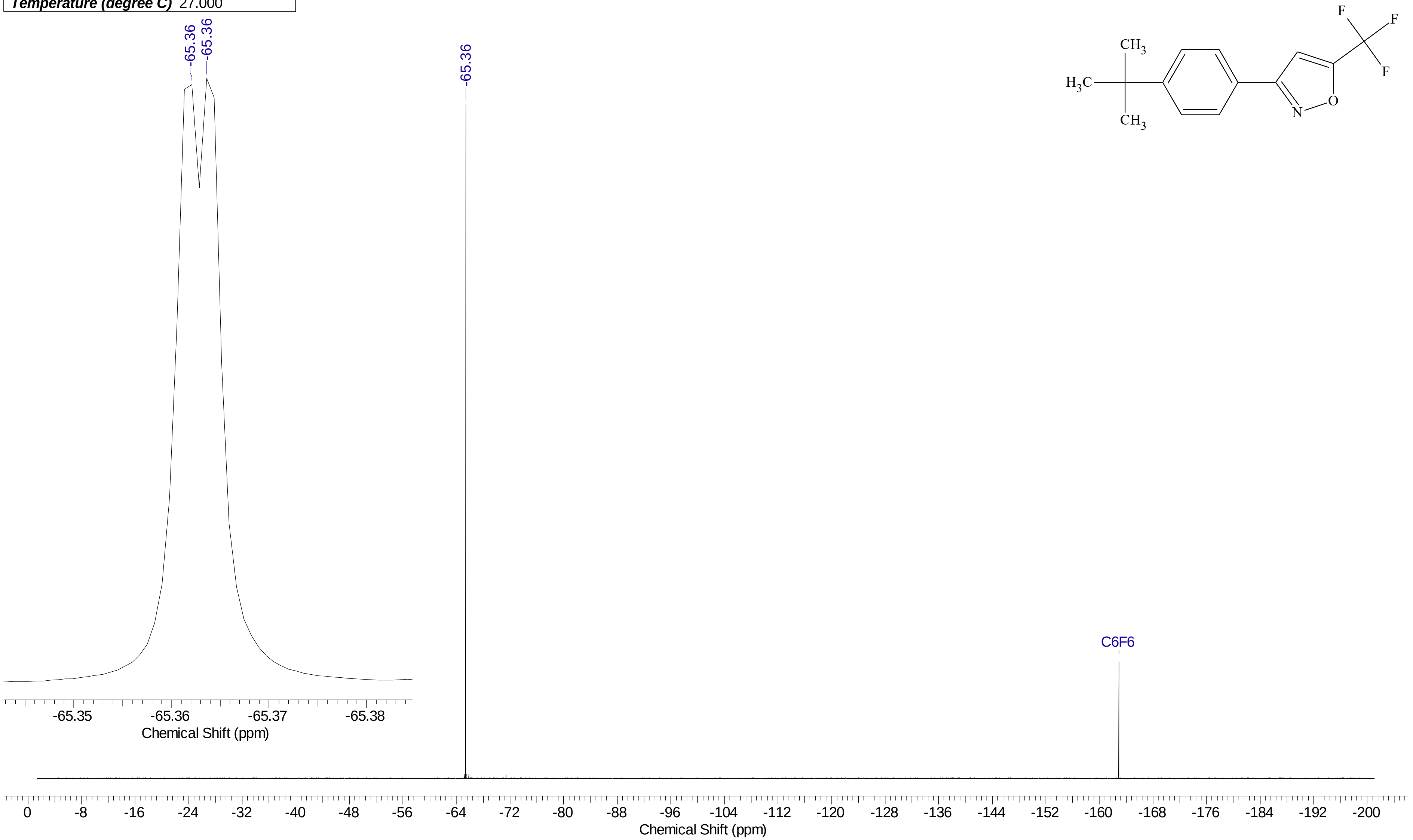

$^{19}$F NMR spectrum of **3h** (376.5 MHz, $CDCl_3$)

3 Aug 2022

| | | | | | | | |
|---|---|---|---|---|---|---|---|
| ***Acquisition Time (sec)*** | 0.6783 | ***Comment*** | Imported from UXNMR. | | | ***Date*** | 20 Jan 2022 12:08:18 |
| ***File Name*** | C:\DOCS\OUTPUT_301\2022\01. 礅図黒BM-2404-3.C_002001r | | | | | ***Frequency (MHz)*** | 100.61 |
| ***Nucleus*** | 13C | ***Number of Transients*** | 637 | ***Original Points Count*** | 16384 | ***Points Count*** | 131072 |
| ***Pulse Sequence*** | zgpg30 | ***Solvent*** | CHLOROFORM-D | | | ***Sweep Width (Hz)*** | 24154.59 |
| ***Temperature (degree C)*** | 27.000 | | | | | | |

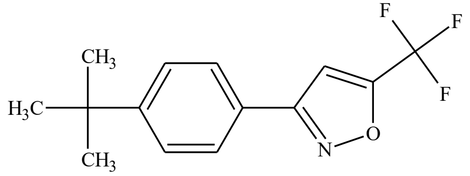

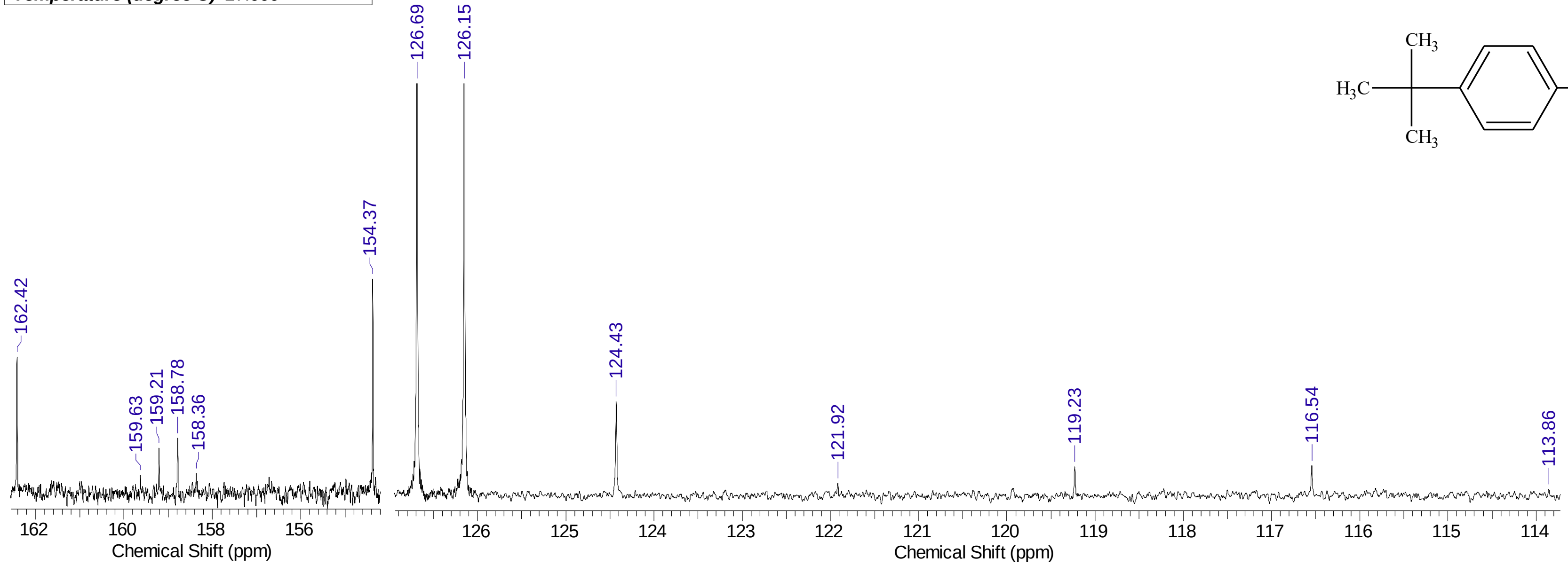


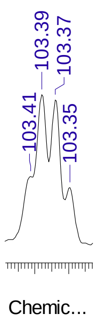

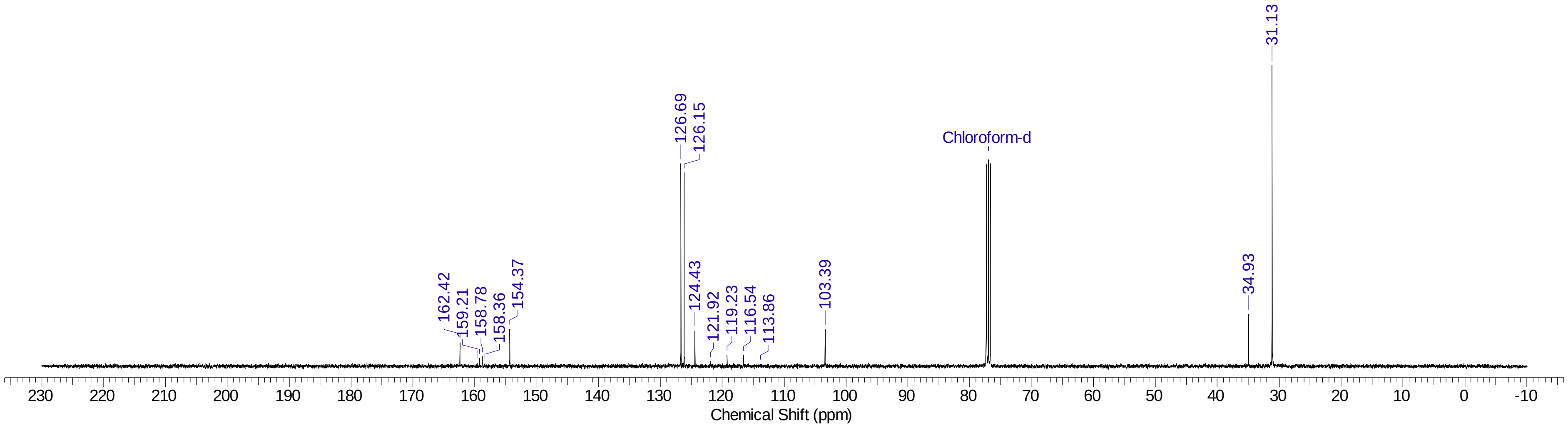

$^{13}$C NMR spectrum of **3h** (100.6 MHz, $CDCl_3$)

3 Aug 2022

| | | | | | | | |
|---|---|---|---|---|---|---|---|
| ***Acquisition Time (sec)*** | 4.0894 | ***Comment*** | Imported from UXNMR. | | | ***Date*** | 24 Dec 2021 15:40:48 |
| ***File Name*** | C:\DOCS\OUTPUT_301\2021\12.溴赅狃黒BM-2400-1.H_001001r | | | | | ***Frequency (MHz)*** | 400.13 |
| ***Nucleus*** | 1H | ***Number of Transients*** | 4 | ***Original Points Count*** | 32768 | ***Points Count*** | 131072 |
| ***Pulse Sequence*** | zg30 | ***Solvent*** | CHLOROFORM-D | | | ***Sweep Width (Hz)*** | 8012.82 |
| ***Temperature (degree C)*** | 27.000 | | | | | | |

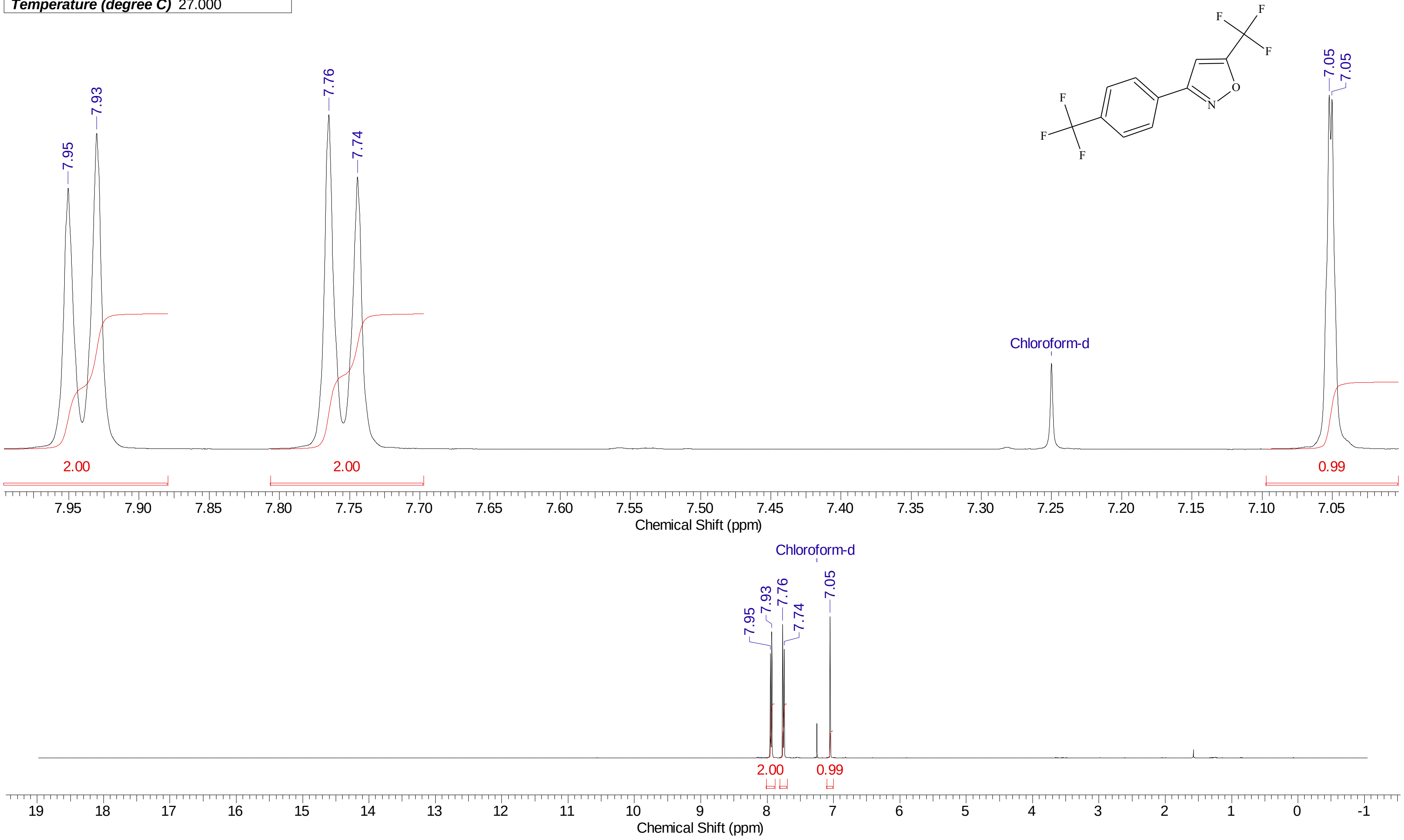

$^{1}H$ NMR spectrum of **3i** (400.1 MHz, $CDCl_3$)

3 Aug 2022

| | | | | | |
|---|---|---|---|---|---|
| ***Acquisition Time (sec)*** | 1.7433 | ***Comment*** | Imported from UXNMR. | ***Date*** | 24 Dec 2021 15:39:16 |
| ***File Name*** | C:\DOCS\OUTPUT_301\2021\12.溴贬狃黒BM-2400-1.F_005001r | | | ***Frequency (MHz)*** | 376.50 |
| ***Nucleus*** | 19F | ***Number of Transients*** | 9 ***Original Points Count*** 131072 | ***Points Count*** | 262144 |
| ***Pulse Sequence*** | zgflqn | ***Solvent*** | CHLOROFORM-D | ***Sweep Width (Hz)*** | 75187.97 |
| ***Temperature (degree C)*** | 27.000 | | | | |

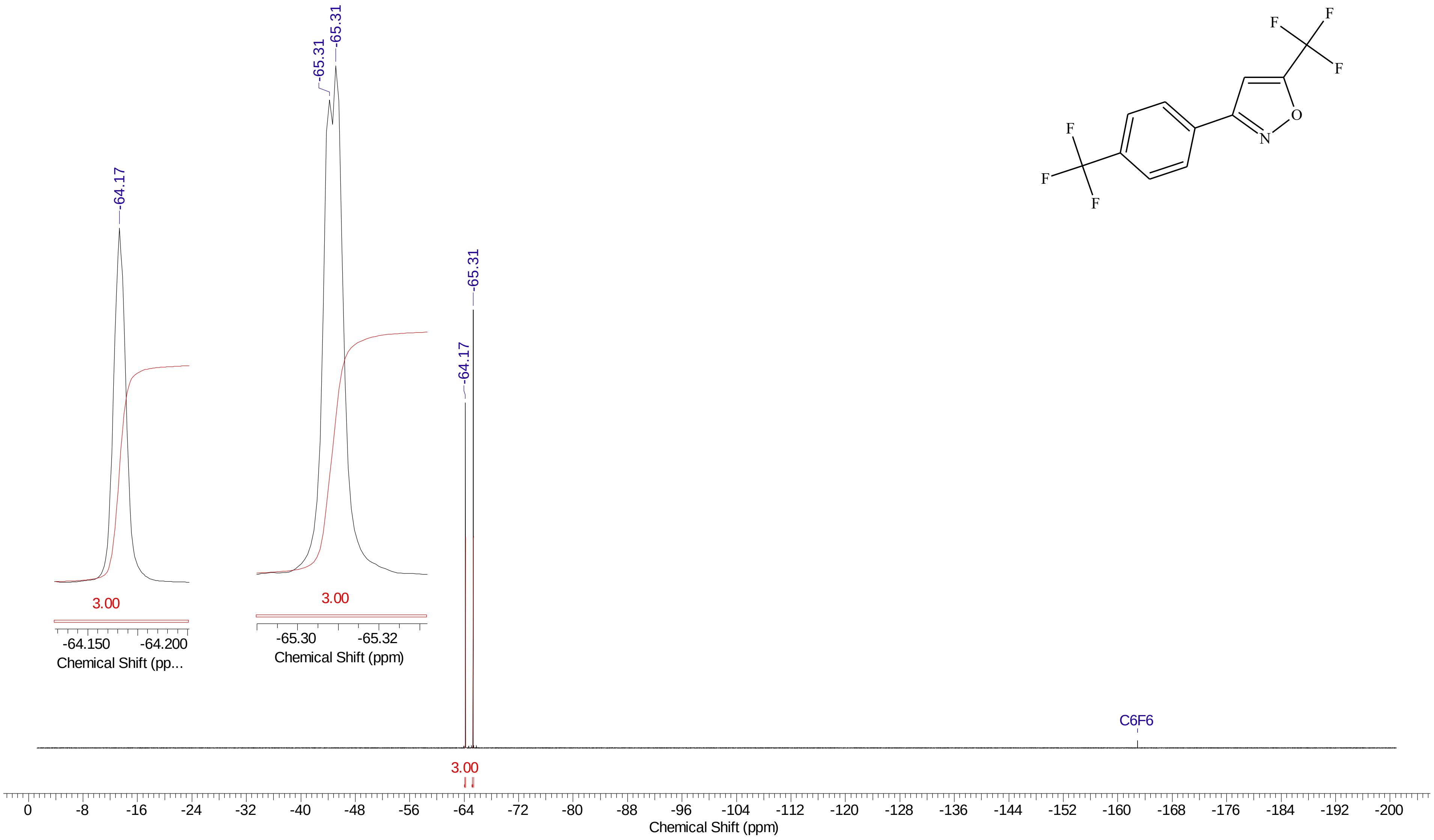

$^{19}$F NMR spectrum of **3i** (376.5 MHz, $CDCl_3$)

3 Aug 2022

| Acquisition Time (sec) | 0.6783 | Comment | Imported from UXNMR. | | | Date | 24 Dec 2021 16:51:54 |
|---|---|---|---|---|---|---|---|
| File Name | C:\DOCS\OUTPUT_301\2021\12.溴啶犸黒BM-2400-1.C_002001r | | | | | Frequency (MHz) | 100.61 |
| Nucleus | 13C | Number of Transients | 186 | Original Points Count | 16384 | Points Count | 131072 |
| Pulse Sequence | zgpg30 | Solvent | CHLOROFORM-D | | | Sweep Width (Hz) | 24154.59 |
| Temperature (degree C) | 27.000 | | | | | | |

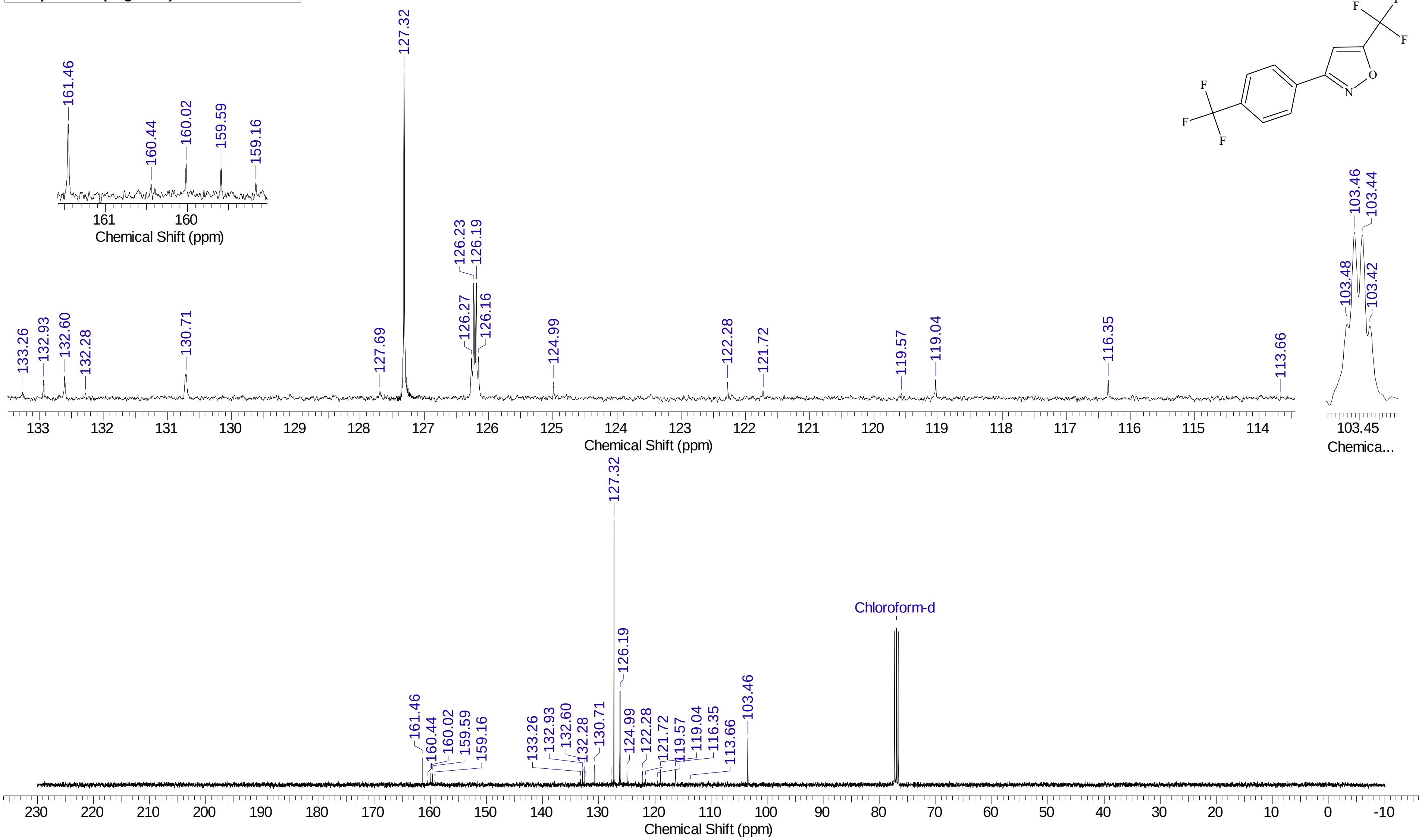

$^{13}$C NMR spectrum of **3i** (100.6 MHz, $CDCl_3$)

3 Aug 2022

| Acquisition Time (sec) | 4.0894 | Comment | Imported from UXNMR. | | | Date | 12 Jul 2022 15:13:36 |
|---|---|---|---|---|---|---|---|
| File Name | C:\BM_DATA\DOCS\12.07.22\12.07.22\SZA-BM-2554.H_001001r | | | | | Frequency (MHz) | 400.13 |
| Nucleus | 1H | Number of Transients | 4 | Original Points Count | 32768 | Points Count | 131072 |
| Pulse Sequence | zg30 | Solvent | CHLOROFORM-D | | | Sweep Width (Hz) | 8012.82 |
| Temperature (degree C) | 27.000 | | | | | | |

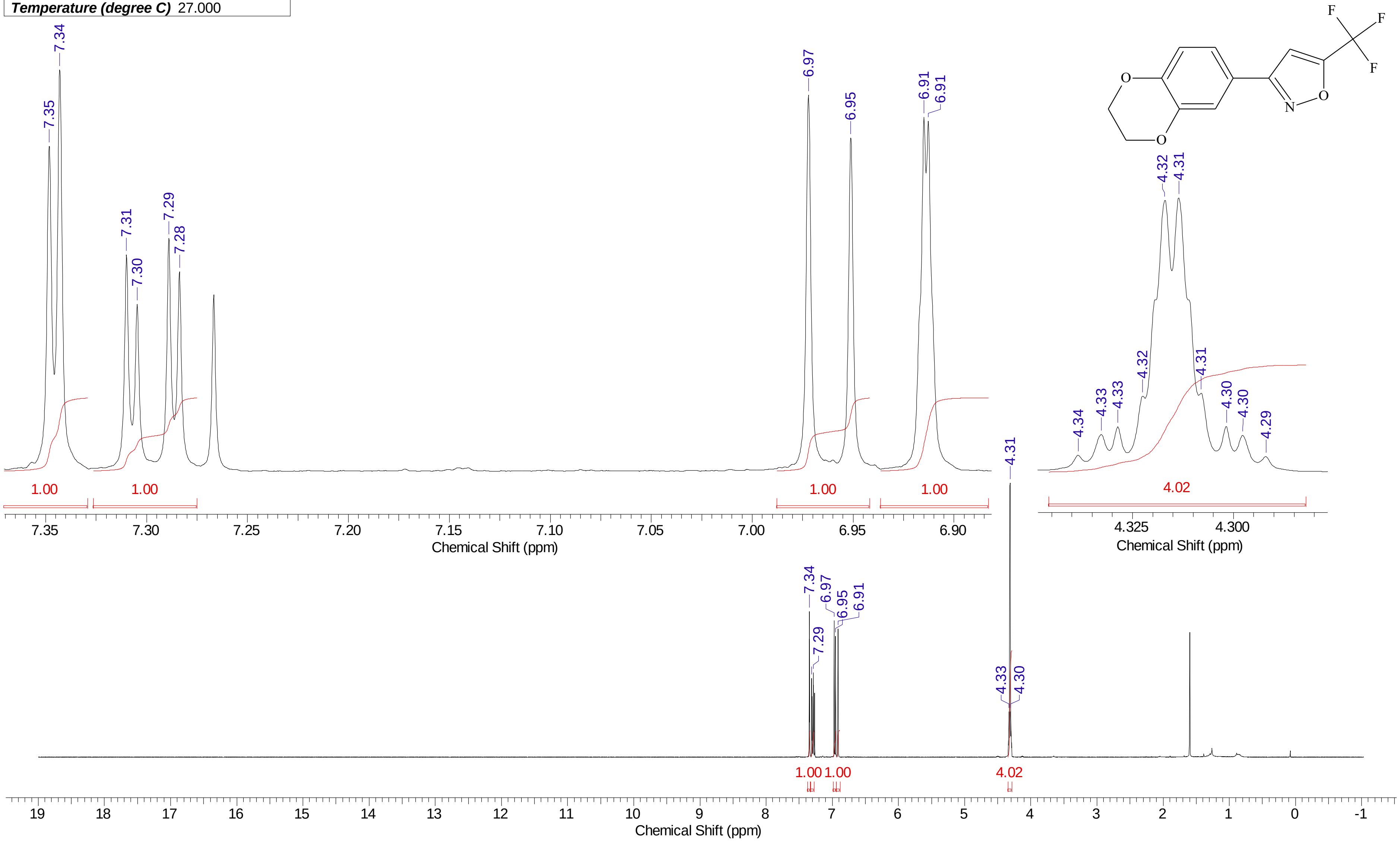

$^{1}H$ NMR spectrum of **3k** (400.1 MHz, $CDCl_3$)

3 Aug 2022

| Acquisition Time (sec) | 1.7433 | Comment | Imported from UXNMR. | | | Date | 12 Jul 2022 15:17:18 |
| --- | --- | --- | --- | --- | --- | --- | --- |
| File Name | C:\BM_DATA\DOCS\12.07.22\12.07.22\SZA-BM-2554.F_005001r | | | | | Frequency (MHz) | 376.50 |
| Nucleus | 19F | Number of Transients | 16 | Original Points Count | 131072 | Points Count | 262144 |
| Pulse Sequence | zgflqn | Solvent | CHLOROFORM-D | | | Sweep Width (Hz) | 75187.97 |
| Temperature (degree C) | 27.000 | | | | | | |

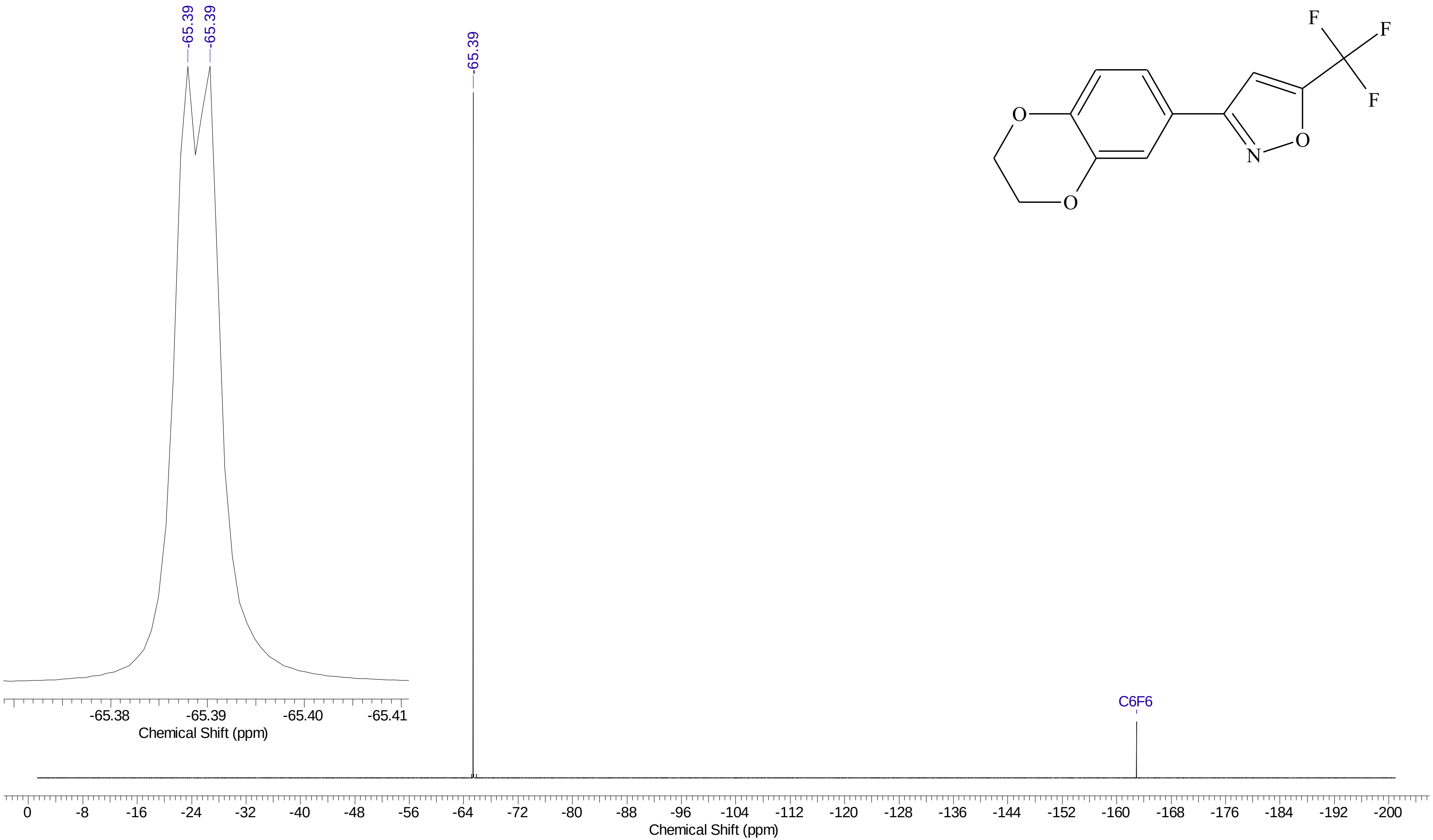

# $^{19}$F NMR spectrum of **3k** (376.5 MHz, $CDCl_3$)

3 Aug 2022

| | | | | | | | |
|---|---|---|---|---|---|---|---|
| ***Acquisition Time (sec)*** | 0.6783 | ***Comment*** | Imported from UXNMR. | | | ***Date*** | 14 Jul 2022 15:58:04 |
| ***File Name*** | C:\BM_DATA\DOCS\14.07.22\14.07.22\SZA-BM-2554.C_002001r | | | | | ***Frequency (MHz)*** | 100.61 |
| ***Nucleus*** | 13C | ***Number of Transients*** | 1154 | ***Original Points Count*** | 16384 | ***Points Count*** | 131072 |
| ***Pulse Sequence*** | zgpg30 | ***Solvent*** | CHLOROFORM-D | | | ***Sweep Width (Hz)*** | 24154.59 |
| ***Temperature (degree C)*** | 27.000 | | | | | | |

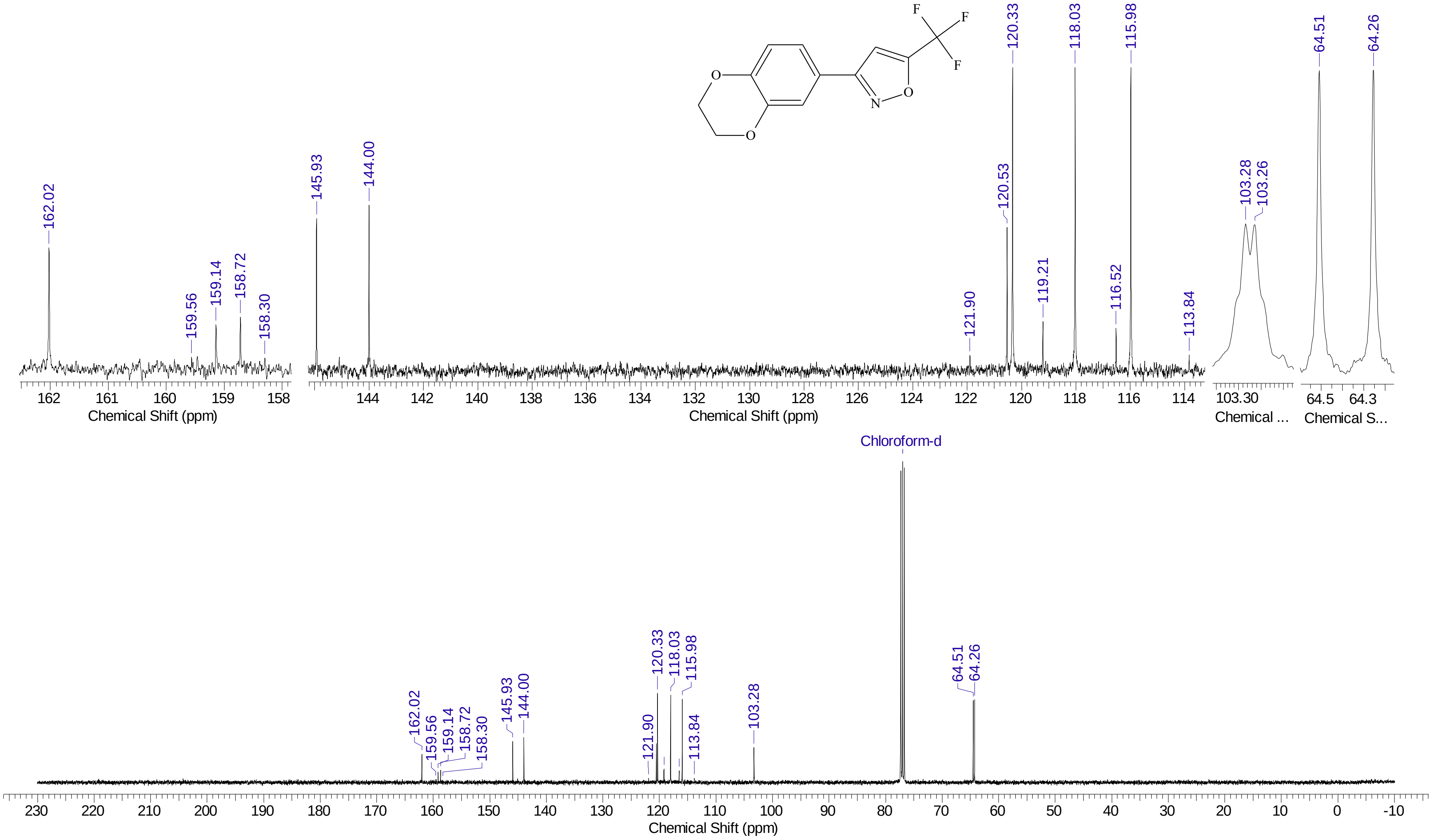

$^{13}$C NMR spectrum of **3k** (100.6 MHz, $CDCl_3$)

3 Aug 2022

| | | | | | | | |
|---|---|---|---|---|---|---|---|
| ***Acquisition Time (sec)*** | 4.0894 | ***Comment*** | Imported from UXNMR. | | | ***Date*** | 17 Jan 2022 15:12:28 |
| ***File Name*** | C:\DOCS\OUTPUT_301\2022\01. 磯囼黒BM-2401.H_001001r | | | | | ***Frequency (MHz)*** | 400.13 |
| ***Nucleus*** | 1H | ***Number of Transients*** | 4 | ***Original Points Count*** | 32768 | ***Points Count*** | 131072 |
| ***Pulse Sequence*** | zg30 | ***Solvent*** | BENZENE-D6 | ***Sweep Width (Hz)*** | 8012.82 | ***Temperature (degree C)*** | 27.000 |

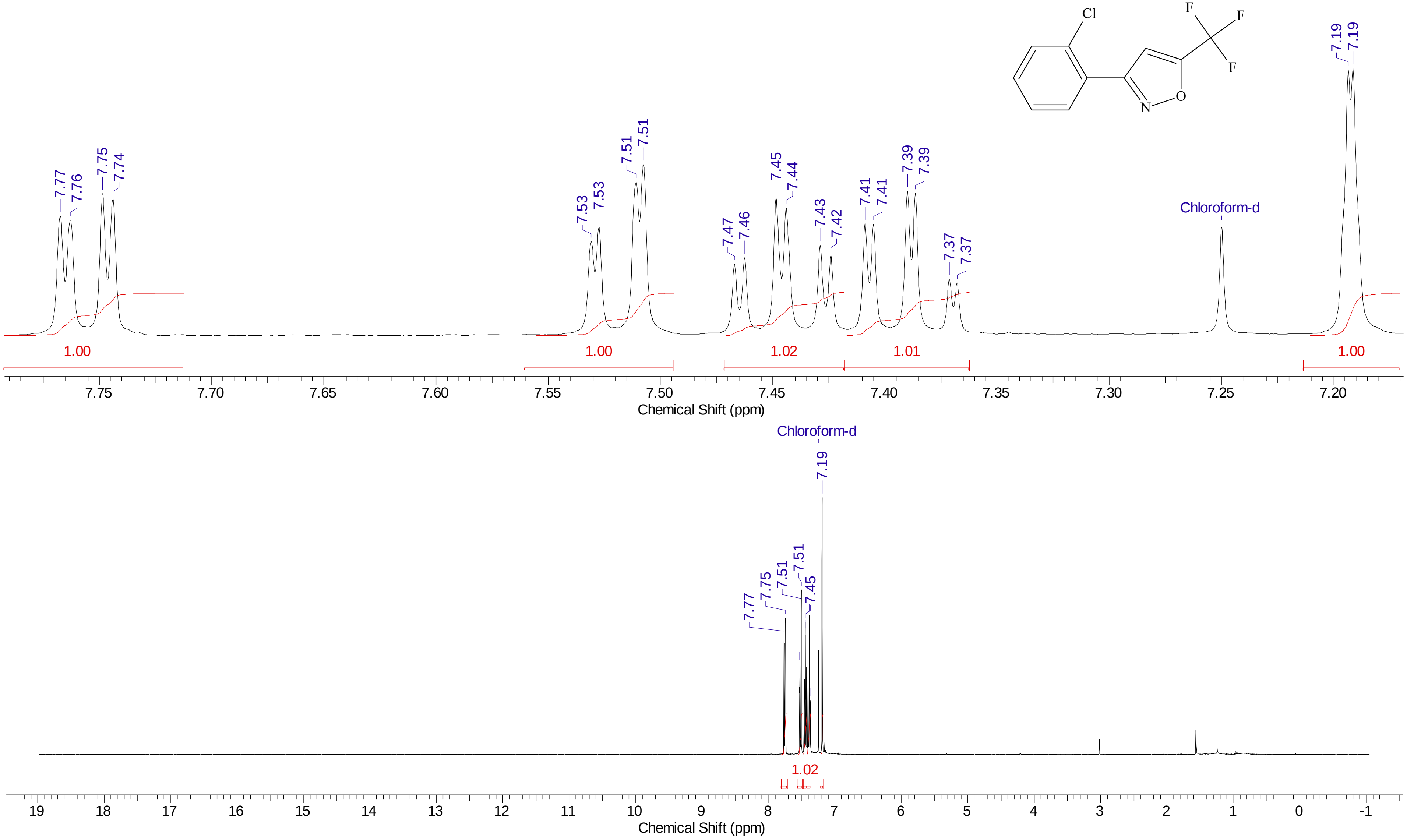

# $^1$H NMR spectrum of **3l** (400.1 MHz, $CDCl_3$)

3 Aug 2022

| | | | | | | | |
|---|---|---|---|---|---|---|---|
| ***Acquisition Time (sec)*** | 1.7433 | ***Comment*** | Imported from UXNMR. | | | ***Date*** | 17 Jan 2022 15:28:34 |
| ***File Name*** | C:\DOCS\OUTPUT_301\2022\01. 磤囵黒BM-2401.F_005001r | | | ***Frequency (MHz)*** | 376.50 | ***Nucleus*** | 19F |
| ***Number of Transients*** | 13 | ***Original Points Count*** | 131072 | ***Points Count*** | 262144 | ***Pulse Sequence*** | zgflqn |
| ***Solvent*** | CHLOROFORM-D | | | ***Sweep Width (Hz)*** | 75187.97 | ***Temperature (degree C)*** | 27.000 |

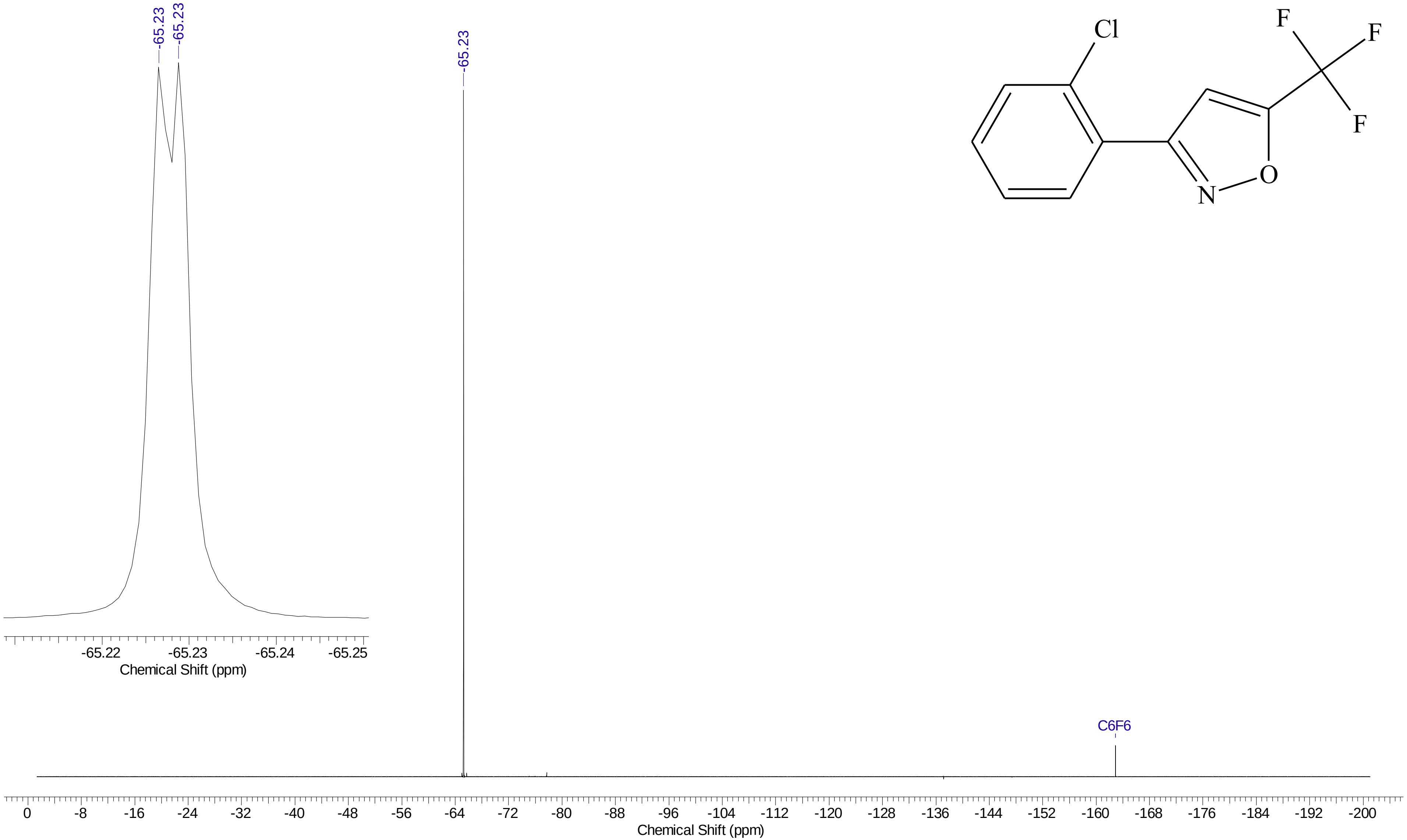

## $^{19}$F NMR spectrum of **3l** (376.5 MHz, $CDCl_3$)

3 Aug 2022

| | | | | | | | |
|---|---|---|---|---|---|---|---|
| ***Acquisition Time (sec)*** | 0.6783 | ***Comment*** | Imported from UXNMR. | | | ***Date*** | 18 Jan 2022 16:05:08 |
| ***File Name*** | C:\DOCS\OUTPUT_301\2022\01. 磁図黒BM-2401.C_002001r | | | ***Frequency (MHz)*** | 100.61 | ***Nucleus*** | 13C |
| ***Number of Transients*** | 364 | ***Original Points Count*** | 16384 | ***Points Count*** | 131072 | ***Pulse Sequence*** | zgpg30 |
| ***Solvent*** | CHLOROFORM-D | | | ***Sweep Width (Hz)*** | 24154.59 | ***Temperature (degree C)*** | 27.000 |

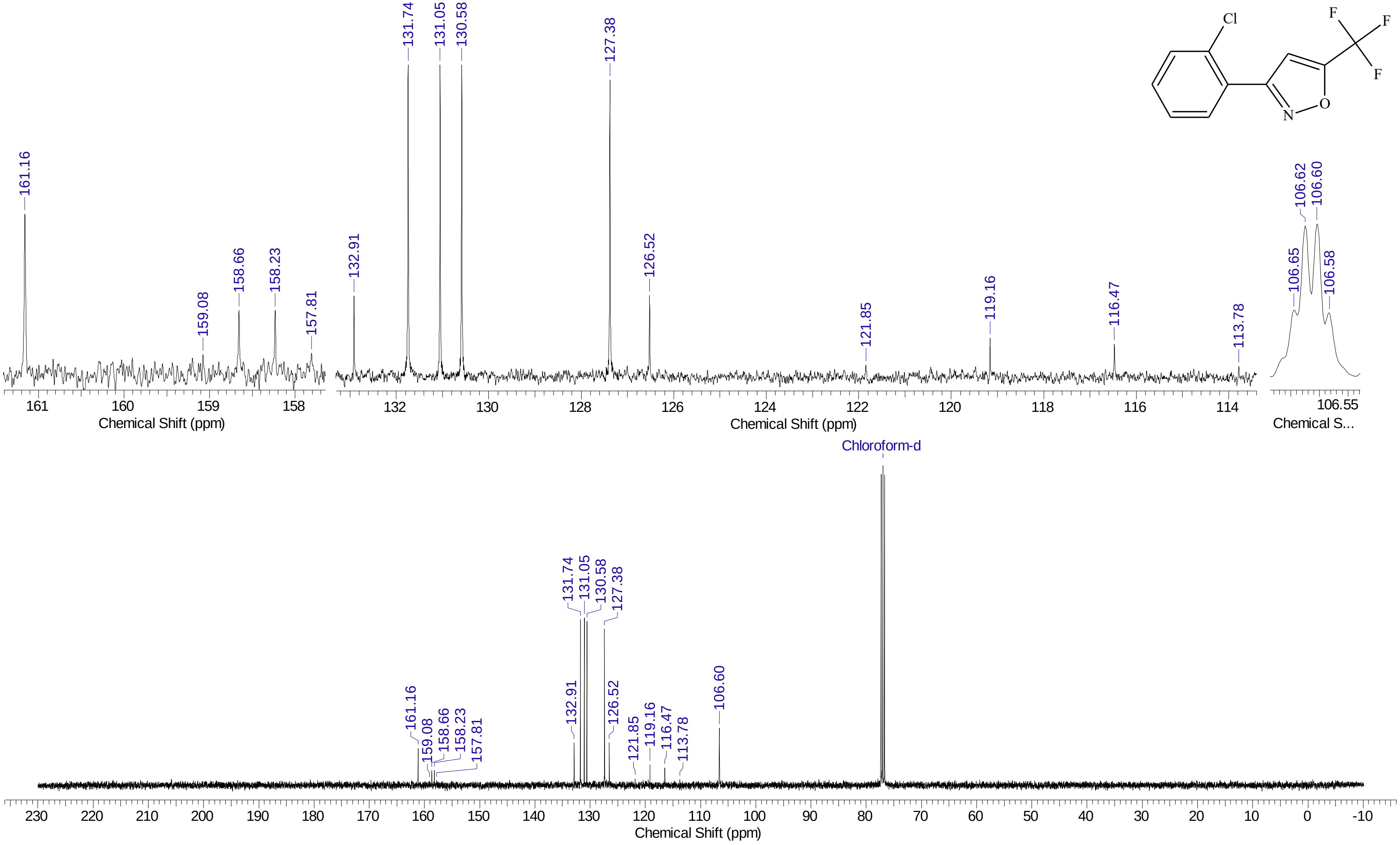

$^{13}C$ NMR spectrum of **3l** (100.6 MHz, $CDCl_3$)

3 Aug 2022

| | | | | | | | |
|---|---|---|---|---|---|---|---|
| ***Acquisition Time (sec)*** | 4.0894 | ***Comment*** | Imported from UXNMR. | | | ***Date*** | 19 Jan 2022 17:43:48 |
| ***File Name*** | C:\DOCS\OUTPUT_301\2022\01. 磤囹黒BM-2403-p.H_001001r | | | | | ***Frequency (MHz)*** | 400.13 |
| ***Nucleus*** | 1H | ***Number of Transients*** | 4 | ***Original Points Count*** | 32768 | ***Points Count*** | 131072 |
| ***Pulse Sequence*** | zg30 | ***Solvent*** | BENZENE-D6 | ***Sweep Width (Hz)*** | 8012.82 | ***Temperature (degree C)*** | 27.000 |

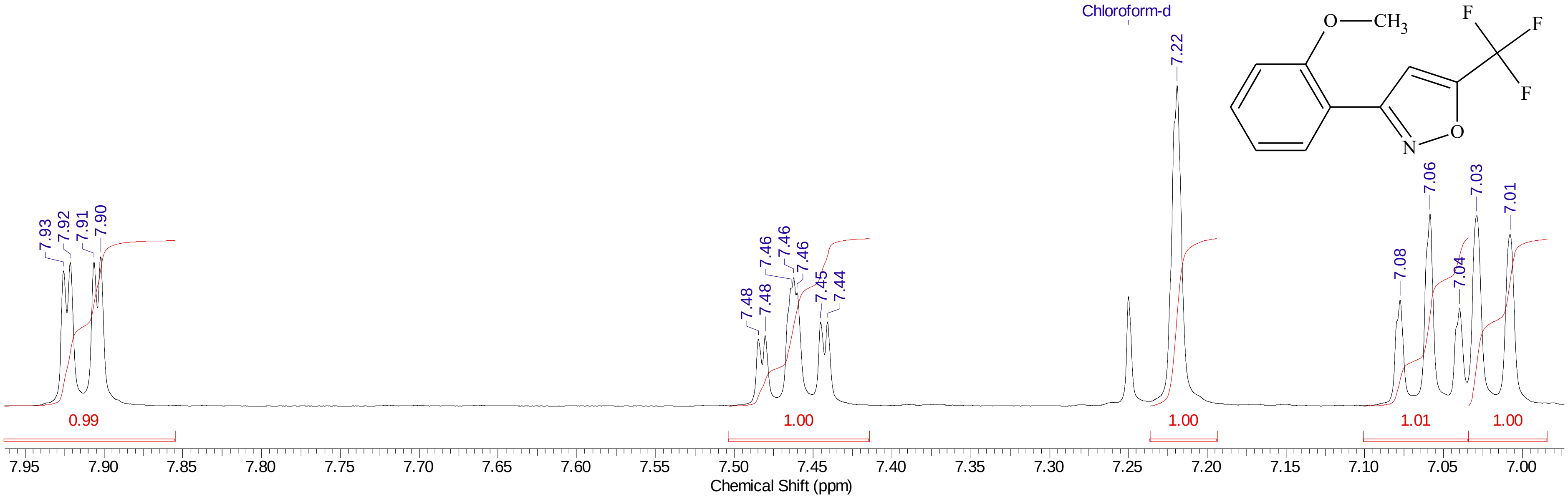


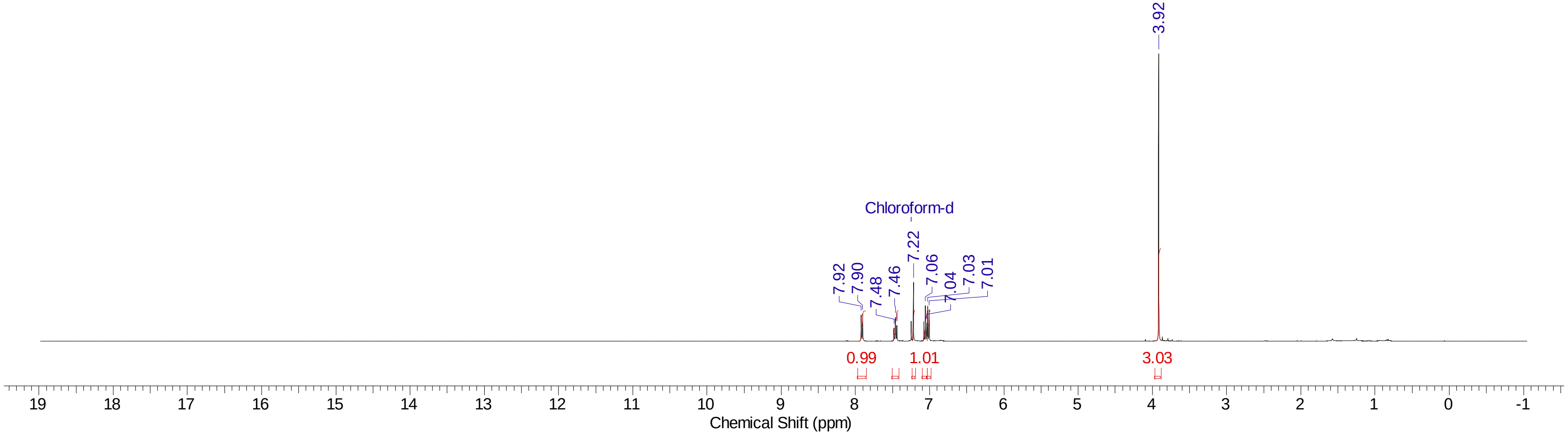

# $^{1}$H NMR spectrum of **3m** (400.1 MHz, $CDCl_3$)

3 Aug 2022

| | | | | | | | |
|---|---|---|---|---|---|---|---|
| ***Acquisition Time (sec)*** | 1.7433 | ***Comment*** | Imported from UXNMR. | | | ***Date*** | 17 Jan 2022 15:32:46 |
| ***File Name*** | C:\DOCS\OUTPUT_301\2022\01. 磝囪黒BM-2403.F_005001r | | | ***Frequency (MHz)*** | 376.50 | ***Nucleus*** | 19F |
| ***Number of Transients*** | 12 | ***Original Points Count*** | 131072 | ***Points Count*** | 262144 | ***Pulse Sequence*** | zgflqn |
| ***Solvent*** | CHLOROFORM-D | | | ***Sweep Width (Hz)*** | 75187.97 | ***Temperature (degree C)*** | 27.000 |

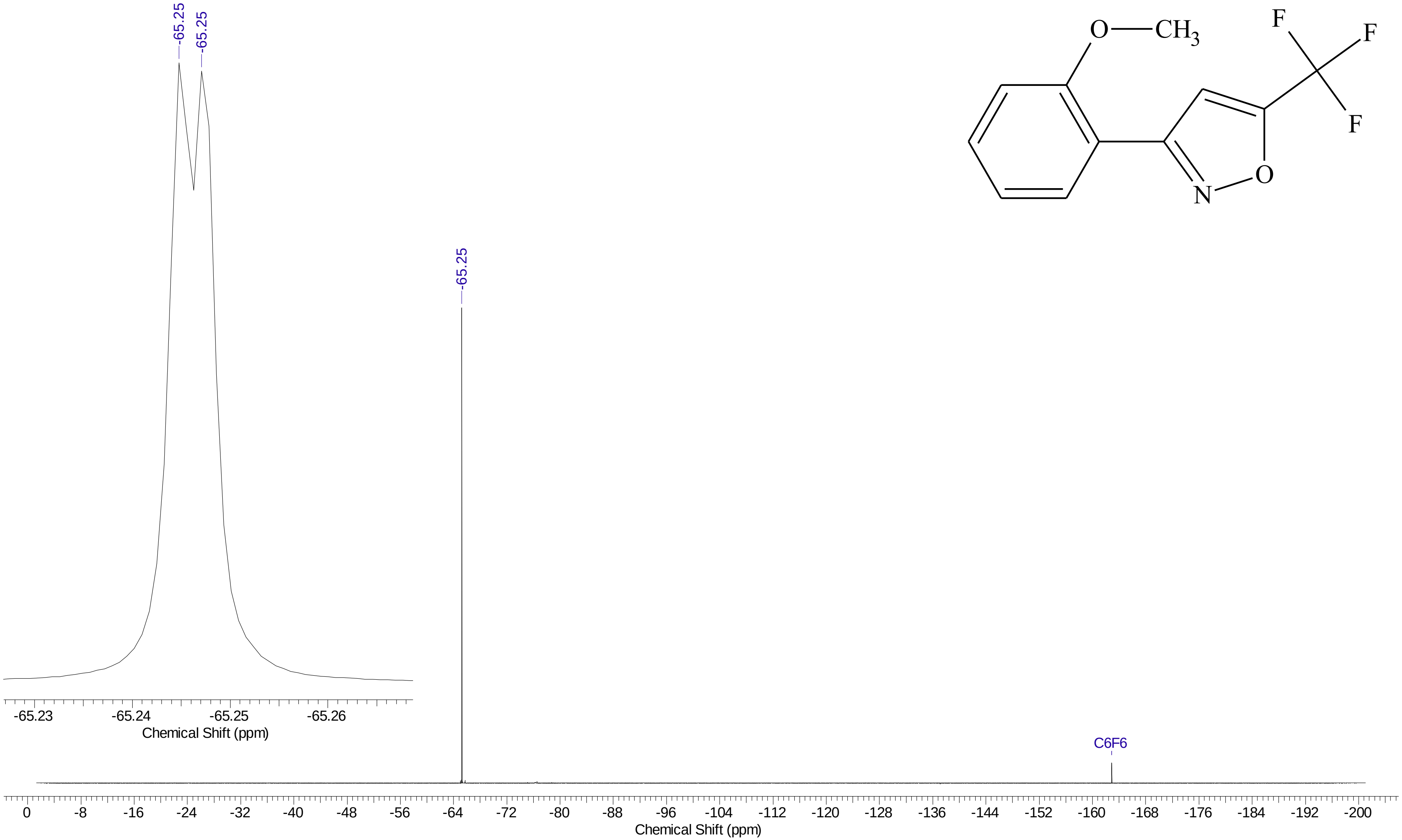

$^{19}$F NMR spectrum of **3m** (376.5 MHz, $CDCl_3$)

3 Aug 2022

| | | | | | | | |
|---|---|---|---|---|---|---|---|
| ***Acquisition Time (sec)*** | 0.6783 | ***Comment*** | Imported from UXNMR. | | | ***Date*** | 20 Jan 2022 12:24:22 |
| ***File Name*** | C:\DOCS\OUTPUT_301\2022\01. 礅図黒BM-2403-p.C_002001r | | | | | ***Frequency (MHz)*** | 100.61 |
| ***Nucleus*** | 13C | ***Number of Transients*** | 351 | ***Original Points Count*** | 16384 | ***Points Count*** | 131072 |
| ***Pulse Sequence*** | zgpg30 | ***Solvent*** | CHLOROFORM-D | | | ***Sweep Width (Hz)*** | 24154.59 |
| ***Temperature (degree C)*** | 27.000 | | | | | | |

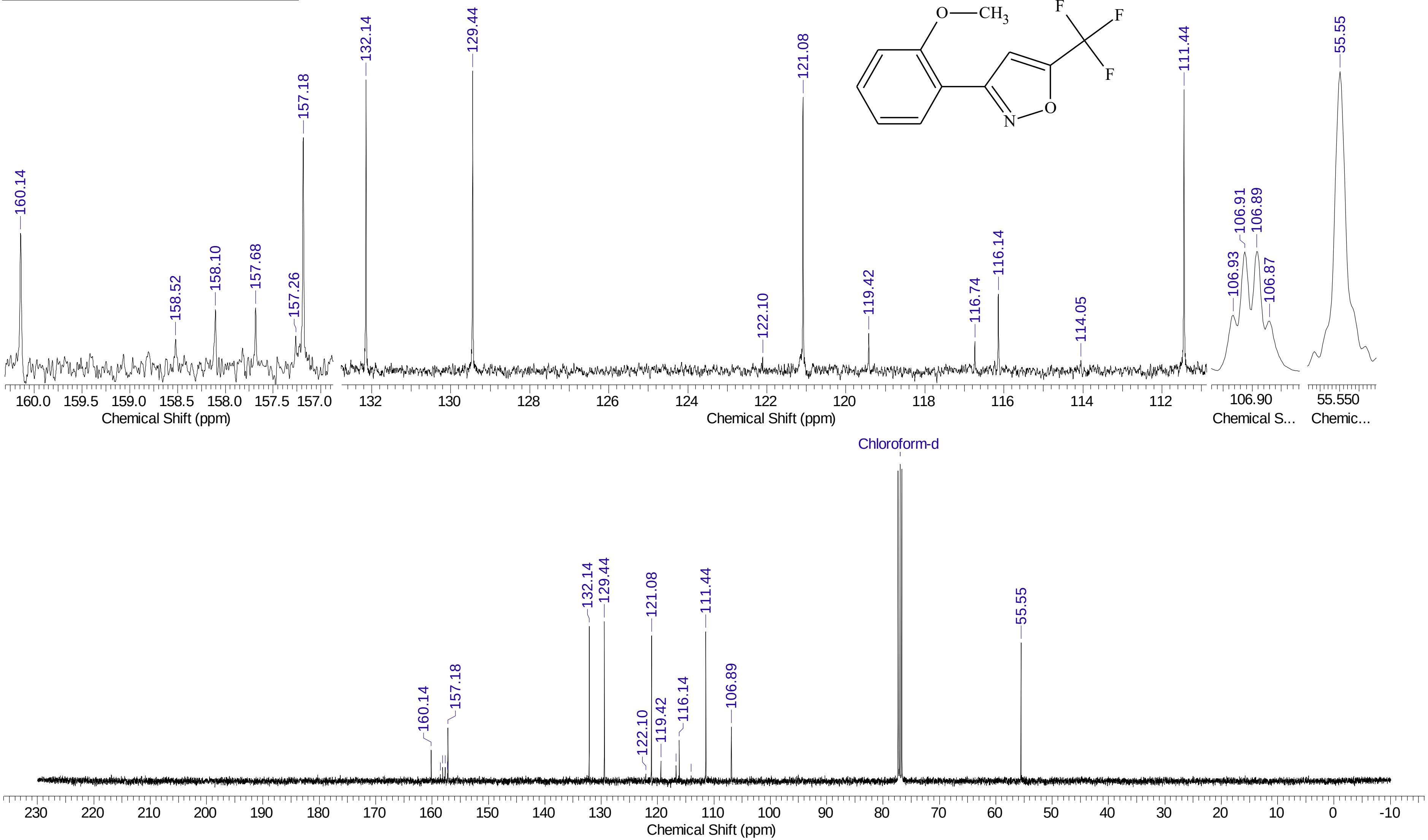

## $^{13}$C NMR spectrum of **3m** (100.6 MHz, $CDCl_3$)

3 Aug 2022

| | | | | | |
|---|---|---|---|---|---|
| ***Acquisition Time (sec)*** | 4.0894 | ***Comment*** | Imported from UXNMR. | ***Date*** | 21 Jan 2022 15:00:58 |
| ***File Name*** | C:\DOCS\OUTPUT_301\2022\01. 磤囹黒BM-2412-1.H_001001r | | | ***Frequency (MHz)*** | 400.13 |
| ***Nucleus*** | 1H | ***Number of Transients*** | 4 | ***Original Points Count*** 32768 | ***Points Count*** 131072 |
| ***Pulse Sequence*** | zg30 | ***Solvent*** | CHLOROFORM-D | ***Sweep Width (Hz)*** | 8012.82 |
| ***Temperature (degree C)*** | 27.000 | | | | |

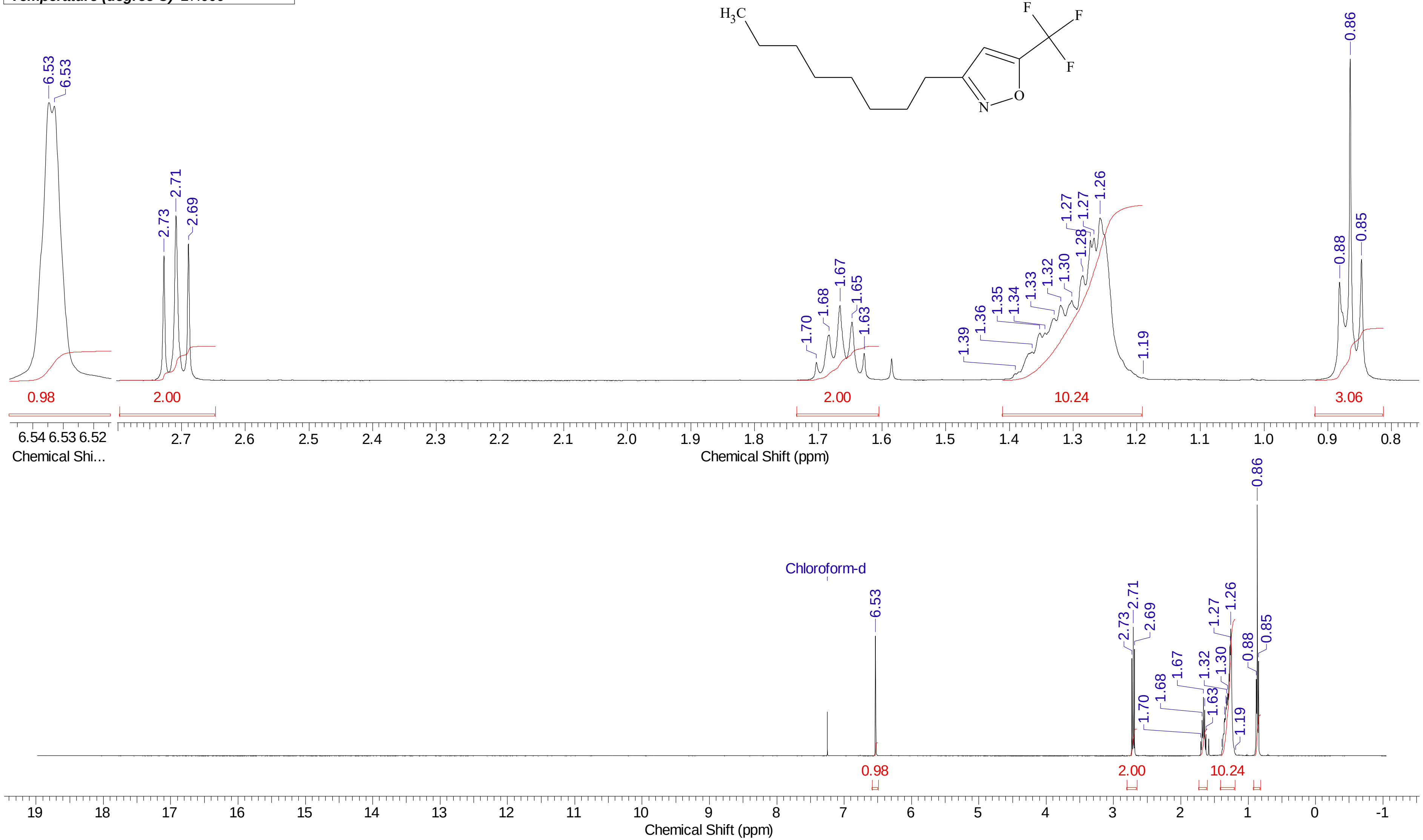

$^{1}$H NMR spectrum of **3o** (400.1 MHz, $CDCl_3$)

3 Aug 2022

| | | | | | | | |
|---|---|---|---|---|---|---|---|
| ***Acquisition Time (sec)*** | 1.7433 | ***Comment*** | Imported from UXNMR. | | | ***Date*** | 21 Jan 2022 15:20:12 |
| ***File Name*** | C:\DOCS\OUTPUT_301\2022\01. 磩囵黒BM-2412-1.F_005001r | | | | | ***Frequency (MHz)*** | 376.50 |
| ***Nucleus*** | 19F | ***Number of Transients*** | 8 | ***Original Points Count*** | 131072 | ***Points Count*** | 262144 |
| ***Pulse Sequence*** | zgflqn | ***Solvent*** | Acetone | ***Sweep Width (Hz)*** | 75187.97 | ***Temperature (degree C)*** | 27.000 |

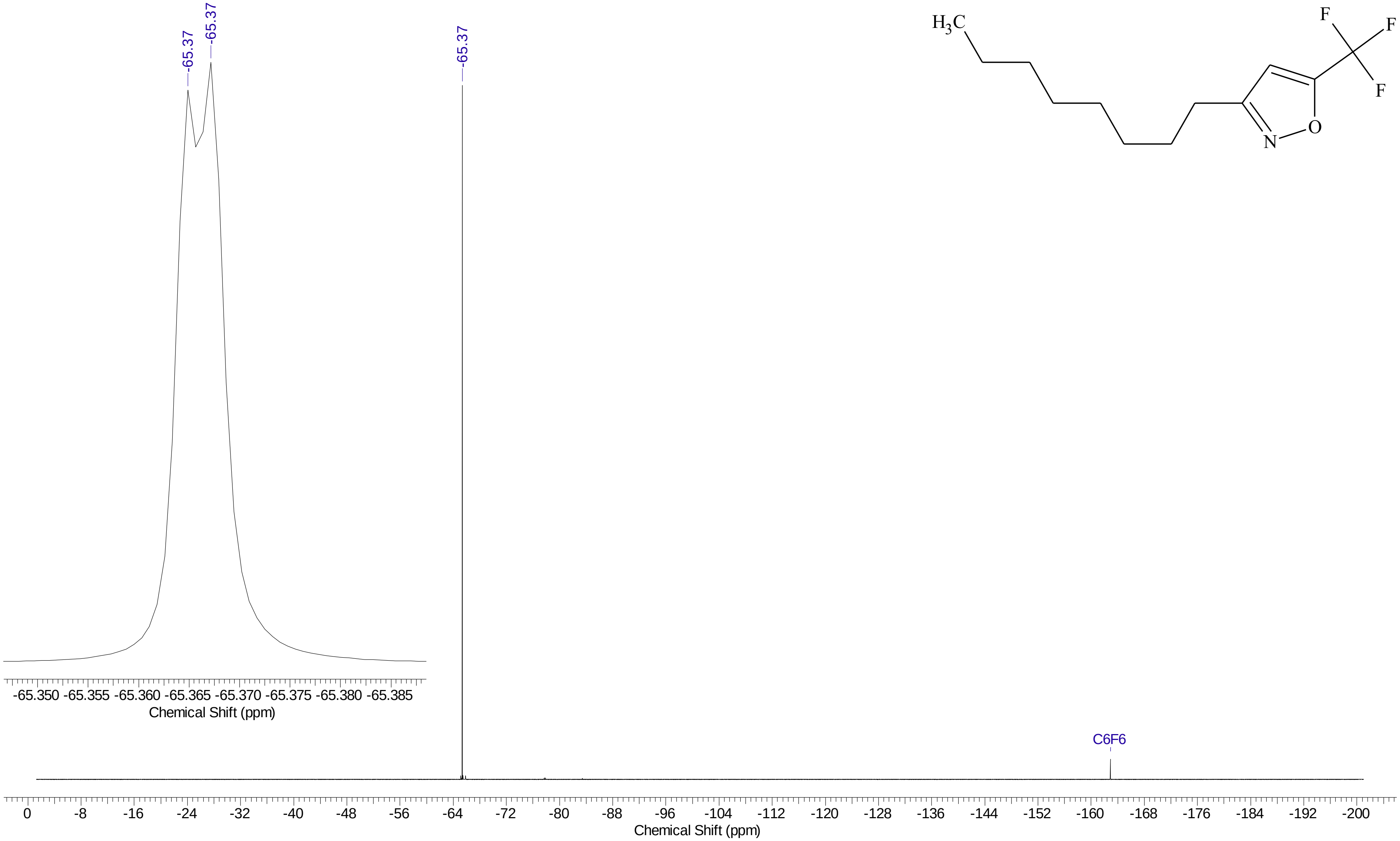

## $^{19}$F NMR spectrum of **3o** (376.5 MHz, $CDCl_3$)

3 Aug 2022

| | | | | | | | |
|---|---|---|---|---|---|---|---|
| ***Acquisition Time (sec)*** | 0.6783 | ***Comment*** | Imported from UXNMR. | | | ***Date*** | 24 Jan 2022 14:48:52 |
| ***File Name*** | C:\DOCS\OUTPUT_301\2022\01. 磤囵黒BM-2412-1.C_002001r | | | | | ***Frequency (MHz)*** | 100.61 |
| ***Nucleus*** | 13C | ***Number of Transients*** | 257 | ***Original Points Count*** | 16384 | ***Points Count*** | 131072 |
| ***Pulse Sequence*** | zgpg30 | ***Solvent*** | CHLOROFORM-D | | | ***Sweep Width (Hz)*** | 24154.59 |
| ***Temperature (degree C)*** | 27.000 | | | | | | |

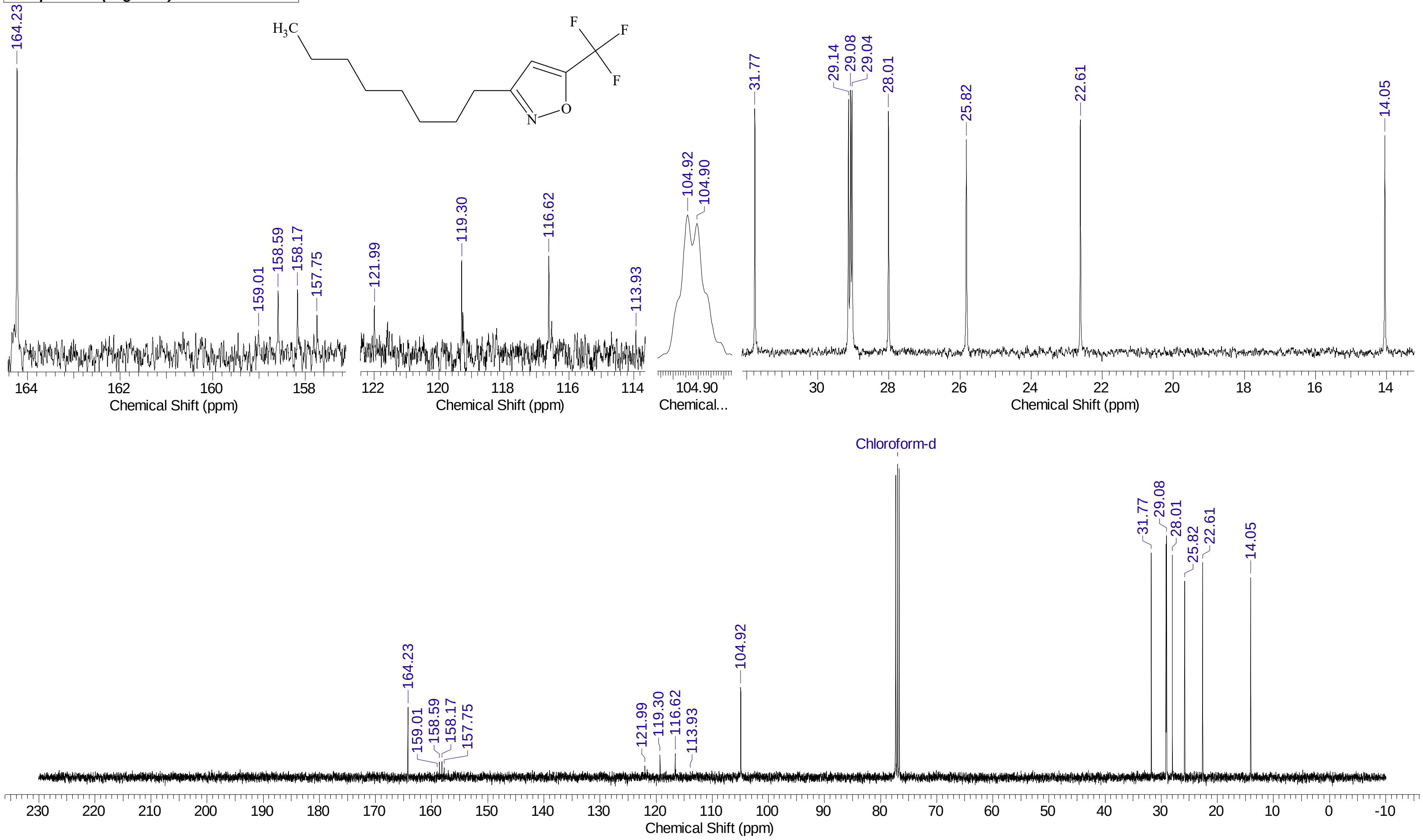

## $^{13}$C NMR spectrum of **3o** (100.6 MHz, $CDCl_3$)

3 Aug 2022

| | | | | | | | |
|---|---|---|---|---|---|---|---|
| ***Acquisition Time (sec)*** | 4.0894 | ***Comment*** | Imported from UXNMR. | | | ***Date*** | 20 Jan 2022 12:26:24 |
| ***File Name*** | C:\DOCS\OUTPUT_301\2022\01. 磡図黒BM-2411-1.H_001001r | | | | | ***Frequency (MHz)*** | 400.13 |
| ***Nucleus*** | 1H | ***Number of Transients*** | 4 | ***Original Points Count*** | 32768 | ***Points Count*** | 131072 |
| ***Pulse Sequence*** | zg30 | ***Solvent*** | BENZENE-D6 | ***Sweep Width (Hz)*** | 8012.82 | ***Temperature (degree C)*** | 27.000 |

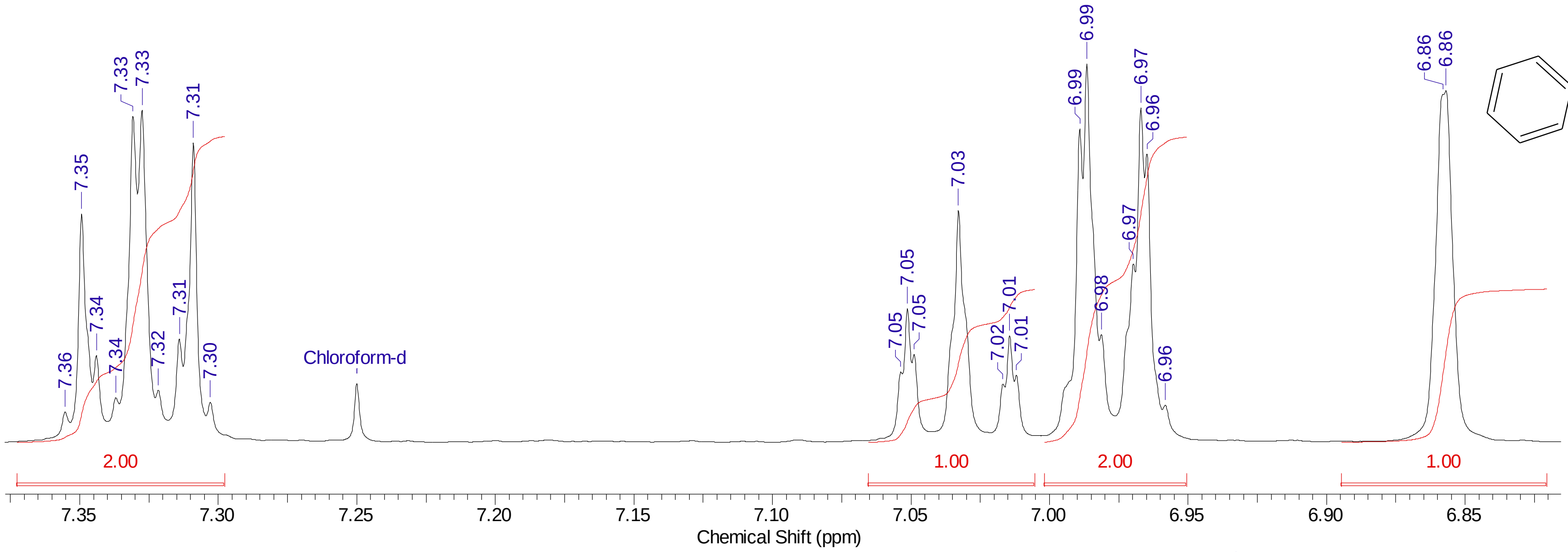


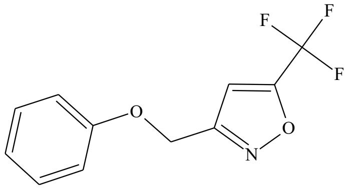


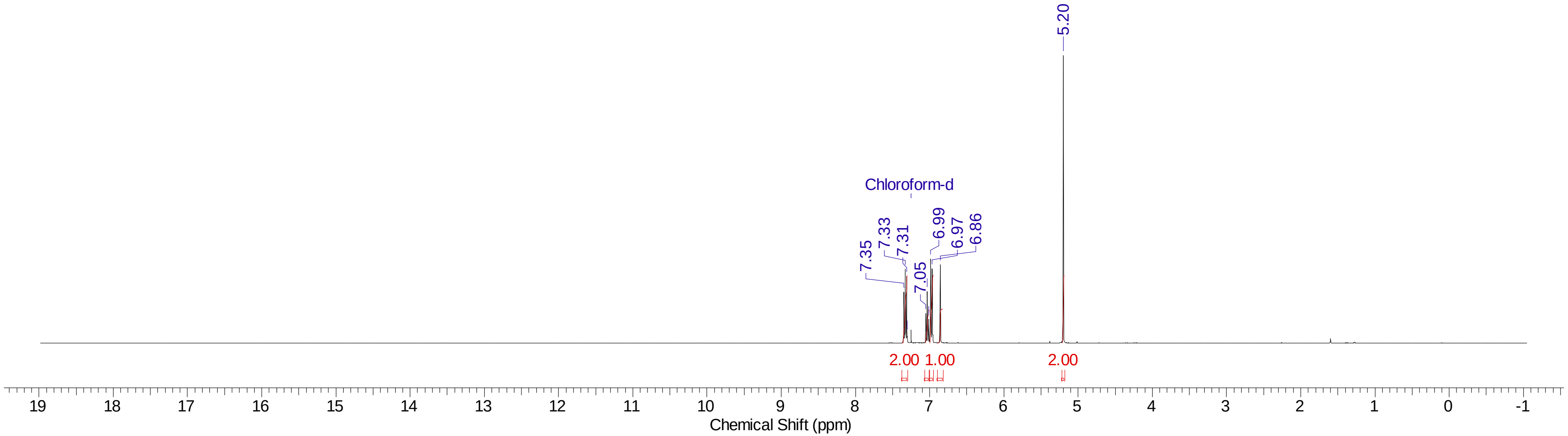

# $^{1}$H NMR spectrum of **3p** (400.1 MHz, $CDCl_3$)

3 Aug 2022

| | | | | | | | |
|---|---|---|---|---|---|---|---|
| ***Acquisition Time (sec)*** | 1.7433 | ***Comment*** | Imported from UXNMR. | | | ***Date*** | 20 Jan 2022 13:02:40 |
| ***File Name*** | C:\DOCS\OUTPUT_301\2022\01. 礅囪黒BM-2411-1.F_005001r | | | | | ***Frequency (MHz)*** | 376.50 |
| ***Nucleus*** | 19F | ***Number of Transients*** | 16 | ***Original Points Count*** | 131072 | ***Points Count*** | 262144 |
| ***Pulse Sequence*** | zgflqn | ***Solvent*** | Acetone | ***Sweep Width (Hz)*** | 75187.97 | ***Temperature (degree C)*** | 27.000 |

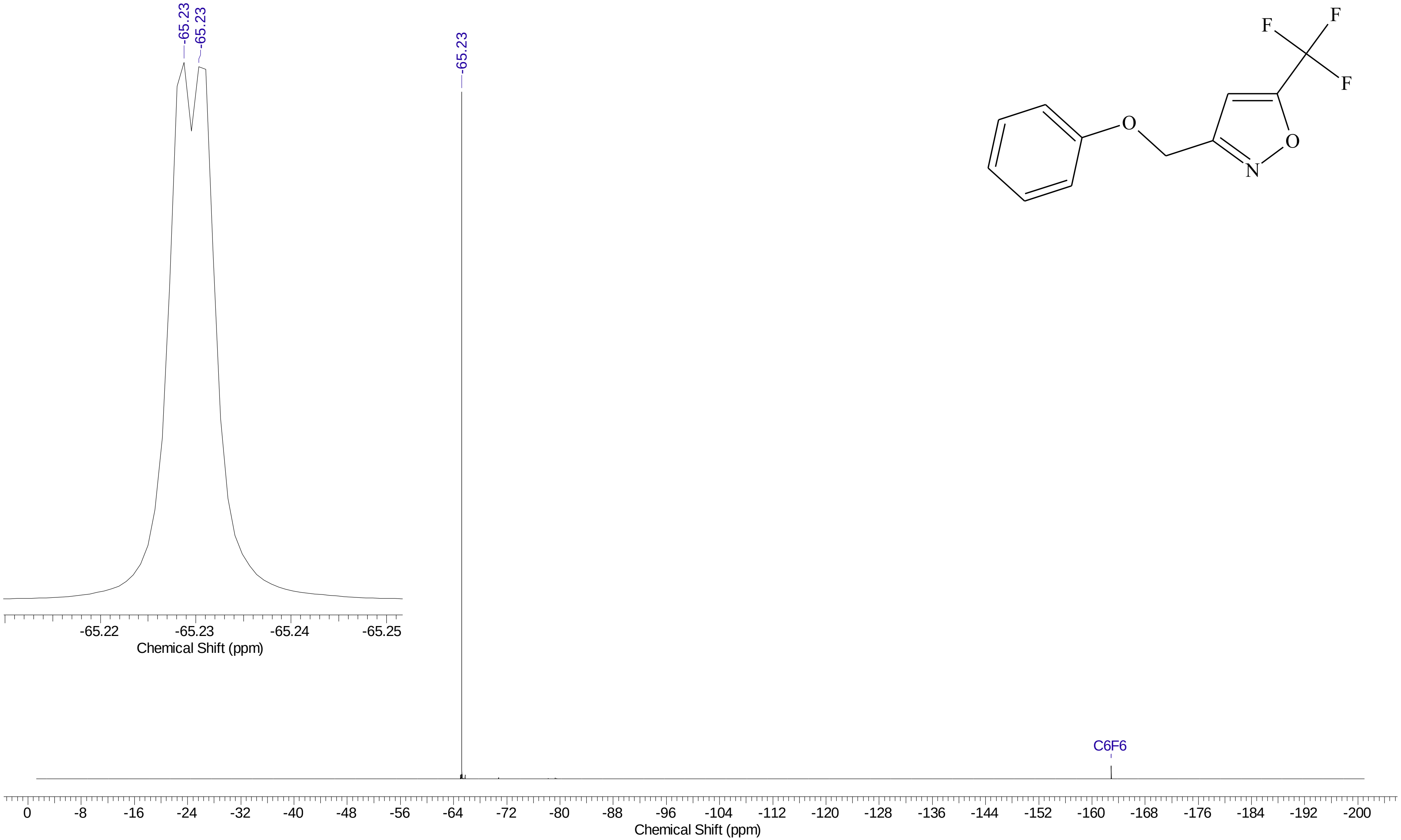

## $^{19}$F NMR spectrum of **3p** (376.5 MHz, $CDCl_3$)

3 Aug 2022

| | | | | | | | |
|---|---|---|---|---|---|---|---|
| ***Acquisition Time (sec)*** | 0.6783 | ***Comment*** | Imported from UXNMR. | | | ***Date*** | 20 Jan 2022 12:31:36 |
| ***File Name*** | C:\DOCS\OUTPUT_301\2022\01. 磤囵黒BM-2411-1.C_002001r | | | | | ***Frequency (MHz)*** | 100.61 |
| ***Nucleus*** | 13C | ***Number of Transients*** | 106 | ***Original Points Count*** | 16384 | ***Points Count*** | 131072 |
| ***Pulse Sequence*** | zgpg30 | ***Solvent*** | CHLOROFORM-D | | | ***Sweep Width (Hz)*** | 24154.59 |
| ***Temperature (degree C)*** | 27.000 | | | | | | |

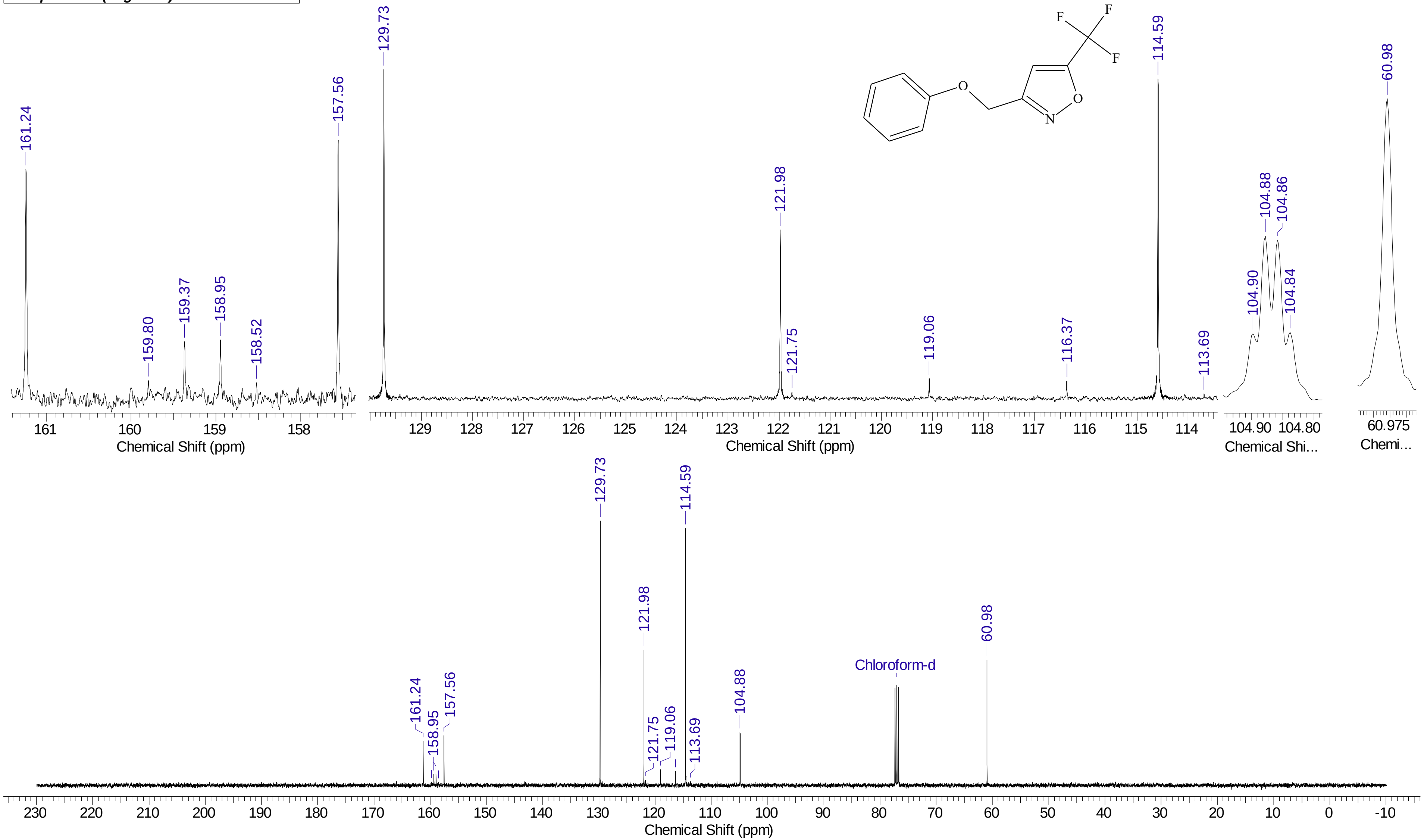

## $^{13}$C NMR spectrum of **3p** (100.6 MHz, $CDCl_3$)

20 May 2022

| | | | | | | | |
|---|---|---|---|---|---|---|---|
| ***Acquisition Time (sec)*** | 4.0894 | ***Comment*** | Imported from UXNMR. | | | ***Date*** | 08 Feb 2022 22:22:36 |
| ***File Name*** | C:\DOCS\BM\咛 峪 盐 雅 娜 \2022\BM-2447-3\BM-2447-3_001001r | | | | | ***Frequency (MHz)*** | 400.13 |
| ***Nucleus*** | 1H | ***Number of Transients*** | 8 | ***Original Points Count*** | 32768 | ***Points Count*** | 131072 |
| ***Pulse Sequence*** | zg30 | ***Solvent*** | CHLOROFORM-D | | | ***Sweep Width (Hz)*** | 8012.82 |
| ***Temperature (degree C)*** | 27.000 | | | | | | |

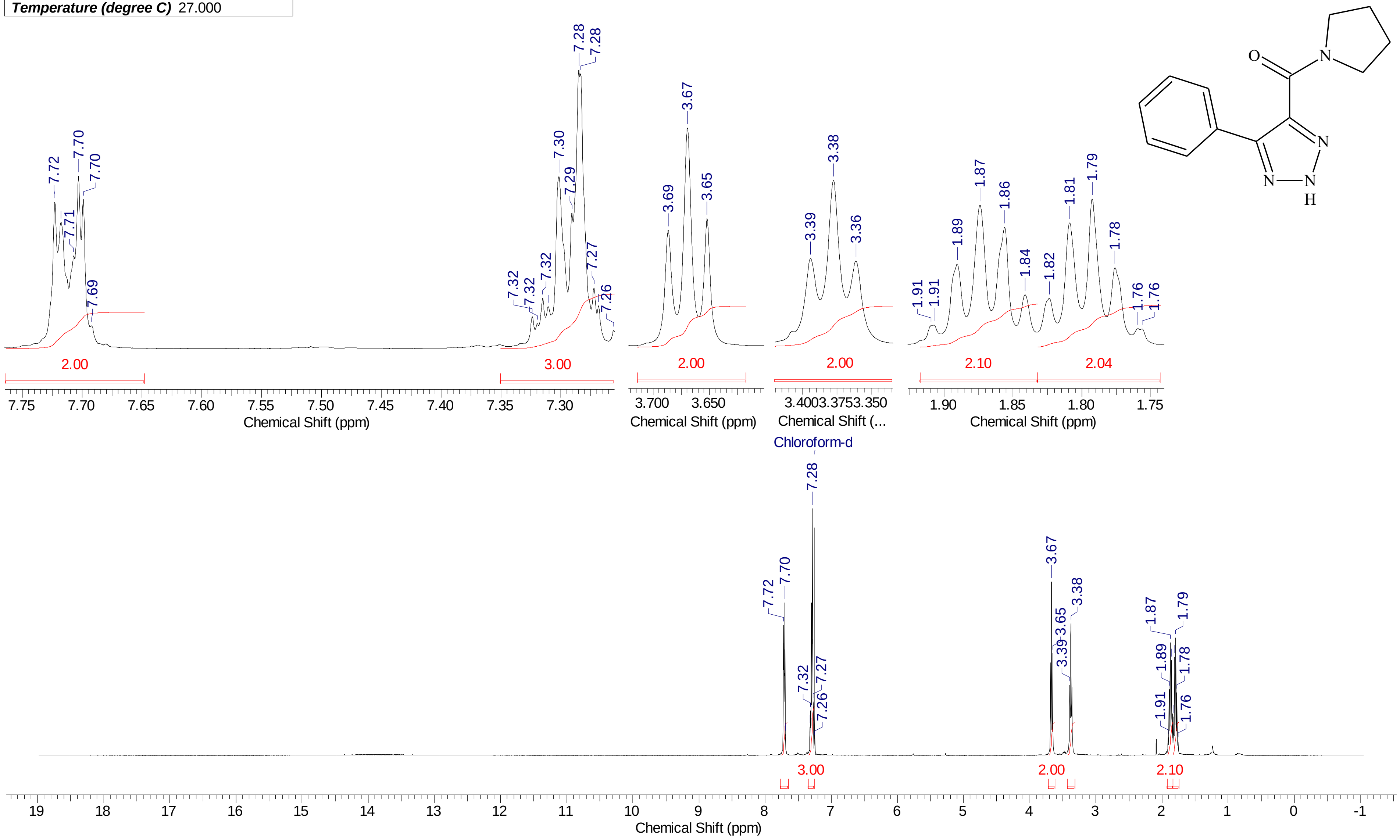

# $^{1}$H NMR spectrum of **4** (400.1 MHz, $CDCl_3$)

20 May 2022

| | | | | | | | |
|---|---|---|---|---|---|---|---|
| ***Acquisition Time (sec)*** | 0.6783 | ***Comment*** | Imported from UXNMR. | | | ***Date*** | 08 Feb 2022 22:50:58 |
| ***File Name*** | C:\DOCS\BM\咛 峪盐 雅 娜 \2022\BM-2447-3\BM-2447-3_002001r | | | | | ***Frequency (MHz)*** | 100.61 |
| ***Nucleus*** | 13C | ***Number of Transients*** | 648 | ***Original Points Count*** | 16384 | ***Points Count*** | 131072 |
| ***Pulse Sequence*** | zgpg30 | ***Solvent*** | CHLOROFORM-D | | | ***Sweep Width (Hz)*** | 24154.59 |
| ***Temperature (degree C)*** | 27.000 | | | | | | |

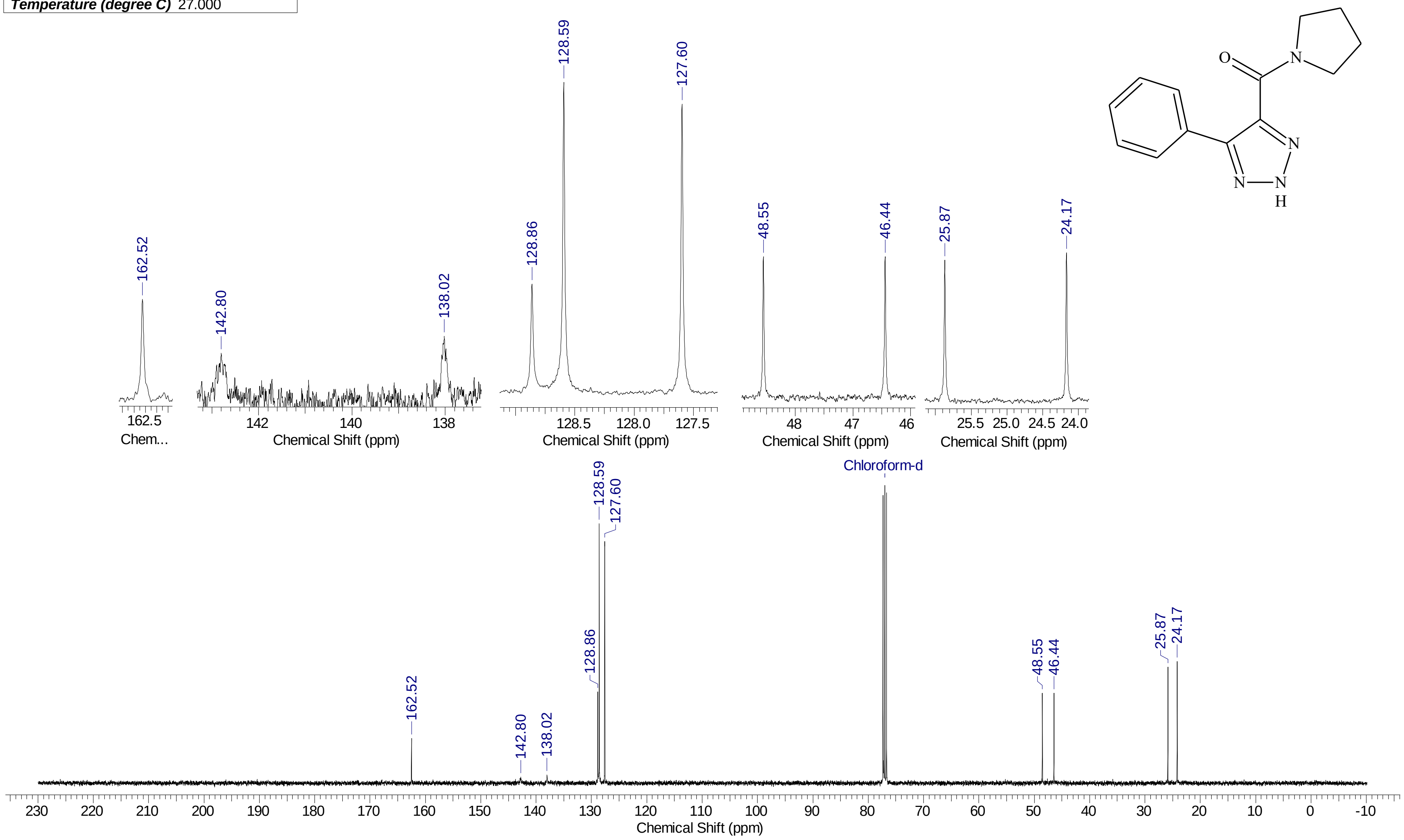

# $^{13}$C NMR spectrum of **4** (100.6 MHz, $CDCl_3$)

20 May 2022

| | | | | | | | |
|---|---|---|---|---|---|---|---|
| ***Acquisition Time (sec)*** | 4.0894 | ***Comment*** | Imported from UXNMR. | | | ***Date*** | 09 Mar 2022 22:11:46 |
| ***File Name*** | C:\DOCS\BM\咛 峈盐 雅 娜 \2022\BM-2449-2p\BM-2449-2p_001001r | | | | | ***Frequency (MHz)*** | 400.13 |
| ***Nucleus*** | 1H | ***Number of Transients*** | 8 | ***Original Points Count*** | 32768 | ***Points Count*** | 131072 |
| ***Pulse Sequence*** | zg30 | ***Solvent*** | CHLOROFORM-D | | | ***Sweep Width (Hz)*** | 8012.82 |
| ***Temperature (degree C)*** | 27.000 | | | | | | |

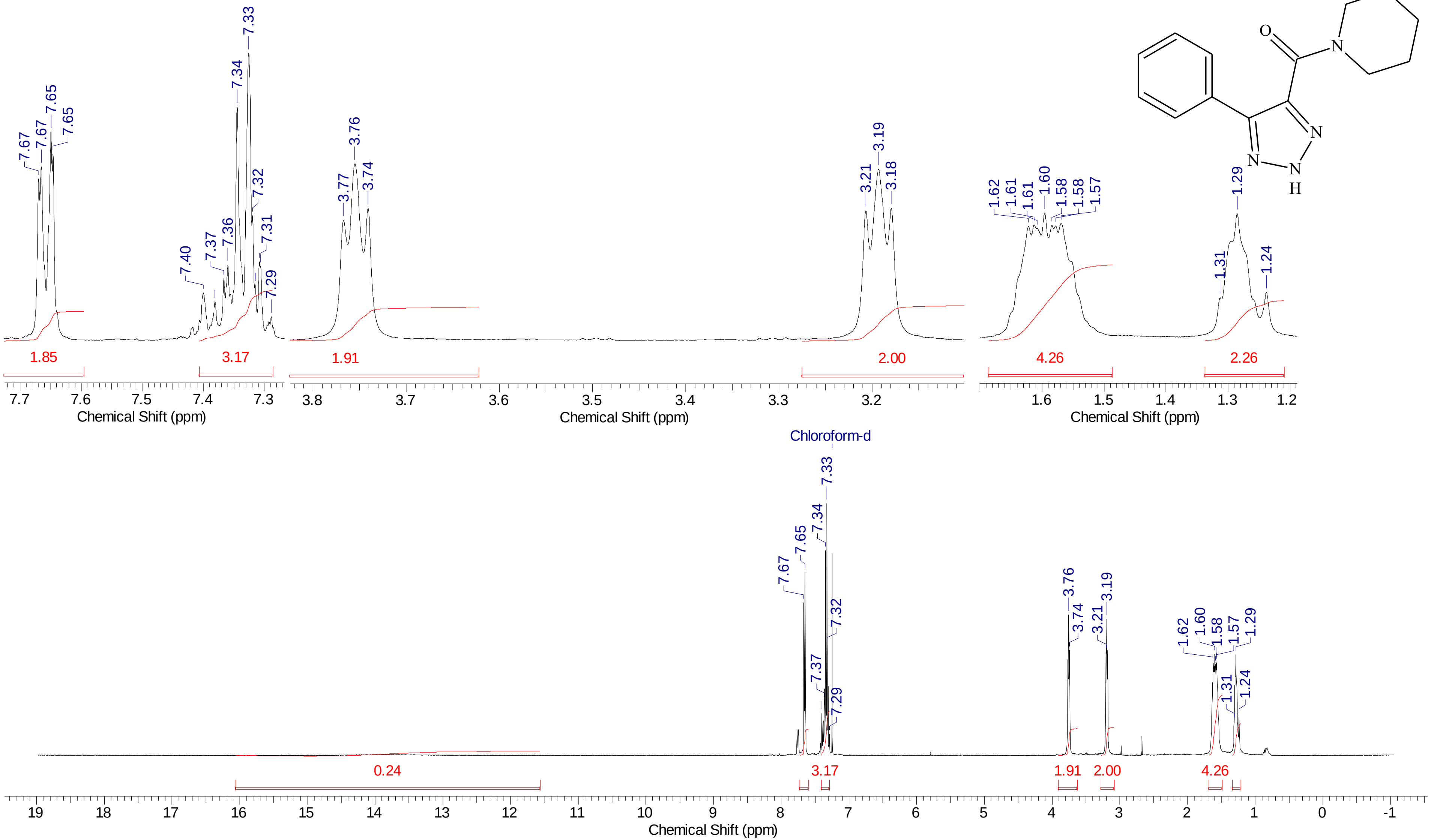

# $^{1}$H NMR spectrum of **5** (400.1 MHz, $CDCl_3$)

20 May 2022

| | | | | | | | |
|---|---|---|---|---|---|---|---|
| ***Acquisition Time (sec)*** | 0.6783 | ***Comment*** | Imported from UXNMR. | | | ***Date*** | 10 Mar 2022 12:24:32 |
| ***File Name*** | C:\DOCS\OUTPUT_301\2022\03.爨 痱\BM-2449-2p.C_002001r | | | | | ***Frequency (MHz)*** | 100.61 |
| ***Nucleus*** | 13C | ***Number of Transients*** | 244 | ***Original Points Count*** | 16384 | ***Points Count*** | 131072 |
| ***Pulse Sequence*** | zgpg30 | ***Solvent*** | ACETONITRILE-D3 | | | ***Sweep Width (Hz)*** | 24154.59 |
| ***Temperature (degree C)*** | 27.000 | | | | | | |

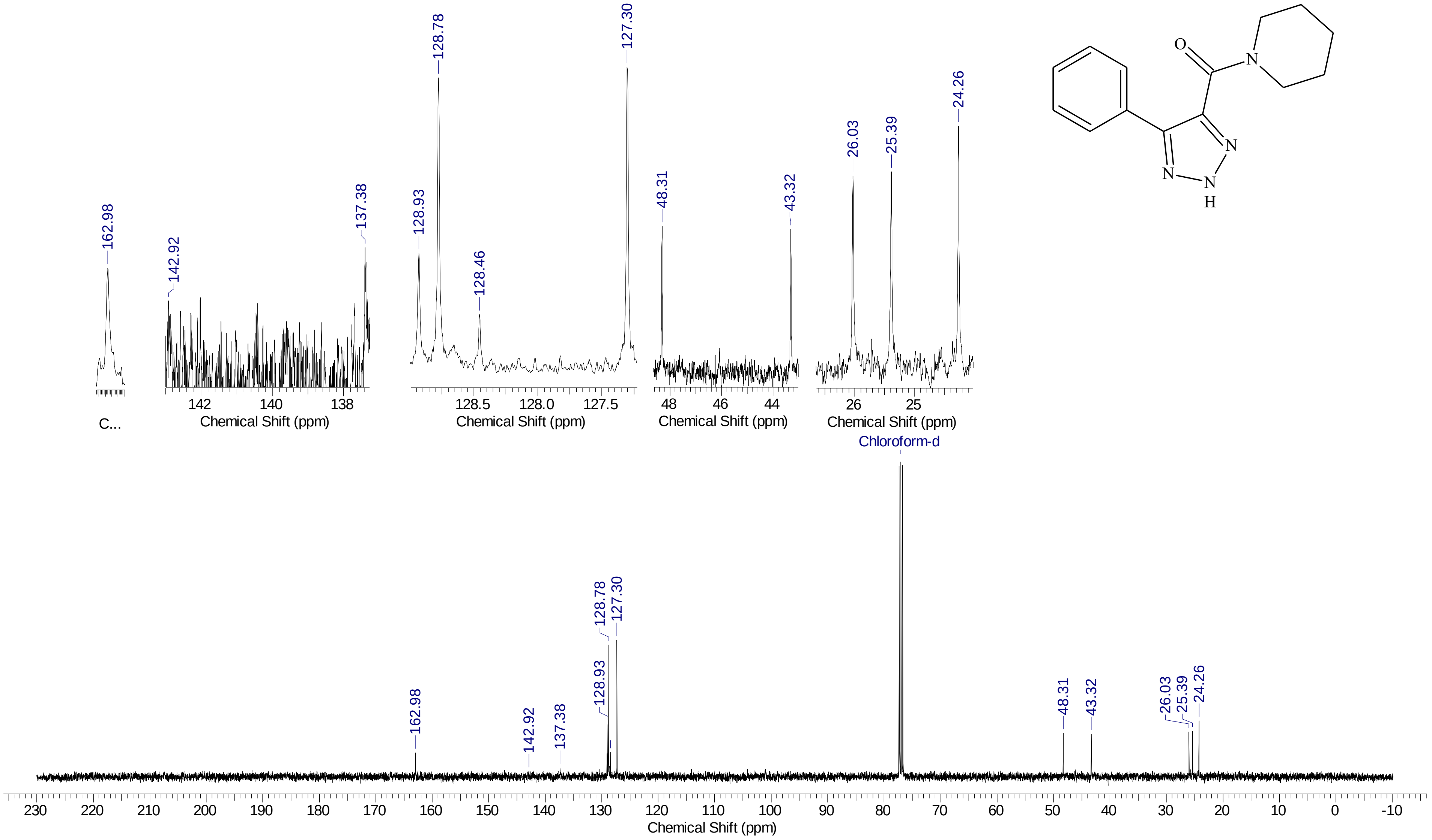

# $^{13}$C NMR spectrum of **5** (100.6 MHz, $CDCl_3$)

20 May 2022

| | | | | | |
|---|---|---|---|---|---|
| ***Acquisition Time (sec)*** | 4.0894 | ***Comment*** | Imported from UXNMR. | ***Date*** | 12 Mar 2022 23:41:18 |
| ***File Name*** | C:\DOCS\BM\咛 峈盐 雅 娜 \2022\BM-2450\BM-2450_001001r | | | ***Frequency (MHz)*** | 400.13 |
| ***Nucleus*** | 1H | ***Number of Transients*** | 8 ***Original Points Count*** 32768 | ***Points Count*** | 131072 |
| ***Pulse Sequence*** | zg30 | ***Solvent*** | CHLOROFORM-D | ***Sweep Width (Hz)*** | 8012.82 |
| ***Temperature (degree C)*** | 27.000 | | | | |

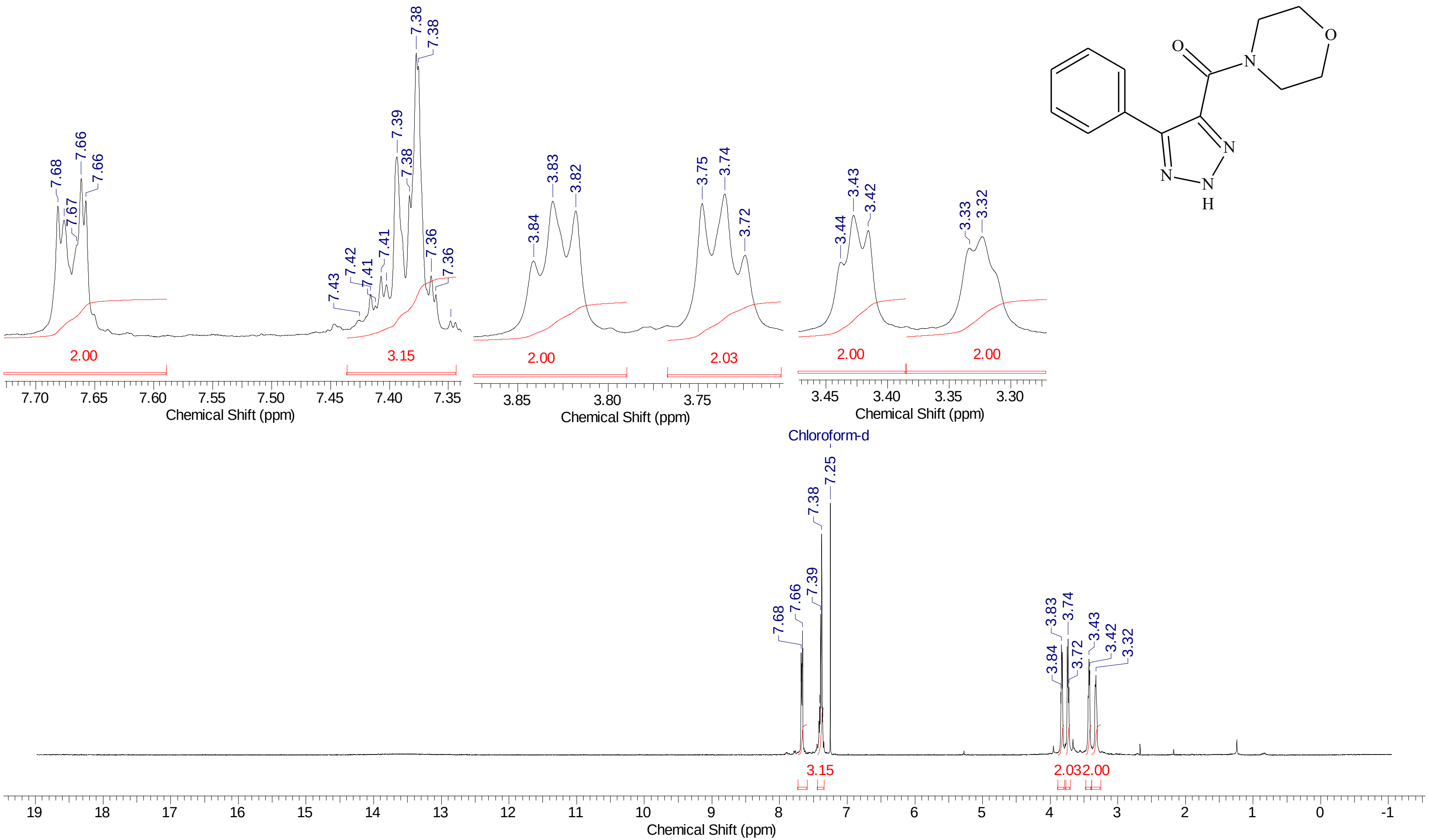

# $^{1}$H NMR spectrum of **6** (400.1 MHz, $CDCl_3$)

20 May 2022

| | | | | | | | |
|---|---|---|---|---|---|---|---|
| ***Acquisition Time (sec)*** | 0.6783 | ***Comment*** | Imported from UXNMR. | | | ***Date*** | 14 Mar 2022 15:38:42 |
| ***File Name*** | C:\DOCS\OUTPUT_301\2022\03.釁 痱\BM-2450-2.C_002001r | | | | | ***Frequency (MHz)*** | 100.61 |
| ***Nucleus*** | 13C | ***Number of Transients*** | 201 | ***Original Points Count*** | 16384 | ***Points Count*** | 131072 |
| ***Pulse Sequence*** | zgpg30 | ***Solvent*** | DMSO-D6 | ***Sweep Width (Hz)*** | 24154.59 | ***Temperature (degree C)*** | 27.000 |

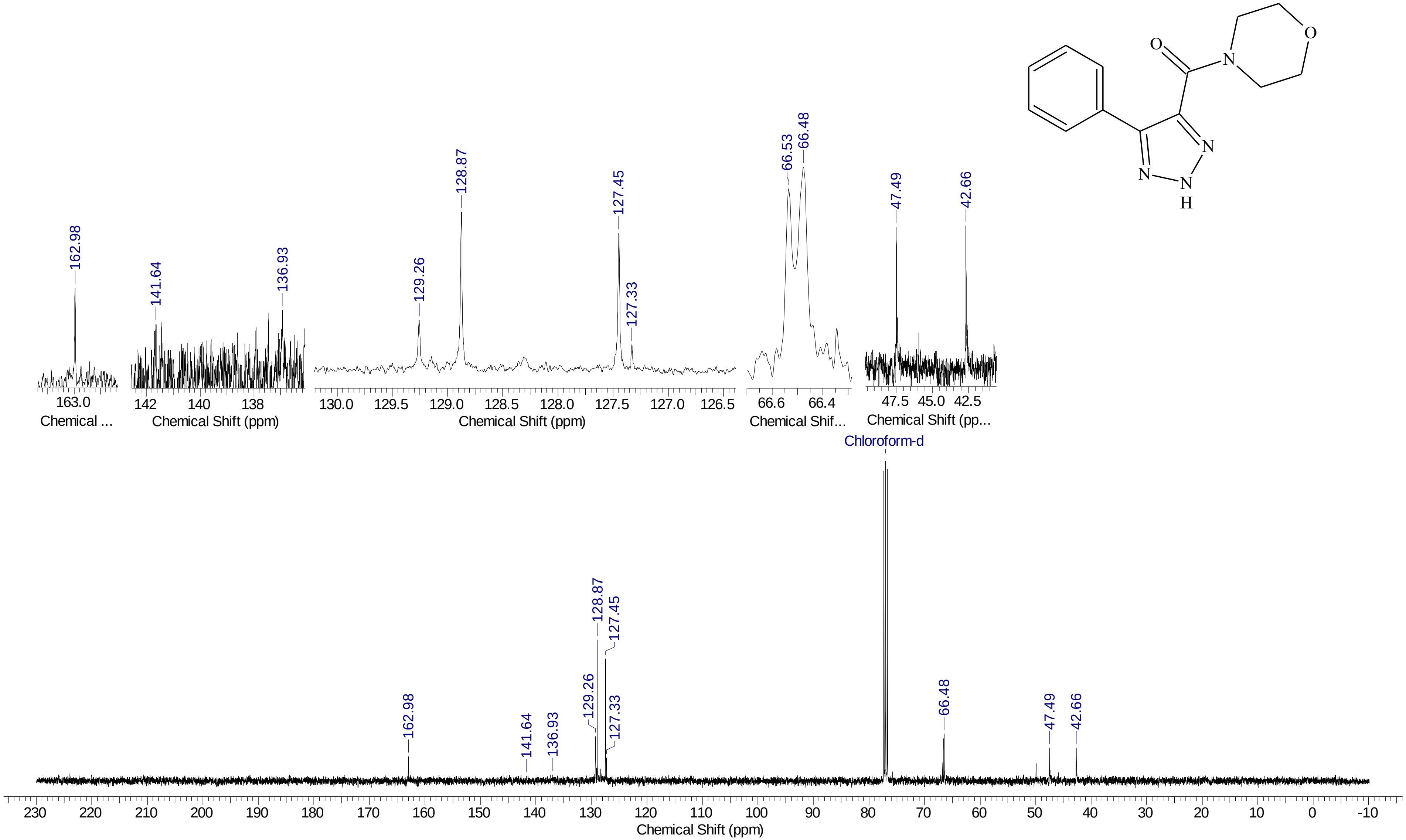

$^{13}$C NMR spectrum of **6** (100.6 MHz, $CDCl_3$)

18 May 2022

| | | | | | | | |
|---|---|---|---|---|---|---|---|
| ***Acquisition Time (sec)*** | 4.0894 | ***Comment*** | Imported from UXNMR. | | | ***Date*** | 29 Mar 2022 14:45:06 |
| ***File Name*** | C:\DOCS\OUTPUT_301\2022\03.爨 痱\SZA-BM-2448-7.H.H_001001r | | | | | ***Frequency (MHz)*** | 400.13 |
| ***Nucleus*** | 1H | ***Number of Transients*** | 4 | ***Original Points Count*** | 32768 | ***Points Count*** | 131072 |
| ***Pulse Sequence*** | zg30 | ***Solvent*** | CHLOROFORM-D | | | ***Sweep Width (Hz)*** | 8012.82 |
| ***Temperature (degree C)*** | 27.000 | | | | | | |

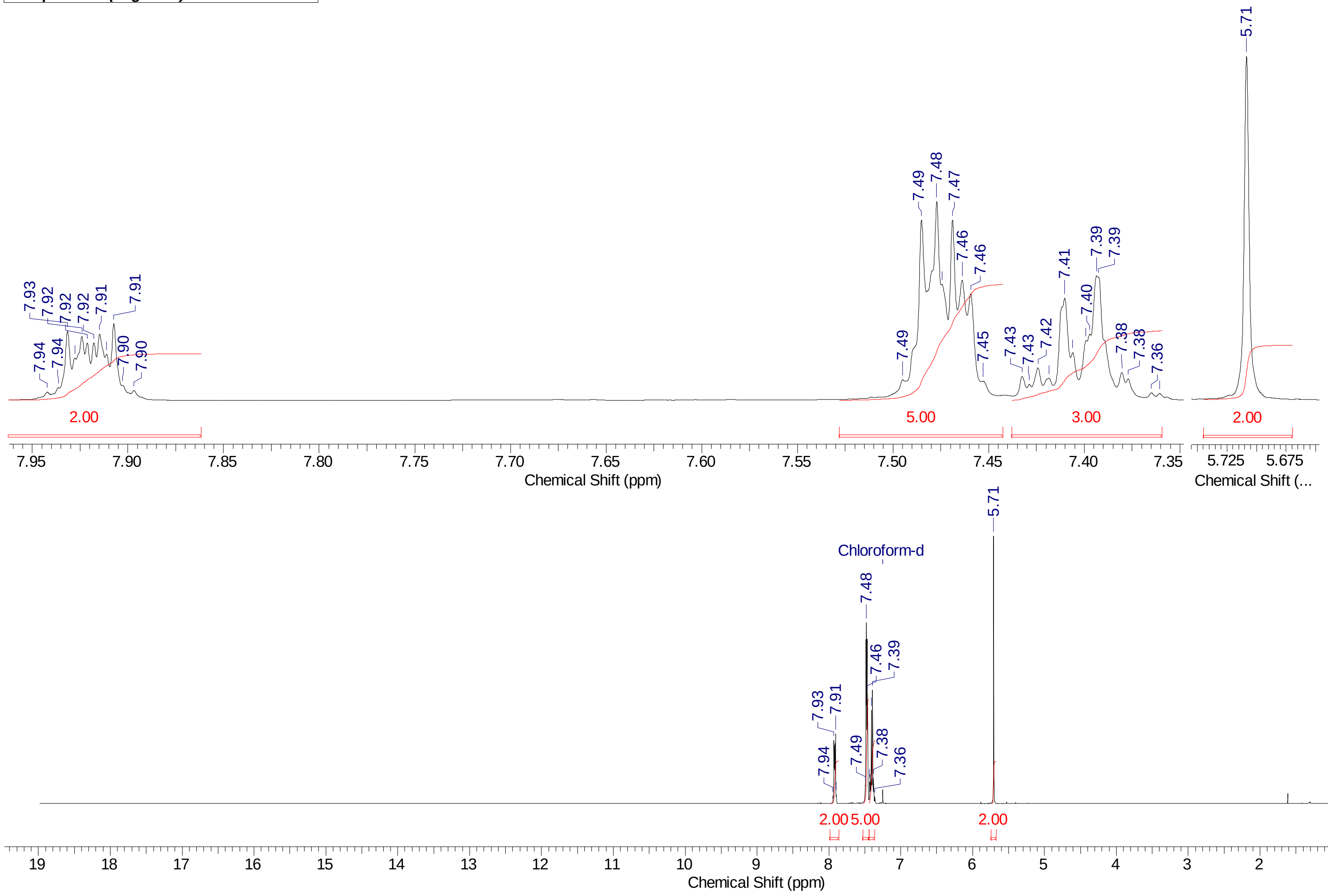


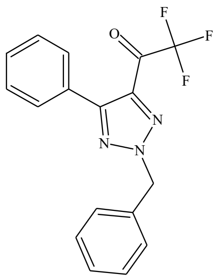

# $^{1}$H NMR spectrum of **7** (400.1 MHz, $CDCl_3$)

18 May 2022

| | | | | | | | |
|---|---|---|---|---|---|---|---|
| ***Acquisition Time (sec)*** | 1.7433 | ***Comment*** | Imported from UXNMR. | | | ***Date*** | 29 Mar 2022 14:56:42 |
| ***File Name*** | C:\DOCS\OUTPUT_301\2022\03.爨 痱\SZA-BM-2448-7.F_005001r | | | | | ***Frequency (MHz)*** | 376.50 |
| ***Nucleus*** | 19F | ***Number of Transients*** | 4 | ***Original Points Count*** | 131072 | ***Points Count*** | 262144 |
| ***Pulse Sequence*** | zgflqn | ***Solvent*** | CHLOROFORM-D | | | ***Sweep Width (Hz)*** | 75187.97 |
| ***Temperature (degree C)*** | 27.000 | | | | | | |

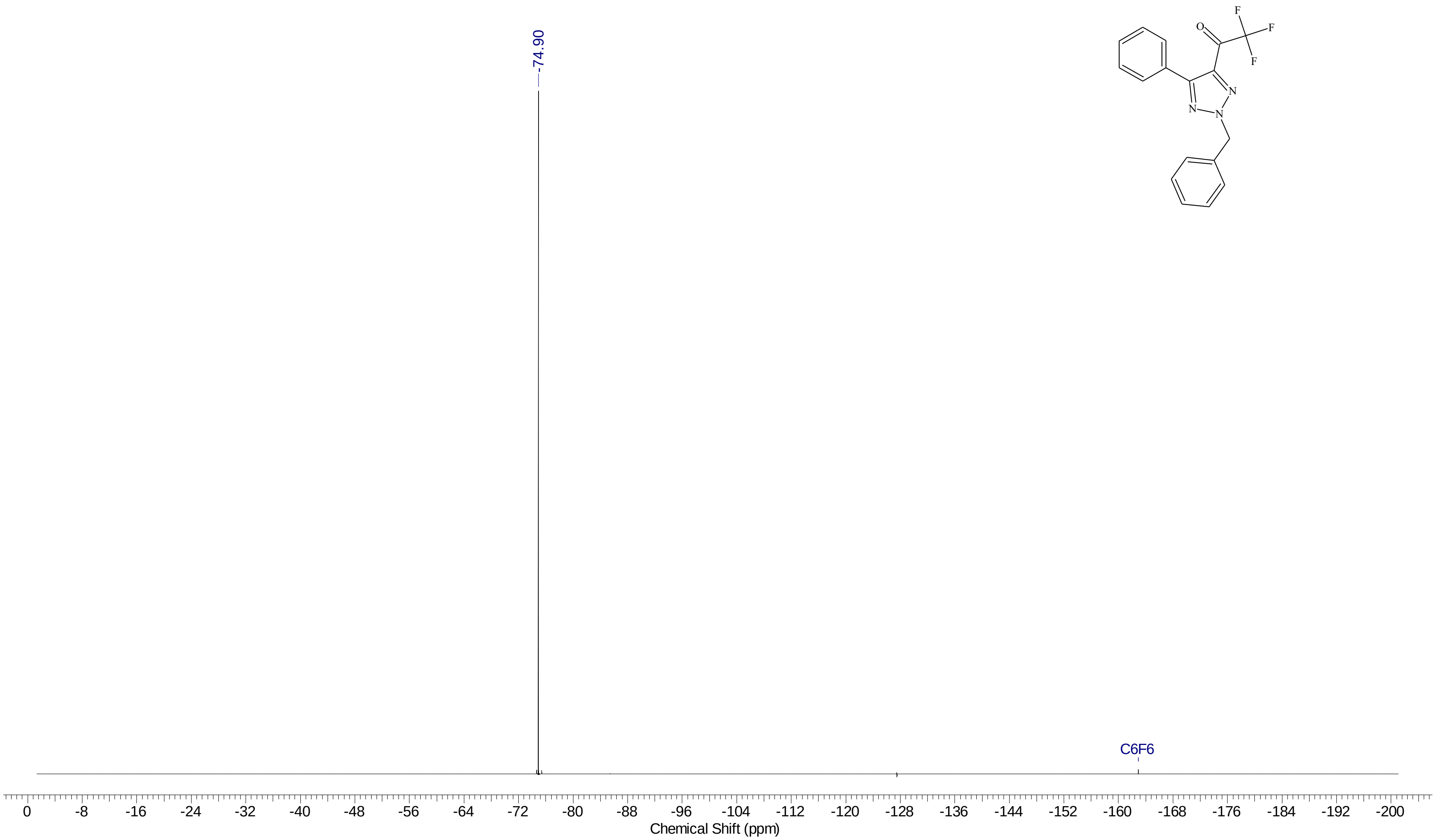

# $^{19}F$ NMR spectrum of **7** (376.5 MHz, $CDCl_3$)

18 May 2022

| | | | | | | | |
|---|---|---|---|---|---|---|---|
| ***Acquisition Time (sec)*** | 0.6783 | ***Comment*** | Imported from UXNMR. | | | ***Date*** | 31 Mar 2022 12:08:00 |
| ***File Name*** | C:\DOCS\OUTPUT_301\2022\03.爨 痱\SZA-BM-2448-7.C_002001r | | | | | ***Frequency (MHz)*** | 100.61 |
| ***Nucleus*** | 13C | ***Number of Transients*** | 73 | ***Original Points Count*** | 16384 | ***Points Count*** | 131072 |
| ***Pulse Sequence*** | zgpg30 | ***Solvent*** | DMSO-D6 | ***Sweep Width (Hz)*** | 24154.59 | ***Temperature (degree C)*** | 27.000 |

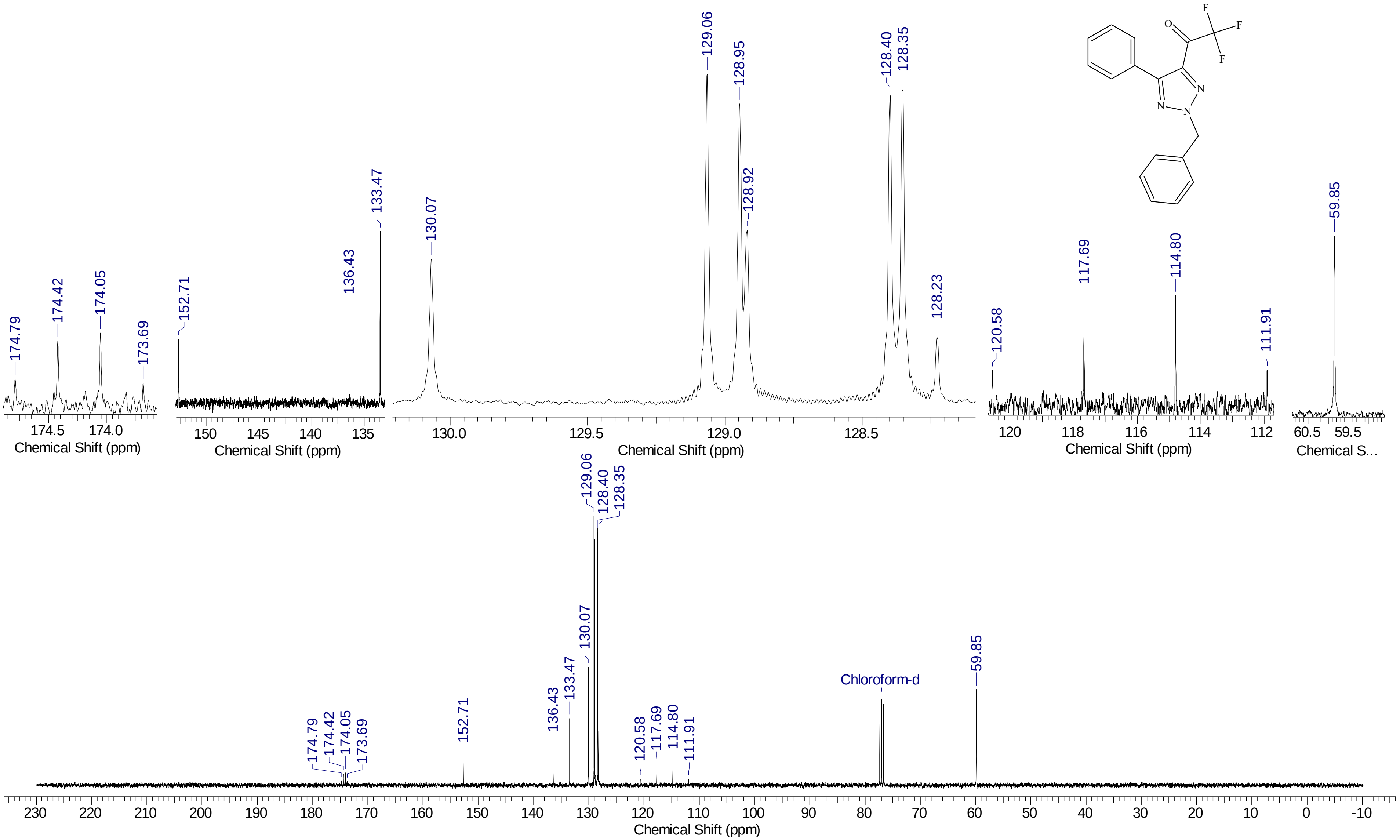

## $^{13}$C NMR spectrum of **7** (100.6 MHz, $CDCl_3$)

18 May 2022

| | | | | | | | |
|---|---|---|---|---|---|---|---|
| ***Acquisition Time (sec)*** | 4.0894 | ***Comment*** | Imported from UXNMR. | | | ***Date*** | 30 Mar 2022 17:43:12 |
| ***File Name*** | C:\DOCS\OUTPUT_301\2022\03.爨 痱\SZA-BM-2448-25.H_001001r | | | | | ***Frequency (MHz)*** | 400.13 |
| ***Nucleus*** | 1H | ***Number of Transients*** | 4 | ***Original Points Count*** | 32768 | ***Points Count*** | 131072 |
| ***Pulse Sequence*** | zg30 | ***Solvent*** | CHLOROFORM-D | | | ***Sweep Width (Hz)*** | 8012.82 |
| ***Temperature (degree C)*** | 27.000 | | | | | | |

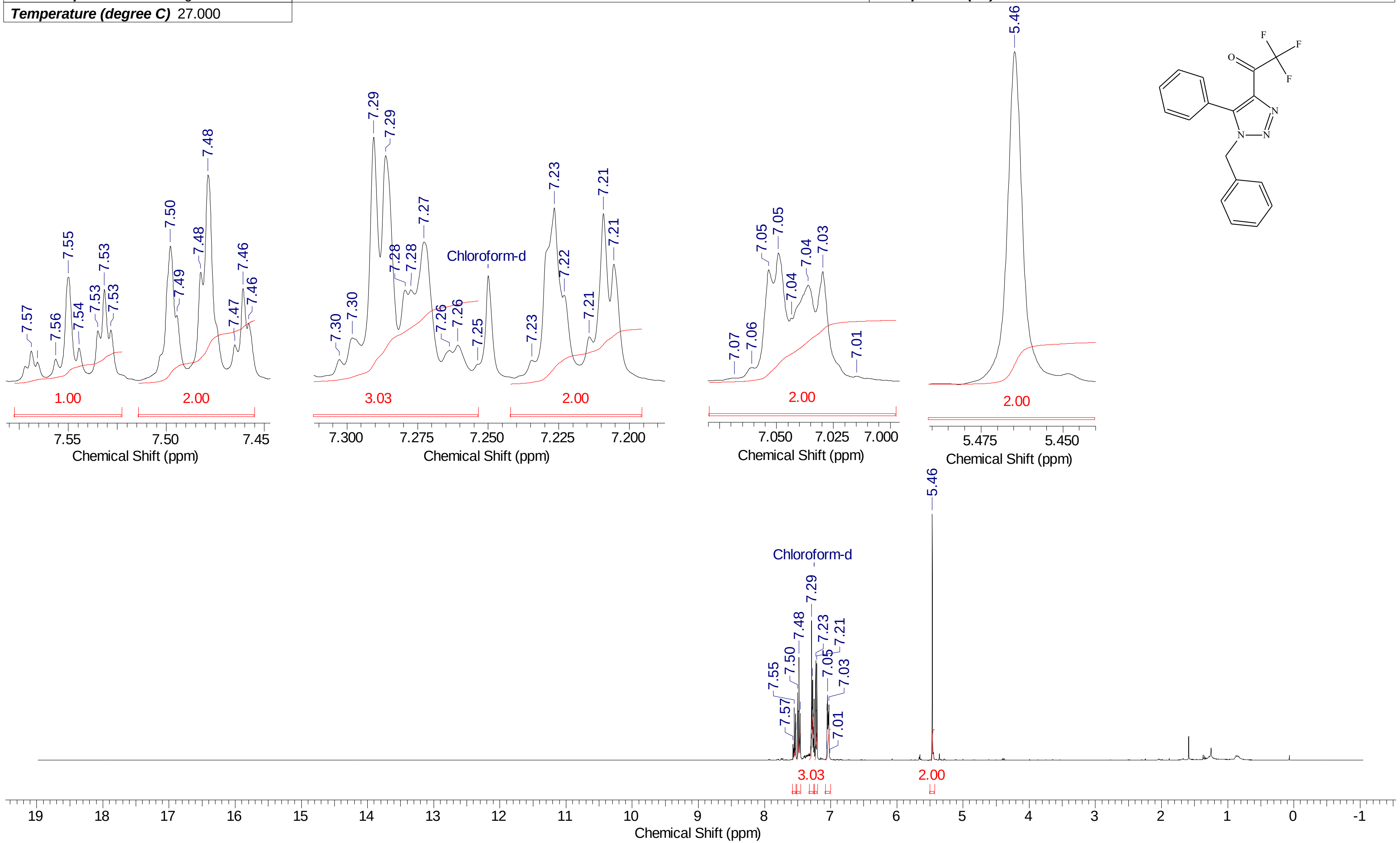

# $^{1}H$ NMR spectrum of **8** (400.1 MHz, $CDCl_3$)

18 May 2022

| | | | | | | | |
|---|---|---|---|---|---|---|---|
| ***Acquisition Time (sec)*** | 1.7433 | ***Comment*** | Imported from UXNMR. | | | ***Date*** | 29 Mar 2022 15:15:50 |
| ***File Name*** | C:\DOCS\OUTPUT_301\2022\03.爨 痱\SZA-BM-2448-21.F_005001r | | | | | ***Frequency (MHz)*** | 376.50 |
| ***Nucleus*** | 19F | ***Number of Transients*** | 4 | ***Original Points Count*** | 131072 | ***Points Count*** | 262144 |
| ***Pulse Sequence*** | zgflqn | ***Solvent*** | CHLOROFORM-D | | | ***Sweep Width (Hz)*** | 75187.97 |
| ***Temperature (degree C)*** | 27.000 | | | | | | |

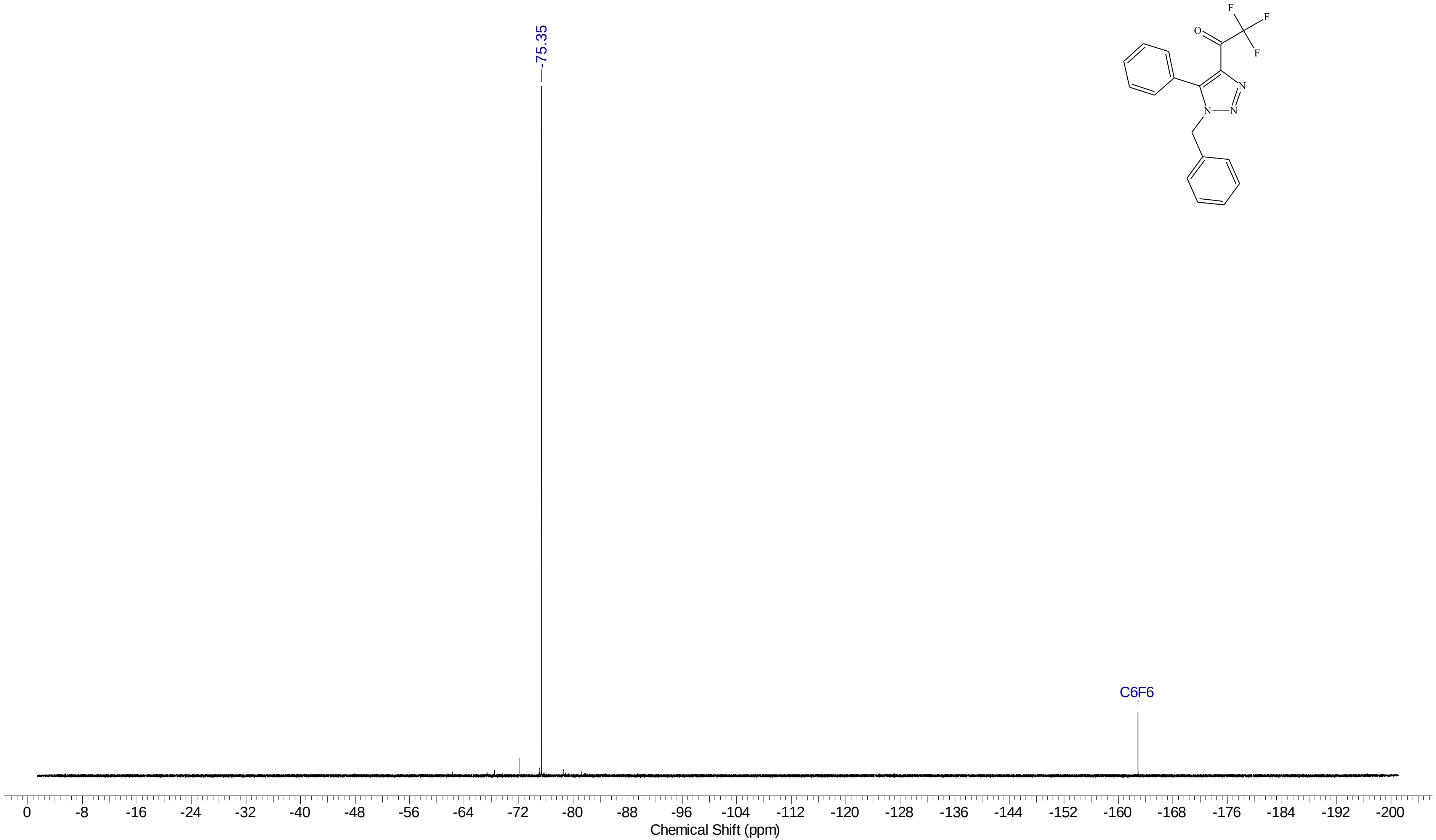

# $^{19}$F NMR spectrum of **8** (376.5 MHz, $CDCl_3$)

18 May 2022

| | | | | | | | |
|---|---|---|---|---|---|---|---|
| ***Acquisition Time (sec)*** | 0.6783 | ***Comment*** | Imported from UXNMR. | | | ***Date*** | 31 Mar 2022 15:24:40 |
| ***File Name*** | C:\DOCS\OUTPUT_301\2022\03.爨 痱\SZA-BM-2448-25.C_002001r | | | | | ***Frequency (MHz)*** | 100.61 |
| ***Nucleus*** | 13C | ***Number of Transients*** | 347 | ***Original Points Count*** | 16384 | ***Points Count*** | 131072 |
| ***Pulse Sequence*** | zgpg30 | ***Solvent*** | DMSO-D6 | ***Sweep Width (Hz)*** | 24154.59 | ***Temperature (degree C)*** | 27.000 |

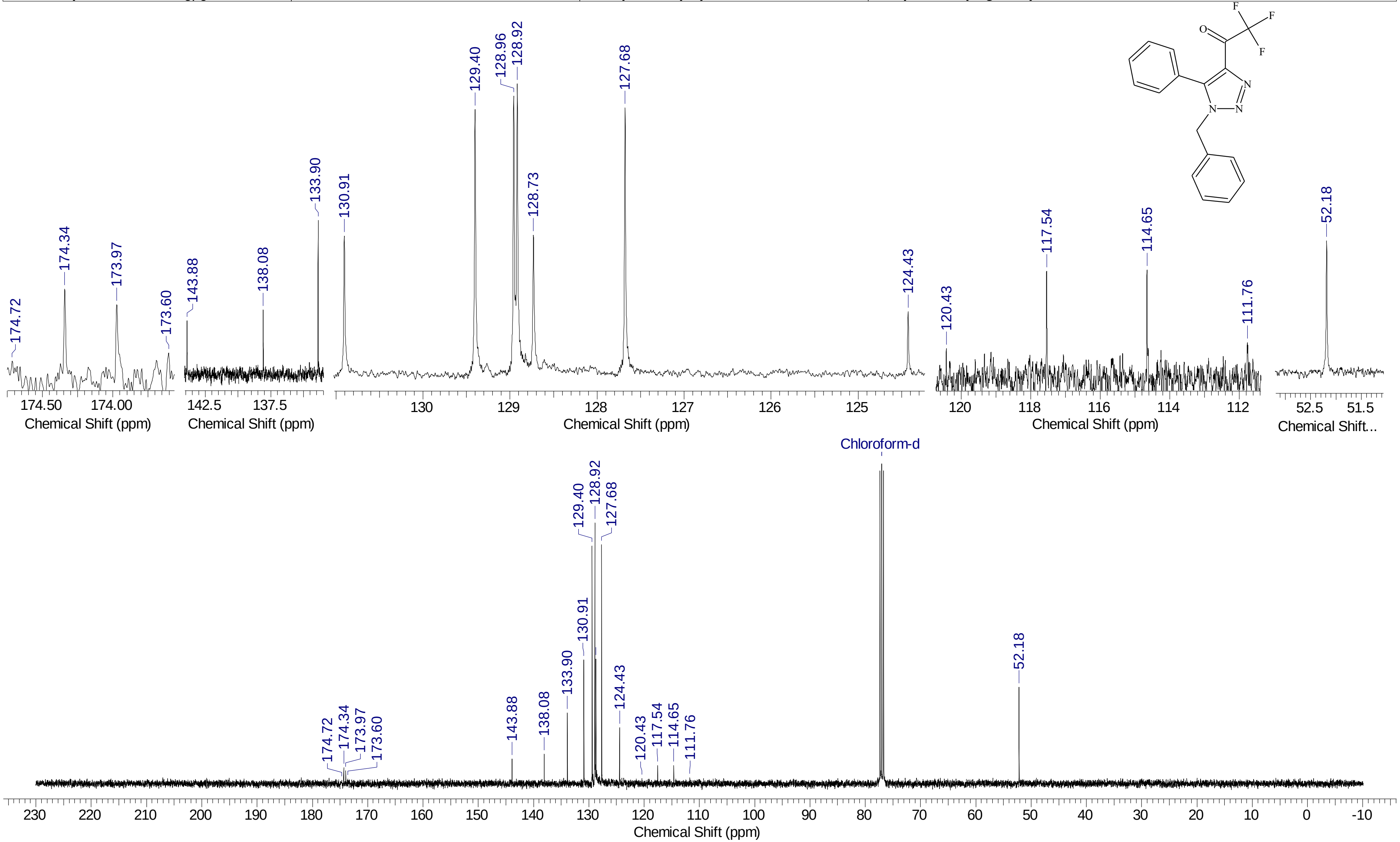

# $^{13}$C NMR spectrum of **8** (100.6 MHz, $CDCl_3$)

19 May 2022

| | | | | | | | |
|---|---|---|---|---|---|---|---|
| ***Acquisition Time (sec)*** | 4.0894 | ***Comment*** | Imported from UXNMR. | | | ***Date*** | 22 Mar 2022 15:43:50 |
| ***File Name*** | C:\DOCS\OUTPUT_301\2022\03.爨 痱\BM-2461-1p.H_001001r | | | | | ***Frequency (MHz)*** | 400.13 |
| ***Nucleus*** | 1H | ***Number of Transients*** | 4 | ***Original Points Count*** | 32768 | ***Points Count*** | 131072 |
| ***Pulse Sequence*** | zg30 | ***Solvent*** | CHLOROFORM-D | | | ***Sweep Width (Hz)*** | 8012.82 |
| ***Temperature (degree C)*** | 27.000 | | | | | | |

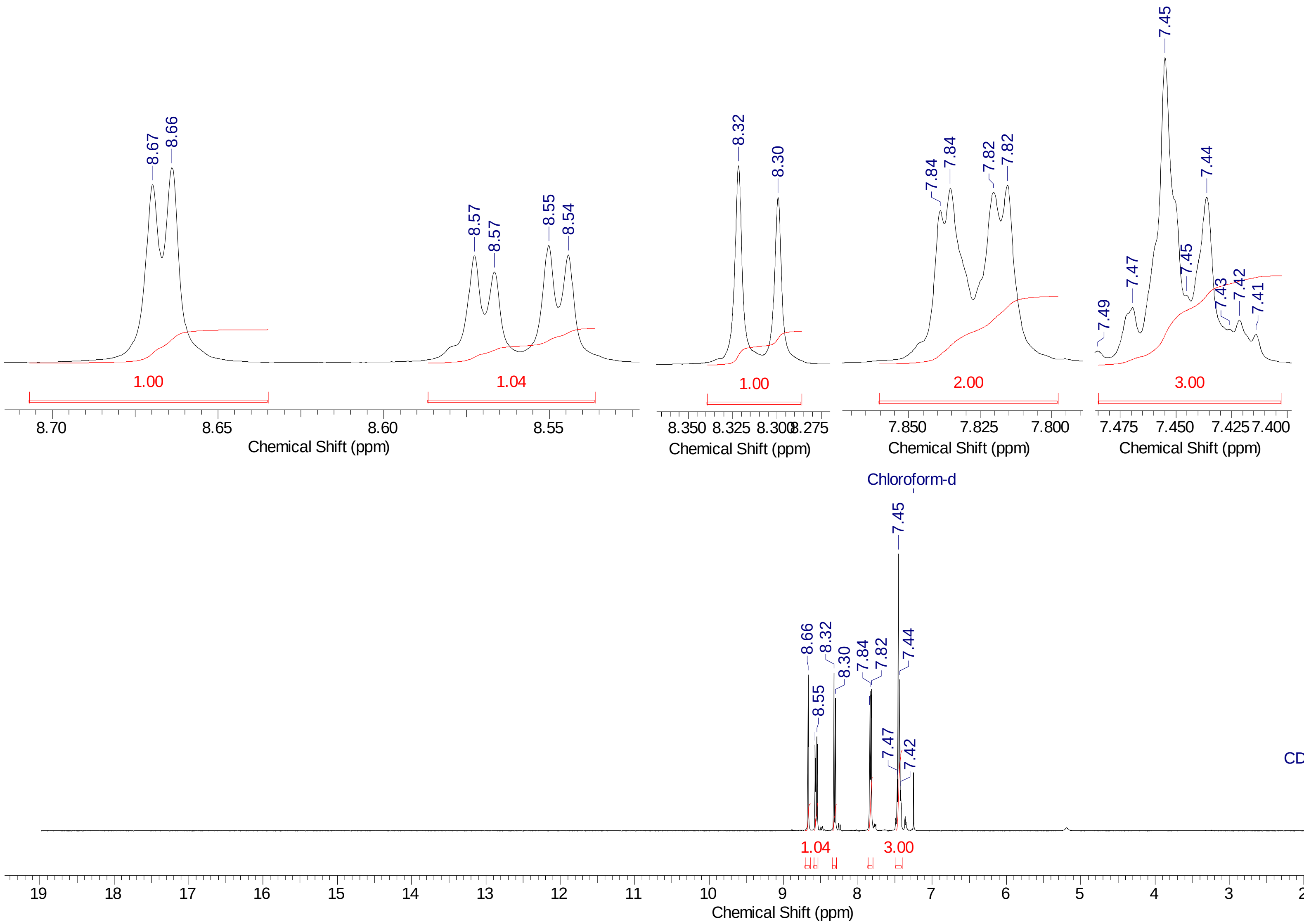


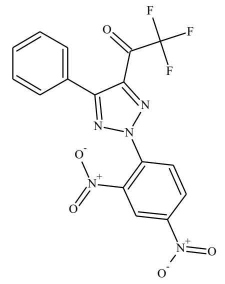

# $^{1}$H NMR spectrum of **9** (400.1 MHz, $CDCl_3$)

19 May 2022

| | | | | | | | |
|---|---|---|---|---|---|---|---|
| ***Acquisition Time (sec)*** | 1.7433 | ***Comment*** | Imported from UXNMR. | | | ***Date*** | 22 Mar 2022 15:28:42 |
| ***File Name*** | C:\DOCS\OUTPUT_301\2022\03.爨 痱\BM-2461-1p.F_005001r | | | | | ***Frequency (MHz)*** | 376.50 |
| ***Nucleus*** | 19F | ***Number of Transients*** | 4 | ***Original Points Count*** | 131072 | ***Points Count*** | 262144 |
| ***Pulse Sequence*** | zgflqn | ***Solvent*** | CHLOROFORM-D | | | ***Sweep Width (Hz)*** | 75187.97 |
| ***Temperature (degree C)*** | 27.000 | | | | | | |

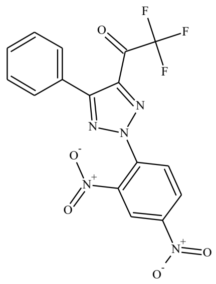

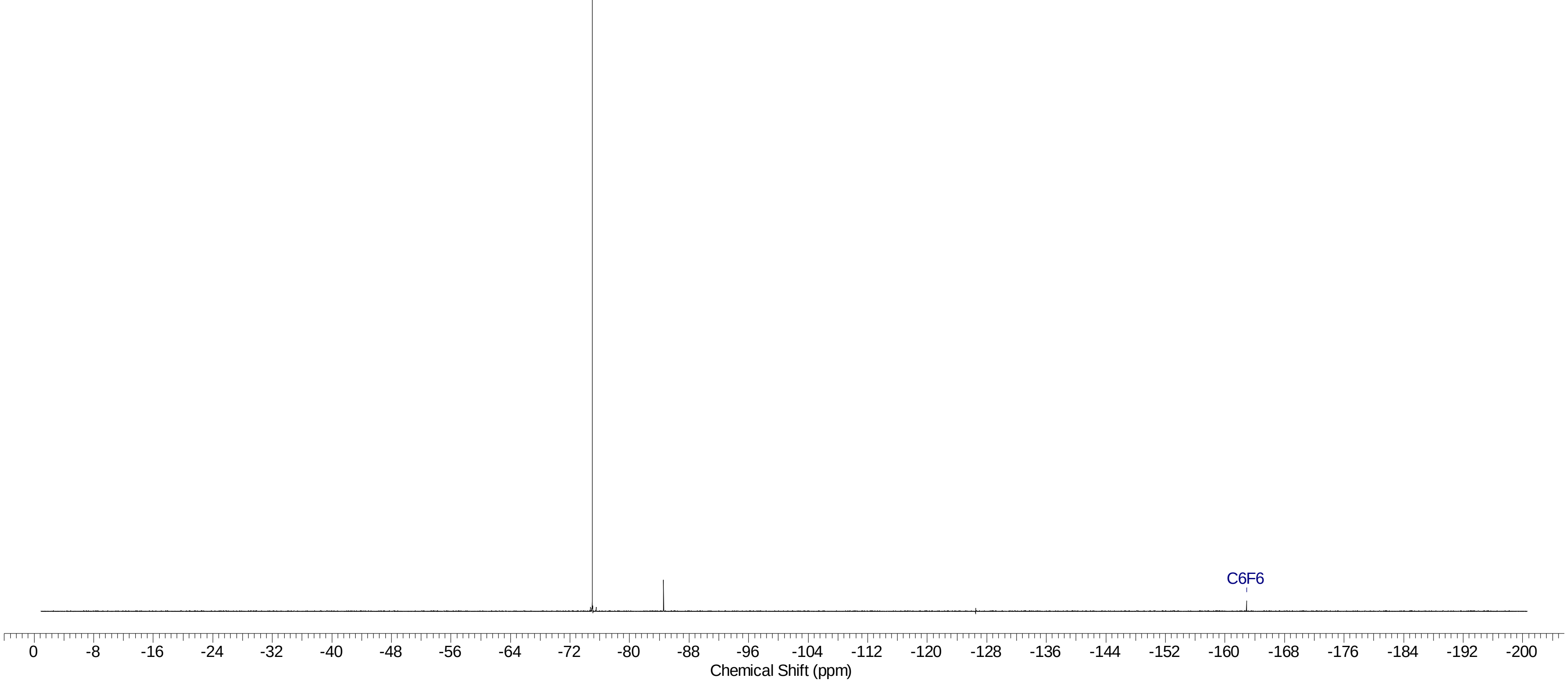

## $^{19}$F NMR spectrum of **9** (376.5 MHz, $CDCl_3$)

19 May 2022

| | | | | | | | |
|---|---|---|---|---|---|---|---|
| ***Acquisition Time (sec)*** | 0.6783 | ***Comment*** | Imported from UXNMR. | | | ***Date*** | 22 Mar 2022 15:54:02 |
| ***File Name*** | C:\DOCS\OUTPUT_301\2022\03.爨 痱\BM-2461-1p.C_002001r | | | | | ***Frequency (MHz)*** | 100.61 |
| ***Nucleus*** | 13C | ***Number of Transients*** | 266 | ***Original Points Count*** | 16384 | ***Points Count*** | 131072 |
| ***Pulse Sequence*** | zgpg30 | ***Solvent*** | DMSO-D6 | ***Sweep Width (Hz)*** | 24154.59 | ***Temperature (degree C)*** | 27.000 |

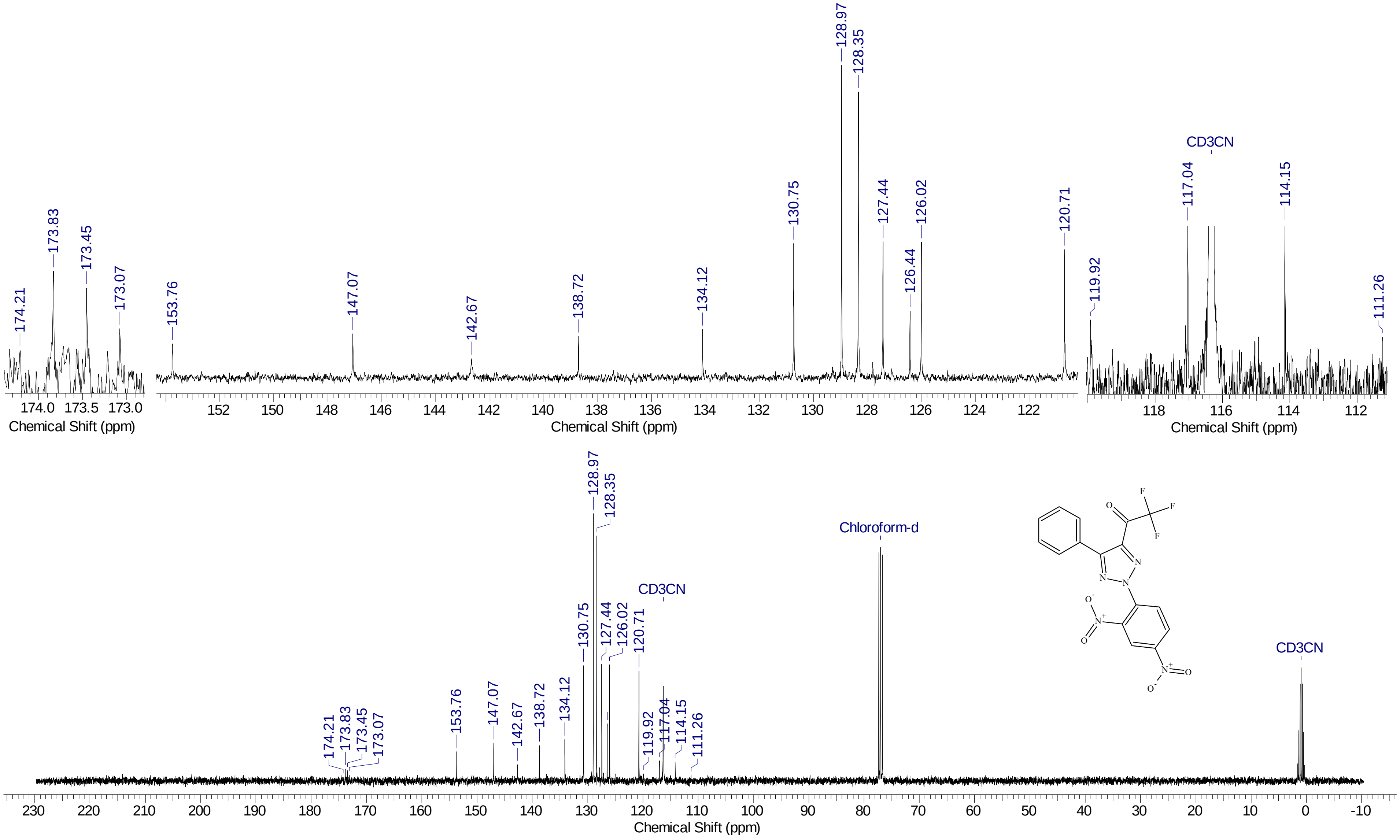

$^{13}$C NMR spectrum of **9** (100.6 MHz, $CDCl_3$)

19 May 2022

| | | | | | | | |
|---|---|---|---|---|---|---|---|
| ***Acquisition Time (sec)*** | 4.0894 | ***Comment*** | Imported from UXNMR. | | | ***Date*** | 30 Mar 2022 17:23:40 |
| ***File Name*** | C:\DOCS\OUTPUT_301\2022\03.爨 痱\SZA-BM-2470.H_001001r | | | | | ***Frequency (MHz)*** | 400.13 |
| ***Nucleus*** | 1H | ***Number of Transients*** | 4 | ***Original Points Count*** | 32768 | ***Points Count*** | 131072 |
| ***Pulse Sequence*** | zg30 | ***Solvent*** | CHLOROFORM-D | | | ***Sweep Width (Hz)*** | 8012.82 |
| ***Temperature (degree C)*** | 27.000 | | | | | | |

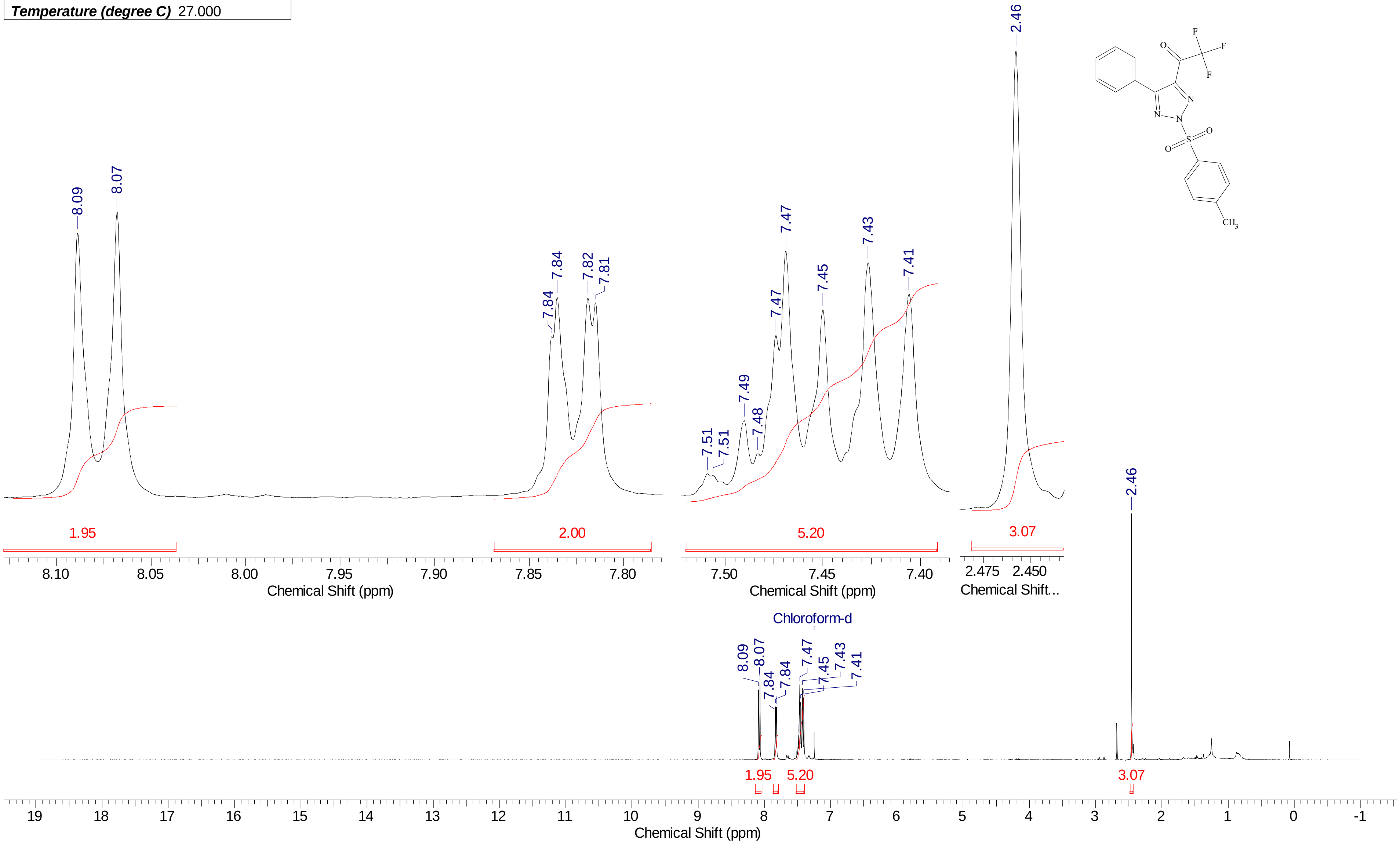

# $^{1}H$ NMR spectrum of **10** (400.1 MHz, $CDCl_3$)

19 May 2022

| | | | | | |
|---|---|---|---|---|---|
| ***Acquisition Time (sec)*** | 1.7433 | ***Comment*** | Imported from UXNMR. | ***Date*** | 30 Mar 2022 17:49:38 |
| ***File Name*** | C:\DOCS\OUTPUT_301\2022\03.爨 痱\SZA-BM-2470.F_005001r | | | ***Frequency (MHz)*** | 376.50 |
| ***Nucleus*** | 19F | ***Number of Transients*** | 16 ***Original Points Count*** 131072 | ***Points Count*** | 262144 |
| ***Pulse Sequence*** | zgflqn | ***Solvent*** | CHLOROFORM-D | ***Sweep Width (Hz)*** | 75187.97 |
| ***Temperature (degree C)*** | 27.000 | | | | |

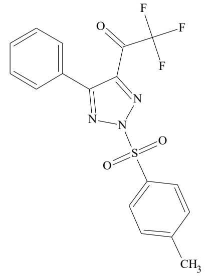


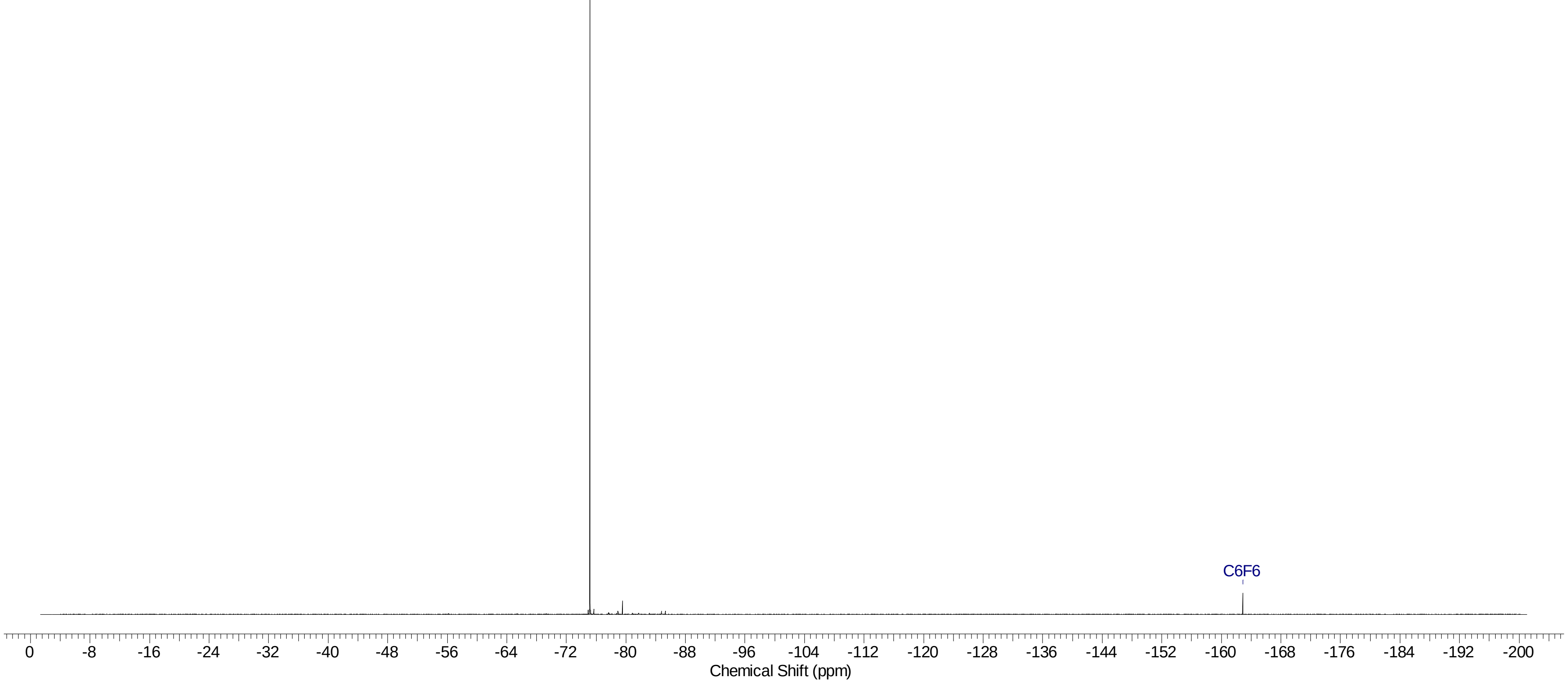

# $^{19}$F NMR spectrum of **10** (376.5 MHz, $CDCl_3$)

19 May 2022

| | | | | | | | |
|---|---|---|---|---|---|---|---|
| ***Acquisition Time (sec)*** | 0.6783 | ***Comment*** | Imported from UXNMR. | | | ***Date*** | 31 Mar 2022 12:54:26 |
| ***File Name*** | C:\DOCS\OUTPUT_301\2022\03.爨 痱\SZA-BM-2470.C_002001r | | | | | ***Frequency (MHz)*** | 100.61 |
| ***Nucleus*** | 13C | ***Number of Transients*** | 305 | ***Original Points Count*** | 16384 | ***Points Count*** | 131072 |
| ***Pulse Sequence*** | zgpg30 | ***Solvent*** | DMSO-D6 | ***Sweep Width (Hz)*** | 24154.59 | ***Temperature (degree C)*** | 27.000 |

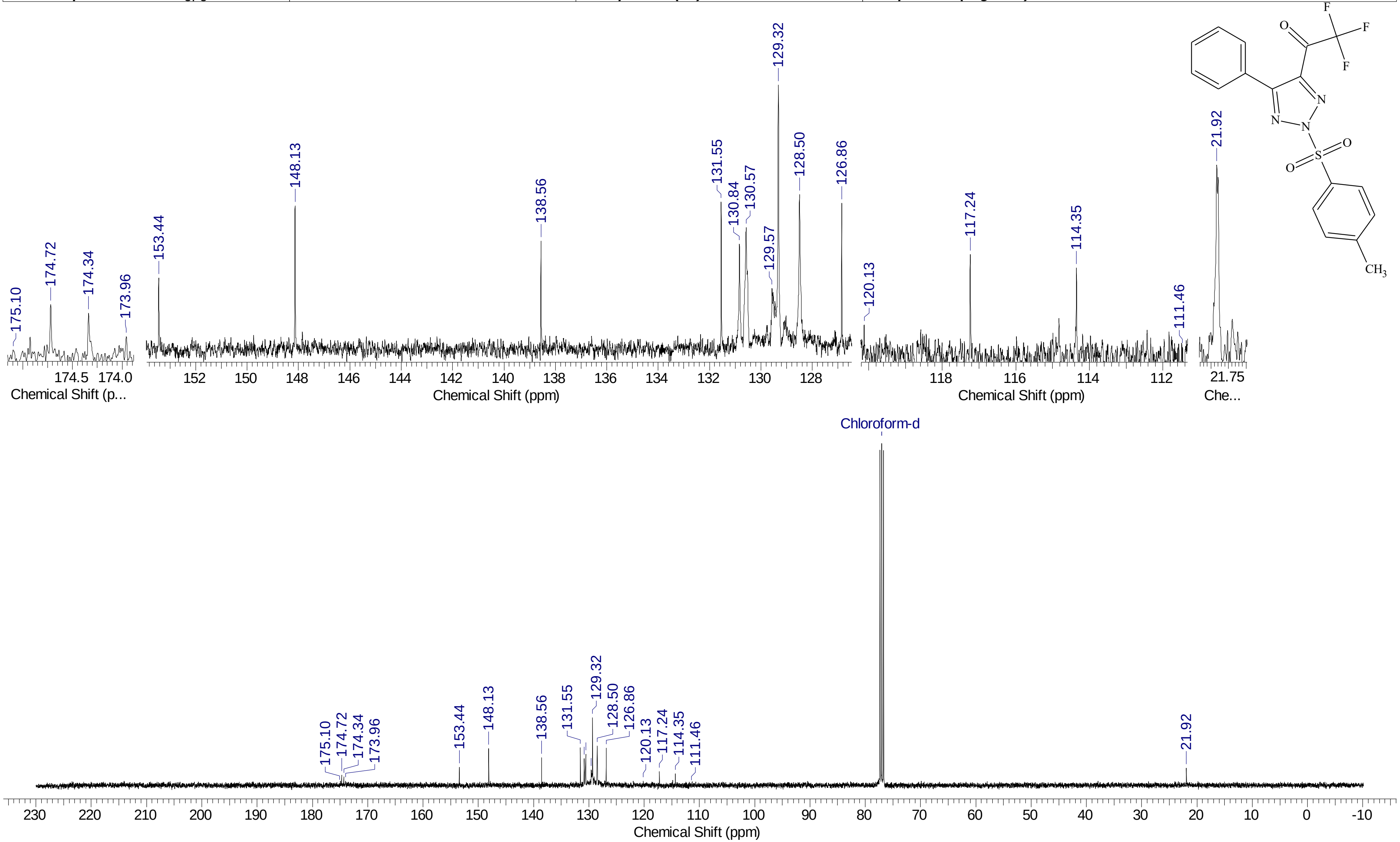

# $^{13}$C NMR spectrum of **10** (100.6 MHz, $CDCl_3$)

19 May 2022

| | | | | | |
|---|---|---|---|---|---|
| ***Acquisition Time (sec)*** | 4.0894 | ***Comment*** | Imported from UXNMR. | ***Date*** | 21 Apr 2022 12:39:06 |
| ***File Name*** | C:\DOCS\OUTPUT_301\2022\04.图痴朦\BM-2505-2.H_001001r | | | ***Frequency (MHz)*** | 400.13 |
| ***Nucleus*** | 1H | ***Number of Transients*** | 8 ***Original Points Count*** 32768 | ***Points Count*** | 131072 |
| ***Pulse Sequence*** | zg30 | ***Solvent*** | CHLOROFORM-D | ***Sweep Width (Hz)*** | 8012.82 |
| ***Temperature (degree C)*** | 27.000 | | | | |

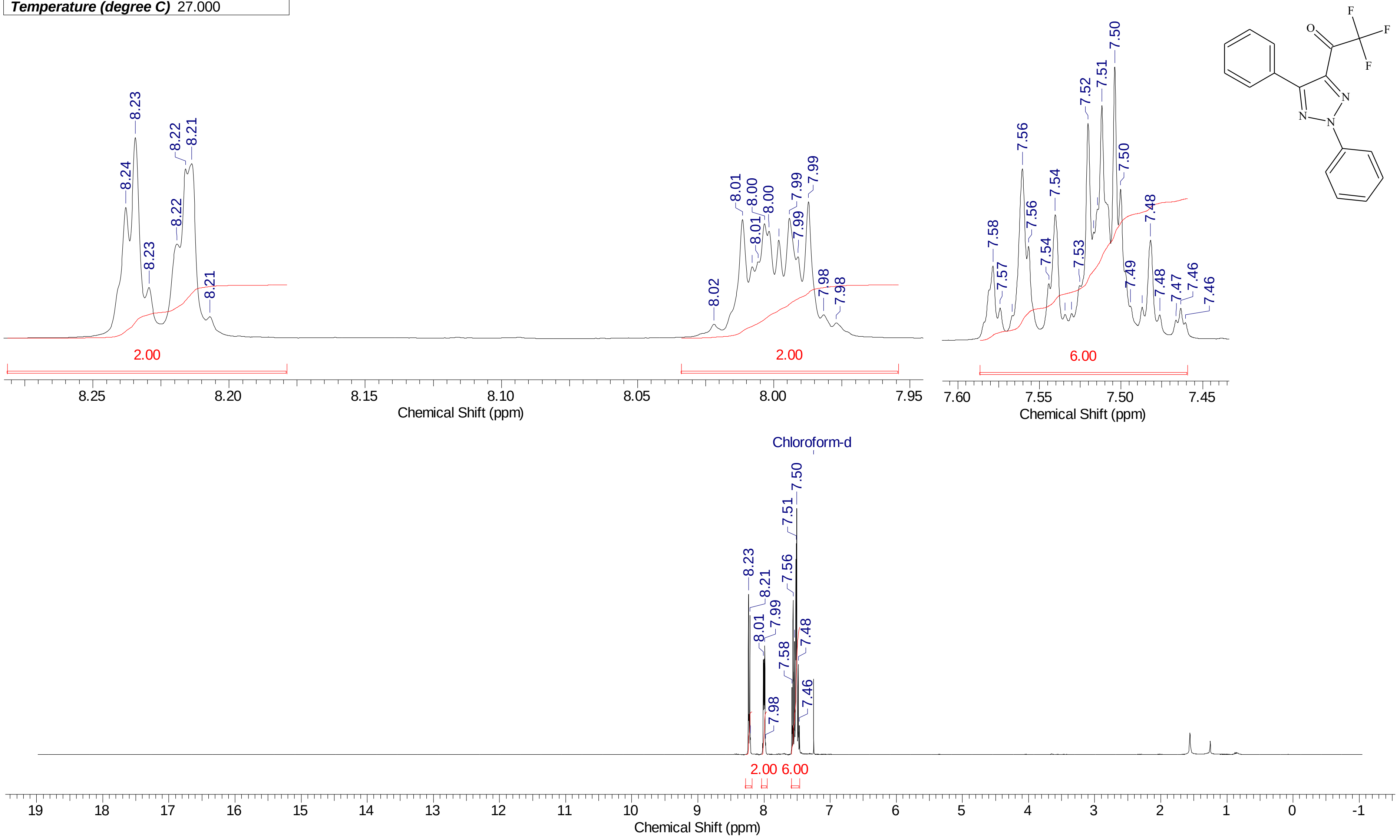

## $^{1}$H NMR spectrum of **11** (400.1 MHz, $CDCl_3$)

19 May 2022

| | | | | | | | |
|---|---|---|---|---|---|---|---|
| ***Acquisition Time (sec)*** | 1.7433 | ***Comment*** | Imported from UXNMR. | | | ***Date*** | 21 Apr 2022 12:05:42 |
| ***File Name*** | C:\DOCS\OUTPUT_301\2022\04.图疱朦\BM-2505-2.F_005001r | | | | | ***Frequency (MHz)*** | 376.50 |
| ***Nucleus*** | 19F | ***Number of Transients*** | 11 | ***Original Points Count*** | 131072 | ***Points Count*** | 262144 |
| ***Pulse Sequence*** | zgflqn | ***Solvent*** | CHLOROFORM-D | | | ***Sweep Width (Hz)*** | 75187.97 |
| ***Temperature (degree C)*** | 27.000 | | | | | | |

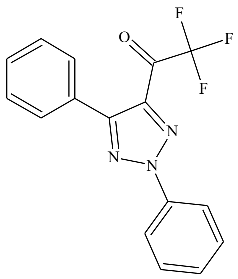

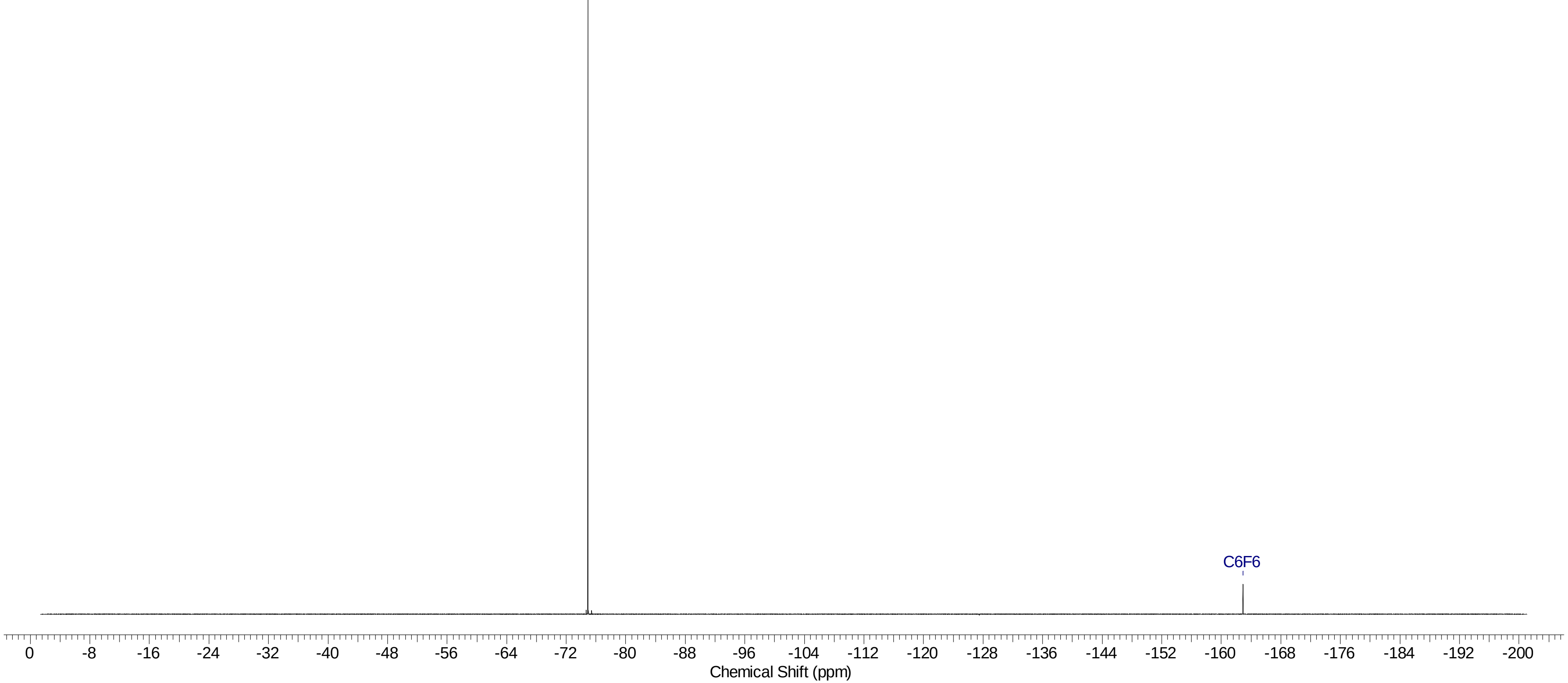

## $^{19}F$ NMR spectrum of **11** (376.5 MHz, $CDCl_3$)

19 May 2022

| | | | | | | | |
|---|---|---|---|---|---|---|---|
| ***Acquisition Time (sec)*** | 0.6783 | ***Comment*** | Imported from UXNMR. | | | ***Date*** | 21 Apr 2022 13:09:40 |
| ***File Name*** | C:\DOCS\OUTPUT_301\2022\04.图疱滕\BM-2505-2.C_002001r | | | | | ***Frequency (MHz)*** | 100.61 |
| ***Nucleus*** | 13C | ***Number of Transients*** | 763 | ***Original Points Count*** | 16384 | ***Points Count*** | 131072 |
| ***Pulse Sequence*** | zgpg30 | ***Solvent*** | CHLOROFORM-D | | | ***Sweep Width (Hz)*** | 24154.59 |
| ***Temperature (degree C)*** | 27.000 | | | | | | |

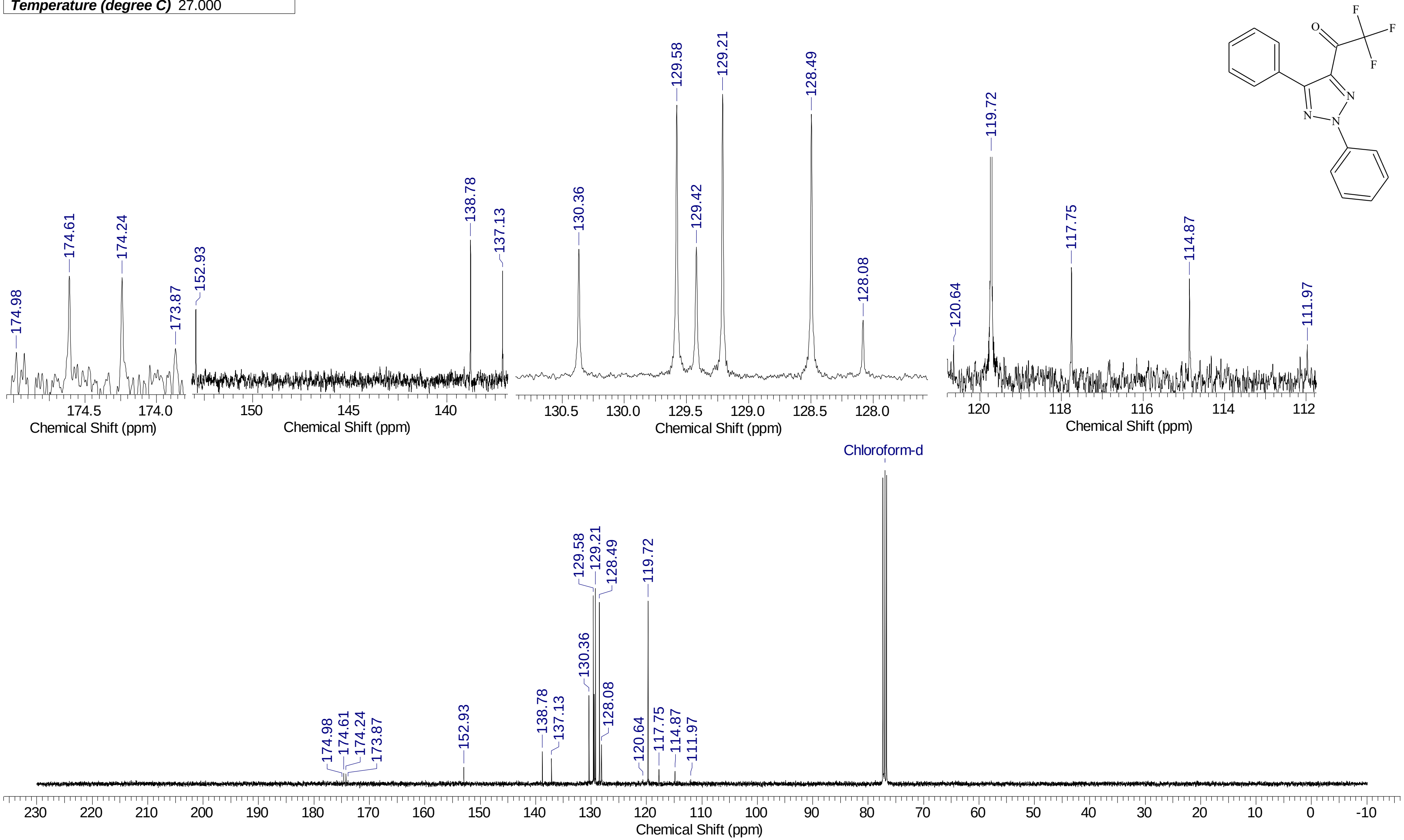

$^{13}$C NMR spectrum of **11** (100.6 MHz, $CDCl_3$)

Copies of FT-IR spectra

8 Nov 2022

| | | | | | | | |
|---|---|---|---|---|---|---|---|
| ***Title*** | 腆 玎脲怦觇? 绿 -2508-3 (窝 湾 磬殇屙觐) iD7 | | | | | | |
| ***File Name*** | C:\DOCS\BM\IR SPECTRA\07-11-2022_15-26-24\腆 玎脲怦觇? 绿 -2508-3 (窝 湾 磬殇屙觐) ID7.SPA | | | | | ***Date Stamp*** | 07 Nov 2022 11:16:05 |
| ***Date*** | 07 Nov 2022 18:49:50 | ***Technique*** | Infrared | ***Spectral Region*** | IR | ***X Axis*** | Wavenumber (cm-1) |
| ***Y Axis*** | Absorbance | ***Spectrum Range*** | 525.0250 - 4000.1229 | ***Points Count*** | 7209 | ***Data Spacing*** | 0.4821 |

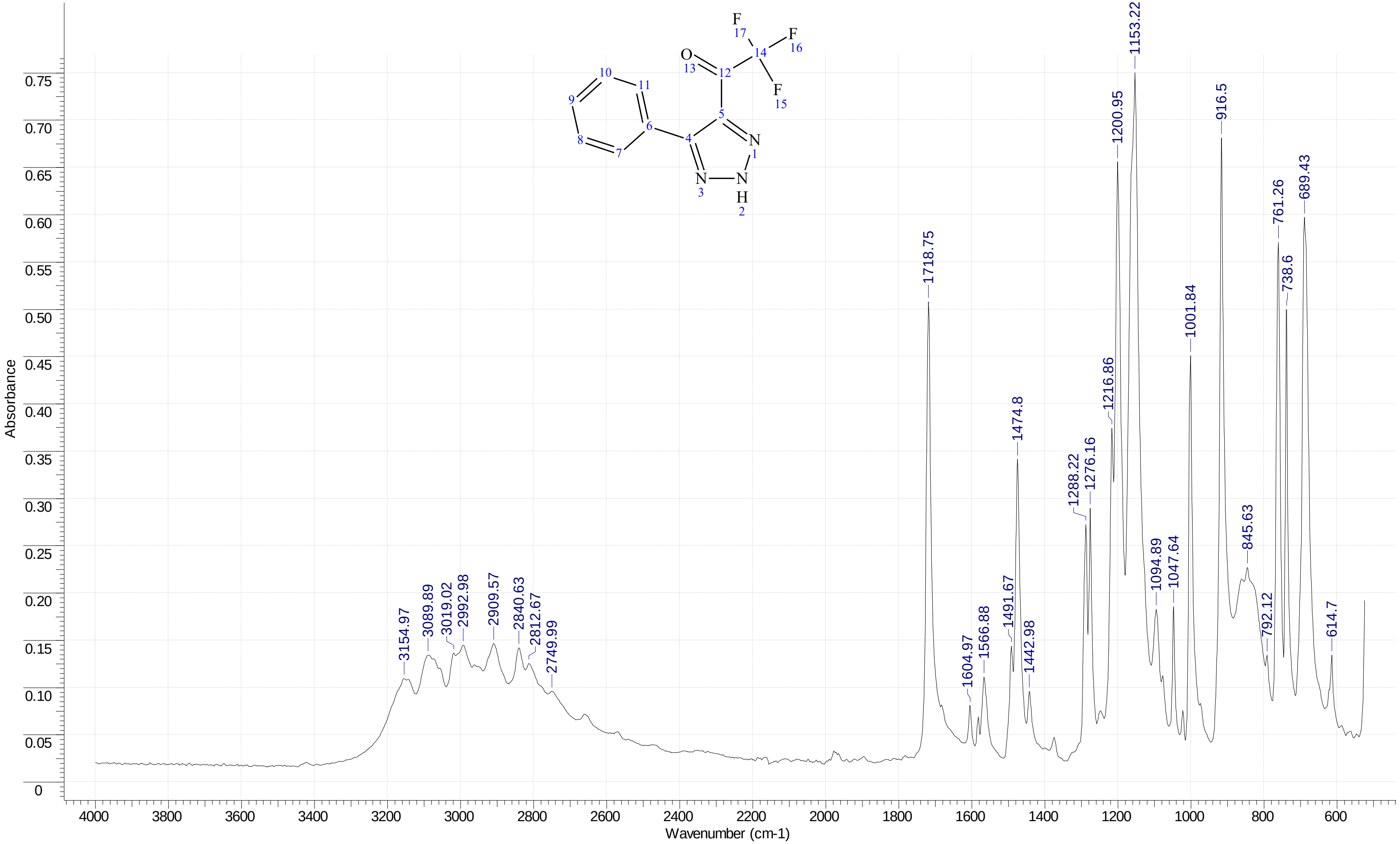

FT-IR spectra of **2a**

8 Nov 2022

| | | | | | | | |
|---|---|---|---|---|---|---|---|
| ***Title*** | 腆 玎脤怦觇? 绿 -2511-2 (窝 湾 磬殇屙觐) iD7 | | | | | | |
| ***File Name*** | C:\DOCS\BM\IR SPECTRA\07-11-2022_15-26-24\腆 玎脤怦觇? 绿 -2511-2 (窝 湾 磬殇屙觐) ID7.SPA | | | | | ***Date Stamp*** | 07 Nov 2022 11:52:12 |
| ***Date*** | 07 Nov 2022 18:49:52 | ***Technique*** | Infrared | ***Spectral Region*** | IR | ***X Axis*** | Wavenumber (cm-1) |
| ***Y Axis*** | Absorbance | ***Spectrum Range*** | 525.0250 - 4000.1229 | ***Points Count*** | 7209 | ***Data Spacing*** | 0.4821 |

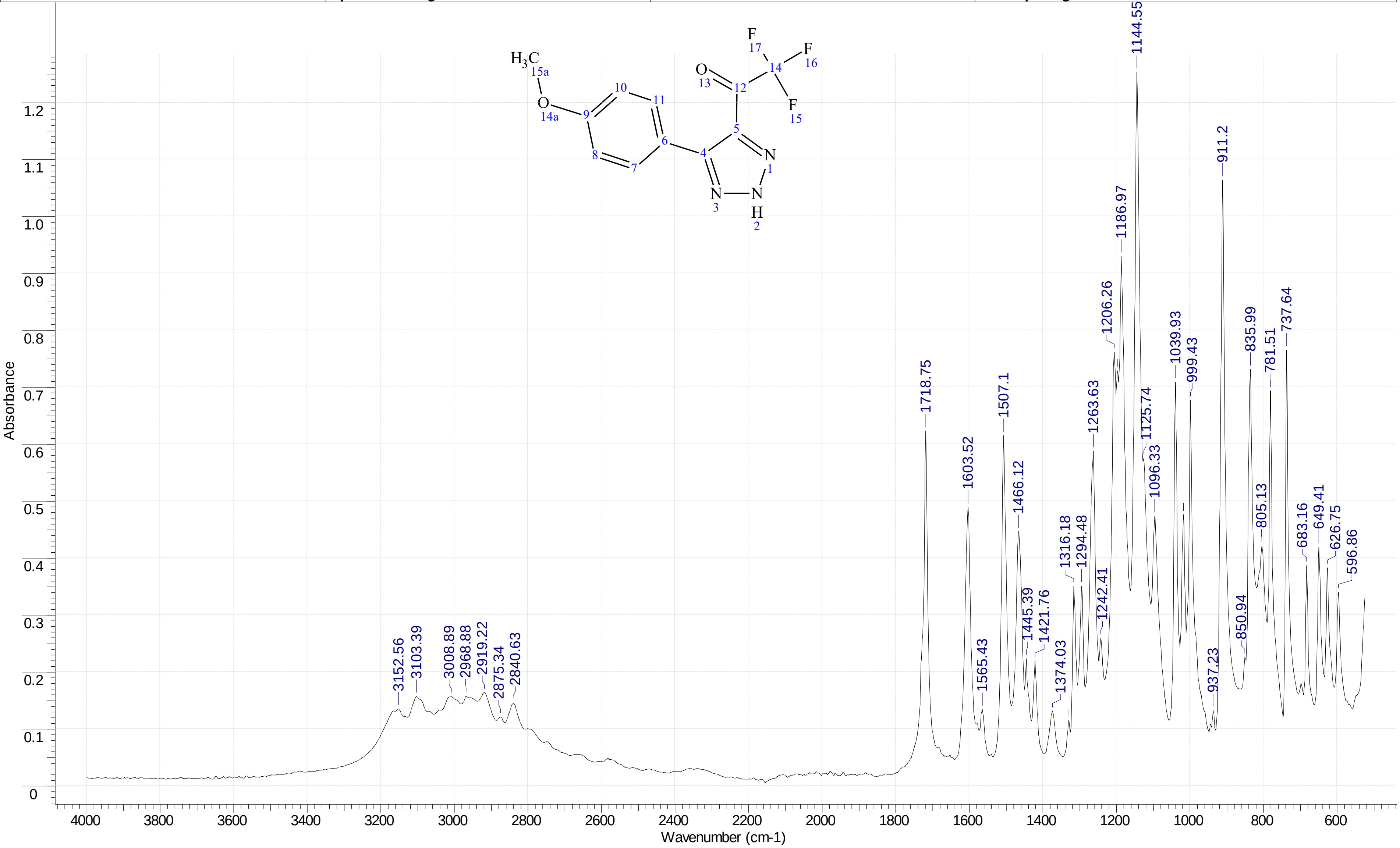

FT-IR spectra of **2b**

8 Nov 2022

| | | | | | | | |
|---|---|---|---|---|---|---|---|
| ***Title*** | 腆 玎豚怦觇? 绿 -2365-3 (窝 湾 磬殇屙觐) iD7 | | | | | | |
| ***File Name*** | C:\DOCS\BM\IR SPECTRA\08-11-2022_13-23-31\腆 玎豚怦觇? 绿 -2365-3 (窝 湾 磬殇屙觐) ID7.SPA | | | | | ***Date Stamp*** | 08 Nov 2022 09:42:25 |
| ***Date*** | 08 Nov 2022 13:56:40 | ***Technique*** | Infrared | ***Spectral Region*** | IR | ***X Axis*** | Wavenumber (cm-1) |
| ***Y Axis*** | Absorbance | ***Spectrum Range*** | 525.0250 - 4000.1229 | ***Points Count*** | 7209 | ***Data Spacing*** | 0.4821 |

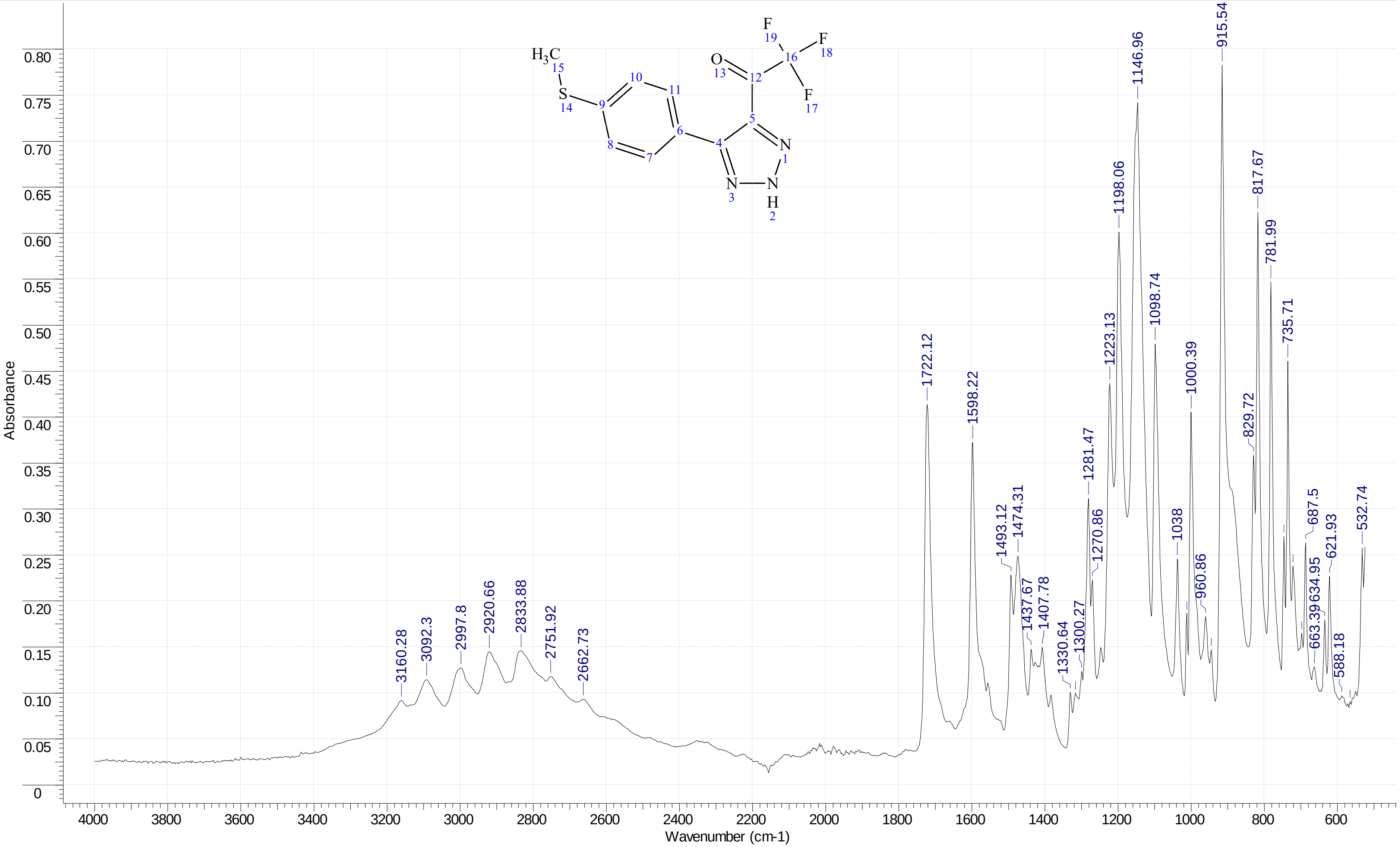


S138

# FT-IR spectra of **2c**

8 Nov 2022

| | | | | | | | |
|---|---|---|---|---|---|---|---|
| ***Title*** | 腆 玎豚怦觇? 绿 -1327-3 (窝 湾 磬殇屙觐) iD7 | | | | | | |
| ***File Name*** | C:\DOCS\BM\IR SPECTRA\07-11-2022_15-26-24\腆 玎豚怦觇? 绿 -1327-3 (窝 湾 磬殇屙觐) ID7.SPA | | | | | ***Date Stamp*** | 07 Nov 2022 11:42:47 |
| ***Date*** | 07 Nov 2022 18:49:52 | ***Technique*** | Infrared | ***Spectral Region*** | IR | ***X Axis*** | Wavenumber (cm-1) |
| ***Y Axis*** | Absorbance | ***Spectrum Range*** | 525.0250 - 4000.1229 | ***Points Count*** | 7209 | ***Data Spacing*** | 0.4821 |

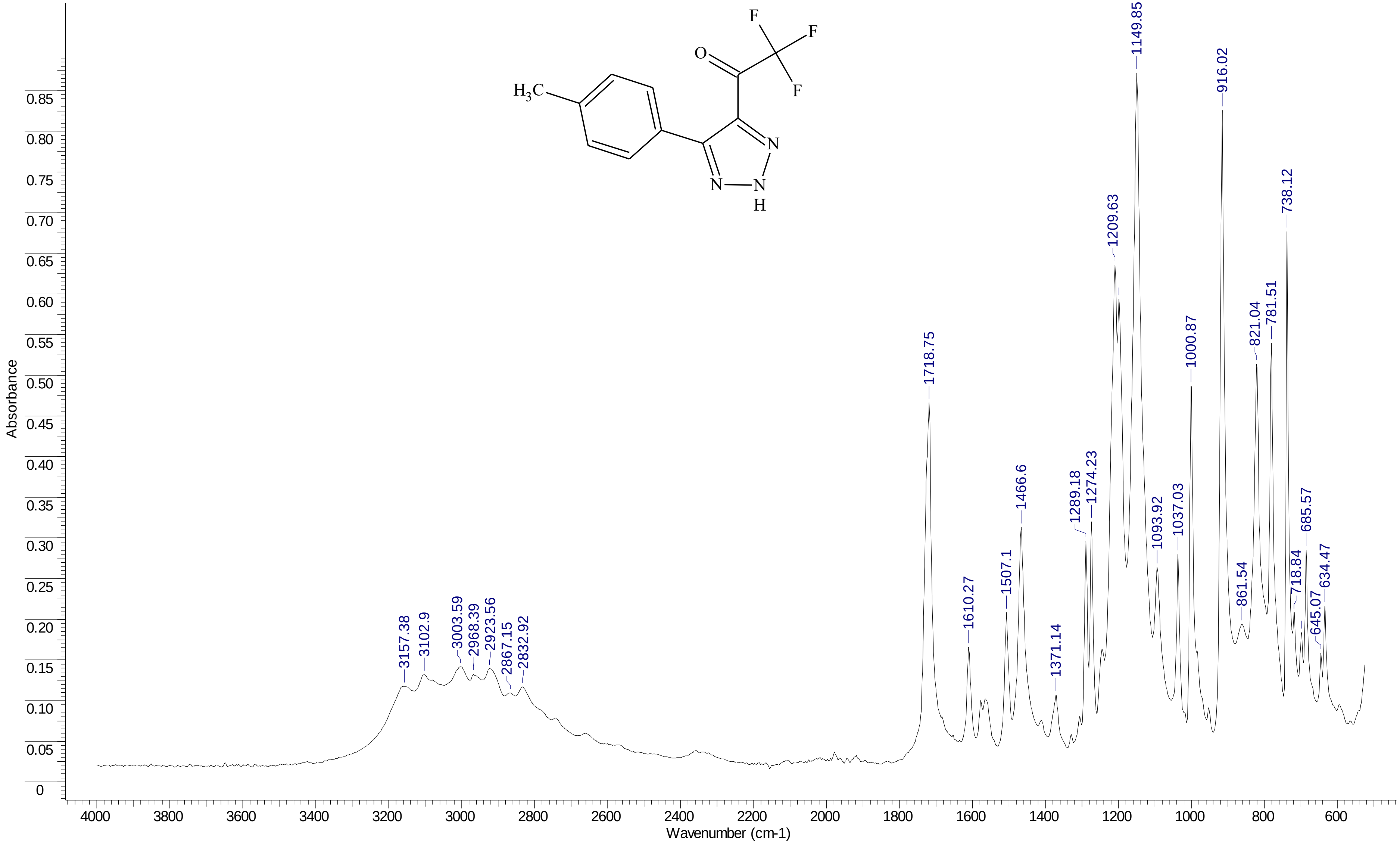

FT-IR spectra of **2d**

8 Nov 2022

| | | | | | | | |
|---|---|---|---|---|---|---|---|
| ***Title*** | 腆 玎脤怦觇? 绿 -2214-2 (寓 湾 磬殇屙觐) iD7 | | | | | | |
| ***File Name*** | C:\DOCS\BM\IR SPECTRA\07-11-2022_15-26-24\腆 玎脤怦觇? 绿 -2214-2 (寓 湾 磬殇屙觐) ID7.SPA | | | | | ***Date Stamp*** | 07 Nov 2022 11:47:33 |
| ***Date*** | 07 Nov 2022 18:49:52 | ***Technique*** | Infrared | ***Spectral Region*** | IR | ***X Axis*** | Wavenumber (cm-1) |
| ***Y Axis*** | Absorbance | ***Spectrum Range*** | 525.0250 - 4000.1229 | ***Points Count*** | 7209 | ***Data Spacing*** | 0.4821 |

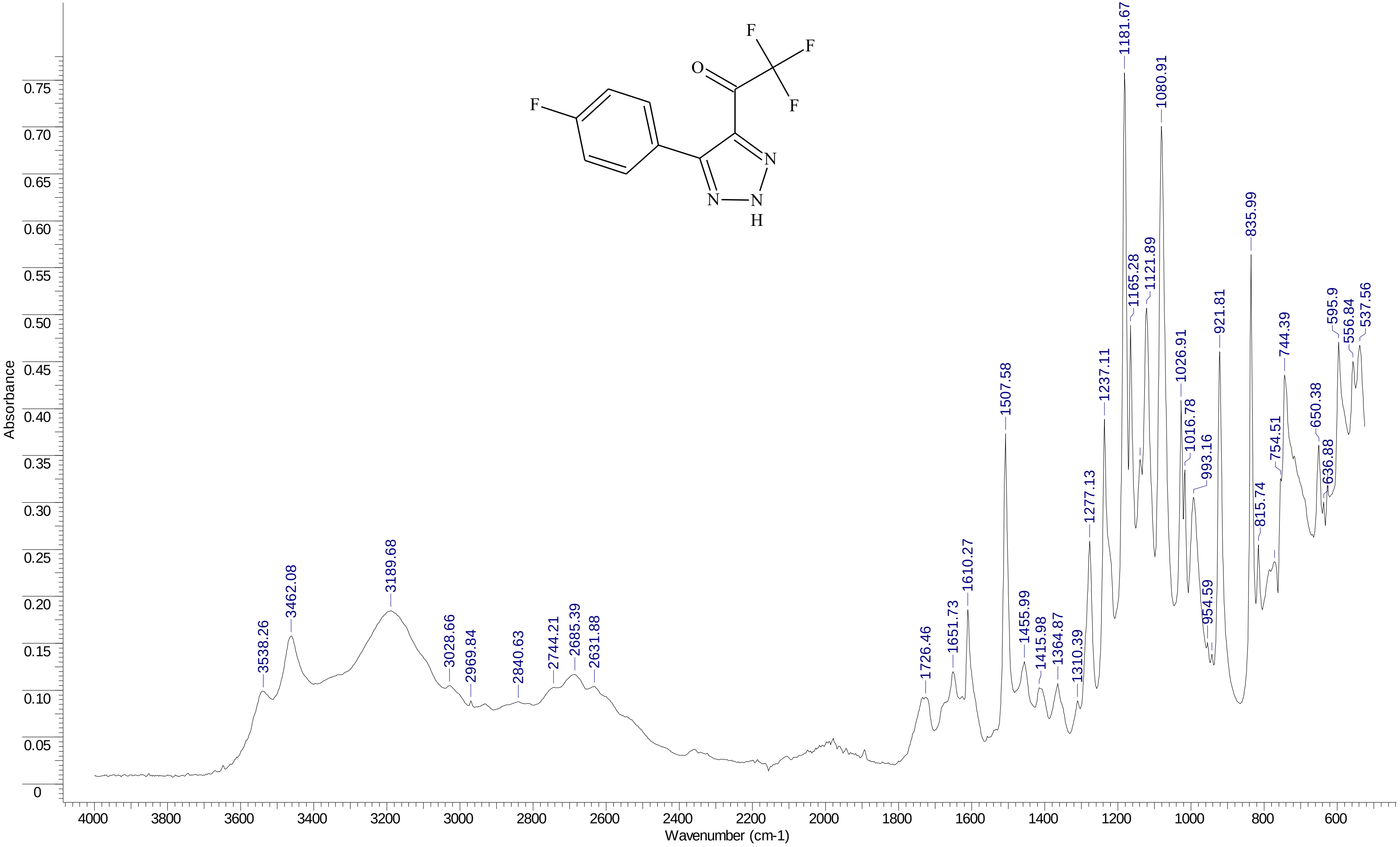

FT-IR spectra of **2e**

8 Nov 2022

| | | | | | | | |
|---|---|---|---|---|---|---|---|
| ***Title*** | 腆 玎脲怦觇? 绿 -2357-3 (窝 湾 磬殇屙觐) iD7 | | | | | | |
| ***File Name*** | C:\DOCS\BM\IR SPECTRA\07-11-2022_15-26-24\腆 玎脲怦觇? 绿 -2357-3 (窝 湾 磬殇屙觐) ID7.SPA | | | | | ***Date Stamp*** | 07 Nov 2022 12:02:20 |
| ***Date*** | 07 Nov 2022 18:49:50 | ***Technique*** | Infrared | ***Spectral Region*** | IR | ***X Axis*** | Wavenumber (cm-1) |
| ***Y Axis*** | Absorbance | ***Spectrum Range*** | 525.0250 - 4000.1229 | ***Points Count*** | 7209 | ***Data Spacing*** | 0.4821 |

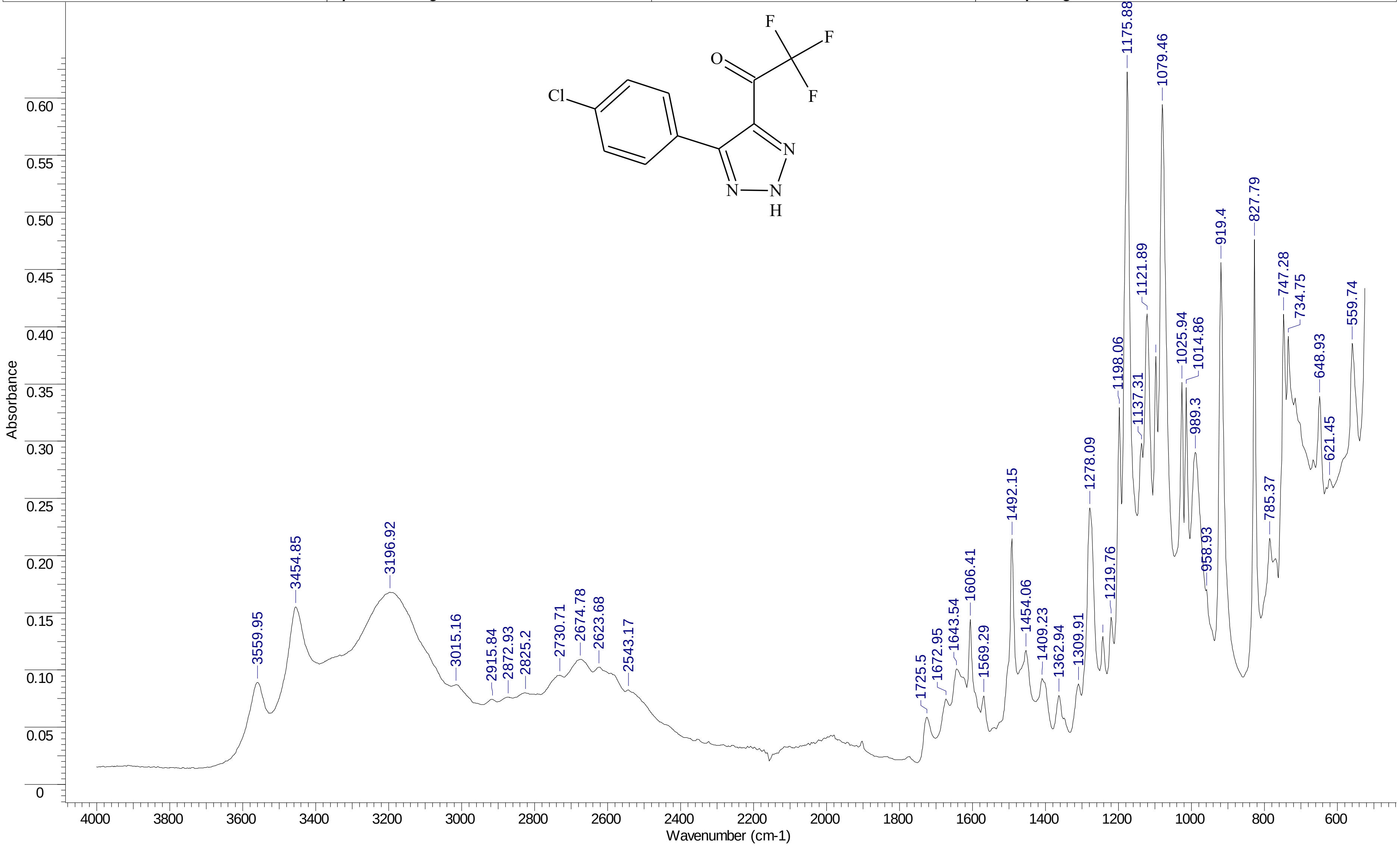

FT-IR spectra of **2f**

8 Nov 2022

| | | | | | | | |
|---|---|---|---|---|---|---|---|
| ***Title*** | 腆 玎脤怦觇? 绿 -2363 (窝 湾 磬殇屙觐) iD7 | | | | | | |
| ***File Name*** | C:\DOCS\BM\IR SPECTRA\07-11-2022_15-26-24\腆 玎脤怦觇? 绿 -2363 (窝 湾 磬殇屙觐) ID7.SPA | | | | | ***Date Stamp*** | 07 Nov 2022 11:29:32 |
| ***Date*** | 07 Nov 2022 18:49:52 | ***Technique*** | Infrared | ***Spectral Region*** | IR | ***X Axis*** | Wavenumber (cm-1) |
| ***Y Axis*** | Absorbance | ***Spectrum Range*** | 525.0250 - 4000.1229 | ***Points Count*** | 7209 | ***Data Spacing*** | 0.4821 |

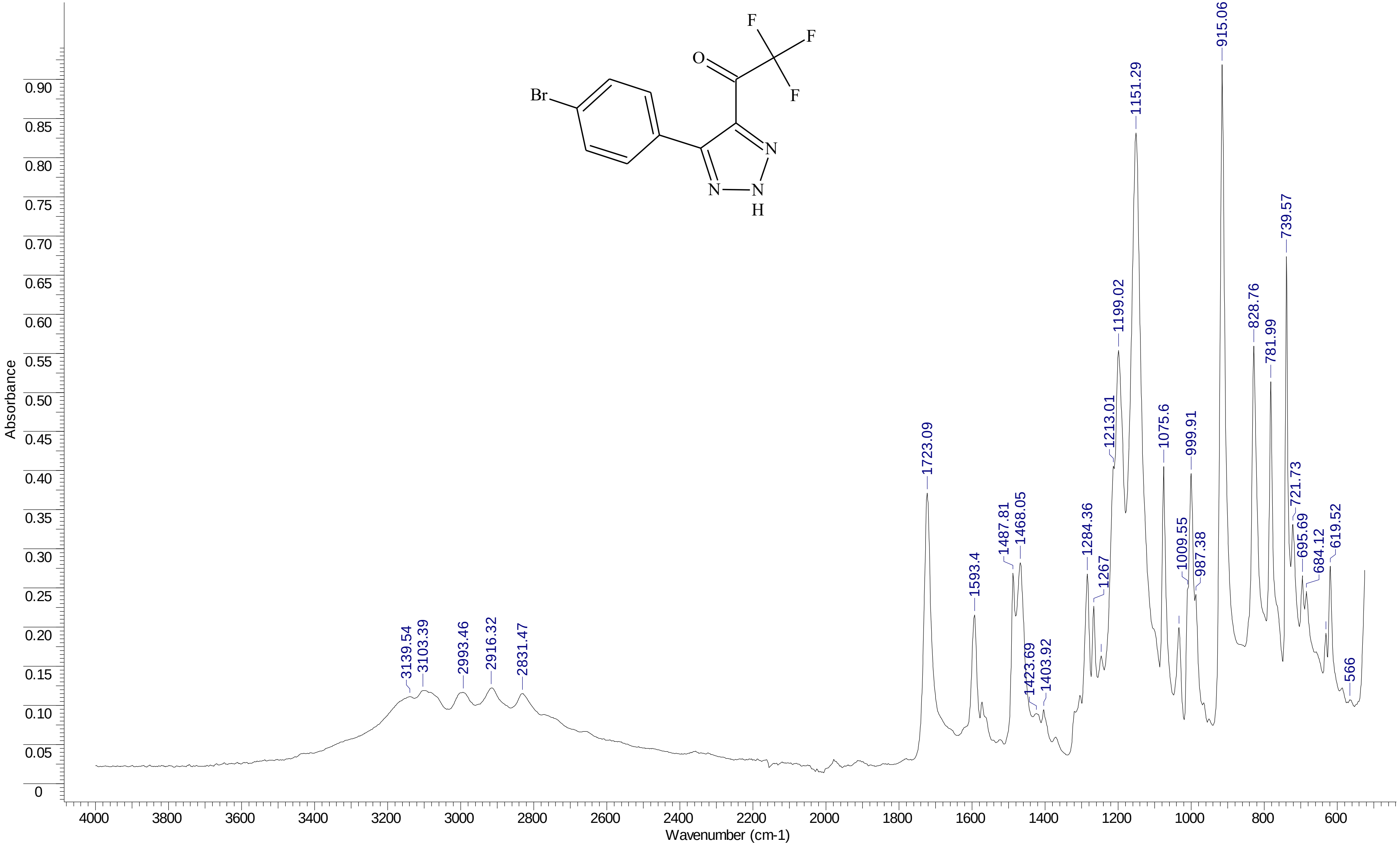

# FT-IR spectra of **2g**

8 Nov 2022

| | | | | | | | |
|---|---|---|---|---|---|---|---|
| ***Title*** | 腆 玎脲怦觇? 绿 -2374 (窝 湾 磬殇扃觐) iD7 | | | | | | |
| ***File Name*** | C:\DOCS\BM\IR SPECTRA\08-11-2022_13-23-31\腆 玎脲怦觇? 绿 -2374 (窝 湾 磬殇扃觐) ID7.SPA | | | | | ***Date Stamp*** | 08 Nov 2022 09:19:57 |
| ***Date*** | 08 Nov 2022 13:56:38 | ***Technique*** | Infrared | ***Spectral Region*** | IR | ***X Axis*** | Wavenumber (cm-1) |
| ***Y Axis*** | Absorbance | ***Spectrum Range*** | 525.0250 - 4000.1229 | ***Points Count*** | 7209 | ***Data Spacing*** | 0.4821 |

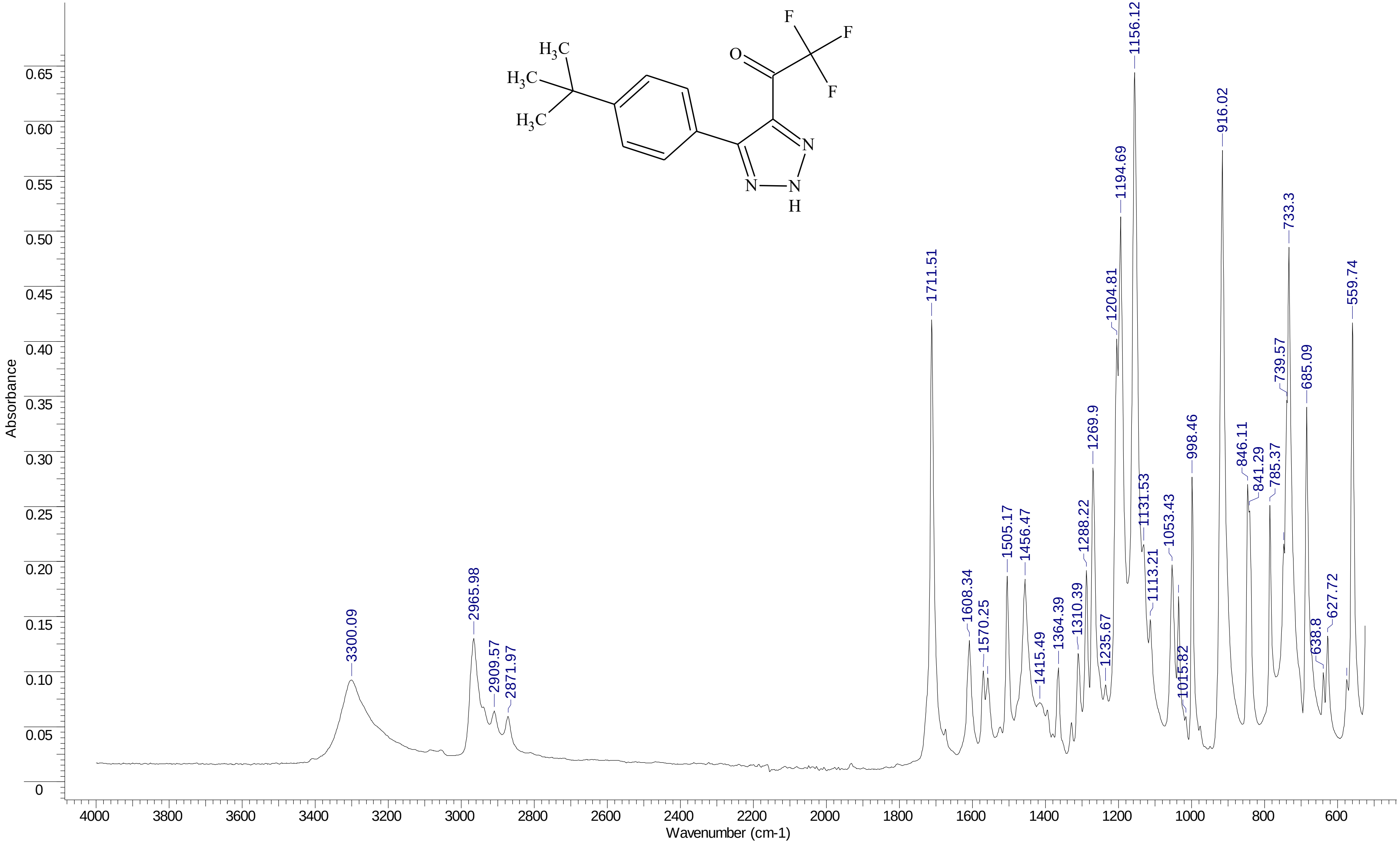

FT-IR spectra of **2h**

8 Nov 2022

| | | | | | | | |
|---|---|---|---|---|---|---|---|
| ***Title*** | 腆 玎脲怦觇? 绿 -2362-3 (窝 湾 磬殇屙觐) iD7 | | | | | | |
| ***File Name*** | C:\DOCS\BM\IR SPECTRA\08-11-2022_13-23-31\腆 玎脲怦觇? 绿 -2362-3 (窝 湾 磬殇屙觐) ID7.SPA | | | | | ***Date Stamp*** | 08 Nov 2022 10:18:30 |
| ***Date*** | 08 Nov 2022 13:56:38 | ***Technique*** | Infrared | ***Spectral Region*** | IR | ***X Axis*** | Wavenumber (cm-1) |
| ***Y Axis*** | Absorbance | ***Spectrum Range*** | 525.0250 - 4000.1229 | ***Points Count*** | 7209 | ***Data Spacing*** | 0.4821 |

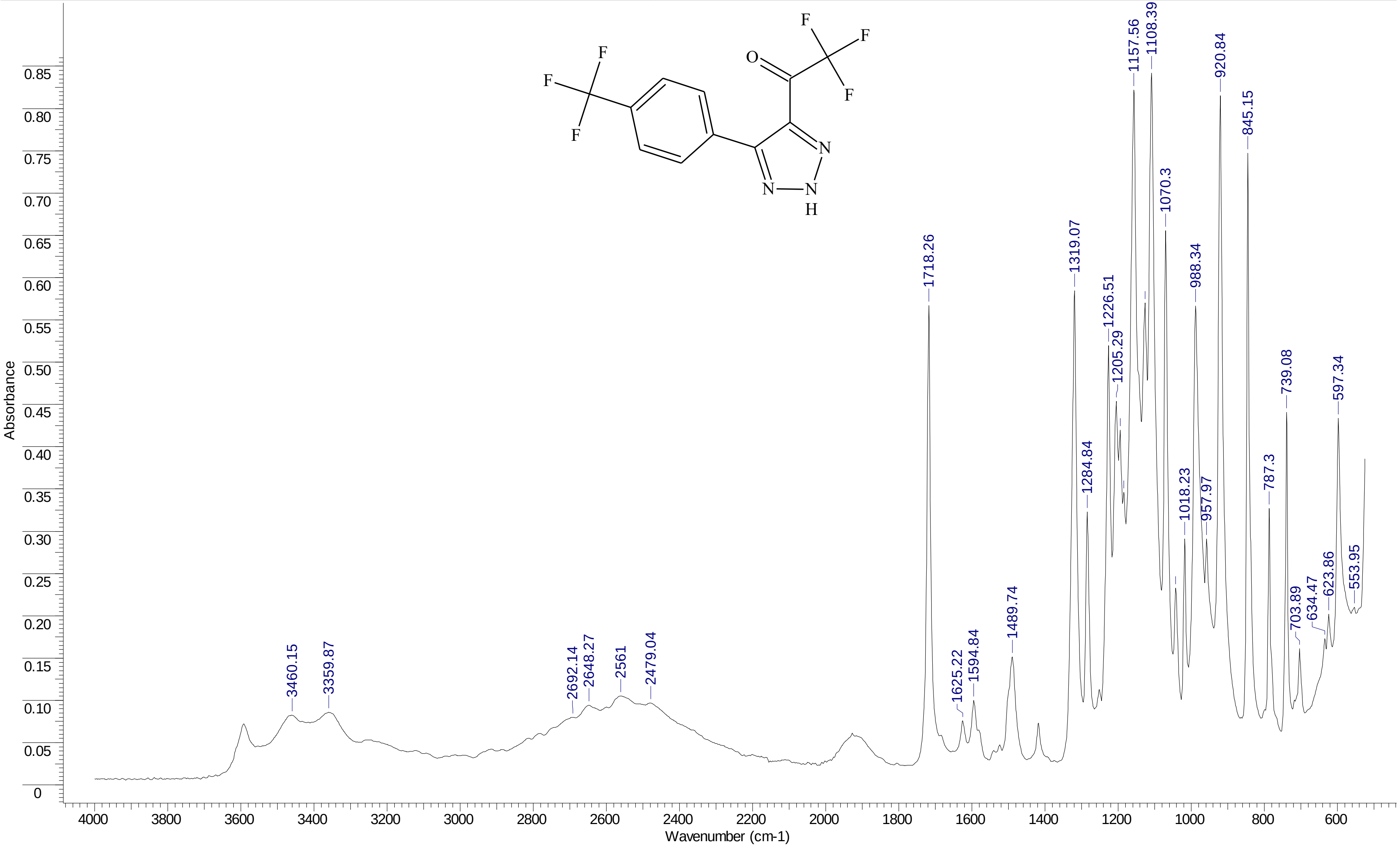

FT-IR spectra of **2i**

8 Nov 2022

| | | | | | | | |
|---|---|---|---|---|---|---|---|
| ***Title*** | 腆 玎脤怦觇? 绿 -2364-3 (窝 湾 罄殇屙觐) iD7 | | | | | | |
| ***File Name*** | C:\DOCS\BM\IR SPECTRA\08-11-2022_13-23-31\腆 玎脤怦觇? 绿 -2364-3 (窝 湾 罄殇屙觐) ID7.SPA | | | | | ***Date Stamp*** | 08 Nov 2022 09:56:29 |
| ***Date*** | 08 Nov 2022 13:56:38 | ***Technique*** | Infrared | ***Spectral Region*** | IR | ***X Axis*** | Wavenumber (cm-1) |
| ***Y Axis*** | Absorbance | ***Spectrum Range*** | 525.0250 - 4000.1229 | ***Points Count*** | 7209 | ***Data Spacing*** | 0.4821 |

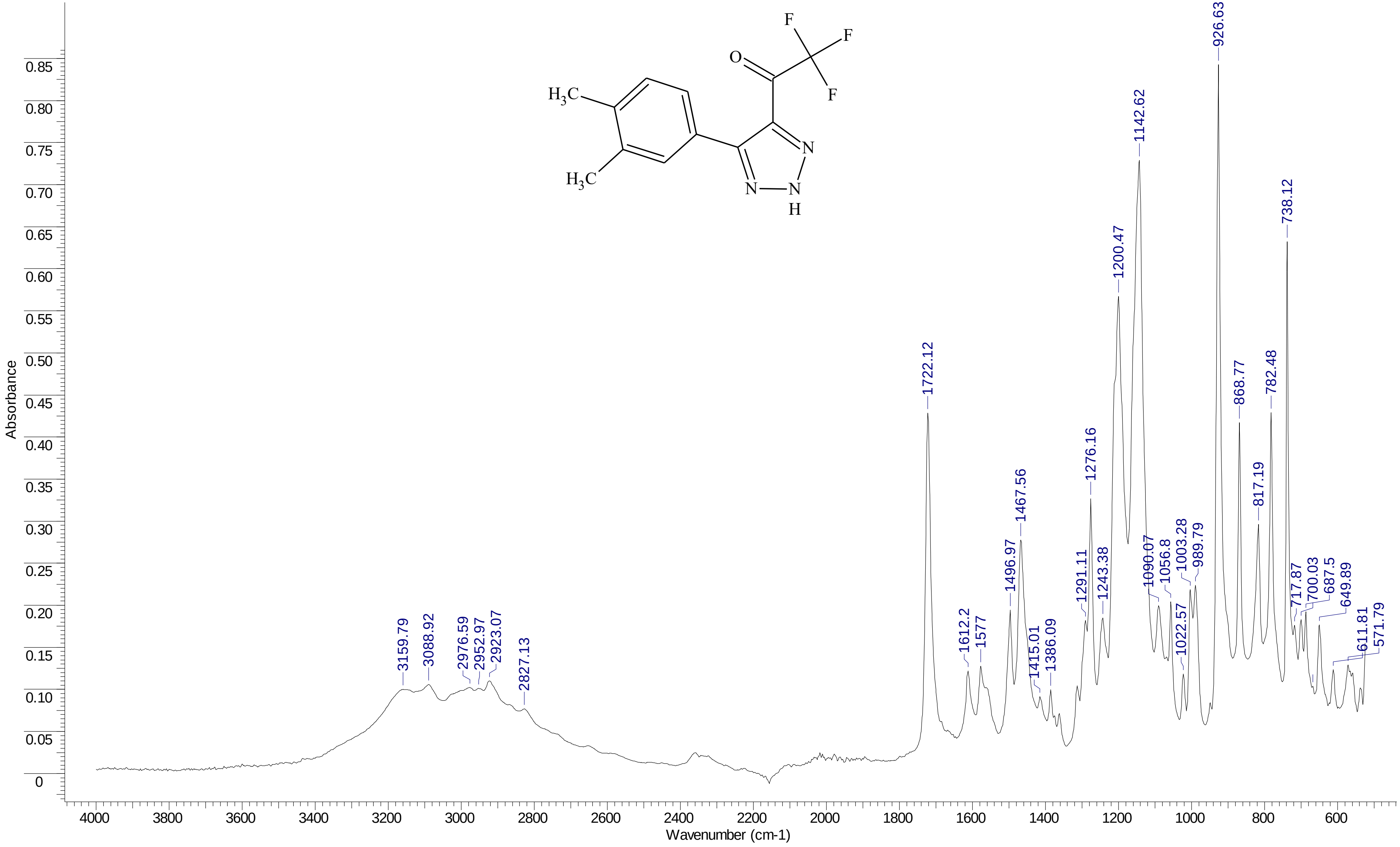

FT-IR spectra of **2j**

8 Nov 2022

| | | | | | | | |
|---|---|---|---|---|---|---|---|
| ***Title*** | 腆 玎豚怦觇? 绿 -2359 (窝 湾 磬殇屙觐) iD7 | | | | | | |
| ***File Name*** | C:\DOCS\BM\IR SPECTRA\08-11-2022_13-23-31\腆 玎豚怦觇? 绿 -2359 (窝 湾 磬殇屙觐) ID7.SPA | | | | | ***Date Stamp*** | 08 Nov 2022 09:38:16 |
| ***Date*** | 08 Nov 2022 13:56:40 | ***Technique*** | Infrared | ***Spectral Region*** | IR | ***X Axis*** | Wavenumber (cm-1) |
| ***Y Axis*** | Absorbance | ***Spectrum Range*** | 525.0250 - 4000.1229 | ***Points Count*** | 7209 | ***Data Spacing*** | 0.4821 |

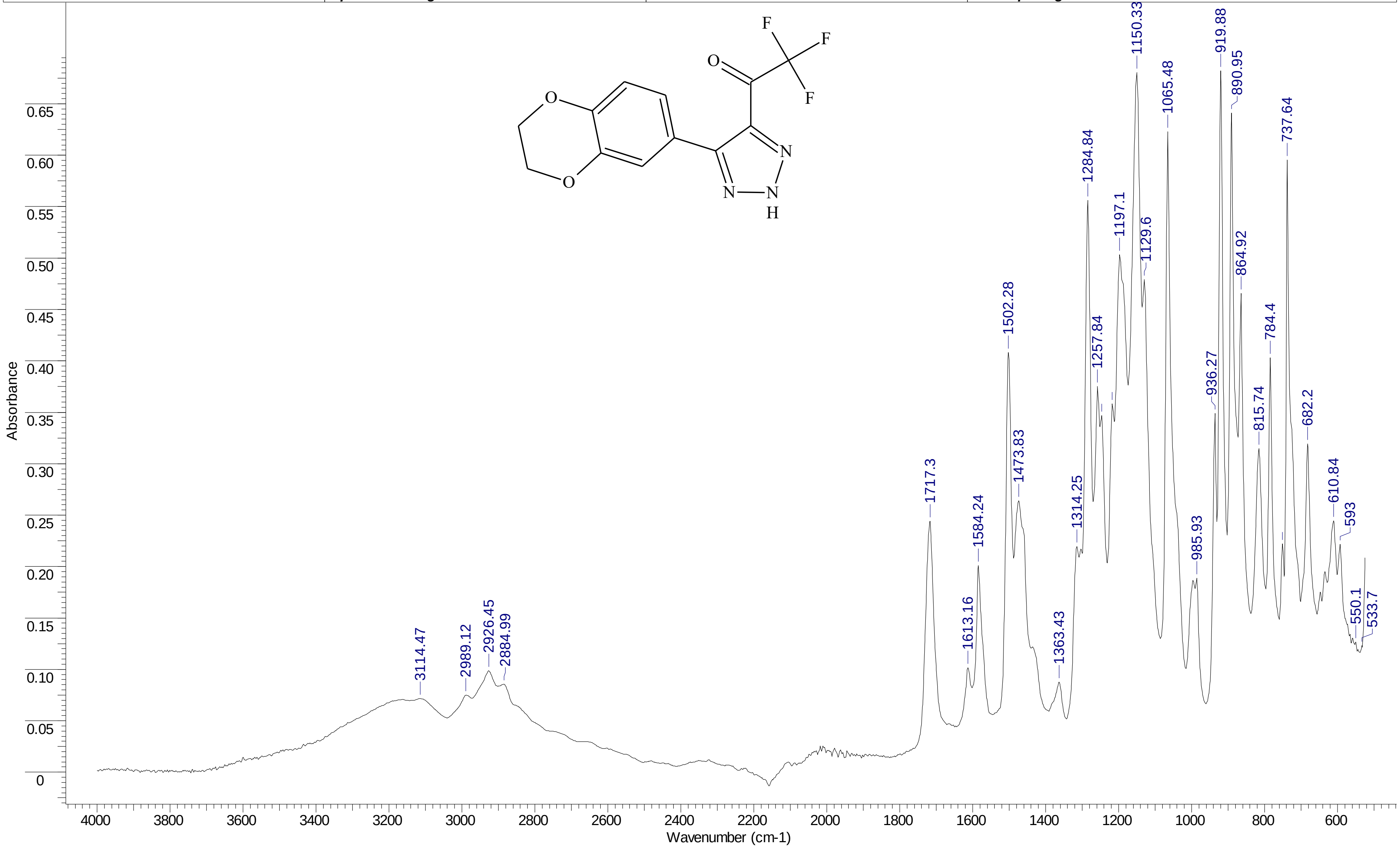

FT-IR spectra of **2k**

8 Nov 2022

| | | | | | | | |
|---|---|---|---|---|---|---|---|
| ***Title*** | 腆 玎豚怦觇? 绿 -2358-3 (窝 湾 磬殇屙觐) iD7 | | | | | | |
| ***File Name*** | C:\DOCS\BM\IR SPECTRA\07-11-2022_15-26-24\腆 玎豚怦觇? 绿 -2358-3 (窝 湾 磬殇屙觐) ID7.SPA | | | | | ***Date Stamp*** | 07 Nov 2022 12:19:57 |
| ***Date*** | 07 Nov 2022 18:49:50 | ***Technique*** | Infrared | ***Spectral Region*** | IR | ***X Axis*** | Wavenumber (cm-1) |
| ***Y Axis*** | Absorbance | ***Spectrum Range*** | 525.0250 - 4000.1229 | ***Points Count*** | 7209 | ***Data Spacing*** | 0.4821 |

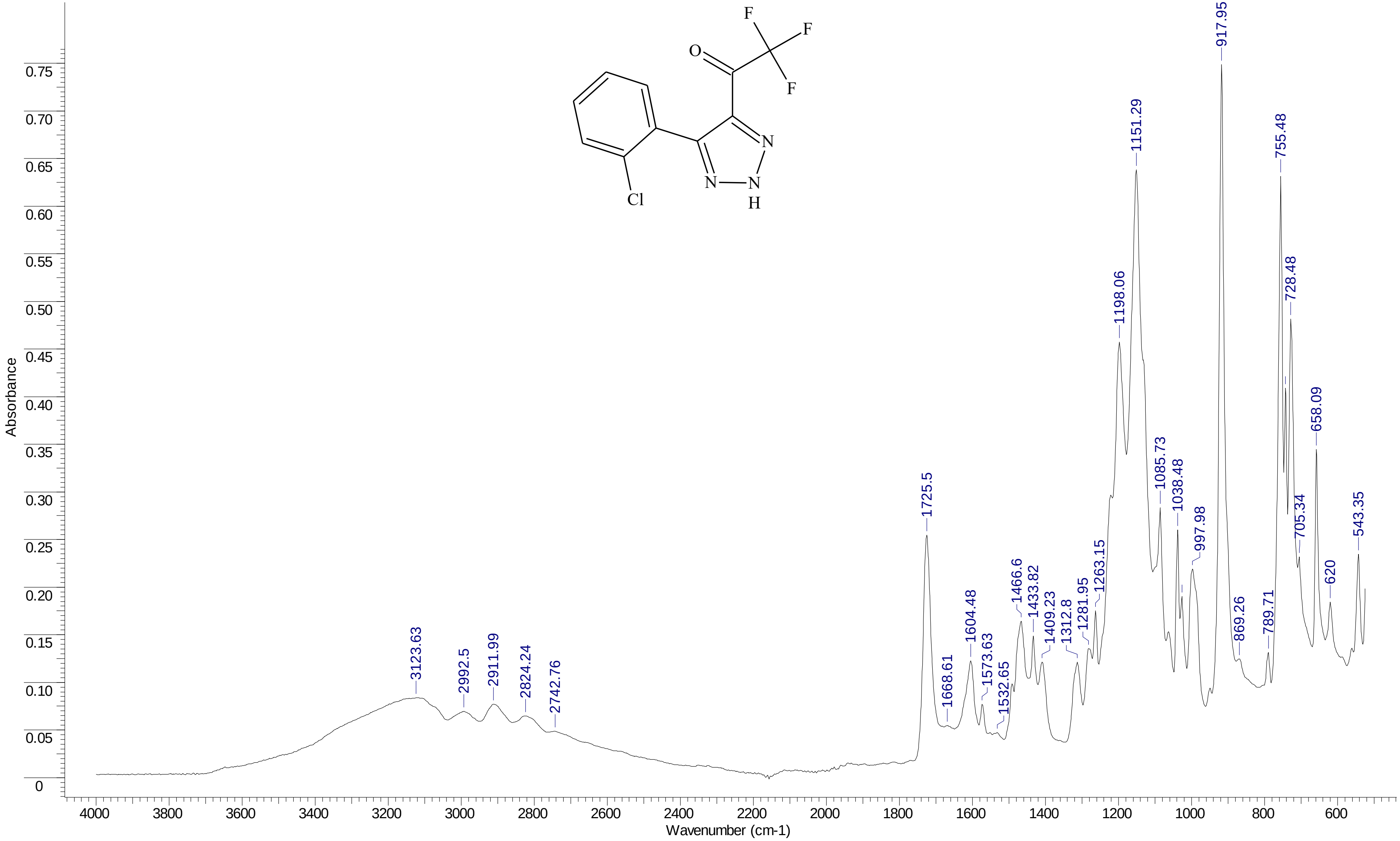

FT-IR spectra of **2l**

8 Nov 2022

| | | | | | | | |
|---|---|---|---|---|---|---|---|
| ***Title*** | 腆 玎豚怦觇? 绿 -2361 (窝 湾 磬殇扃觐) iD7 | | | | | | |
| ***File Name*** | C:\DOCS\BM\IR SPECTRA\07-11-2022_15-26-24\腆 玎豚怦觇? 绿 -2361 (窝 湾 磬殇扃觐) ID7.SPA | | | | | ***Date Stamp*** | 07 Nov 2022 12:08:13 |
| ***Date*** | 07 Nov 2022 18:49:50 | ***Technique*** | Infrared | ***Spectral Region*** | IR | ***X Axis*** | Wavenumber (cm-1) |
| ***Y Axis*** | Absorbance | ***Spectrum Range*** | 525.0250 - 4000.1229 | ***Points Count*** | 7209 | ***Data Spacing*** | 0.4821 |

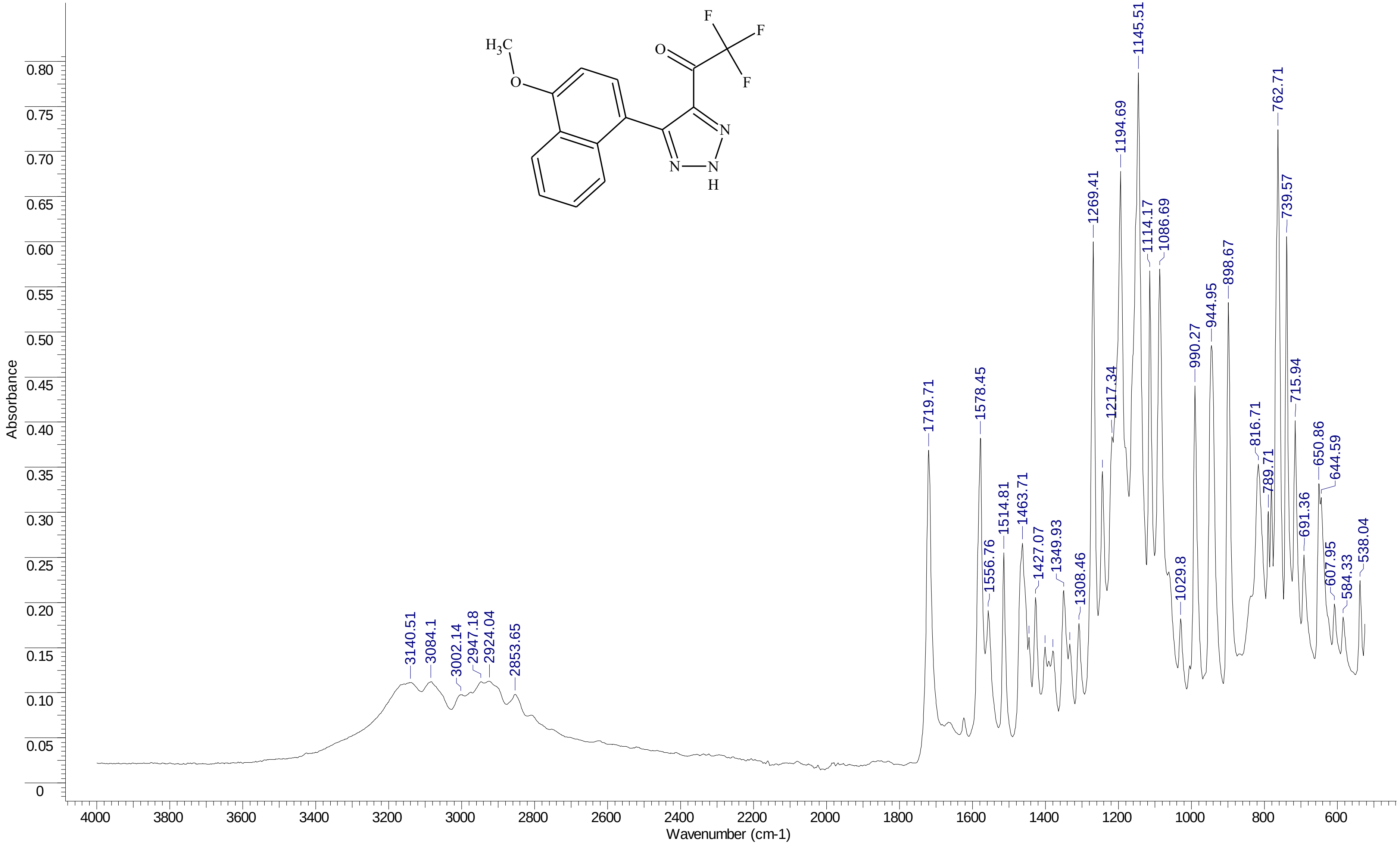

FT-IR spectra of **2n**

8 Nov 2022

| | | | | | | | |
|---|---|---|---|---|---|---|---|
| ***Title*** | 腆 玎脤怦觇? 绿 -2356-3 (窝 湾 磬殇屙觐) iD7 | | | | | | |
| ***File Name*** | C:\DOCS\BM\IR SPECTRA\08-11-2022_13-23-31\腆 玎脤怦觇? 绿 -2356-3 (窝 湾 磬殇屙觐) ID7.SPA | | | | | ***Date Stamp*** | 08 Nov 2022 10:06:09 |
| ***Date*** | 08 Nov 2022 13:56:38 | ***Technique*** | Infrared | ***Spectral Region*** | IR | ***X Axis*** | Wavenumber (cm-1) |
| ***Y Axis*** | Absorbance | ***Spectrum Range*** | 525.0250 - 4000.1229 | ***Points Count*** | 7209 | ***Data Spacing*** | 0.4821 |

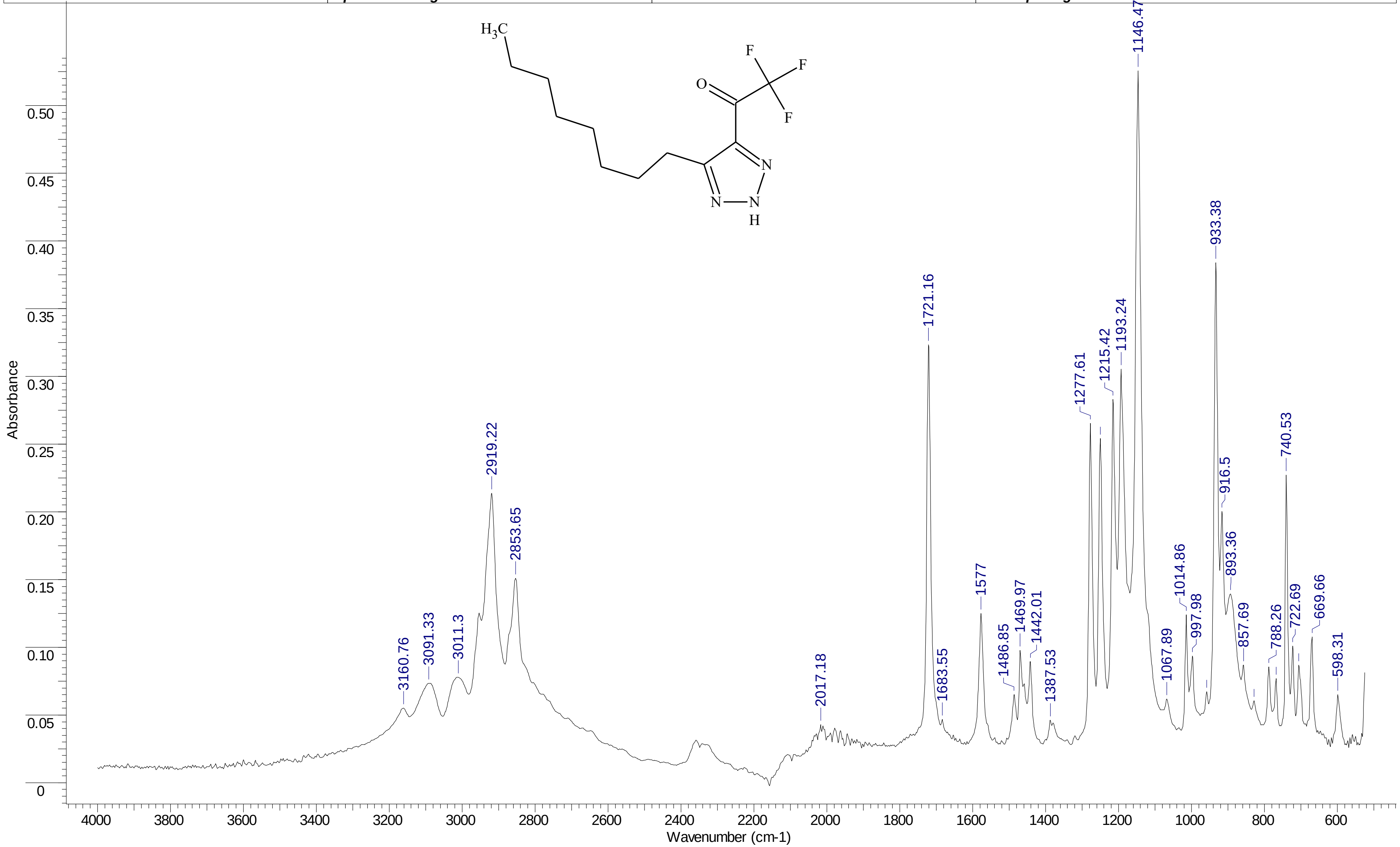

FT-IR spectra of **2o**

8 Nov 2022

| | | | | | | | |
|---|---|---|---|---|---|---|---|
| ***Title*** | *腆 玎脲怦觇? 绿 -2447-3 (窝 湾 磬殇屙觐) iD7 | | | | | | |
| ***File Name*** | C:\DOCS\BM\IR SPECTRA\07-11-2022_15-26-24\腆 玎脲怦觇? 绿 -2447-3 (窝 湾 磬殇屙觐) ID7.SPA | | | | | ***Date Stamp*** | 07 Nov 2022 11:20:07 |
| ***Date*** | 07 Nov 2022 18:49:52 | ***Technique*** | Infrared | ***Spectral Region*** | IR | ***X Axis*** | Wavenumber (cm-1) |
| ***Y Axis*** | Absorbance | ***Spectrum Range*** | 525.0250 - 4000.1229 | ***Points Count*** | 7209 | ***Data Spacing*** | 0.4821 |

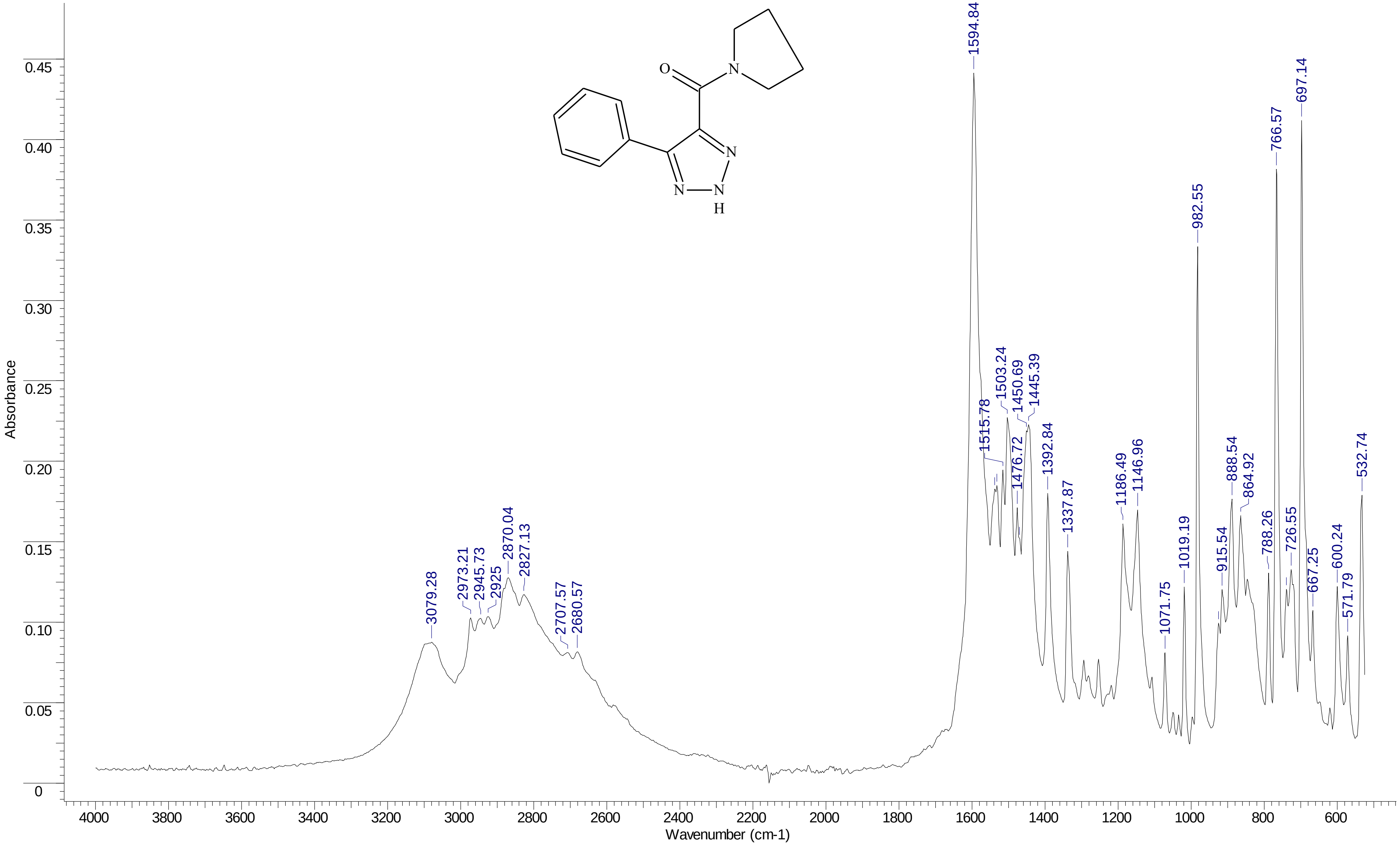

# FT-IR spectra of **4**

8 Nov 2022

| | | | | | | | |
|---|---|---|---|---|---|---|---|
| ***Title*** | 腆 玎脲忤觇? 绿 -2449-2? (窝 湾 磬殇屙觐) iD7 | | | | | | |
| ***File Name*** | C:\DOCS\BM\IR SPECTRA\07-11-2022_15-26-24\腆 玎脲忤觇? 绿 -2449-2? (窝 湾 磬殇屙觐) ID7.SPA | | | | | ***Date Stamp*** | 07 Nov 2022 11:38:08 |
| ***Date*** | 07 Nov 2022 18:49:52 | ***Technique*** | Infrared | ***Spectral Region*** | IR | ***X Axis*** | Wavenumber (cm-1) |
| ***Y Axis*** | Absorbance | ***Spectrum Range*** | 525.0250 - 4000.1229 | ***Points Count*** | 7209 | ***Data Spacing*** | 0.4821 |

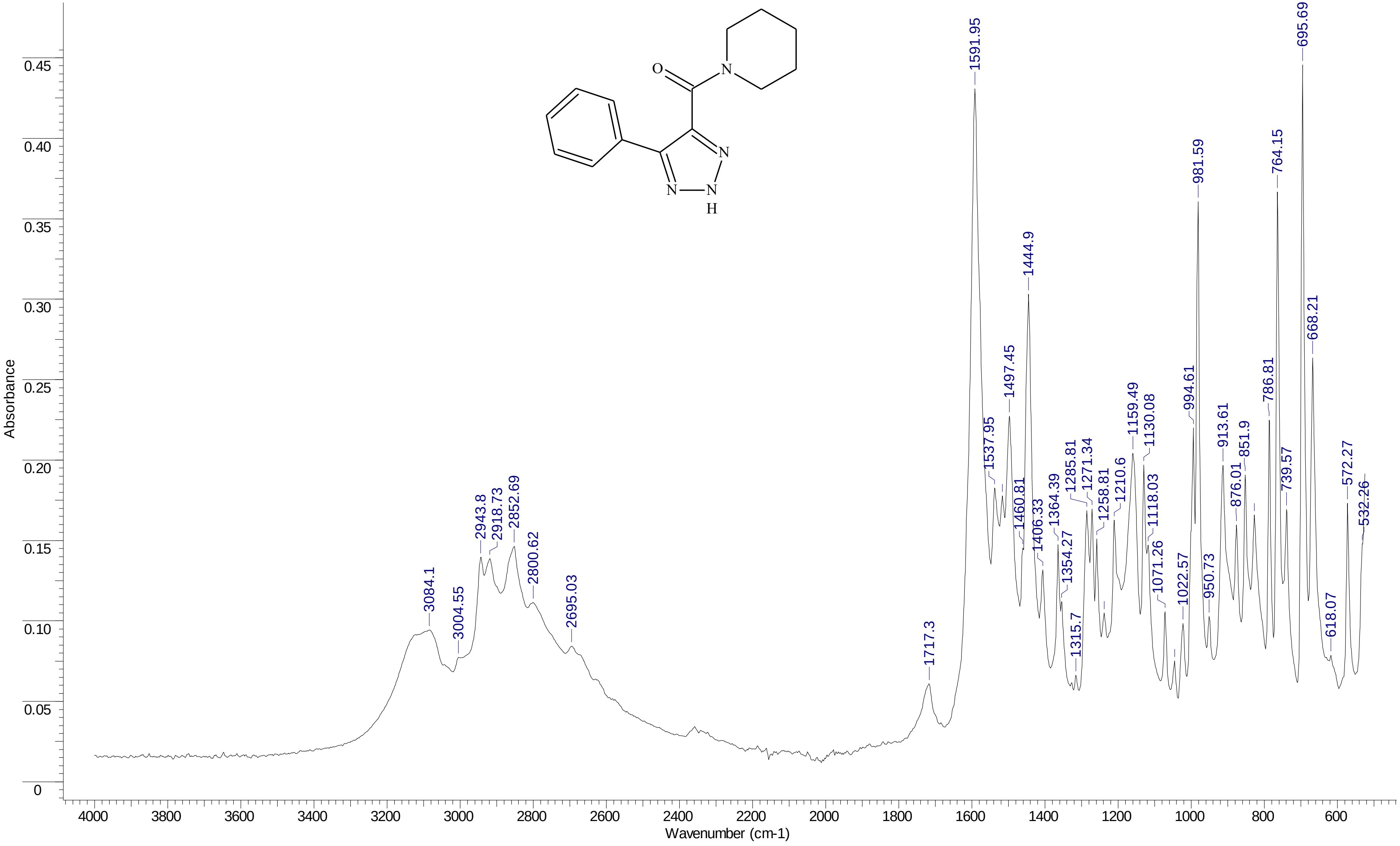

FT-IR spectra of **5**

8 Nov 2022

| | | | | | | | |
|---|---|---|---|---|---|---|---|
| ***Title*** | 腆 玎豚怦觇? 绿 -2450 (窝 湾 磬殇屙觐) iD7 | | | | | | |
| ***File Name*** | C:\DOCS\BM\IR SPECTRA\08-11-2022_13-23-31\腆 玎豚怦觇? 绿 -2450 (窝 湾 磬殇屙觐) ID7.SPA | | | | | ***Date Stamp*** | 08 Nov 2022 09:29:32 |
| ***Date*** | 08 Nov 2022 13:56:38 | ***Technique*** | Infrared | ***Spectral Region*** | IR | ***X Axis*** | Wavenumber (cm-1) |
| ***Y Axis*** | Absorbance | ***Spectrum Range*** | 525.0250 - 4000.1229 | ***Points Count*** | 7209 | ***Data Spacing*** | 0.4821 |

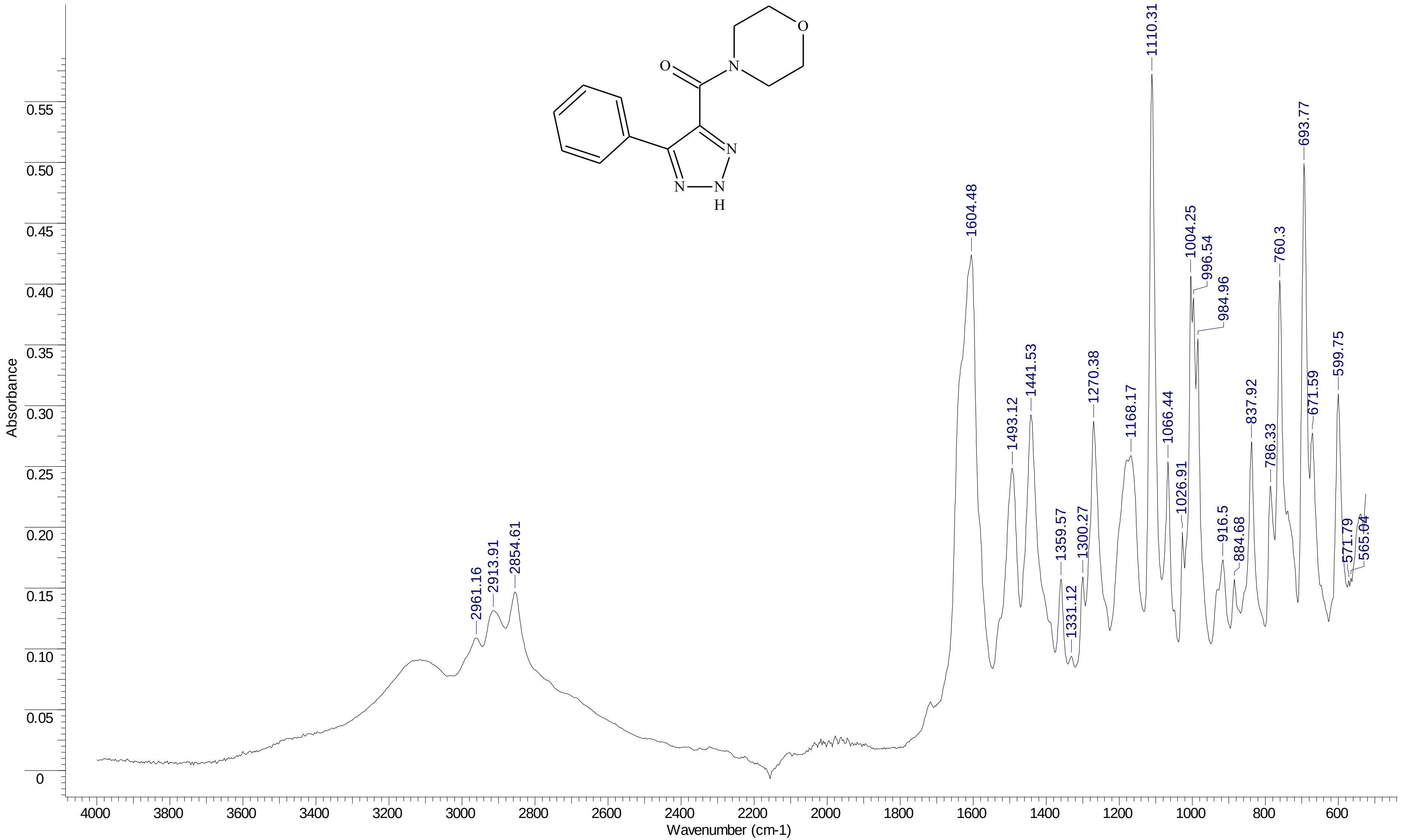

FT-IR spectra of **6**

8 Nov 2022

| | | | | | | | |
|---|---|---|---|---|---|---|---|
| ***Title*** | 腴 玎脲怦觇? 绿 -2448-7 (窝 湾 磬殇扃觐) iD7 | | | | | | |
| ***File Name*** | C:\DOCS\BM\IR SPECTRA\08-11-2022_13-23-31\腴 玎脲怦觇? 绿 -2448-7 (窝 湾 磬殇扃觐) ID7.SPA | | | | | ***Date Stamp*** | 08 Nov 2022 09:33:50 |
| ***Date*** | 08 Nov 2022 13:56:40 | ***Technique*** | Infrared | ***Spectral Region*** | IR | ***X Axis*** | Wavenumber (cm-1) |
| ***Y Axis*** | Absorbance | ***Spectrum Range*** | 525.0250 - 4000.1229 | ***Points Count*** | 7209 | ***Data Spacing*** | 0.4821 |

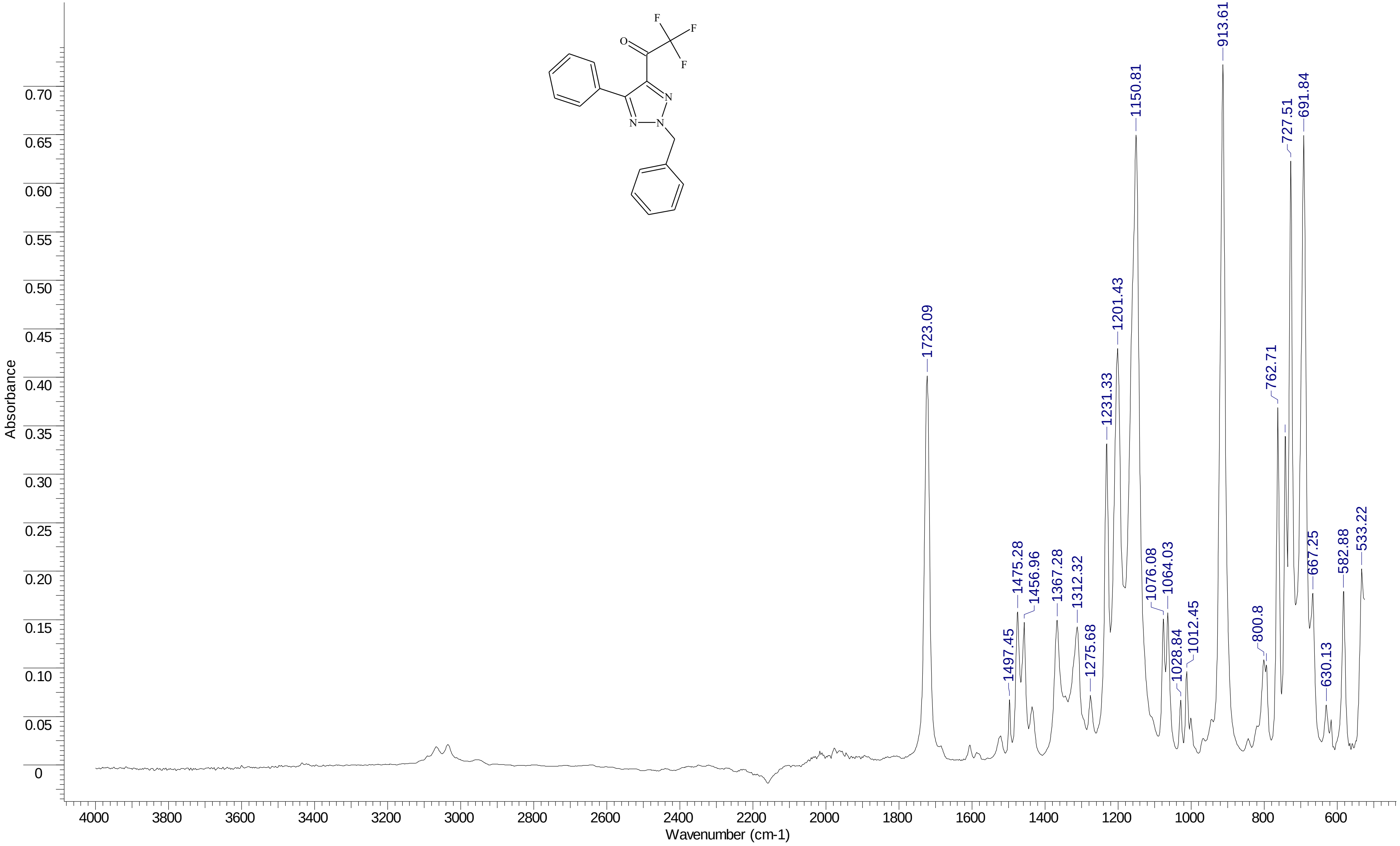

FT-IR spectra of **7**

8 Nov 2022

| | | | | | | | |
|---|---|---|---|---|---|---|---|
| ***Title*** | 腆 玎脲怦觇? 绿 -2461 (窝 湾 磬殇屙觐) iD7 | | | | | | |
| ***File Name*** | C:\DOCS\BM\IR SPECTRA\08-11-2022_13-23-31\腆 玎脲怦觇? 绿 -2461 (窝 湾 磬殇屙觐) ID7.SPA | | | | | ***Date Stamp*** | 08 Nov 2022 10:00:55 |
| ***Date*** | 08 Nov 2022 13:56:38 | ***Technique*** | Infrared | ***Spectral Region*** | IR | ***X Axis*** | Wavenumber (cm-1) |
| ***Y Axis*** | Absorbance | ***Spectrum Range*** | 525.0250 - 4000.1229 | ***Points Count*** | 7209 | ***Data Spacing*** | 0.4821 |

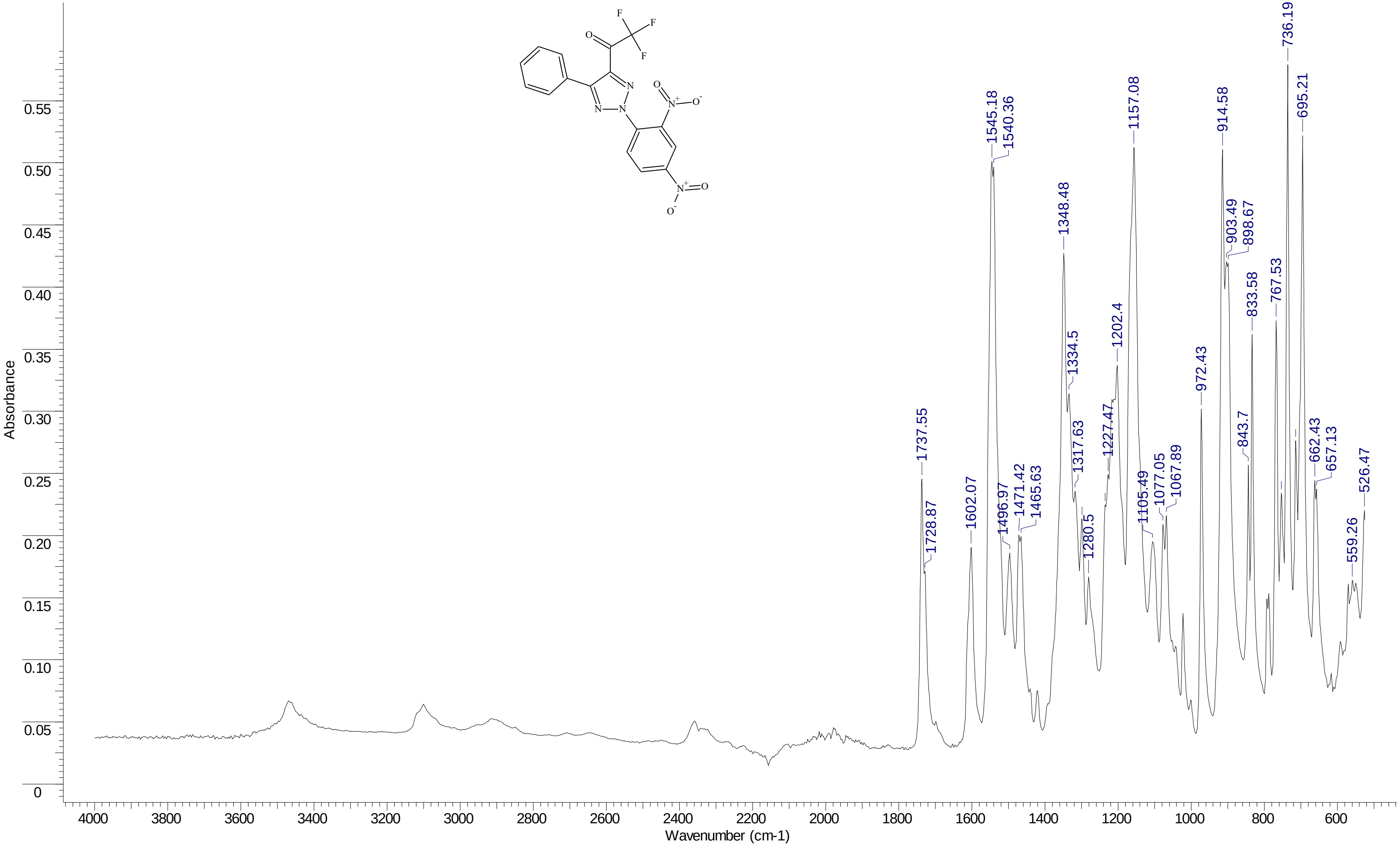

FT-IR spectra of **9**

8 Nov 2022

| | | | | | | | |
|---|---|---|---|---|---|---|---|
| ***Title*** | 腆 玎脲怦觇? 绿 -2470 (窝 湾 磬殇屙觐) iD7 | | | | | | |
| ***File Name*** | C:\DOCS\BM\IR SPECTRA\08-11-2022_13-23-31\腆 玎脲怦觇? 绿 -2470 (窝 湾 磬殇屙觐) ID7.SPA | | | | | ***Date Stamp*** | 08 Nov 2022 10:14:22 |
| ***Date*** | 08 Nov 2022 13:56:38 | ***Technique*** | Infrared | ***Spectral Region*** | IR | ***X Axis*** | Wavenumber (cm-1) |
| ***Y Axis*** | Absorbance | ***Spectrum Range*** | 525.0250 - 4000.1229 | ***Points Count*** | 7209 | ***Data Spacing*** | 0.4821 |

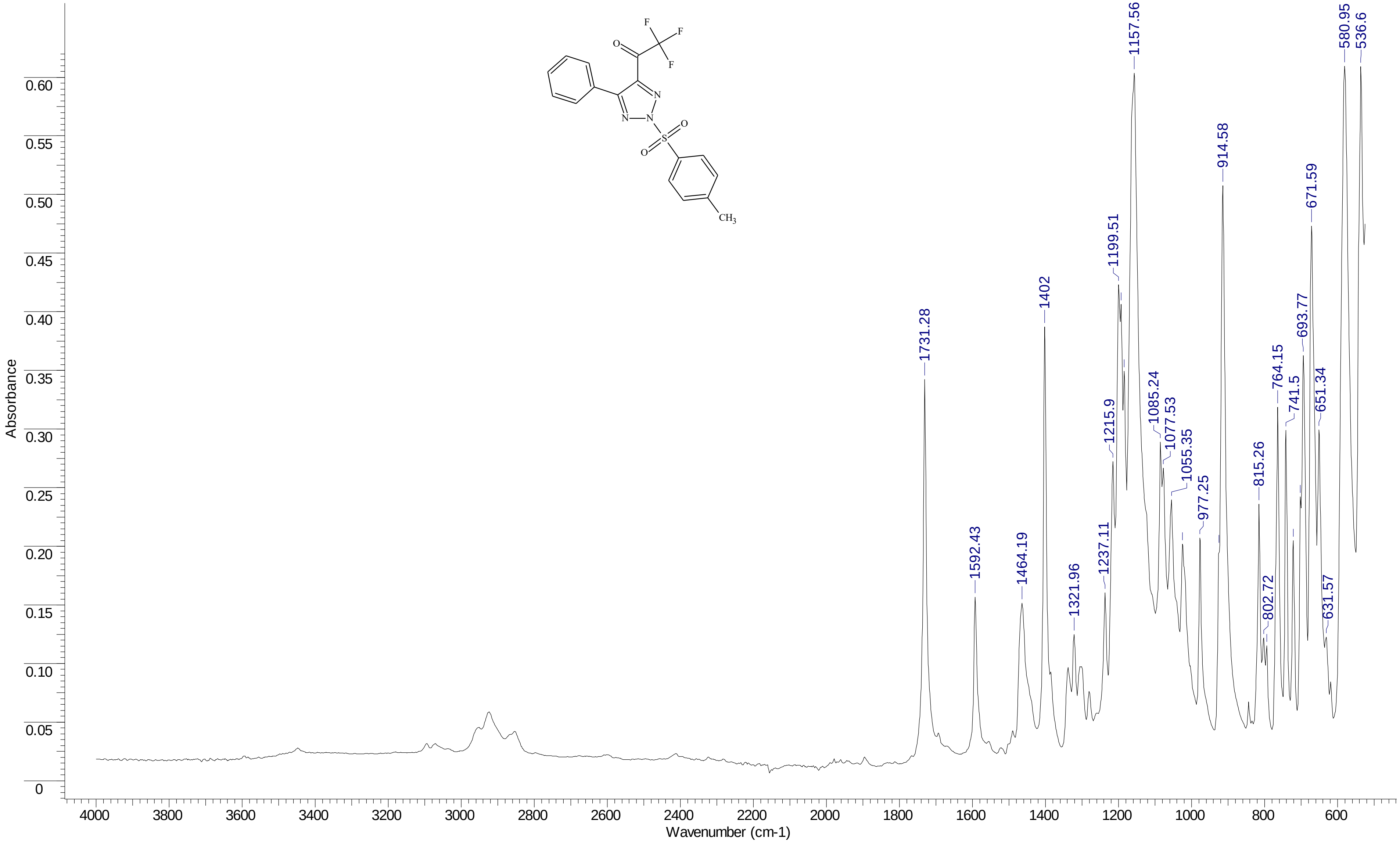

FT-IR spectra of **10**

8 Nov 2022

| | | | | | | | |
|---|---|---|---|---|---|---|---|
| ***Title*** | 腆 玎豚怦觇? 绿 -2505-2 (窝 湾 磬殇屙觐) iD7 | | | | | | |
| ***File Name*** | C:\DOCS\BM\IR SPECTRA\08-11-2022_13-23-31\腆 玎豚怦觇? 绿 -2505-2 (窝 湾 磬殇屙觐) ID7.SPA | | | | | ***Date Stamp*** | 08 Nov 2022 09:51:38 |
| ***Date*** | 08 Nov 2022 13:56:38 | ***Technique*** | Infrared | ***Spectral Region*** | IR | ***X Axis*** | Wavenumber (cm-1) |
| ***Y Axis*** | Absorbance | ***Spectrum Range*** | 525.0250 - 4000.1229 | ***Points Count*** | 7209 | ***Data Spacing*** | 0.4821 |

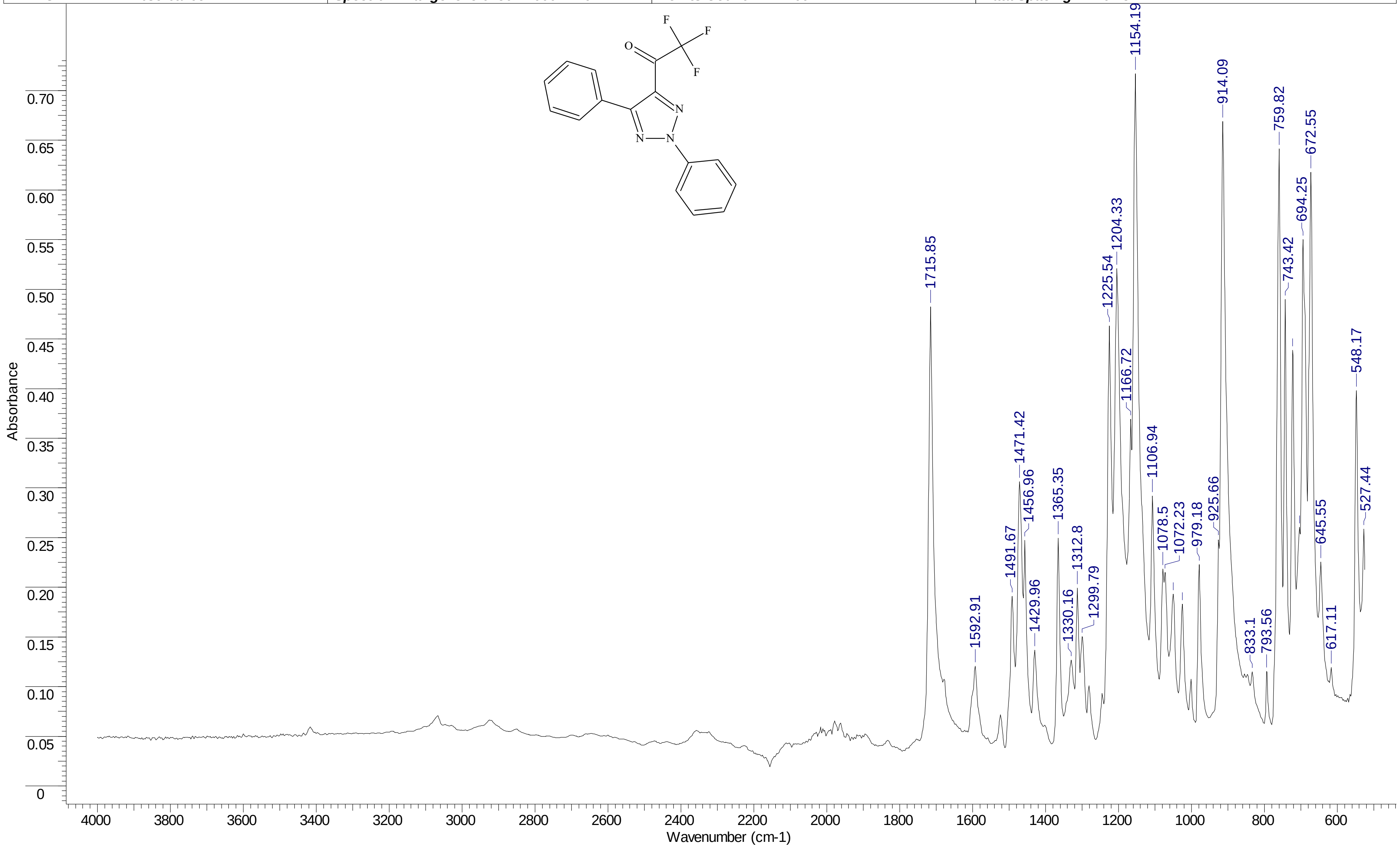

FT-IR spectra of **11**

## Computational Details

Geometry optimizations were conducted at the DFT level using the Perdew-Burke-Ernzerhof (PBE) functional. Valence electrons were treated using a TZ2P basis set. The innermost electrons of non-hydrogen atoms were emulated using effective core potentials ECP-SBKJC. Stationary points were characterized as minima (i=0) or first order transition states (i=1) by calculations of normal modes of vibrations at the same level. Single point energy calculations were done using the long-range-corrected LPBE density functional, a scalar-relativistic approximation, L1 wavefunction, and density-fitting basis sets implemented in PRIRODA program.[10,11] Solvent effects using CPCM[12] approach and D4 dispersion corrections were calculated using PBE functional and def2-TZVP basis set using Orca 5.0.3 release. Optimized geometries are collected in separate .xyz file. Utilization of long-range-corrected functionals, combination of explicit and implicit solvent models, dispersion corrections for correct modeling of chemical reactions in condensed phase is comprehensively reviewed by Grimme et al. recently.

In modeling of the azide formation under non-acidic conditions we used combination of explicit solvation model to emulate first solvation shell of sodium cation with four ethanol molecules and CPCM approach for the outer shell. Modeling of transformation of neutral alleneole **D** was performed using three explicit ethanol molecules for correct description of hydrogen transfer reactions mediated by solvent molecules.

**Table S3.** Calculated energy parameters ($E_t$ = $E_{Total}$(LPBE/L1) a.u.; G = G(PBE/TZ2p) kcal/mol; $E_s$ = Solvation energy (PBE(CPCM)/def2-TZVP) a.u.; $E_d$ = Van der Waals Correction $E_d$(PBE(CPCM)/def2-TZVP) a.u.; G = $E_t$ + G + $E_s$ + $E_d$ kcal/mol).

| Molecule | $E_t$ | G | $E_s$ | $E_d$ | G |
|---|---|---|---|---|---|
| A | -1706.52455 | 229.4 | -0.03466 | -0.05853 | 0.0 |
| TS-AB | -1706.51818 | 232.8 | -0.02839 | -0.06145 | 9.5 |
| B | -1706.55538 | 232.9 | -0.03108 | -0.06295 | -16.4 |
| TS-BC | -1706.53316 | 234.2 | -0.03356 | -0.06484 | -3.8 |
| C | -1706.65590 | 236.2 | -0.03263 | -0.06465 | -78.2 |
| | | | | | |
| D | -1389.41392 | 196.1 | -0.01544 | -0.04982 | 0.0 |
| TS-DE | -1389.38577 | 194.1 | -0.02066 | -0.05508 | 9.1 |
| TS-DG | -1389.38029 | 195.5 | -0.01699 | -0.05163 | 18.5 |
| E | -1389.41885 | 193.9 | -0.02226 | -0.04988 | -9.5 |

| | | | | | |
|---|---|---|---|---|---|
| TS-E3 | -1389.38029 | 194.6 | -0.02364 | -0.05231 | 12.9 |
| 3 + $N_2$ | -1389.49533 | 185.6 | -0.01960 | -0.04757 | -62.8 |
| TS-E2 | -1389.37969 | 194.2 | -0.01879 | -0.05258 | 15.8 |
| 2 | -1389.49415 | 199.3 | -0.02084 | -0.05250 | -52.2 |
